# Supplementary material for: Genome-Wide Identification, Structure Characterization, and Expression Pattern Profiling of the Aquaporin Gene Family in Betula pendula
Source: Int J Mol Sci. 2021 Jul 6;22(14):7269. doi: 10.3390/ijms22147269 (PMC8304918; doi:10.3390/ijms22147269)
Supplement: Supplementary file 1 [file ijms-22-07269-s001.zip › Supplementary Files Betula MIPs/FigureS1.pdf]

Figure S1. Detailed *Bpe*AQP sequences (genomic, CDS and protein sequences) from *Betula pendula*.
All sequences are presented in their original version.
For the genomic sequences, the introns are in blue.
Codons “Stop” and “Start” are in red.
All sequences are classified according to their subfamily.
>**FXXK01000000.1** corresponds to NCBI information
>**Bpev01.c0000.g0000.m0001** corresponds to CoGe information
5 AQP subfamilies : PIP ; TIP ; XIP ; NIP and SIP

PIP1 – Plasmalemic Intrinsic Protein type 1

*Bpe*PIP1;1

>**FXXK01000191.1:**845000-855000 *Betula pendula* genome assembly, contig: Contig190, whole genome shotgun sequence
>**Bpev01.c0190.g0072.m0001**
Contig190 850,536-851,776 (1) ::
join(850536..850869,850961..851256,851339..851479,851684..851776)

>*Bpe*PIP1;1
AATATTTCTCGTTTGGGCCAACATCATCAAGCACAATTATTATGAGTAATCATACGTCTCCAGCTGATAATGCAT
GTGTATAATAAATAATTAAAGACTTAGTAGTGTGTGTATATATATATTGTAGCCAAGCTGAAAAGACTAAATTAA
TCACTTGGGACAATAAATTTCATCACTTTATCTTATCGTCTTAAGCACAACAATTTTTTAAACCCATAAGCTATT
GGTGTATTTGTGGGTGGCAGAATATAACGAATTTTCTAGATGGGCCGGGTATGCTACTTAAACTAGTGATTTCGA
ATGAGACATATCGTAGGGTCGGCAGTGCGTGGTTTGTAATTAGTCATCATCAGGCGGTGAGGGGCGGTCTCTTTT
TTTATTTTATTTTTTTGGTTTTGGTTTCTTTTTTATTTTTTATTTTATTTTATTGTAAAGATAAATTTGTAATTT
TACAACAAGGTGGCTGAGTTATCAGCCATGCCACGTGACAGTCGATTGGTTTTGATGTGAATTCTCGTTTGCATC
CAAAGAGTCTTGCCAAACAACAACAAAGAGGAGCACTTCGGACAAATAGTTCTTAGGAATTTGTTTAGTACTTTT
TGATACCCTAAAATCAGATTGGTATAATTAATTTCCCATTTTTTTATATTAAAACATTGTTCAACATTGCCCTAG
GGTTTTCTAATGTCCAAACATATTTCATTTTACTTTCGCTAACAAAGACTTAATACAATTCAAATTTTCAGCAAT
TTGTTACTCGTATTGTTATAAGAGTTCTTATGAAGCTCACTTGTCCAAATAGATAAGTGGATAATTCAAAATTTG
TTTTGACAGGTAAACTTTATATTAACTTTCTTGCTTTTGTTGTTCTAATTATTAGCAATCCAAACAGTAAAAAAA
TAGAGATCTCTATCTGACTTAATCTAAATTATAATAATTCGGAAACTATAGGATGTTCATAACTGTGATAGAAAT
TTCAGAATTCGACTTAAAGTTAAGACCAAACTTGAAAGCTCTTTAGGTATATGCTATTACAGCATACGCGTGCAG
GACCCTTTAAGTGCCACTATCACTTCCAACTCTCGAAGTGGTTCATCCAATCGTGCTTCGCCATCATTTCCAAAC
TTTGATTGAAAGTCAAGGATAATTGCAAGTTAAATAAAAAAGCAAAGTGAGAGTTACAAACAAAAAAGACAAGGA
CGCCCATGATGATTTTTGCAAGCCAAGAAGCCAGCTTTGATAAAGGTTGGTGGATGACTGAATACATGGATCGGT
CGGTGGGAGGTCACGACAATGCACAGTTTATGCTCTCTAAATTACTGATAAAATACCAAAATGAATTCAACAACA
CCTGCCAACCTTTGTCCCATATATACTTGCCCTACAAATACCCACACCTCACTTCACTCAAGTCTCAAACAAAGC
TTCCACAAAGCCTAAAGATAGTGAGTGCACATAGCCTTAGAGAGAAAGGAA**ATG**GAGGGGAAGGATGAAGATGTG
AGGCTGGGAGCAAACAGGTACAGAGAGAGGCAACCAATCGGAACAGCGGCTCAGAGCCAAGATACCAAGGACTAC
CAGGAGCCACCGGCGGCACCCTTCTTTGAGCCAGGGGAGTTATCATCATGGTCCTTTTACAGGGCCGGCATAGCC
GAGTTTGTGGCCACTTTCTTGTTTCTTTACATCACCGTTTTAACGGTGATGGGTGTCTCCAAGTCTCCCTCCAAG
TGTTCGACTGTTGGTATTCAAGGCATTGCTTGGGCTTTCGGCGGCATGATCTTTGCTCTCGTCTATTGCACCGCT
GGGATTTCAGGTAAGAATAAGTACATATTTTTTATTTGATCCGCCGGTGTGAAATTTTGAGAGGACTAATTCTCA
TTGTTTGTGTTGGGGTGGTGGTGCAGGGGGTCATATCAACCCTGCAGTGACTTTCGGGCTGTTCTTGGCAAGGAA
GCTGTCGCTGACGAGGGCGGTGTTTTACATAGTAATGCAGTGTTTGGGAGCCATCTGCGGTGCTGGTGTGGTAAA
GGGCTTCCAGAAAAACCAGTACGAGAGGCTTGGTGGTGGTGCCAATACCATCAGCTCGGGATATTCCAAGGGTGA
TGGCCTTGGAGCCGAGATTGTTGGCACCTTCGTGCTCGTCTACACCGTTTTCTCTGCCACCGACGCCAAGCGAAA
TGCCCGAGATTCCCACGTTCCTGTAAGCAAAATCTCTACGCAATGCGCATATATCTTTGTTTCTAATTATTAATA
ATGACCACCCAATTAAACTTTATGTTCAGATCTTGGCGCCATTGCCTATTGGGTTTGCTGTGTTTCTGGTTCACT
TGGCCACCATCCCCATCACAGGGACAGGCATCAACCCGGCAAGGAGTCTTGGGGCAGCCCTCATTTTCAACAAGG
AACAAGCTTGGCATAACCATGTAAGCCTTTTTTTTTTTTAATTATTATTATTATTATTATTTTTTATCATTCTTG
TCTATTACAAGTGAAAGTTTAATAGATTGAATTGGAAGAATTTTCTTCAAAATTCGAAAATTTGGGGGAAAATTT
TGTTCAATTTAAACCTGAGAACAGATGTTTTGCAGAATTGACTGACAGTTTCTTGTTAAATACTTCTGGTGCAGT
GGATTTTTTGGGTGGACCTTTCATTGGGGCAGCACTAGCTGCTTTGTACCATCAGATAGTGATCAGGGCCATTC
CATTCAAGTCAAAA**TGA**TTACATTATTGAAGCAATGACCTGCAAAATGGTTGGTTGGTATCTAAGTTATCCTATG

GCCTTTCGTTTTTACCAGGGTATTTTTCTTGTATTTTCTTTATGTTTGTATGTAAACGGGCAAGCAAACAAGGTG
ATCTGGCGTAGGTTGTGGTGGTTTCTCCACTCGACTTCTCTCTCTATTGTGTTTGCTTGTTATAATATAAGTACT
GAAAATCAAGAATGGCAGTTGTATAAATATTACAGCATTCCAATATTTACAAATAATATCATTTTGAAGCTCTCT
GCTTCCCTTCAATGTACCCTAAATGAGGTAATTTTGAACACTACTCAAATGCAACAAGTGTCTAGATGTGTCAAT
TTTTGTGCACATAGTTTAGCTCAGTAAGCCGCTTCCAATCTTGTTTTTGAAAGTATTCCTATAAAATCTCCCATC
ATTTCTTTTATCTGGATCAGAAGTGAAAAAGATCTTGTCCTGTAACCCTTTTCTCCATTCAATTAGAAAAAAAAA
AAATGCAACAAGTTCAAGTCACAAACCCTCAACAAATAATAGACAACAAGAATAAGAACATAAATGAGTCCCGAA
AAGAACAAAGTCAGCTTGAGATCTTGTTCAAAAATCAGCTCTAAATATGAAAATTATATGGAGTGCAAGAGCCAA
AAATTAATGTTACACATGAGTACAATCACTACAAGAATATAATCATAATGAAAATAAGGAGCCAAAAAAAATGTA
ATCAAGAGCCTCTGTAATGACGGCTGAAACCGTCATTTGAACAAAAAACGTGTTATTATAGCATATTTTTAGATT
ATCATTATAATGCAAATAAAAAATCCAAGCAAAAAAGCTCCAATATCATTATACACCATTTTTTTAACACGAAGG
TTATATTTGGTACGTGAAATGACTATTCCATTAAAAAAAA

>*Bpe*PIP1;1

ATGGAGGGGAAGGATGAAGATGTGAGGCTGGGAGCAAACAGGTACAGAGAGAGGCAACCAATCGGAACAGCGGCT
CAGAGCCAAGATACCAAGGACTACCAGGAGCCACCGGCGGCACCCTTCTTTGAGCCAGGGGAGTTATCATCATGG
TCCTTTTACAGGGCCGGCATAGCCGAGTTTGTGGCCACTTTCTTGTTTCTTTACATCACCGTTTTAACGGTGATG
GGTGTCTCCAAGTCTCCCTCCAAGTGTTCGACTGTTGGTATTCAAGGCATTGCTTGGGCTTTCGGCGGCATGATC
TTTGCTCTCGTCTATTGCACCGCTGGGATTTCAGGGGGTCATATCAACCCTGCAGTGACTTTCGGGCTGTTCTTG
GCAAGGAAGCTGTCGCTGACGAGGGCGGTGTTTTACATAGTAATGCAGTGTTTGGGAGCCATCTGCGGTGCTGGT
GTGGTAAAGGGCTTCCAGAAAAACCAGTACGAGAGGCTTGGTGGTGGTGCCAATACCATCAGCTCGGGATATTCC
AAGGGTGATGGCCTTGGAGCCGAGATTGTTGGCACCTTCGTGCTCGTCTACACCGTTTTCTCTGCCACCGACGCC
AAGCGAAATGCCCGAGATTCCCACGTTCCTATCTTGGCGCCATTGCCTATTGGGTTTGCTGTGTTTCTGGTTCAC
TTGGCCACCATCCCCATCACAGGGACAGGCATCAACCCGGCAAGGAGTCTTGGGGCAGCCCTCATTTTCAACAAG
GAACAAGCTTGGCATAACCATTGGATTTTTTGGGTGGGACCTTTCATTGGGGCAGCACTAGCTGCTTTGTACCAT
CAGATAGTGATCAGGGCCATTCCATTCAAGTCAAAATGA

>*Bpe*PIP1;1

MEGKDEDVRLGANRYRERQPIGTAAQSQDTKDYQEPPAAPFFEPGELSSWSFYRAGIAEFVATFLFLYITVLTVM
GVSKSPSKCSTVGIQGIAWAFGGMIFALVYCTAGISGGHINPAVTFGLFLARKLSLTRAVFYIVMQCLGAICGAG
VVKGFQKNQYERLGGGANTISSGYSKGDGLGAEIVGTFVLVYTVFSATDAKRNARDSHVPILAPLPIGFAVFLVH
LATIPITGTGINPARSLGAALIFNKEQAWHNHWIFWVGPFIGAALAALYHQIVIRAIPFKSK*

*Bpe*PIP1;2

>**FXXK01000171.1:**510000-525000 *Betula pendula* genome assembly, contig: Contig170, whole genome shotgun sequence

>Bpev01.c0170.g0038.m0001

*Bpe*_Chr14 5,426,780-5,430,337 (-1) ::
complement(join(5426780..5426875,5426970..5427110,5429613..5429908,5430010.
.5430337))

>*Bpe*PIP1;2

TAAAATATATTTTATGTCAAAATTTAAAGTTGGCCTAACAATGTACATTATATTATATTATTTAAATTATTTAAA
AGACGTATCAATATTAAAGGCATGAAACTTGTTAGATATATAAATTTTAAATTTTGACCATTAAATAAAGGTTAT
ATCTATTTCAATTCGAACGAAAAAGATCTTGTTTTGTGAGTTTATAAAAGACTAGTAATTAAAGTTATAGTAATT
TAGTAACTGTTTTTTTTTTTAAATTTTTGGTTAAAAAAAAAAAGGGTGAGATTTCTCATTTTCCCTGGTTAAAAAA
CTTGAATGCTATGATGGCCAACTTCTCTGGAAATGGACCCTAAACCCTAAGTACGATGCTAGAACGTTGCAAAAG
TGTCATACATAGCCGTTGCCACGTGCTTGCACAAAGGGTGTCGCTTTCCTAACGGTCACAAAACGGCCACTTTCT
CTCAGCCATTCGATTTGCTTTCCGTTTTCTCATTATTGTGTGGCTGCGATTGCTCCAAGTCTCTATCGTTGAAAC
TTGAAAAGTAATCCCGTTACTTGGCAAAAAAGGGCAAAAATATATTTAAAAAAAAAAAGTTACACAAGAAAATAA
AGAGAAACAGTCTACACAAGAGAAGATCATTTCCCCTATTAAGTAAAAGGTCAAAAAACAAAAAACAAAAGATTT
AATATAAGGTTTAAACTTTTCATAACATGTTAAATTATTATTTATTTTAAAAATTTAAAAATTTTTTTTTTAAAT
AATTTAATTAAGATTTTAACATAGAATATTTTAAGTAAAATTAGATAAACAACCCATTTAGCTAAGTTGTGAACC
CATTAAAGTTAATTAGGAGTTGGGGCTACAAAAAAAAAAAGGGCTGCAAACAGTCCCAGAGCAGAAAATGAATTC
AGCTCAGGAGTAACATCATGATAAAGCATAACCCCATAACTTGGTCTTAAAAGTCCAATCCATTTTCTTCTCTTC
TCTTTTTTTGTGTTTTTTTTTAGTCCTTGTAAATGTGATATAAACCACTAATAAAGGGCTTTGTTAATATTATTG
AGAAAACCCTCATATTATTGAACCCCATCATGGATGCCATGGATTAAGTATATAATTCTATTTTTGAGTTAAAAA
CAAAAAGAATTGACCTTCTAATCCAAAAAGAAAAAAAGAAAAAAAGAAGAGAAAAGAGAGAAGAATAAAGAAGTA
AGCTTTCATTCTTTTTTCTTCGCCTGAATGAGTGACTTTGGGAAAAAGGCAGGAGGGGCAAGCAAGCTTTCACTT
CCTTTAAGCAAACACACTTTATAACAAGTTCTGAAATTCACTGAGCTTGACCACATGTGACCACAACACCTTTAT
TTTTGGACCTTTTTTTTTCCACAGACATTCTTTAATTAAAGATTAGGTGAATGATCAAATGGCCCAGAACAGCTA
ATCAAGTTCATCAGAATTTCAAAATCCATCAAGAGACAAAATAATCATAAAGTATGATACCTACAAAAAAAATAA
AAAAGTATGACACTCTGTAGAGGATAAGAACAAAAGAAAGGCACCAACCAGAACCTGTCAACATCCCTCTGCTCT

TTCCAGAGCAATCCCCTATCCCCTAATACACTTAACTAAAAAGCAAAAAAATAATGACCAAAATTTATTAGAAAA
ATAAAAAAGACAACCCATTTTTGCTTTCAGCTAAATAATACACTCCCCCATCTATAAAAACCCACTTTAGCTTCA
CCCTTCTCCCCACTTCACAGCCTCTGTTTTTGAGTGTGCTGAGAGAAAAATATCATCTTTTTGAAGAGAGAAAGC
TCAGTGAGGTAGTGAACAGTAAAACC**ATG**GAGGGAAAGGAAGAGGATGTTAGGCTTGGAGCAAACAAGTACACAG
AGAGGCAGCCGTTGGGCACAGCAGCTCAGACAGACAAGGACTACAAGGAGCCACCTCCAGCTCCTCTGTTTGAGC
CAGGGGAGCTCTGCTCATGGTCCTTCTACAGGGCTGGGATTGCTGAGTTTGTGGCCACTTTCTTGTTCCTCTACA
TCACCGTCTTGACTGTCATGGGTGTTAAGCGCTCTAGCAGTGTTTGTGCTTCTGTGGGTATCCAAGGAATTGCTT
GGGCTTTTGGTGGTATGATTTTTGCTCTTGTCTACTGCACTGCTGGTATTTCAGGTAAAATAGCTTCCCCTGAAT
CCCCTCCTCTCTCCTTTTTCTGGGTTTTTTTTTCTTTTTCTTTTTCTTTTTTTATAAGTTAAATCAATTTATATT
AACAGGTGGACACATCAACCCGGCTGTGACCTTTGGACTCCTCCTTGCGAGGAAGCTCTCCCTCACAAGAGCTGT
ATTCTACATAATCATGCAGTGCCTTGGAGCCATCTGTGGGGCTGGTGTTGTGAAGGGGTTCGGGAAGACCCCGTT
CGAGATTAATGGTGGTGGAGCCAACGTTGTGAACCATGGCTACACCAAGGGTGATGGGCTTGGTGCTGAGATCGT
TGGCACCTTTGTCCTTGTCTACACTGTCTTCTCTGCAACTGATGCCAAGAGAAACGCCAGAGACTCTCACGTCCC
TGTATGTATTCTCTGCTCTTTCTTTGTATAACTTTATTTTTTGTGATCTTTGCAAGTGCTTTTGATAGCTTTTGG
AATTTGCAGGTTGATTTTTCATGATTTCTTCTTGTGCTATTTTACTTGACTTTTTATAAAGTATTTGTTTTTCTG
CAGGTTTTCCTCTTTTCTTGTGCTATTGCTTGCTAGTTTGGTTGTTTGGCTGTATTTTCCTTGAGTGGGTATTGT
GGTGGGTTATTTTTGGGACCTGTTGCTAAAAGTTGATTATTTGATTGATTTCCTAACTCTCCCTTTCCCTTGGTT
TCCAAAATAAGCTGGTTTTTATTTAACAAACTTAACAAAATAATTTTTTTTTTTTTTCAAAAACCAAGAAAAGG
TTTCTCTTTATCGAAGTAAGCTATATATATAGTCTGCATTTTTTTTTAATAGTATTTTTTTCTGATTTTTTGAAC
TTTTGTTCTTCTTTTTCTTTTTATTATATATATATATATTCACGGGGACTAGGGTTGCACTTTTGATCTTTCTCA
TGATATTGAATTACTTTTAAAAAAAATTGTGCTGAGAGAGAGAGATGTCTTGGGTGCTTTTAGGTGAGACCTCGT
GGGTTTGAGACCCCCACCACATATTAAAGTATAAAACAATTTGTCGTGGCTCCTTTAGGCCTTTGGTTTCTCTAC
TTTCCCTTATCCTCTGGTTCCTTCCTCCGCTTTGGATTAGGAAAAGAGAAATATTGCTAGGTTTACTATTTTTTT
TTAAAGATATGCTCGGTGTCGTCCAACTCTGTCTTACTTGTGAGTAATTTTTATTTTTTATTTATTTTTAAATGA
AGTCAATTAGACGAGGTTAGACGACTTGGTAGGATACCAATCATATCTTTTTTTTTTTGGGGCAATGTTAGTTGT
ACAATCGAGTTGTCCAACTTTCTTCGTGTTAACATGAAGAAAGTTGGACCAAGTTGGACACTTCGGTTGTACAAC
TAGCATTGCTCTCTCTTTTATTTTATTTTATTTTTTCAAAAAGTTGGTTTCCAAGTAATGTGTCATTATTTCATC
ACTGCTTCTATTTACTAAAGATAAAGGCTATGTGTCTATCCATATTGTTTGTCTTTATCCATCCAATTTACGTAT
TTTTGTCTAAAAAACAAAGATTGATGGACAAAGTGGATGGACGCTTGCTTGGCCTTTCTCTTTACTGAAACAGTA
GATCACATTAGATGGTGACACATTATTTTTGAACTAACTTTTTGAGAAAAAAAATTTGAGAAAACTAGCATTTCT
CGAAGGGAAATATTGTATACACCTAGCCAGCTCGATCAATTCTTTACCATGGATTTTAGGGATTAACGTTAATTT
GTGGGTAACACCTTGCAAATAACAGTATCTGTTCATGTGCCAAAGAAATGGATTGGAACTTAGCTCATCATTGAT
GTGTCCCAATCTATTGTATAAAAATGAGCTTGGTGAGTGTCATCCATTTGGTTTTTGTTTAAATCATAGGGTTAA
TTTTCTTTATCAAAGTGATTATTTCCGCAATATATCATATGAGATTCATAAAACAATGTGTTCAAATCCTTTTTC
ATGCTGACATGGCAGGAATTTTAGTGGAAGCTATAAATGTTGTCCTCCTGACTTTGTTCTTATAGTATTTAATTC
CTGCCATTTCAATGTGAAAAAGGATTTGAGCTAATTGTCTGATGTTTTTCCACCGATAACTTTATCATGCAAATA
TTCACTTTGATCAACTTTATGTTGATATATTGTCAAAGGATTAAGATAAGTTTTTTTGTGCACTTGATGTTCTAA
GTGGGAGTAGTTAGATAGATTGTAAGATTTTACTCGAGAAACATAGAATACATAGAAGAAATGTGCGAGAGGCTT
AGCCTTAGCATGAAGGAATTAAATTGAGGGAGCTGGGTTGTACTTGTGTGGAAGATACAAAATGAGTTATCCTTA
TGGTTGATGTTAGAATATTACCATGATTGTAATAGAATATTCTCATAATATTACTATGGGCCTAATGTTGGATCG
GTACACACGTGATGATGAGTGAACTTGCATGGGAAGTTGCTGGTTCAAGTGACACGTGAAGATTAACATACCGAG
AATATTATGAAAATAATCTATTACAATTATGGTAATATTTTATCTAGATTGGTAACAATTTAACAGCTGAGCTAC
CTTCTTGGCACTTTTGGTGAGCATGGCTTCTTTACAATTTCAAATTCCAATCATGGACATATATATCAACTTATT
TGTCCATCTATCTCTGTCAATCACATGACATGTGGATGGTTTATGATTGGACTGAATTGTATATCGAAGGAGCCC
TTCCCTCCCTCATGTGTTCTTCAACCTACCATATGTATGTGTCACATTTGTTTTTAGCAGTTTTTCCTGTGCTTT
TTCTTTGTTTGAATTGAATTATTATACTGAAAAGCAACAAAAATTACTATGCATCAAATCAATATTCTTGTTCTG
ATGTTGTATTTTTTTATCATGTCTGCAGATTTTGGCTCCACTTCCCATTGGGTTCGCGGTGTTCTTGGTTCATTT
GGCCACCATCCCCATCACAGGAACTGGTATTAACCCAGCCAGGAGTCTTGGAGCTGCCATTATCTACAACAAAGA
CCACGCATGGGATGACCACGTAAGCATTTTGATGAAAAACCATTTATAATTATCTTGTGCTTTAAGTGGATGAGA
TTTATAATAATAACAATAATCTCTCTTTGCTTGACCAGTGGATCTTCTGGGTGGGACCCTTCATCGGAGCTGCTC
TTGCTGCTGTGTACCACCAGATAGTCATCAGAGCCATTCCTTTCAAGGCCAGGGGT**TAA**GATTCTTCTTGAGGCT
TGGGGATAATATCAAACTATCTCTCTGTTTTCTTCTTTTTTCTTCTTCTGTTTTACTTTGTTGTGTATTTTCAGA
GCCATTCCTTTCATGTGTTTGGTGTTGAGGAGTTAATTATGTGTGTAAATTATATGTCAAGTTTGTGACAGCTTT
TAATGGAGAATTCTTTAATCTTTTATGCTACATTCCCCCAGCTTTTAATGGGAATACCACTTCATTACGGGTGGG
ATGGTTAGTTAGGCAGGTCATTCAATGTTGAATTGCTCTTTCTGTAAGAAGAAACCATGAAACAAGCAAACTAGA
TGTTGAATTTATTGCATTTTTAACAGCCTATTGTGAAGTAAGTTTTAAGAAATGCATAAGGCTTCATCTATCCCT
GACGTCCCTGGGAAAGCCTTATCACACCAAAGTTATCACACCATGCGTTATTACTCTACAATGTTAGTCAAGTTA
TAATTGAAGTTTTTTAAAATTATTTATAAAGGTTAATTTTACATAGTGAAGAATCAATATATAACTTAACACTCT
GAATTCCTCAACAATCTACCTGTTCTATTAATAGAATCATGTGTTCTAAAGATGTATATCGGTTTAATAAGTCCG
ACGAAGGACCAACCCTATTTGAAAGAAGTTTTGAGAACATGCTTAATGAATTCATCTGCTTTTAAACAAGCAAAC
AAAGCTGGGAACTCTGAGATGGGTTTGGGAATATACTTCATTGGCTTTGTCCCCTTTTCTTTTTTGAATTTACTT
AGCCCTCAATCTACGTTATATTTTTCCCACTCCCACTTGATGATCAAAGACTTTGTTAAGAAGGAAGTAAGAAAA
AAAATACTAATGATCTAGCATGTGAAAGAGTAAAAGCATTTAAGGTGGTTGAGATGTTGACATGAAAGATGATGT

GCACCCATCTCAAAAAAAAGAAAAAAAAAAAAAAAGAAAAAAAGAAAGATGATGTGCACCTACCCCTTCCAAAAGG
GACACCCGTCACACCCCGCCTGTGATGGGTTGACAATTATCCCCTCAATTACTCCAAGATGCAATACCTTATTGG
ATGAACCACCACTAGTCCTTTGGAAGCTCTCGAATTGACAAGCTCCTAACATATTTGATGACTTTATGATAGGCC
AAAAATAGACCCTTGGCCAAATTCGGGTTGGATAGCGGATCTTTAGTATTTGGGTTAGGATTATAAAGCTCAAAC
TTCAAATTTTAACCGAAAGTTAGAGCAATGAAACAGAACAAAGAACAAAT

>*Bpe*PIP1;2

ATGGAGGGAAAGGAAGAGGATGTTAGGCTTGGAGCAAACAAGTACACAGAGAGGCAGCCGTTGGGCACAGCAGCT
CAGACAGACAAGGACTACAAGGAGCCACCTCCAGCTCCTCTGTTTGAGCCAGGGGAGCTCTGCTCATGGTCCTTC
TACAGGGCTGGGATTGCTGAGTTTGTGGCCACTTTCTTGTTCCTCTACATCACCGTCTTGACTGTCATGGGTGTT
AAGCGCTCTAGCAGTGTTTGTGCTTCTGTGGGTATCCAAGGAATTGCTTGGGCTTTTGGTGGTATGATTTTTGCT
CTTGTCTACTGCACTGCTGGTATTTCAGGTGGACACATCAACCCGGCTGTGACCTTTGGACTCCTCCTTGCGAGG
AAGCTCTCCCTCACAAGAGCTGTATTCTACATAATCATGCAGTGCCTTGGAGCCATCTGTGGGGCTGGTGTTGTG
AAGGGGTTCGGGAAGACCCCGTTCGAGATTAATGGTGGTGGAGCCAACGTTGTGAACCATGGCTACACCAAGGGT
GATGGGCTTGGTGCTGAGATCGTTGGCACCTTTGTCCTTGTCTACACTGTCTTCTCTGCAACTGATGCCAAGAGA
AACGCCAGAGACTCTCACGTCCCTATTTTGGCTCCACTTCCCATTGGGTTCGCGGTGTTCTTGGTTCATTTGGCC
ACCATCCCCATCACAGGAACTGGTATTAACCCAGCCAGGAGTCTTGGAGCTGCCATTATCTACAACAAAGACCAC
GCATGGGATGACCACTGGATCTTCTGGGTGGGACCCTTCATCGGAGCTGCTCTTGCTGCTGTGTACCACCAGATA
GTCATCAGAGCCATTCCTTTCAAGGCCAGGGGT**TAA**

>*Bpe*PIP1;2

MEGKEEDVRLGANKYTERQPLGTAAQTDKDYKEPPPAPLFEPGELCSWSFYRAGIAEFVATFLFLYITVLTVMGV
KRSSSVCASVGIQGIAWAFGGMIFALVYCTAGISGGHINPAVTFGLLLARKLSLTRAVFYIIMQCLGAICGAGVV
KGFGKTPFEINGGGANVVNHGYTKGDGLGAEIVGTFVLVYTVFSATDAKRNARDSHVPILAPLPIGFAVFLVHLA
TIPITGTGINPARSLGAAIIYNKDHAWDDHWIFWVGPFIGAALAAVYHQIVIRAIPFKARG*

***Bpe*PIP1;3**

>**FXXK01001700.1:**4600-7500 *Betula pendula* genome assembly, contig: Contig1699, whole genome shotgun sequence

>**Bpev01.c1699.g0001.m0001**

Chr *Bpe*_Chr5 1,627,018-1,628,297 (1) ::
join(1627018..1627354,1627572..1627867,1627959..1628099,1628205..1628297)

>*Bpe*PIP1;3

AATCCTTAAGCTTGTATACTAAGATTTTCTTAATTTTAAGTAATGTTAAGATGATTTTTTTAAGTGAAATATCTT
AGTATAAATAGTTAAGGAATACGTTACACATCTATTATTTATTTATTTTTTATTCATTTTTATATAAAAAAATAT
TTAAATTTAACATTGAATTATAAGATTATACATATGATCAAGGACGGAGGGAAGGGGGGCTGGTAGGGGCCCGGC
CCCCCAACAAACAAGAAAAAAAAAATTTCAAGCTTAATTACAAGTTAATTTTGTTTTTTTTACCTTTTGGCTCATA
CCCAAATTTTTTTAACCCTACCTTTAAAATTTTTTTAATTTTGATTCATACTTATTTTATCTCTCGACTCCGTC
CCTGCATATGATCTCATAAATTAAATAAAAAATTTATACATATTTTTAGAAGCGTAAAAATTAATAATAAAAAAA
ACAATCATTTATCAACCAGTTAAATCTAACAAAAACAAATTCGTACACCATGTGATGATTTATGAAAGAGGAGCA
AAATCAAGAATTGCCTATTTCTTATTAGACGTAAATGTATCGTGCAATCTTTGTTAGCCACGTATGCAGTATGCA
ATATGCAACATGAGCTGAATTGATGTGATGTAGCCAGGAACCGACCGACCCCATGAAACTGACTTGGACTATACT
ATTACGAAAGCTGGAAAACTAAAACAGTAAAACCCAACATTTTCCTCGTTCTTTAAGCACATGGACGGTGGACTC
ATTCATTCAAACTCACAGATGTATGGACGAGGTTGTGCCAAGTAGGATAATCTCTGATGTCACTTTCAACAAAAC
ACGCGGGGGTATTGAAGTCCTTTAACATGAAAACTCAGAAAATGATTGGTCGGCCGCGAGATAACAAAAATAAAT
TCAATTAGAAATACCAACGAGGTCCCACCACCTCACCTGCTTAGCCCATGATGCCTTATATAATCCCACTACTTC
TACTCTCACCAGTCTCATCGGAGAGATCAAACGTCACTCACTAAATTACTAATCAACTTGAGAGAGAGAGAGCTT
TGTGAGTAGAGCTAAA**ATG**GAGGGCAAGGAAGAGGATGTTAGATTGGGAGCCAACAAGTTCCCGGAGAGGCAGCC
GATTGGGACGGCGGCTCAGAGCCAAGACGAGGGCAAGGACTACATAGAGCCTCCGCCGGCGCCGCTGTTCGAGCC
GTCTGAGCTCACATCTTGGTCCTTTTACAGGGCTGGAATCGCCGAATTCGTGGCCACCTTCCTGTTTCTGTACAT
CACAGTTTTGACTGTGATGGGCGTTGCTAAGTCCCCGAGCAAGTGCTCAACCGTCGGGATTCAAGGGATCGCTTG
GGCCTTTGGCGGCATGATCTTCGCCCTTGTCTACTGCACCGCTGGCATCTCAGGTTCGACACTGCAAAAACTGCA
CGTTATTTTGTTCTTTTTAGTTATTATGTTTAATTTCGGCTTTGACCCAAAAAAAACAAAAAACAAAAACGAAAA
ACGCCCACCGTGGGGCTCGAACCCACGACCACAAGGTTAAGAGCCTTGCGCTCTACCAACTGAGCTAGACGGGCT
CACATGATACAGGTTGATTGATGATGGTAATTATTTTAATGGCAGGGGGTCACATAAACCCGGCGGTGACGTTTG
GGCTGTTTCTGGCGAGGAAGCTGTCGTTGACGAGGGCGTTGTTCTACATAGTTATGCAGTGCCTGGGTGCGATTT
GCGGTGCTGGTGTTGTGAAGGGTTTCCAGAAGAGCCAGTACGAAATTTTGGGCGGTGGGGCCAACTTTGTGAACC
CTGGTTACACCAAGGGCGATGGCCTTGGCGCTGAGATTGTTGGTACCTTTGTTCTTGTCTACACTGTCTTCTCTG
CCACTGACGCCAAGCGTAGCGCCAGGGACTCCCACGTTCCCGTAAGCAATCTCACTCTCCCTCTTCCTCTTCCTC
TCTAAATATGATTTTTCTTTTTTTCTTTTGTTAACGATGGTGTATGTATTTGATCAGATTTTGGCACCTCTACCA
ATTGGGTTCGCGGTTTTCTTGGTGCACTTGGCTACCATCCCCATAACCGGAACTGGTATTAACCCAGCTCGGAGT
CTTGGTGCGGCAATCATCTACAACAGGGACCGCGCCTGGGATGACCATGTAAACCATCTGATTTCTCCCATCTCT

TAATTCTAAGAACCTATCACCTAATCAATGTTCTTACTCGTTACAACTTGTTTTGTTTTCATATACTTTTGTGTG
CAGTGGATTTTCTGGGTGGGGCCATTCATTGGGGCAGCACTTGCAGCTCTGTACCACCAGGTTGTGATCAGGGCC
ATTCCCTTCAAATCGAAG**TGA**TGATTGATCAGATCATACAACCCGTGATGTTCCAAGTTCAAGATCAAGCCAAGC
TTGTCGATCCAGTCTCTCCGTTTTTCTTTCCTTTTTTTTTTTTTTTTTTTTGTTTTATTTCTTTCTTTTCTTTC
CTTTTTCTGTGTGTATGTATCTGTACGTAAGATGTGTAAAAGTAGGCTAGTAGCATGCTTCTTTCAAGGGCCTTC
TTTTCCACTATCTTTTTTGTCCAAACGGAGGTTGTTGATGGCCCCATTTCTAGTGGGTGGGCTTTGTATGCTTC
TGCCAGCTTGTATTGTCTATCCAATAACAATGATTCTGTATGTTCAAATTGAATTA

>*Bpe*PIP1;3
ATGGAGGGCAAGGAAGAGGATGTTAGATTGGGAGCCAACAAGTTCCCGGAGAGGCAGCCGATTGGGACGGCGGCT
CAGAGCCAAGACGAGGGCAAGGACTACATAGAGCCTCCGCCGGCGCCGCTGTTCGAGCCGTCTGAGCTCACATCT
TGGTCCTTTTACAGGGCTGGAATCGCCGAATTCGTGGCCACCTTCCTGTTTCTGTACATCACAGTTTTGACTGTG
ATGGGCGTTGCTAAGTCCCCGAGCAAGTGCTCAACCGTCGGGATTCAAGGGATCGCTTGGGCCTTTGGCGGCATG
ATCTTCGCCCTTGTCTACTGCACCGCTGGCATCTCAGGGGGTCACATAAACCCGGCGGTGACGTTTGGGCTGTTT
CTGGCGAGGAAGCTGTCGTTGACGAGGGCGTTGTTCTACATAGTTATGCAGTGCCTGGGTGCGATTTGCGGTGCT
GGTGTTGTGAAGGGTTTCCAGAAGAGCCAGTACGAAATTTTGGGCGGTGGGGCCAACTTTGTGAACCCTGGTTAC
ACCAAGGGCGATGGCCTTGGCGCTGAGATTGTTGGTACCTTTGTTCTTGTCTACACTGTCTTCTCTGCCACTGAC
GCCAAGCGTAGCGCCAGGGACTCCCACGTTCCCATTTTGGCACCTCTACCAATTGGGTTCGCGGTTTTCTTGGTG
CACTTGGCTACCATCCCCATAACCGGAACTGGTATTAACCCAGCTCGGAGTCTTGGTGCGGCAATCATCTACAAC
AGGGACCGCGCCTGGGATGACCATTGGATTTTCTGGGTGGGGCCATTCATTGGGGCAGCACTTGCAGCTCTGTAC
CACCAGGTTGTGATCAGGGCCATTCCCTTCAAATCGAAG**TGA**

>*Bpe*PIP1;3
MEGKEEDVRLGANKFPERQPIGTAAQSQDEGKDYIEPPPAPLFEPSELTSWSFYRAGIAEFVATFLFLYITVLTV
MGVAKSPSKCSTVGIQGIAWAFGGMIFALVYCTAGISGGHINPAVTFGLFLARKLSLTRALFYIVMQCLGAICGA
GVVKGFQKSQYEILGGGANFVNPGYTKGDGLGAEIVGTFVLVYTVFSATDAKRSARDSHVPILAPLPIGFAVFLV
HLATIPITGTGINPARSLGAAIIYNRDRAWDDHWIFWVGPFIGAALAALYHQVVIRAIPFKSK*

***Bpe*PIP1;4**

>**FXXK01000028.1:**937000-947000 *Betula pendula* genome assembly, contig: Contig27, whole genome shotgun sequence

>***Bpe*v01.c0027.g0083.m0001**
*Bpe*_Chr5 22,880,015-22,885,763 (-1) ::
complement(join(22880015..22880110,22880210..22880350,22882759..22883054,22
885436..22885763))

>*Bpe*PIP1;4
ACAAAGAGAGAAAACTCAGAAAATATGCATTTAAAAAAAAAAAGAAGCAGATCAAGATTTTTTTAAAATAATTAG
AGTATTTTATTGTGTAGATTCTTTCAAATATTAATTATTGTTTTCAAATTAAATGTTGAGTCCGAAATCCAACAA
TAAGCTATGAGCGAAGGACTTTCGTTGTTCCTCACTTGTATCACTGGTGAGGGCATTGTGGTCCAATAAAAATGG
ACGATGAGAGAAGGGTGCGGAAGAAGTAAAAATGGGTGTGGAAACAGCACCTCCCATGATGGAAAAGTGGGACTA
AAAGAAGATGAAAATTGAAAAGGGCCTACAGAAAGATAAGGACAGCCCAGCATCTTGGCATTGTGACAGAGTATC
CCCGCTTTTCCTCATCCATCTTTTTAGCACTTTCCAGAAGCATCTCTCGACTTAACCCACAGAAAACCCATCTTT
TTAAACCCCGGGAATTGCAACCCTAAATGGTTATGTTTGGTAAAGGATAATCCGTTTGGCTTCTTTTATTTTTTA
TAAAAATTATTTTAAGAAATAATCATTTAAGGGTGTTTATTTGTTTTTTTTTCTTTTTAAGTTGAGAATTTTGAA
AAAAAAAAAACTTTAAAGGTATTTAAATTTAAGTAAATTTAATTTCTCAACTCAAGTTATTTTCATCATTTTAT
GTTAAAAATTAACATGCTGTGTCACGTGATTTTTCTGTTTAATTTAAAGTGCTTGACAAATGGGAATGTTTTTTT
CATCAATTCGAAATTTTTTTAGTTTCTCAACTCAGGATATTTTCATCAATTTATGTTAAAAATTAACAAATCCGT
GACCGTGTCACTTTTCATTTGATTGAACGTGCTCAACTAAGGAAATTGAGTGTGAAAATTGGTAGTTGGTGGGAG
AATGTGCAAATGAGTGATAATTAAGGGATTAAAGTGTAATTAATCTTAAAAAAACCCGGGTTGAAACAGTTGAAT
GAGATGTTAGTTTAAAGGGGGGAGGGGGGGCCAAGCTTTTGTACCATGATCAAGTACTGTTCAGTGACTACTATC
TTTTCCTTCTATATAATCATTCCACTCAGCTGTTGTTCCACATCTTCAAGCTTTAAGCTTTATCAGAGAGAAAGA
GAGAGAGAAAGAAAAGAGTTGCAGAAGAAAGAAGGAAAGAAAGA**ATG**GAGGGGAAAGAAGAGGATGTGAAAGTAG
GAGCGAGCAAGTTCCCAGAGAGGCAGGCCATAGGGACGTCGGCACAGACGGACAAGGACTACAAGGAGCCACCAC
CGGCTCCACTGTTTGAGCCCGGTGAGCTTCAGTCCTGGTCTTTCTGGAGGGCTGGGATAGCTGAGTTCATTGCCA
CCTTCCTCTTCCTCTACATCACTCTCCTCACTGTCATGGGTGTTTCCAGGGCTCCCAGCAAGTGTGCCTCTGTTG
GTGTTCAGGGCATTGCTTGGGCCTTTGGGGGTATGATCTTTGCCCTTGTTTACTGCACTGCTGGTATCTCAGGTT
AGACCCTCTCTCTCTCTCTCTGTTTGAATTTCTGGGCTTTTCTTTGTGGGTTTTTGTTTTTTTGAGAAGCTTTTG
TGGTTGTAGATGTTGAGAAATGTACGGATTTTCTTGGTTGCGAGTTGTTTGTTTGTTTGCCTTTTTTTTTTTGTG
GGATTTTGTTTTTTGAGAATTCTTTGTGGTTCTATATGTTGAGACATGTACAGATTTTCTTGGTTCCAAGTTGTT
TGTTAAAGGTGTTTGTTTCATGGGAAATGGGAAGAAAATAAACTTGTTATAACGAGGGTAGCTTTTTTAAGATGC
CATGTATTCCTCTTATGTCTTTTTAGAATCAGGGGGAGGGTTGGTCGTTTTTGTAATGAATTTTTCTTTTTTTTA
TTTGGCTGTTTGTTTGCTGATGAAAACTGAGAAAAAGAGAATAAAATGTATAGACTAAATGGGTTTTGAGGGATC

TGTTTTATATTTTGCCACTTTTTTTTTTTAACTGTTGAGTACGTTAGGTTTTATGATGGCTAATTCGATAACTAA
GTTTGGTCTAAGTTTAAGATTGTTAGTCAAATTGGTACCTCGGTTGGTGGGGCGTCTGAACGAGAAACTTATACA
AAGAATCAATAGTCTATATAAACCTCGGAGTGCAGGATTTTCCACAGACCCTGAGGCCAGACCTTTTGCATCCAT
ACGCCTTCAATTTTATCTCTCTTAGACTTTGTTTAAACCACACAAATTCTTAAAACCTTCAGGAATTCTGGGTTG
CAAGAGCAGTCTCGATAGAAGAATTGGCCAGAGTTCTTGAGCTACTATGGTAGAGACAAATGGGTTGTTTGTTGT
GGTACACATTAGGTTTAATTACAAAGGTTTTGTAAGAGTAAACCATGTAAATTTATATTTCTATAATGGATGAGT
CTCTTGGGTTTGGTTGTTCTAATGTGGTTTTACTTTTGAAGAGTTCTTTAAAAGGTTTCCACATTCTCACCAAAC
TTGTTGTGCTTGATTCTTTGGTTAACTTTGGTGATTGTGAAAATTTGATTGAGTTTGCTGGAAAAACACCCATGC
ACCCCATCTAGGTGTGTTTGGGGTCATTGGGCATAATTTCAGAGTATACGTGTTATCGTATAAAGATTGAGTCTA
TCTTGGAGAGGTGAGTGGTTAATATAACACATTTTTTTTTTTGATAAGTAAGAGTCAATTCATTAAAAGCGCAAA
AGCGCAGTCTTGGCACACAGGAAGTATACAAAGGACCGCAAGGGGAATAAAAATATACAAGAAAATCATTAAAGC
TTATCACTAGAGGAGTTAGATAAGCAGCTGTCCAAGAAAAGAGAGTGTGAAAAAAGAAAGACTTGAGCTCCTCAA
TCGATCTTTTTTTGTCTTCGAAATTACGATCATTTCGTTCCCTCCAAAGACATCACATGAGGCAAGATGGCACCA
TCTTCCACACAACAGCACTCTGGGAGCGACCACCAGTCCACCAGCTGGCAAACAGGTCTATCACATGAAGAGGCA
TAACCCACGATAACCCAAAACGGCTGAAGATAGTAGTCCATAAGGCGCTAGTAGCCTCATAACATAATTGGATCC
AAACCTATAGGCTTAAGCTTTTAACTTTTAGGTTGAGTGTTATTCATCAAAAAAAAAACTTTTAGGTTGAGTGTT
GTCCTAACATGCAATATTATGGTCTCATTATAAGGCTCTCCAAAGTATCAATATTCTTAACAAATGATATCAAAG
CCGATTGTTGTGTGCAGTATGGTGGAGTGGTGCAGGCACGTGATTGTTGAGTACAAACATGTGAGTGTAAAATAT
TGAGTCCAACATCAGAAAGGTCAAAGGTGTCACAAGTATGAATGATTAATATAGGATAGTTGAGCCAAACCCATA
GGTTTAAGCTTCTAAGTTAATTGGTGTCCTACCATGCTATATTATGGTCTTTTTAGAAGACTTTCCAAGCTATCA
ATCTCCCTTCCATATGGTTTCTGATAAATATGAGGAAAATAATTTGCATCAAAGTTGTAAAGTTTATGACTAGGT
TGACCCGCGATGCTATGATTTGACCAAAAGTGATTCCCTGTTTTGCACGCTGATAGGAACTTGTGGATCTGGGTC
TTACACTGTTGATCCACGAAGCTGATTTCTGCGAGTGCATTTTTGTTTTTATTATTTTACCTATATATATATATA
AAAAAAAAGAAATTAAAAAAAAAATCTATTACTAGAATTTCTGAGATTTTCCATTGGGACTGTTTTTAACATTTG
GTTGGACTATGAGAAAATTGGAGGAATAAGGAATCAAGTACATATTAATTCTTACATTTCCTCACTTAATAAGCT
GTTCACCTTTGTCTGATTTTTAATCAAGTGCCTTGTGTTCATGGTTTGTCCAGGTGGACACATCAACCCAGCTGT
GACATTTGGTCTGTTTCTTGCAAGGAAGCTTTCCTTGACAAGAGCACTTTTCTACATTGTAATGCAATGCCTTGG
CGCAATCTGTGGTGCCGGTGTGGTAAAGGGATTCCAAAAAACACAGTATGAAAGGGTGGGTGGTGGAGCCAATGT
AGTGAACCATGGCTACAGCAAGGGGGATGGCCTTGGCGCTGAGATTGTTGGCACTTTCGTTCTTGTCTACACAGT
TTTCTCTGCAACTGATGCTAAGAGAAATGCACGAGACTCCCATGTACCTGTAAGTATTACCCAGTTTCTTTGTTC
TTTTTCTATGCATACCTGTAAGTATTTGTCCCCTCATATTGTTGTTTGGATCTAGTTTGTTACATCAAATCAAGA
CTTTGAATGACAAATAAGTACAGAAGAGGAAAAAGAAATAAATCAATTACACACAAGAGACACAATACTAATGTG
GCTCGACATAAAGGCCTACATGCAGGCAGATACAATCACAGAGAAATTTGACTGACAAGTGATGGATTACTAAGG
TTGCGAGAAACACTCAGATCCCGAGCCTACTAACCTTAAAGCTCAATATACTAAAAGCTTTACTCTCACAACATG
GTAAGAGAACCTTTTGATTGGACTCCTCTCTTTGCCATGATATAGAACGCTCTGAGAAAAACACTATACACCCCT
ACATACATATAAAGACTAAGTTGGCTAGAGCAAGTTGGTTAAAGCCAAGTCAGAATGCATGGCCACTAAGTTGGG
AAGGTTTTGCATTTAACTGACTCAACAATAAAGATATGCACAGTAAAAAAAAAAAAGAGCATTCAAATTTCAACA
GTATATAACAGTGACTCCTACTGTAAGTCTAGATTAAAAACCAAACCTCTCAACTAATGGCCTCTTTTCATTCCC
TTTGTCGCTTAATAATTTGTGGTGATGTGGCTTGAATGGTATGGGAATGTATGGTTTGGGTTGCCAAACTATTAT
ATACTTATAAAAGGCTATTACCTGCGTACAAGTTCTCCTTGAAGTAGATGATGCCTTTTCTTGTCCTTGTAGGCA
TGCCAAGCTTTGGGTGGTCACTCTTGTTACTCCACTCTACCCTTTTGTGATATTTGGGCCTATTGGAAAAGTCTT
ACGTGATAAGTGCAAGGACAAGCTTTTGCTGAAGTTAGCAACAATACTAATGTGTGAATGCTTTTTGTTGGGTTG
TGTTGTTTCCACAACTCTTGTATACATGTCTTATGCACAAAGACATGCATCAGCCACTAGTTGCATGATTTGCCC
AACAAACACATGGTTGGTTAACTTGGTTTGCTTGTGCAGAATATATACATAATATAACAATATTTTGTGCAAGTA
GCATTTTTCAATACAAAAAGTGCATTCACTTAATAATTTTTGTGGAACTCACTAATGATTATGACATTGTGGTGC
ACTGCTCAGTTTAATGTAGAGGTCCATCCACATAGTTTGAATTGATGTTTGTGTTTAAATTTTGCATGTGGGGTT
CACATGTATGCCTGTTGAGTGCGTGCCTTGTTTGATGGTTTTAAGAACCTTTTGAAAAGATACACCGCTGGTTAG
CCAGTACTTAGGGACTGTACTCTGCATGTGAACTTTGACTAGGTTCAATGCCTTAGACTAGTCATAGTTTGAGTT
TCTTTTAATGTATTAATATATGAGCCAAATCAATTTATTGGGACCACATGTCTTTATTAATACTCTGTTAATGGA
CTTAACAAAGTCTAGGTTTTGTTTCTGGATAGATTCCCAATCTAATGGGATCGTTTAAGGAGGAGGGCAACTAGC
TTATGATGGGCTAGGTGTGAGTGGGATACATGCGTGTTTGTATGGACACACACAAAATTATTTCAACTCAAAATT
ATTTCAACTAAGGCATTGTCATGATCATAGGTATAAAGTATAAACCTGTCTGAAAAGATGGTGTTAGGGGTCAGC
AATTTGGGGTTGAAATCGAAATCAATTGCTGTCAGGTGTAGTGTAGCTTGAAATTGAAATAATGGTGAGTACTTT
GAATGTTTTAAGCCAAACATTCTACATGTTTGCATTTTTTCAGTCAAACATTCTGCATGTTTGAAGCTTGTATTC
TTTTTAGAAAGAGCACTTAATTTCTACTCCTTGGAAAAGGACCAAGCTAATCTATGAGCTTGTCTAGTCTCTAGT
CAGAGAGATGTTGCTCTTGTAAAGTAGCTTGAAGCAAGACTTAATGCAATGTGTAAAATTCAAGCATAAGTTTGG
ACTCGGCATGAATTAATACCAGAACCTCCACTTGGAACCTGACTTTGGGATAAAAAAAATTGACTTATAAGCTAG
TCCTACTTCGTACTTGGTTTTGATTTGTGTTTACTCCTTGTTAGAATATTAATTAAATATTACATTTATTATTTT
CTATTAACTTTAAGCTTTCGAAATAAGTAGTGATTCAACATAGGATCAGAGTAAAGATTTTAAGTTTGAACTTTG
TCTTTATTAATTTACCTTCTATTTCAATTAAATATTTCACGTGTGGTCTCAATTATTAAATAAAAGTTTGAATTT
ACACGTGAGGAGTAGTGTTAGAATACTAATTAACTAATTAAACTTATCATTAATTTGCTGCTAGCTACTATTACT
TTCTTGCCTTACAAATATGCTGGTCATAAACGTCTCCTTGTTTGAGTTCAACTGCAGCTCTTGGCTCCACTTCCA
ATTGGGTTTGCAGTGTTTTTGGTTCACCTGGCAACCATCCCCATCACAGGAACTGGCATCAACCCTGCTAGGAGC

CTTGGGGCTGCCATCATCTACAACAAGGACCACGCATGGGACGACCAGGTGAGTAAAAAGTGTTTTATGATTGCA
TACATTCACTTCTGGATGCGTGGACATGTGATTTATAACTGAAGCGTATGATGTGTTTGTTGCGTGCTTCAGTGG
ATCTTCTGGGTGGGGCCGTTCATTGGAGCTGCACTTGCTGCCCTTTACCACCAGATAGTTATCAGAGCCATGCCA
TTCAAAACCAGAGCC**TGA**GAACTCGTGTATTTCGTCTTCAATCTTGTGTTTTGCTCTGTTTGGATCATGTTTGAC
CCGTCCTTCTCTTTATTAATGTTGAGAGTGGAATTGATGTGATGTTTATGTGCATAAGTAATCTGGCTTTGTGAC
AGTGGACAACTTTTTTTTTTCTNCAGTAGACAACTCTTAACCTGTAAGTCTTGTTTAGTACGCAAAATGATTATT
TTATTAAGAAAATTAATAGTTATTATTTGAAATGAAAGAATGCGGAATAAATAACTATTTATATTCTTTAGTTTG
ATAATAACACATATGTTTTCATTGAAATTATATCAAAATTATTAAAATATCTAAAAAAAAACACTAATTAAAAAA
AAAATCCCTAGAAAAAGTTTTTGGTGGCTGACCACCCCCATCATGGAAGAACCACAGGAGTGGTTGTACAACCCC
TGATTTTTTCGGTTGCCCCGGTTGTTTTTCAGTGTATGGGTATTTTGATTATGTTGAAATTTTTTGGTAAGCGGA
TGGGCGAATGAAAGGTTGATTTTAGGGCCTTGGATTGTTGTTCTTGCTAATTGTGGATGGTTGAACTGGGAGGTA
ATGATTTTGGTGGAAGTCGATTTTCGATTGAAGAAGGTTTGATTCAGTTGGCTTAACATATTGGGTGTTATGGAA
TCTCGATGGCTGATGAATTATGAGTTGTGAATTGGCCGAAGGGCCGATTATGCAAAGGTTCTGTAGGATAGTTTA
CTGGGTTTCATATTGTGGAAACTTGGGTTGTGATGGTTTTAGAAATTGATTTGGCCGAAAATTTGAGTAGTAATT
GGTGTGGGTTTTTGCGGTAATTTTGGTAGCTAACCGATTGGAGAGAAGGGGCAGATTGGTAGCTCTTTTGTTTTG
TTTGGGTTGTGCTGTGGACGATTTCGTAGACTGAAAGGAAAGAAATTTTTGGAGTTGATGGGAATTTTGGATGTT
CTTGAAGGTCTGTTATGGTGTTGTTAATTAACGTGCCCTTGGGCATATGGAGGGAGCATATACACTACCACTGAA
GTTTTCTCTATCTTTACAGCCATGCTTAGTAAGGCTGTC

>*Bpe*PIP1;4

ATGGAGGGGAAAGAAGAGGATGTGAAAGTAGGAGCGAGCAAGTTCCCAGAGAGGCAGGCCATAGGGACGTCGGCA
CAGACGGACAAGGACTACAAGGAGCCACCACCGGCTCCACTGTTTGAGCCCGGTGAGCTTCAGTCCTGGTCTTTC
TGGAGGGCTGGGATAGCTGAGTTCATTGCCACCTTCCTCTTCCTCTACATCACTCTCCTCACTGTCATGGGTGTT
TCCAGGGCTCCCAGCAAGTGTGCCTCTGTTGGTGTTCAGGGCATTGCTTGGGCCTTTGGGGGTATGATCTTTGCC
CTTGTTTACTGCACTGCTGGTATCTCAGGTGGACACATCAACCCAGCTGTGACATTTGGTCTGTTTCTTGCAAGG
AAGCTTTCCTTGACAAGAGCACTTTTCTACATTGTAATGCAATGCCTTGGCGCAATCTGTGGTGCCGGTGTGGTA
AAGGGATTCCAAAAAACACAGTATGAAAGGGTGGGTGGTGGAGCCAATGTAGTGAACCATGGCTACAGCAAGGGG
GATGGCCTTGGCGCTGAGATTGTTGGCACTTTCGTTCTTGTCTACACAGTTTTCTCTGCAACTGATGCTAAGAGA
AATGCACGAGACTCCCATGTACCTCTCTTGGCTCCACTTCCAATTGGGTTTGCAGTGTTTTTGGTTCACCTGGCA
ACCATCCCCATCACAGGAACTGGCATCAACCCTGCTAGGAGCCTTGGGGCTGCCATCATCTACAACAAGGACCAC
GCATGGGACGACCAGTGGATCTTCTGGGTGGGGCCGTTCATTGGAGCTGCACTTGCTGCCCTTTACCACCAGATA
GTTATCAGAGCCATGCCATTCAAAACCAGAGCC**TGA**

>*Bpe*PIP1;4

MEGKEEDVKVGASKFPERQAIGTSAQTDKDYKEPPPAPLFEPGELQSWSFWRAGIAEFIATFLFLYITLLTVMGV
SRAPSKCASVGVQGIAWAFGGMIFALVYCTAGISGGHINPAVTFGLFLARKLSLTRALFYIVMQCLGAICGAGVV
KGFQKTQYERVGGGANVVNHGYSKGDGLGAEIVGTFVLVYTVFSATDAKRNARDSHVPLLAPLPIGFAVFLVHLA
TIPITGTGINPARSLGAAIIYNKDHAWDDQWIFWVGPFIGAALAALYHQIVIRAMPFKTRA*

***Bpe*PIP1a-like**

>**FXXK01000380.1** :39453-39706 *Betula pendula* genome assembly, contig: Contig379, whole genome shotgun sequence

>**Bpev01.c0379.g0007.m0001**

Chr Bpe_Chr14 13,436,305-13,436,932 (1) ::
join(13436305..13436468,13436863..13436932)

>*Bpe*PIP1a-like

AAACAAAACAAAAACAAAAACTGGACTGGCTGTGCTAGATTCATAATTTAATGATGAACTAGCATATCACATTGC
AGAGAGATTCAGAATTTAGCTTCACCCTTATCCCCACTTCACAGCCTCTGTTTTTGAGCGTGCTGAGAGAAAAAT
AGCATCTTTTTGAGAGAGAGAGAGAGAGAGAGAAAGAAAGCTCAGCATTGGTAGTGAACAGTAAAACC**ATG**GAGGGC
AAGGAAGAGGATGTTAAGCTTGGAGCAAACAAGTACTCAGAGAGGCAGCCGTTGGGCACAGCAGCTCAGACAGAC
AAGGACTACAAGGAGCGACCTCCAGCTCCTCTGTTTGAGCCAGGGGAACTCTCCTCATGGGTGTTAAGCGCTCTG
GCAGTGTATCCATATGTTCACCACTCAAAGAAAAAAGAAAAGAAATGGTTACGGACTAATGAAATGTGTGATGGA
GTGGAAAAAAATCTTTAGGATGCTTTCATGGATGAGATTTAGGACATAAAAACATTGTCACCACAGAAGAGATCTC
TTGGGTGATCATGTCT//

>*Bpe*PIP1a-like

MEGKEEDVKLGANKYSERQPLGTAAQTDKDYKERPPAPLFEPGELSSW

***Bpe*PIP1b-like**

>**FXXK01000008.1**|:31941-32239 *Betula pendula* genome assembly, contig:
Contig7, whole genome shotgun sequence
>**Bpev01.c0007.g0002.m0001**
Chr5 15,083,781-15,094,079 (-1) ::
complement(join(15083781..15083934,15086266..15086354,15093924..15094079))
>*Bpe*PIP1b-like
ATGCACCGCACTACTACAGATTTCCCCATCTATACGCGCGACCATAATAGCCTTGAGGCTACACCTGCTTTTCCC
CACTCTGAGAACCACCACCATGATTCGCCACCACCTCCTCCATCAGCAGCAACAAAGAACCATCGGTGCGATAGG
AGTCCATGCGGCGTAGCGCAGCACAACACAGCTAGTTCGTGGAGCTGGTGCGGTGCGGCGAAGCTAGTCCGTGGA
GGTGGAGCTGGTGCGGAGAGAAGAAGCAAAGAAAGA**ATG**GAGGGGAAAGAAGAGGATGTGAAAGTCGAAACGACC
AAGTTCCCAGATAGGCAGCCCATAAGGACCTCGGCGCAGACGGACAGAGACTACAAGGAGCCACCACCAGCTCCA
TTGTTTGAGCCCAGTGAGCTC**TAG** //
>*Bpe*PIP1b-like
MEGKEEDVKVETTKFPDRQPIRTSAQTDRDYKEPPPAPLFEPSEL*SWCFWRARVDEFIRDLLERERNCVLELGV

PIP2 – Plasmalemic Intrinsic Protein type 2

*Bpe*PIP2;1

>**FXXK01000659.1**:65175-71398 *Betula pendula* genome assembly, contig:
Contig658, whole genome shotgun sequence
>**Bpev01.c0658.g0014.m0001**
*Bpe*_Chr3,complement(join(19090629..19090748,19090840..19090980,19091085..19
091380,19091486..19091774))) Genomic Location: 19090629-19091774

>*Bpe*PIP2;1
GAATTTCAACCTTGCATAAGCTTAGATAAATTTCTAGAATTATAGTCGTAATAGCAAACGAGAGCGAGAGAGTGG
TTTCTGACTTAGTACCTTGTTCTTGATACCATGTAAATTAACTATTTGTCATAAAAATTTAAGTTAATAAAAAAT
AAAATTTATTTTTTCTTGGTCCTCTACAACTAGAGAAAAGTTATCTCAAACTATTAGAGCATTCCCGAGACCAA
AATGAATTACATTCGGCTCAGTAAAATGGTAAAATATATGGTGAGTTGCTACAATATCCACCAGATTTGGTGAGC
TTGGTTCACTCACCAAATGTGAAAAAGAAAAAAAAAATTAAAAAAAATCTACACTATTTTTTCTCATCTCAAAGA
AAGAATAAAAAAATAAATGAAAATGTATATTTAAAGTCAATAGTTAAAATAAAAAATTTAAGAGAAAATCTTTCC
ATAATTTTTCACACGTTTTGAAATACCCTTTTTGATATTTGATTTTTCTCACTTTGACCTTTCATACTTTCAAAA
TATTTCATTTAGCTTCCATCTGTTAAGATTTTCCTTTAAATCCTAACGAAGTAGTGCAAAAATTATCAAAATACC
ATTTATTTTAAATTTTTTTATAATTTTTTTTTTAAAATACACGAATTTTTTTATAATTTTAATAAGGTTTGACGA
TAAAAATATGCAAAAAAGGGCTAAATAATATAAATTAAAAATATAAAAAGTAAAAGTAAAAGAAATCAAATATCA
TAAAAATATTTTAAAATGCATGAAAGTTTGACGGCTTTACTAAAGTTTTCCCAAAACTTAATGTAAATGCAGTAG
TCTCCTTTGCGTTTAGCAATATTCATTGACATTAGCAAAAATCCACATCGGCAGCAGCCCCCCCATCTTGAAATT
AACCCAAAAAGGCCTTTTTCTAGTTTTCTAAGCATGCACTAGTGCCCTTCATTACTCTTAACAAAAACCCAAAAG
GCCTTTTTAGTTGTTTAACATGGTCCTACTGAATTAGGGTAGTCTCAAAAAAGTAAAGAAGGGTTGCCCTAAAAG
AGAAGACAGGGGATGGCAAAAAGACAAGAAATAACCGTTGTAGGCGTGGCGTCAGGCGTGGCTTTGGTTGGATGC
CAGGCTGACTCATGAGCCCTACCCCAGAGAGAGATGATTAGCCCTCACATGGTCTTCGCTGTACACCTCGGTCCC
ACTCCCACGTCCCACCCCTCTCTAAACCCTAAACCTAATCCTCTGAAAAAAACTCTTTCCTATTTCCCAACCATT
TTTATTTTTCTACATATTTTATATTATTATACATAGCTGATAGCACCACTTTATATTCCCAAACTGCCCATTGGC
TTATCCATATAATGCACCATTCCCCCTACCCCTTCTCTACACTCTTTATAACTCTCTCTCTCTCCCTCGCTCTTT
AGCTCACAAAAGTTCCCGAGAAACAAACACAAACCAACAGAGAGAGCTTCACAAGGAGTACTGTGTGAAAGAATC
AGAAAG**ATG**TCGAAGGAAGTGAGCGAAGAAGGGCAGAGCCACCAGCATGGGAAGGACTACGTGGACCCTCCACCG
GCACCACTCCTCGACCTGGCCGAGCTCAAGCTCTGGTCCTTCTACAGATCCCTCATTGCCGAGTTCATCGCCACC
CTCCTCTTCCTCTACGTCACTGTCGCCACTGTCATCGGCCACAAGAAGGCCACCGGGCCCTGCGATGGCGTGGGC
CTTCTGGGTATTGCTTGGGCCTTCGGTGGCATGATCTTCGTCCTTGTCTACTGCACCGCCGGTATCTCTGGTAAA
AACCACCCCATACCCCCACAATGCTTGAAATTTCACAAACTCTCTTGTTTGAAGTAATTTTGTTGATGGGTTTT
TGCTTTTGTGTTGCTATATATGCAGGTGGGCATATCAACCCGGCTGTGACGTTCGGGTTGTTCTTGGCCAGGAAG
GTCTCGCTGATTCGGGCTGTGTCGTACATGGTGGCGCAGTGCTTGGGAGCTATTTGCGGTGTTGGGTTGGTCAAG
GCTTTCATGAAGCACGACTACAACATGCAAGGCGGTGGAGCTAACTCTGTTAGTCCAGGGTACAGTACCGGCACC
GCCTTGGGTGCTGAGATCATCGGTACCTTTGTGCTCGTCTACACCGTTTTCTCAGCCACTGACCCAAAGAGGAGT
GCTCGTGACTCTCACGTGCCTGTATGTAACCGTTTTTCACTTTTTGATTTCTTCATGTTCAAGTAATTCGTTGCC

TATTTTAGGCCATGTGGTAGAATACGGTTTTCATGTTTGTCTGCGTGCAGGTGTTGGCTCCACTCCCTATTGGGT
TCGCCGTTTTCATGGTGCACTTGGCCACCATCCCCATCACTGGAACTGGTATTAACCCAGCTAGGAGCCTTGGTG
CTGCTGTTATCTACAACAACGGCAAAATCTGGGATGACCAAGTAAGTATATATACACTAACAGGCACATCCGGGT
GAATTTGTGAATTTTTTTGGAGTTACTAACAAGTGGGTAAATATATGTAATGTGCAGTGGATCTTCTGGGTTGGT
CCGTTTGTGGGAGCACTGGCTGCAGCGGCGTACCACCAGTACATACTGAGAGCAGCGGCGATCAAGGCTTTGGGA
TCCTTCCGCAGCAACCCAACCAACTAAAGGAGACAAAGACAAAGCAACCAACCGAAACCTGAATGAAAACTTTCT
AAAATGTCTCATAATTTTCTCTTTTCTCCACTACTATTTGTTTGTTTGCGTGTATGAGATCGAGGATGTTGATGA
TGATTATGATGATGACCCTTCTTCTTCTTTTCCTTGTTTTTATTTTTTTTTATTTTTTTTATAATTTAATCTTTTA
TTGTTAGCATTGGATGTGTATCATATATTGTTCATGACTAGTGATGATGGTGGTGCTCCTTTTTTAATCCCTGTT
GTTGGACTTCTCTCTCTCTCTCTAATCTTCTCCCTAATCTTCTTTCGTTATGGTCTGTTTATTCTAATTCTCTGA
CAATAAAAAGGATTTAAAACCAAATTAAGTCTTCATTTTGTTGAAACGACAAAACGGTGCGCTTATAGGGGTTTT
AACGTCAGATATAATAGGATCGAGTGGGTTTAGCCCTTGGATTCTCCCTAAAGACAAGCCACGTGTCCTTCCAG
GAGCACACCTGGGAAGGGCCAAAGATGCCCTTAAAGCTTTTAATGGGTCTGATACCCAGCTTTCTCTCTCACTCT
CCCTCCGAAAGGTCACTTGACATATTCTGGCAAGCCAAAGTGTTTACTGTAGTGTCCGCTTTTAGGACAAAGGTC
CAACCAATGGGACGAAGCGAAGAAAAAAGCAGAAAATGAAAGAGGATGGGATTCGAGGATGTGCCACGCTGTGAG
GGGCCACGTGTCATGGGACCATAGGATAGCATGTCATGGCATCACATGGGTGGGTAAAGTGGG

>*Bpe*PIP2;1

ATGTCGAAGGAAGTGAGCGAAGAAGGGCAGAGCCACCAGCATGGGAAGGACTACGTGGACCCTCCACCGGCACCA
CTCCTCGACCTGGCCGAGCTCAAGCTCTGGTCCTTCTACAGATCCCTCATTGCCGAGTTCATCGCCACCCTCCTC
TTCCTCTACGTCACTGTCGCCACTGTCATCGGCCACAAGAAGGCCACCGGGCCCTGCGATGGCGTGGGCCTTCTG
GGTATTGCTTGGGCCTTCGGTGGCATGATCTTCGTCCTTGTCTACTGCACCGCCGGTATCTCTGGTGGGCATATC
AACCCGGCTGTGACGTTCGGGTTGTTCTTGGCCAGGAAGGTCTCGCTGATTCGGGCTGTGTCGTACATGGTGGCG
CAGTGCTTGGGAGCTATTTGCGGTGTTGGGTTGGTCAAGGCTTTCATGAAGCACGACTACAACATGCAAGGCGGT
GGAGCTAACTCTGTTAGTCCAGGGTACAGTACCGGCACCGCCTTGGGTGCTGAGATCATCGGTACCTTTGTGCTC
GTCTACACCGTTTTCTCAGCCACTGACCCAAAGAGGAGTGCTCGTGACTCTCACGTGCCTGTGTTGGCTCCACTC
CCTATTGGGTTCGCCGTTTTCATGGTGCACTTGGCCACCATCCCCATCACTGGAACTGGTATTAACCCAGCTAGG
AGCCTTGGTGCTGCTGTTATCTACAACAACGGCAAAATCTGGGATGACCAATGGATCTTCTGGGTTGGTCCGTTT
GTGGGAGCACTGGCTGCAGCGGCGTACCACCAGTACATACTGAGAGCAGCGGCGATCAAGGCTTTGGGATCCTTC
CGCAGCAACCCAACCAAC**TAA**

>*Bpe*PIP2;1

MSKEVSEEGQSHQHGKDYVDPPPAPLLDLAELKLWSFYRSLIAEFIATLLFLYVTVATVIGHKKATGPCDGVGLL
GIAWAFGGMIFVLVYCTAGISGGHINPAVTFGLFLARKVSLIRAVSYMVAQCLGAICGVGLVKAFMKHDYNMQGG
GANSVSPGYSTGTALGAEIIGTFVLVYTVFSATDPKRSARDSHVPVLAPLPIGFAVFMVHLATIPITGTGINPAR
SLGAAVIYNNGKIWDDQWIFWVGPFVGALAAAAYHQYILRAAAIKALGSFRSNPTN*

***Bpe*PIP2;2**

>**FXXK01000578.1a**|:150836-155543 *Betula pendula* genome assembly, contig: Contig577, whole genome shotgun sequence

>**Bpev01.c0577.g0022.m0001**

Chr *Bpe*_Chr9 1,816,676-1,817,768 (-1) ::
complement(join(1816676..1816786,1816869..1817009,1817105..1817400,1817480..1817768))

>*Bpe*PIP2;2

AGCCATGGTCGTCCAGCCAATACACTGGATTTATCCCAGAAATGCTGGAATTCAATCACGAACAGATTTTCTCCA
ATAACATTAAACGTACAACCTCCCGAAAGCTTCCACCCCTTCGCAAGAGTAGATTTTATAATCTCCTTGCTTACT
ACTCGGTCAGCCATAAGTTTTCCAACCACACATTGTTGTCCTCGAGTGACGTTATCTACAAGCTCCTCCACCTGA
ATCTCCAGGCCCACATTCTCCTCCTCCGTCAGAGAGAATTTTCCCAAGAGTTTGGACAAGTCGTTCGCCACAGAA
CCCATAGCAGTCGTCTCTGTCAATGATAGTCAACCCCTTCCAGAGATGTCCACCCGTATAAACTAAAGACAAAAT
CTGAATTCTACTTCTCAGTCCTAAAAGGACGAGAAAAAACTTTTCTTCTCCTCTAGAACCCTCCAGAGAGAAAAC
TCATGCACGAAGCCTTAAATTTATTACAAGTTGGTATAACCACATGAACTTATTATATATTTTTCCATTTGAAAA
TTTATAGGGAGGGCGGGCTCCATGACTAAGCCAAATAGGCCACGTCCTCAATTTATAAAGGCCCCTAAAAAGGC
CTTTTATTTATATAAAATTTGTCAAAAGAAAAAGTGAAAAAGGGTAAAAGGCCTTAAAAAATAAATAAAATAAGA
AGAGGTCATTTGTTATGCGCGCTATGAGGGTTTTTTTATAAAAAAAATCTAACTAAATTAAGCACTATGCACTC
TGTTTTTATATTTATTTTTATCTTATTTTCTTTTATCGCTTCTAATCGTTTGTTTATTGGTTATATCGTTCTTTA
TTTTTTTTTGATATTTTATATGTTATACCTTCAATCAGATGTCATTGATTTTTGTTTTCGGTGTTATGAGTTTCA
TTCACGTAATTGATTTTAGTAAATTTTACATCTCAAAAAGTTAGAACGATGATTTTTTAAATTAATTTTTAGATA
TTTAGTCTAAAAATTTCTTATATTAATATTTTATAATTAAAAATAACCATTTTTTAATTTTTGCCAATCCACAAC
TTTTATTAAGCCTTTCCTCTTTCTCTTTCTCTCTTACGCACATGACTAGATTTATAATTAATTTACAACTTGATT
TATTGGAGCTATATCAATCACTAAAAATGATTATCACTCTCAAATCACATAATGACATGTTTCAACAAATTTTTT
AAAAAATGTAAATCAGTTGTAAGTCTAACATATATTATTGTCGGAGAACAAATACAAGACCATGTTGTGTAAATC

ACGAAGTTCTTCATGTTCGCATTGAAAAAAAAAAAAGAAAGAAGAAAAAGTGAAAGTGATATTTGGTGATCGAACA
ACACTAACAGACAATGTGGTTGTTCCATCAGCATAATCTCAATCAGTGTAGCGCCTGTACGTATAAGGACGTACA
CAGTTTTAAGCACTAAAATTGCACATTACGTCTCTGACATTCTTGAAGATTTTTACTGCTTTCCCGATTAAACAG
ATGGATTAACAAAATCGTGGGCTGAGCATTCACACTGGGTAATCAAAATAGTCAAAAAAGTTATTTTGATTAATT
GAAAATAGTAAAACAACTTTTTCATTCGTCTAATCACTTCAGCTAAGAAATTGGTTAATTGATACAGTATTCAAC
AAATTTTTTAGTACGTATAAAAAAAATTAATAGAAAAAAACAATTGATTTTCATTTCTTTTGGGTATAGTTAAAA
CAGATTAAAAAAATATTTAAAATTTAATAATAAAAATAGATAGTTACAATATTGTGCAGCATGTAGGTGCACAAA
AAAAGTTCATAACTTTTTTTTTAAAAAAAAAAGTATTAATTAAATTTTAACCAAATATTCGTGTGGCGGCCGGGA
TGTGAATGTTACGTACATACCATCATTATCTCTTGATTTTGCGAGTAAATTCTCATCAAATAAAAATACAAAAGA
TGCTAATTTTGTCTTCGTTTTGAAAAATTAGACTTAGGTTACGAGTGACACTTCAGCTTGCCAATAACCTCTAGC
CAGAACCGCCCGGACTAGAAAAGATGCTCTGCCTGCTGCATCGATCCCATTTGTTCCACTCAAATTCTGTCAATC
AAGTTCTCATTTGTCTTCAGTAGCGAAGGACAAAGAGAGAACGTACAATTTATAAAGAGCTAATCTATTGCATGC
TCTAAACTTGAGTCTTTTAAAAATTTTATGAAG**ATG**TCGAAGGAAGTGACTGAAGAAAGAGAAGCTTCTCAGCCG
GTGAAAGACTACGTTGATCCACCAGCAACACCACTTTTTGACGTAGCAGAGTTTACTATGTGGTCCTTTTACAGA
GCAGTTATCGCCGAGTTCATTGCGGCCCTTCTCTTTCTCTACATAACTGTCGCCACCGTTATCGGTTACAAGAAG
CAAACCGATCCATGCGAGACAGTTGGCCTTCTGGGCGTCGCTTGGGCCTTTGGGGGCATGATTTTCGTTCTTGTT
TATAGCACTGCAGGAATATCAGGTTTATTTCTCTATTTATTTTATTTAATAGATTTTTCACGACATATATATGCT
AATTAATTAGTCATATATATGTACAGGTGGTCATATTAATCCAGCCGTGACGTTCGGTTTATTCCTGGCACGAAA
GGTTTCGTTGATAAGGGCGATTTGTTACATGGTGGCTCAGTGCTTGGGAGCAATATGTGGAGCAGGGTTGGTGAA
GGGGATCATGAAGGACTCCTACGATGCTTTTGGAGGTGGCACGAACACCGTGTCTCCAACCTACTCCAAGGGAAC
CGCTTTAGGAGCTGAGATTATTGGGACTTTCGTACTTGTCTATACAGTCTTCACTGCCACAGATCCTAAGCGAAA
CGCACGCGACTCCTACGTCCCAGTAATTAAGTAGCTTTAGTTTCTAAGTCGTTTGAAATTTATAACGCCAACCTC
GTACTAATAACTAACATATATATTTGTGTGTTAAAATGATAGGTTTTGGCTCCCTTGCCAATTGGGTTTGCGGTA
TTTATGGTGCACTTGGCCACCATCCCCATCACAGGCACAGGAATAAACCCAGCTCGTAGTTTTGGGGTTGCTGTA
ATCTACAACAACGACAAAGTTTGGGACGATCAGGTCAGCTTAATTTTAGCACCATCGATTAATTGATCAGACTTT
TGATTTGTTGTTGACTGAACGTGGTGATGGTGATAAATAGTGGATTTTCTGGGCGGGACCTTTTGCGGGAGCGCT
CGCTGCAGCACTATATTATCAGTTTATTCTGAGAGCCACCGCCATGAAAGCTTTGGGATCGTTCACCACCAAC**TA**
GGCCAATATTTCTCCTCACTTCTCAGTTTGTTCCTCTGTACTTTTTTTTTTTGGTTCCTCTGTACTTATCTATGT
GTACAGGTGTATGTGATTTTTTTTTTTTTGGTTCTTTCTTGTGTGTATAGTTATCCTCGTACTAATCTATAAAAT
TAAATAAAAAAAAAGTCATCCTCGTACTACTATTGATGATTGATGGTTACGATCTTGGTTGTGGGGTTACTACTA
TCTCGATCTCGTGTTGTGTTCTGTTCTGGGTGTTCTCAAAATATTGGATGGTGCGTCTAATATATATATATATAT
AATATATATATATATATATAGACACACGCGACGTTAACATAGAACTGATTAGATCGATGTGGAAAATGAAAGTAG
TGCAGCATAAGTTAAATGTTTGACAAGGAAGATAATTATATATATATATATTTTGGACGTTGGTTGGATTGCGGG
TAAGCTTTAAAATAAGAAGAAAATTATCGGAGTGGCTTAGGCCGATGGCTAGTGGAAGAGCCTCAGCATTTTTGT
CTCAAACGAGGAAATTCTTTTGAGAGAAGAACATACCTGAGTTATGGGGACAAGGGATCTTTTCTTTGAGGCTTC
TTTGGAGCTCTTTTGAACTTTTATGAGAGTGGGCCTTTACTTCGGCCGGGTATTGGCCCTTGGGCTCAGACTTAT

>*Bpe*PIP2;2

ATGTCGAAGGAAGTGACTGAAGAAAGAGAAGCTTCTCAGCCGGTGAAAGACTACGTTGATCCACCAGCAACACCA
CTTTTTGACGTAGCAGAGTTTACTATGTGGTCCTTTTACAGAGCAGTTATCGCCGAGTTCATTGCGGCCCTTCTC
TTTCTCTACATAACTGTCGCCACCGTTATCGGTTACAAGAAGCAAACCGATCCATGCGAGACAGTTGGCCTTCTG
GGCGTCGCTTGGGCCTTTGGGGGCATGATTTTCGTTCTTGTTTATAGCACTGCAGGAATATCAGGTGGTCATATT
AATCCAGCCGTGACGTTCGGTTTATTCCTGGCACGAAAGGTTTCGTTGATAAGGGCGATTTGTTACATGGTGGCT
CAGTGCTTGGGAGCAATATGTGGAGCAGGGTTGGTGAAGGGGATCATGAAGGACTCCTACGATGCTTTTGGAGGT
GGCACGAACACCGTGTCTCCAACCTACTCCAAGGGAACCGCTTTAGGAGCTGAGATTATTGGGACTTTCGTACTT
GTCTATACAGTCTTCACTGCCACAGATCCTAAGCGAAACGCACGCGACTCCTACGTCCCAGTTTTGGCTCCCTTG
CCAATTGGGTTTGCGGTATTTATGGTGCACTTGGCCACCATCCCCATCACAGGCACAGGAATAAACCCAGCTCGT
AGTTTTGGGGTTGCTGTAATCTACAACAACGACAAAGTTTGGGACGATCAGTGGATTTTCTGGGCGGGACCTTTT
GCGGGAGCGCTCGCTGCAGCACTATATTATCAGTTTATTCTGAGAGCCACCGCCATGAAAGCTTTGGGATCGTTC
ACCACCAAC**TAG**

>*Bpe*PIP2;2

MSKEVTEEREASQPVKDYVDPPATPLFDVAEFTMWSFYRAVIAEFIAALLFLYITVATVIGYKKQTDPCETVGLL
GVAWAFGGMIFVLVYSTAGISGGHINPAVTFGLFLARKVSLIRAICYMVAQCLGAICGAGLVKGIMKDSYDAFGG
GTNTVSPTYSKGTALGAEIIGTFVLVYTVFTATDPKRNARDSYVPVLAPLPIGFAVFMVHLATIPITGTGINPAR
SFGVAVIYNNDKVWDDQWIFWAGPFAGALAAALYYQFILRATAMKALGSFTTN*

***Bpe*PIP2;3**

>**FXXK01000578.1B:**153006-168500 *Betula pendula* genome assembly, contig: Contig577, whole genome shotgun sequence
>**Bpev01.c0577.g0023.m0001** (next to Bpev01.c0577.g0022.m0001)
Chr *Bpe*_Chr9 1,830,657-1,832,603 (-1) ::
complement(join(1830657..1830767,1830849..1830989,1831922..1832217,1832315..1832603))

>*Bpe*PIP2;3
TAATGTCCGATCTAGTGGTCATGATCACCCCGTGTGGTGGTTGCAAGTGAGTGTACATCAATCTTCTATATCGGC
GCACTTTTAGAAGTAAAAATAGTGTTTTACATATTTTAATTGGTATTCTATTTAATATTTTTATAATGTTAGCAA
AAAATTATTTGGAATTTATTTAATAGAGTACTTTAGTAGCTTGAAGGTATTCAGTACGTAATATTTTTCGATTTA
ATGGAGATTAGTATGTAATATAAAACAAAAAAAGAATATGATGTAATTAATTTCATAAAACCTCTAGCGAGGTGT
AATTTTCCCTTTTAGTATTGGTATGTTTATCATAATTAACCAGTAGACTTTGAAAGTGGCCGCACTGCCGGCCAC
TATATTCTACATCTCTATCATCTGGCAATACATCAATATCAATGATTAAAGTTAAAGCCATTACACAATTTTTAG
AATTAATATTAATGAAGAGCTAATTGTGGAGAAGGGAATATGCCGATAGAAGTAGGAATATCTTTTGCTTGCCAT
GCACAAAAAGTATTATAATTAATACAAGTGTATGAGATTAGGTTAATTAGTAATCTAGCTTATTGAATAGATACT
TCTTCTTAATTTGTTCCACTCACTATAATTTTCTTAGTAAGTATAACATTATAATATTGTACGAACACTACAAGA
CCATTTCTTATAAATCACGAAGTTCTTCACGTTCCAAATTAAAAAAATAAAAAAAAATAAAAGAAAAAAGAAAAA
AGAAAGAAAGATGTGAAAGTTAAATCATCCATATGCATGAAATATATGACTTAATTATTTAATTAATATTTTAAC
ACATTTTTTATTGAGGGTTCAAACTCTATTTTAATAAGTGAGACTTAAGGTATAAAATATTTAACTAATCAAAAT
AAGAGATGAGTGACAAAGATAATTAAGTTTAAACTCAAGATCTTGCTCTCATATCACAACTTATTCAAAAAAAAC
TTAAATTGATAAAAATTCTAACATATAACAACTCTCAAAAGAGAATGTGATCGATTAATTGGTCTATCAGCAGAA
TCTCAATCATTGTAGTAGTACTGTCCAGCCTGTCCTGTATGAGGATAGAGTTTTAAGCAATAAAATTGAATATTA
TTTCCTCGACATTCTTGAATATTTTTACCTACCTGCTTTCACGATTAAAGAGATGAATCAACCAAATGGTGGGTT
AACCATCACTATCGTTTGAATTTACGAGTAAATTCTTGTCAAATAAAGGTAACAAAGATGCTTTCGTCTTCATTT
GAAAATTGAACCTAGCTGAGGGTACTATACGAGAGTGACACTTCAGCTTGCCAATAATCTCTAGCAGAACCGGGA
CTAGAAAAGATTCCCCTGCCAGCATCACATTTGTTCCACTAATATTCTATAGCATATGCTCTCTCTCTATTTACA
ATTTTCTCGCACATAAATTCAATCTTCCACTCACACCCTCATCAACTTCTCAGTGTCTTAAGAACAAATTTATAA
AG**ATG**TCGAAGGAAGTGACCGAAGAAGCAGAAGCTTCTCAGCCGGTGAGAGACTACGTTGATCCACCACCAGCAC
CACTTTTTGACGTAGAAGAGCTTACTGAGTGGTCCTTTTACAGAGCAGTTATCGCCGAGTTCATTTCGACCCTTC
TCTTTCTCTACATCACCGTCGCCACCGTTATCGGTTACAAGAAGCAAAACGATCAATGCGAGACAGTTGGTCTTC
TGGGCGTCGCTTGGGCCTTTGGGGGCATGATTTTCATCCTTGTTTATAGCACAGCAGGAATATCAGGTTTATTTA
TCTATTTATTTTATTTAATATATTTTTCACGACATATATACATGAGTAATGCCTAGTCATATATTTATTCTTTTG
GATATAAGTACAGGTGGTCATATTAATCCAGCTGTGACTTTCGGTTTATTCCTAGCACGAAAGGTTTCGTTGATA
AGAGCGATTTGTTACATGGTGGCTCAGTGCTTGGGAGCAATATGTGGAGCAGGGCTGGTGAAGGGCATCATGAAG
GACTACTACGATGCTCTTGGAGGTGGTACGAACACCGTTTCTCCTACCTACAACAAGGGAACTGCTTTAGGCGCT
GAAATTATTGGGACTTTCGTACTTGTCTATACAGTCTTCTCTGCCACGGATCCTAAGCGTAAAGCACGCGATTCC
CACGTCCCAGTAAGTATAGCTTTTTTTTTTTTTTTTTTTTTCTTTTTTTCTTTAAATTTTTAATTTATATTTTTA
ATTATTATTATCATTAGTATTATTATATTAGAGCATTAGCAGCAGCTTCCCTTAAACTCCATTTTCTTCCCTAGT
GTAGGGAAAACTGTTAACAAAAAAATACAAAAAAGTCCCTGCAGCAGCTTCCCTAAGTGTTTCCTAAACTATAGA
AACACCAAGGGAACCACTTTTGGTTCCCTTGATGAAGGGAACCAAAAGTGTTCCCTTAGCATTATTAAAATATAA
AAAACTTTCTCTCTCCTCTACCATTTGTTTAAAACAATATTTAAATGATTTCTCTTTCCTCTCTCTTTCTTCTAG
CATTTAATTTAAATAGTATTTAATTCATGTAGGGAAAGATGAAAGGAAGCTGCTATGTAGTGCATTTAACTAGGG
AACTAAAAAATGGTTTTGTTCCCTACATTAAAGAAAAGCTTAAGGGAAGCTGCTGCTAATGCTCTTATATAGAAG
TGCCAGTAAGTGGCTTTAGTTTCTAAGTCCTAATTCGAATTTTATATGGCCAACCTCGTTAGTTATTAACTAACA
TTCGGAAATTGGTATTGGCGTTGGTAATATGGATATAAAATTTTATATGTTAATCTTAATTAATTTGACCTATAC
AATTAGATAGGTTGGATTCCTCGACTCAAATTACACTAATTTTGTATTAAATTTATAAATTATATAAAAAATTAT
TAGCCCTTAGGTTGTATGTCAATATCAAATTTAGGTTTTATCGAAATATATAATTAAATTAACATCAATATGACC
TATTTAATTAAATGAATAATATCTCTTTTAATTTTATCCTACAAATTTTTTATTGAGTTCGTTTAGTCATACCGG
CCCGTATTAATAACTAACATTTTTGTGTGTGAAATTTGTAGGTTTTAGCTCCCTTGCCAATTGGGTTTGCGGTAT
TTATGGTGCACTTGGCCACCATCCCCATCACAGGCACAGGAATAAACCCAGCTCGGAGTTTTGGGGCTGCTGTAA
TCTACAACAACGACAAAGTTTGGGACGATCAGGTCAGCTTTTAGCTCCATCGATTAATTGATCAGACATTTGATT
TGTTGTTGACTGAACGTGGTGATGGTGATAACAAACAGTGGATTTTCTGGGTGGGACCTTTTGCGGGAGCGCTCG
CTGCAGCACTATATCATCAGCATATTCTGAGAGCCACCGCCATCAAAGCTTTAGGATCGTTCACCACCAAC**TAG**G
CCAAGATTAGTCCTCACTGCTCAGTTTTTCCTCTTGTACATATCTATATATGTGTGTTGTACAGGTTTATGAGA
TATTTTCGTTTGTTCTTTCTCGTGTGTATAGTCATCCTCGTACATGCTAGCTATTGATGATTGGTGGTTACGATG
TTGGTTGTGGGGCTACTATCTCTCGTGCGGTGTCCTCTTCTCAGATATTGAATAATTTGGATTTGTGCGTCTAAT
ATATATCTCTTTGGTGTCGCATCAGAAGCATCGGATGAATGATGAATCTGTATTCTTCATGTTCCACCACACACA
CACACACACACACACACACACATATATATATATATATATACAATTTATTTTGTATTTTTTTCTACTTTGTGTTAA
GTTTATTTATTTATTTTTAATAAGTTATCCGCTAGCTACAAAAGGACTTTTTTCAGTGAGCATGTCACAGTTAAT
TACTCTTATTTTTTTCAACTTTTTTTTTTTTTTAAAAGATAAAGAAACAACTTCTATATTCTCTCGCGTAGATTG

ACATCACACAGCATCAATCATACGTACGCTCGTCCGCAGCATCAATTCATGAAGCTTCTTTATTAATTTTTTCGT
CATTAATTTAGGGCGTTCATAACGCGCACACGTTCTCTTGTGGAGTAACGTACAATACGTCACTCCTCAAGAAGA

>*Bpe*PIP2;3

ATGTCGAAGGAAGTGACCGAAGAAGCAGAAGCTTCTCAGCCGGTGAGAGACTACGTTGATCCACCACCAGCACCA
CTTTTTGACGTAGAAGAGCTTACTGAGTGGTCCTTTTACAGAGCAGTTATCGCCGAGTTCATTTCGACCCTTCTC
TTTCTCTACATCACCGTCGCCACCGTTATCGGTTACAAGAAGCAAAACGATCAATGCGAGACAGTTGGTCTTCTG
GGCGTCGCTTGGGCCTTTGGGGGCATGATTTTCATCCTTGTTTATAGCACAGCAGGAATATCAGGTGGTCATATT
AATCCAGCTGTGACTTTCGGTTTATTCCTAGCACGAAAGGTTTCGTTGATAAGAGCGATTTGTTACATGGTGGCT
CAGTGCTTGGGAGCAATATGTGGAGCAGGGCTGGTGAAGGGCATCATGAAGGACTACTACGATGCTCTTGGAGGT
GGTACGAACACCGTTTCTCCTACCTACAACAAGGGAACTGCTTTAGGCGCTGAAATTATTGGGACTTTCGTACTT
GTCTATACAGTCTTCTCTGCCACGGATCCTAAGCGTAAAGCACGCGATTCCCACGTCCCAGTTTTAGCTCCCTTG
CCAATTGGGTTTGCGGTATTTATGGTGCACTTGGCCACCATCCCCATCACAGGCACAGGAATAAACCCAGCTCGG
AGTTTTGGGGCTGCTGTAATCTACAACAACGACAAAGTTTGGGACGATCAGTGGATTTTCTGGGTGGGACCTTTT
GCGGGAGCGCTCGCTGCAGCACTATATCATCAGCATATTCTGAGAGCCACCGCCATCAAAGCTTTAGGATCGTTC
ACCACCAAC**TAG**

>*Bpe*PIP2;3

MSKEVTEEAEASQPVRDYVDPPPAPLFDVEELTEWSFYRAVIAEFISTLLFLYITVATVIGYKKQNDQCETVGLL
GVAWAFGGMIFILVYSTAGISGGHINPAVTFGLFLARKVSLIRAICYMVAQCLGAICGAGLVKGIMKDYYDALGG
GTNTVSPTYNKGTALGAEIIGTFVLVYTVFSATDPKRKARDSHVPVLAPLPIGFAVFMVHLATIPITGTGINPAR
SFGAAVIYNNDKVWDDQWIFWVGPFAGALAAALYHQHILRATAIKALGSFTTN*

***Bpe*PIP2;4**

>**FXXK01000043.1**:236200-249239 *Betula pendula* genome assembly, contig: Contig42, whole genome shotgun sequence

>**Bpev01.c0042.g0019.m0001**

Bpe_Chr5 4,990,578-4,993,085 (-1) ::
complement(join(4990578..4990697,4990811..4990951,4992381..4992676,4992779.
.4993085))

>*Bpe*PIP2;4

TTATCCCTCCACTGGAAATGTCCGTTACTTTGCACTGGCTACTGTGGGGGCTTGGCTGGGCTGGGCCCCGTTCCC
AAACGTTCCACAGCCAAACCGCCAAGCAATCTGAAGCCACCAGTTTCACCACCACGTGTAGCTGTTTTTTCTCTC
TGAAATCTCAACCACAAAGTGAATCTCACAACCATCCACGTCCACTCTTTCCCTAAATTCCCACACGTTCAAAGA
GCCCCTTGGCATTTTGTGTTTTGGCCTTTTGCGATCATCAGTCATGAATCTTCTTTCTTTTGCTACACGTATTTT
TTTTTTATCTCAGTAGTGGATTTCGACAACTGTTTTGAAACTAAACACTGTTTGGTGCTTGCCATGCCGTACGGC
TACGTTTTGTTGTCCACCGGTTCTAATTTTTCACGTGTCTGTCTTTGGCACGGGTTCAACCCATTGGCTAATGAT
TCTTATATTATTGGTCTCATTAATTGGTAACTAATTATTTTTACTTTTTTCATAGGCTAATAGGTAGTTAAATCT
TTTACATCAAGAATGTCATTGGTCTTCGGTGCATCTTATTGTGTGTATAATAGACAAACAGCTAGGCCGTATAGA
ACCAAAACTGATATTATCGTTGAGAGGGATGAATTGATAGTTTTGGTTCATGTGATTTCATTTTGTAACAAATAA
TTCCACGAGGTTTGAAATGGACTTATAAATTCATGTGGTATGTCTATGTATAAAACAAAATGATCAGTTCATAAA
TTTCAAGCATTTTTTTGATAGTTTTTAGGAAGTTGCACATTTATCCTTAAATAAATAATAACAAAAAAAGAATAA
AGAAAAAAGAAACAAAATTTGGTATGAGATGGCTGAACCATCTTTATAGCCAAACCTGCTAGAGGAGATGATCAA
ATCACCTCCAAAAAAATGAGGTAATTCGACTACCCTCAAAATTGAGCAGTGAGGGTGACTAAACCATTCTTAATG
CTATGAAGATGGTTCAACCACTCTTAATACCAGCCGTCGAGGATGACTGAACTATTACTAAAGTTATTGAAGATA
GTTTAACCATCCCACAAAATCATGTAGAGGGGTTAGCCAAACTGCCCTTAAGAGCGATGAGAATTGTTCAATCAC
CCCTATAATTGGTTTTCAAGAATGATCGAACTACCAACTAAGGTCTTTAGATCAAACCATAACAAGCCGACCACC
TCCCAACCAAGTTTTGTTTTGTTTTTGTTTTTCTTTTTGGGTTTTTATACTTTTTTATTTATGAATAAAATTGTA
ATTTCACGAAGGTGCTGTTGAAGAAGTGGTTGGGAATTTATCCGGATGTATCATTTTGTTGCATAGACGTACATC
CGTGATTTACAAATTTATTTTAAAATACATAGATTTCTTTGTTACAAAGTCAAATACTGCTAGATCAATTTAATA
ATTTTGTTTTAAGATATGATAGTATATATGTTTAGGGATGATATTTTCTTTTATTATTATTATTATTAGATAATC
TCTTGAATCTCATCTAGTATCTGTTTAGGCGTTAATTTGTTAACGATCGAACGAGTAAGATAAGACAGTCTTACT
TGTACAAGAGGTAATCTATAGGATTTTTAATCCACATTCTAAGATTCAAGGCTACGATCATACTCGGTCCGTACG
CATTAGATAATTACAGAACAAGAAAAAAAACAAACAAACAGCCGTATATATAGTAAGTGCGATGCAGAAGACTTG
CCAGTACATGCATGACAGATAGAGAGAGAGAAGAAATGGGGTAAGTGCGGGTTGGCGTGGTAAAGTCAGGCAGGG
ACCGAAAGAGACCAAAAAGCTAGTGCTTAGTGGCTCTGCTCAGGCCTCAAATTGCAGTAATTAATGTTTTTTTTT
TTTTCCTTATATTTTATAAACGGGTAGGAGGGCTACTCTAGTTAGACTTACCATAGTAGCAGTAGCATTTACTCG
CAAGGGTATTATTCAGAAAAAGTCTGGACAGTAAAAACTGCAACACTCTTAATCCTTATTTGCGGGGCATTGCTA
AAAAAAAAAAACCTAAACAATAAAAATCACAACACAAATACTTTTTTTAAAGACCAATTGAACTATACCTTGAAC
CGTCCAACCAAACACACCAAAGGACGATTTTCAAATACTTCCCATTTTTTTTTTTTTTGAACGCCAAATACTTCC
CATTTGAGAGTGAGTCTTGGATCGGGAACCCACATGATACTCGAATGCGACTAATCCCTCGGTGGTAGTAATTAA
TGTCTTAATCCCTACTGTTTATACCAAAACTCCTTGACTAGTCCCTCCTTCACTGATTCCTCTTCCTGTATATAT

ACCCCACAGCGCTCACGTCCTTCCACACCCAAAGCTTCTCTTTTTTGCTTGCTGTTAGTCTTGAGCCTCTGCTTA
ATTTCTTCTCTCTTGCTTGTTTTCTTTTGGGTCTTATA**ATG**GCCAAGGACGCGGAGGTGCCAGAGCATGGCTCTT
TCACGGCGAAGGACTACCACGACCCACCTCCGGCGCCGCTCTTCGACGCCGTGGAGCTCACCAAGTGGTCCTTCT
ACAGGGCTTTGATAGCCGAGTTTATAGCCACTTTGCTCTTTCTATATATCACAGTGCTCACCGTGATAGGGTACA
AGAGCCAAATTGACCCCGCCAATGACGGCGAAGCCTGTGGTGGGGTCGGCGTTCTTGGTATCGCTTGGGCCTTCG
GAGGCATGATCTTTGTTCTTGTTTACTGCACTGCTGGGATTTCAGGTGGATTTTTTTTTTTCCATAATTACTTTT
TCCTATTTCATAAATACATTGTGGGGGCTTTGGTAAATTTATTAATTATTATTTTTATTTGTTTTTACGCAGGGG
GTCACATTAACCCGGCGGTGACGTTCGGGCTGTTTTTGGCTCGAAAGGTGTCGCTGGTGCGGGCGGTGAGCTACA
TGGTGGCTCAGTGCCTGGGAGCCATCAGCGGTGTTGGGCTAGTGAAGGCCTTTAATAAGGCGCACTACACTAGGT
ATGGTGGTGGTGCCAACACGCTGGCTGATGGGTACAGCACAGGCGTTGGATTGGGCGCGGAGATCATCGGGACCT
TCGTGCTTGTCTACACCGTGTTCTCTGCTACGGATCCCAAGAGGAATGCTAGAGATTCCCATGTTCCGGTACGCA
CCTTAACATTCCTCCACTTTTTAGGGTTTTTTATACTTAAGCCATCAATATATCATGCTAATCATGGCATAAACA
CATGGTAACTTTTACCAGATATTTGGTTAATATACGAGGACTAGGATTTCTTCGAATTTTTGAAGGTTAAATCTT
AATTATTGGACCAATGATTGAGATTTTGCCATATCAATAGTTATCATTTTGGAGATAAATATGGTAAGTATTGAG
GTGATAACGGGTTTCTTATTTAGAAAGTATTTTAGTTTCTTCCCAAACATAAACCCCTCAATGGAAGAGTAGTAG
AAGGAATTTGTTGCAAATAATCGTAAAAACAAAACAAATTAAACCTGCCGTTCTTTGAGCTATTTCTCCTCATGT
AAAGGCTTCACATGAAGAGAACGTGATCCTCAATGTAGCCGAGTTCTTTAAGCTTTTAATTTCTGGGAAAATATA
TTACTTGTATATTTCCCTCATAATCTCTTGCCCAACCCAAACAAAGAGTCGGAGCAAATGGTAAGAAAGTTATGA
GAGGAAACTCCCTGCCCGAGCTGGGAAGCTCCGTCCAAACAGAAGAAGCAGAGAGTCCGTCAAGCCTTTACGTAT
CCTTGTCTAAAGAGAAGGGATAGGGATAGGGATAGGGATAGGATAGGGATAAGGGATCTGCTTGCCCCAAACTAG
CTCGGAGTATGGGGCTCACATGCCCGGAGCAAGCGGTCCCCGAGCCCCTTCTCTTTGTGGGGCTGCCTGCATAGA
TGGAACTATGGGGTGGCTGGTAGGGGCCGTGACCCCGTCGGCTCTTTCCTCCACAAAAAAAAAAAAATTAAAAAT
TAAAAAAAAAAAAATTTTAAATTTGTTGGCCGCTGAATAAAATTTTGATATACTTATGGCTCCTCTCAAGTTAAA
TTTTAGTTTCGTCACTGACCAAAAAAGGTAGCCAAGCAAATTGGAGCCGATCCTGACAAGAGGGTGAATCATGAT
TTTGCATTAAATTTTTTCTTTTAATTAGTTTTGTGGTGGGAAAATTGTGTTGATTTCCTGAGATATTTACGAGGG
GGTAATGTAGCATGAGCCATGCGGTGGCTAAGGTGGGTCCGTGGACAAGCATATGCATATGCACGGGACAACTAA
TTTTTGTTTCCTTCCAAAAAGTGATTCTCCTTTTCCAATGCGTGAATACGCCTTGTTGTTTTCTCACCAAGGACA
GTAGGTGGTGGCTTTGAGTAAGGTCCGAAATGAATGGCTCAAAAAAGTAGAAAAAAAGAAAAGAAAAGAAAAAAG
AATGTTCAAAATAAATGCGAAATGTCTTTTGTGCATCTCTCAGAGTCAGGGAGCTATTGTTTTCCTAAAGATTAT
CTTCAATATAATGATCTATTATTATTATATGTGAATGTTATATTAACATTGTGTACTTGTCCTGACCTCAAGGTT
TTGGCTCCACTCCCAATTGGATTTGCGGTCTTCATGGTTCACTTGGCCACCATCCCGATCACCGGCACCGGCATC
AACCCCGCTAGGAGCCTCGGAGCCGCACTTATCTACAACAAGGCCAAAGCCTGGGATGACCAAGTTAGTATATAT
ACCTTTCTTCTTACTGTTGATATTTGTAGCATTTTATCATCCTTGTAAATGCCATTGAATTACAATGTGTCTTAC
ACTCTTTTTCACTTTGTTTTTTTCAGTGGCTCTTCTGGGTTGGACCATTCATTGGCGCAGCTATTGCAGCTTTCT
ACCACCAATTCATCTTGAGAGCAGGTGCTGCAAAAGCTCTTGGGTCATTCAGGAGCAACCCCAATGTT**TGA**CTTT
GATAAGAAGAAGAAGAAGGCTTTCATAGTTTAAGAAGAATATTAATGGGGGATTGGTTGTGCTAATGAGGATCAT
TAAATTGTAGACGAAAGTCTCTTTGAGAAAAGGGAGCTTTTTCTGCCCCTCTTTGTCCTTCTAACCCCACTTGCC
TTTCAATTTACTTCTATGTTGTCTTTTTTTTTCCACTTGTCTCTTTGGTTCTTCTTTGTACTCTTGTGATTTTAA
TTAACGTTTGTACTGAGGATCACTTTTTGCTTTTTTTTGGTGTCTCAATTCAGTGAATTTGGTGCTAGGGGGGAG
ACATGCCCCACTAGATCATACTAGGGAAATGACTTTTAACACTAGGGATAGATGGGCCTGATTTTGCTATGTTTA
ATCTTATGCTTTTGGTAGGTGAAATTTCATCTTCACTATAAAATGAATCTTCCTTATTTTAATACGGATCTGGCT
TCTGTGCTGTAGTGAGAACTAAAGCACAGATGCTGTAAAATTATTGGACGGTTCAGATTTAATCAAATTTCTTTT
AACTTAAATTGGTCGTTTTTAACTCAAAGTCTTCTTCCAATAACACTTTCTCATCTCTTTCATTTCTCTTTC

>*Bpe*PIP2;4

ATGGCCAAGGACGCGGAGGTGCCAGAGCATGGCTCTTTCACGGCGAAGGACTACCACGACCCACCTCCGGCGCCG
CTCTTCGACGCCGTGGAGCTCACCAAGTGGTCCTTCTACAGGGCTTTGATAGCCGAGTTTATAGCCACTTTGCTC
TTTCTATATATCACAGTGCTCACCGTGATAGGGTACAAGAGCCAAATTGACCCCGCCAATGACGGCGAAGCCTGT
GGTGGGGTCGGCGTTCTTGGTATCGCTTGGGCCTTCGGAGGCATGATCTTTGTTCTTGTTTACTGCACTGCTGGG
ATTTCAGGGGGTCACATTAACCCGGCGGTGACGTTCGGGCTGTTTTTGGCTCGAAAGGTGTCGCTGGTGCGGGCG
GTGAGCTACATGGTGGCTCAGTGCCTGGGAGCCATCAGCGGTGTTGGGCTAGTGAAGGCCTTTAATAAGGCGCAC
TACACTAGGTATGGTGGTGGTGCCAACACGCTGGCTGATGGGTACAGCACAGGCGTTGGATTGGGCGCGGAGATC
ATCGGGACCTTCGTGCTTGTCTACACCGTGTTCTCTGCTACGGATCCCAAGAGGAATGCTAGAGATTCCCATGTT
CCGGTTTTGGCTCCACTCCCAATTGGATTTGCGGTCTTCATGGTTCACTTGGCCACCATCCCGATCACCGGCACC
GGCATCAACCCCGCTAGGAGCCTCGGAGCCGCACTTATCTACAACAAGGCCAAAGCCTGGGATGACCAATGGCTC
TTCTGGGTTGGACCATTCATTGGCGCAGCTATTGCAGCTTTCTACCACCAATTCATCTTGAGAGCAGGTGCTGCA
AAAGCTCTTGGGTCATTCAGGAGCAACCCCAATGTTTGA

>*Bpe*PIP2;4

MAKDAEVPEHGSFTAKDYHDPPPAPLFDAVELTKWSFYRALIAEFIATLLFLYITVLTVIGYKSQIDPANDGEAC
GGVGVLGIAWAFGGMIFVLVYCTAGISGGHINPAVTFGLFLARKVSLVRAVSYMVAQCLGAISGVGLVKAFNKAH
YTRYGGGANTLADGYSTGVGLGAEIIGTFVLVYTVFSATDPKRNARDSHVPVLAPLPIGFAVFMVHLATIPITGT
GINPARSLGAALIYNKAKAWDDQWLFWVGPFIGAAIAAFYHQFILRAGAAKALGSFRSNPNV*

***Bpe*PIP2;5**

>**FXXK01000553.1:**54000-60000 *Betula pendula* genome assembly, contig: Contig552, whole genome shotgun sequence
>**Bpev01.c0552.g0003.m0001**
*Bpe*_Chr13 14,364,373-14,366,680 (-1) ::
complement(join(14364373..14364486,14364614..14364754,14365558..14365853,14
366371..14366680))

>*Bpe*PIP2;5
AATAAATACAGCTTGAATTATTATTATTGTGAGTCTTGATATTAATATATGTGTACGTGTTTTTATGTATGTATA
AATATATGTGTGTGCGAGCATAATGAATTTATATACATACACATAAACTTGAGATTAAATTATTTAAAATTCATA
TTGATTTATTTTATTAACAATAATTAATTAATAATTAGTATAGATTTATATAAAAAATAAACAATCGTGATATTT
ACTCTATATAAAGAAATAATAAATTAATTGAATAGCTTGTATAAACAAATTTAAATTCATTCAAAAAATAATAAG
TTAAATTTGAATAAATAATCTAATTCGGTAATAAACTTAAACACAATTTATATTTAAAATAAAAATAAATAAATA
AAAATTAAACATTCAATACTCCACACAACTTTTCTAATCAGTTGTCTACAACCCTCTCAAAGGTCATGCCAAGTC
TAATGGAAGAAGAAAACTAGTGCTGGAGGGCGAGCTTGCAGAGAGAATTACAGGGTCTTATTGATGTTTTTATAG
GCCGTGTCGTTGCGACGTCGTATCCTTACTCTTTTCCTACCGGAAACTGATAACCTCATGCTTGAATCTAGTTAC
TTTTTTACACCAAGCTGGCCTTTTTAACTGTCTTGAACTTGCAATAAATTTCAGAAAAAACATGAGTTTTTGTTT
TTTTTTAAATCTGCGCAGACAAATTGCAATAAATCTCCACTACGTAGAATTTAATATGCTTTTTTTTTTTTAATTG
AAAAAGGAAAAAGATGACACAGAAAAAAATTTTACCACTCTTTATCCAAAAAAAAAAAAAAAAGTCAAAGATTCTA
AATAAAAAATAACAAAATTAAGACTTTTCTGAAAATATGCATTAAACTTTGAACAATATTATAAAATTAAAATAAT
ATTGTAAAAACATTGGGTGTGCTTGAAAGAGAGCATAGTTAAGACTTTTCTTTTTCCCCTTTAAACAAATACTTT
AGGATAAAGGTTTTTTTTTTTTTTCACTCAATCTATTAAATTAATTTATATTTCAAAATATATGTGGAACTCACA
TATTTTATTAAAAATATATGTAATTTTTACATATATTTTAAACAATAGTAATTTAATAGACTAAATTAAAAAAAA
AAAAAATTCAACTCAATTTACAGTTACCCCCTTGTCCAGCATAATGAATGCCTCTTGGTAGTTGTTTCACGAATC
ACAATGCATCCTCTTAAGCAATCAATTATAACCAAAAAAAAAAAAAGGTTCCAACAAAGCTCGAAGACAGTGAAGA
AATAGACTGTTGCCTACTTGCCTGCCTAATCTCGATTAGATGACCGTTTCACTTTTAACTACAACCACACACATA
TATATATATAGCCGTTAAATTTATGGAATTTTGGGCACCATACCGGGACCATCGAAGTAGCTGGAAGAAGAAAAG
GAAAAGAAAAAAAAAGGGAGAAAGTAATCAAGAGCCGTTAGAGGACAGGGTGGGTCCATGCAAGCGAAATCAAGC
AAGATGGGCGGCAAAAAACTATGTTAATCTTATCATTAGTGTCCATAAATAATAATTATTTATGGACTCTTAATT
ATTGACCTAATACTCTCTGCCTCTCCCATAAATACCCCCCCTGTACTCTCCTCTTAATCCACACACACACATACT
GCAGCATAACACAAAACACTTCCTACAGTGAGT**ATG**GCGAAGGACGTTGAGGTTGCAGAGCACCAAGGAGAGTAC
TCGGCAAAAGACTACCATGACCCTCCTCCAGCACCTTTGATCGACTACGACGAGCTGATCAAGTGGTCTTTTTAC
AGAGCTCTCATAGCAGAGTTCATCGCCACCCTTCTTTTCCTTTACATCACAGTCTTGACTGTGATTGGCTACAAG
AGCCAGACTGATCCCTCCAAGAACCCCGATCAATGTGGTGGGGTTGGCATTCTCGGCATCGCTTGGGCCTTCGGC
GGCATGATCTTCGTCCTCGTTTACTGCACCGCCGGTATCTCTGGTATGGTAGCCAATTTATTTACCTTTTTCTTT
TTCTTTTTCCTTTTTTTTTTCTTTTTAAACTTCATATAGTCCCCATAGTCTCCAAAACATCGGTTCCTTTCTGCA
TGTTCCATAATTTTTAAAAAAATTTACTTTGGTATTTTGTTTATAATTTAATTTATTTTCGTTAGATTCATGTTA
AATTTTGTCAAAATTTTTAAAATATTCTTATATTTTTTTTAAAAAACAAATTATAAAATCTTTTTTGTCAAAATA
CTTTCAAAATTCAAGCAGATATTTTTACAAATTTAGTTAAACTCTAAATCTTTGGTTTTTTTATTTCTAAAAAAT
TATAGGTATTTTAAAATTTTTTACATAATTTAACAATGAATCCGATCGATTAAAGTTAAATGATAAATATTCAGA
ATTCAAAAGAACAGAAAGTTAAAGAGAGCTATCTATTTTTTTTATTTTTTATTTTTTATTTTTGGGTTATAACTA
AAAGAAATTAAATGAAATATTTCTAATGTGGGCAGGAGGACACATTAACCCGGCAGTGACGTTCGGGCTGTTTTT
GGCGCGGAAGGTGTCGCTCATCAGGGCGTTGTTGTACATGGTAGCACAGTGTTTGGGTGCAATCTGTGGTGTTGG
GCTGGTGAAGGCCTTCCAGAAGTCTGACTACAACAGGTACGGAGGTGGGGCCAACGAGCTGAGTGTTGGGTACAA
CAAGGGCGTCGGATTGGGGGCCGAGATCATCGGTACCTTTGTTCTTGTCTACACTGTCTTCTCAGCCACTGATCC
TAAGAGGAGCGCAAGAGACTCCCATGTTCCTGTAAGCACCTGATCGCACCCTGTCCTTTAATGCAATTATATTCG
TATATATGCTTTATTTATCGTCTCTTTTTTTCTTTTTTTTTTTTAAGTCTTTATTTATTGTCTTGAATCTTGGTC
TTGCCTACCTAAAAGTGAAAGAGACATGCTGAATATGTGAGCTACTTTTATTAGCCTGTATGTATGTGCCTATTG
AGTTCCGATTAGCATCAGTACTGGCTGAAATACTGTAGCATCAGTTTGAGTTCCGATTAGCATCAGTACAGGCTG
AAATACTGTAGCATCAGTACAGGCTGAAATACTGTAGCATCAGTTTGAGTTCTGATTAGCATCAGTACAGGCTGA
AATACTGTAGCATCAGTTTGAGCTGATGTTTACAATTACCCATGCCTATTATATTCTCTCAAGTGGTGGATTATT
TTCATTTCATTTTTTTTTTTTTGTCTTTACTTTAAAGAAAAGTGATTGAGCCATTTGTGCTTCATTACCTACCTT
TTTGATGGGTTCCGTTTAACTTTTTGAAAAGTAGCTTGGATGGATGAATTTAACTACCTGATTTGACAAAATAAT
CATTTTTTTTAAAGAAAATTCAGCCTATTAATTATTTGGTAGGTAATGTTTTGCATGAAATTATAATTTTGTATG
GAAGTAATTTTTTTTTTTTTAGTAAGTAATTCATGTATATGTTATAATTGTTATCAAAGCCTAATTTTCCTTAAA
AATGAATCCACAAACTTATACAAGTGTTGGATATATATATATGGTGATTTTTGGTTGATTAATTTGTTGTTAATT
AAATGAAAGGTTTTGGCACCACTCCCTATCGGATTTGCTGTGTTCATGGTTCACTTGGCAACAATCCCAATCACT
GGAACTGGTATCAACCCAGCAAGAAGTTTTGGAGCAGCAGTGATCTACAACAAAGATAAGGCCTGGGATGACCAA
GTATATATCTCTTCTTCCTTTCATAATTATTTATATATATATTACTCTTTTCTTTTTCTTTTTCTTTTTGTTAAT
AAATTAATTAAGATCACTTTCATCTTATCATTATCTTTATGGCATGTTGCAGTGGATATTCTGGGTTGGACCTTT
TATTGGAGCTGCCATTGCTGCCTTCTACCACCAATTCATCCTAAGAGCAGGAGCCATTAAAGCTCTAGGATCTTT

CAGAAGCAATGCTTAAAAGAGCTTTTCCCCCCA**TAA**ATTTCCAAAGAATAACAATATTGCGGAGTAAATTAATGG
GAGGGGCCATGGAATTGTAGATAAACATGCCTATGTGGCAGGGGTGTATTTTGTATCCTGTCTTGTCCCTTTTTT
TTTTGTTGGCTTTGCATGTATTTATTTGGTCATTGTCGTACTTTTGCATTTTTACTTCTTTCTTTGTGGTGTATC
CGTATTGTTTCCACATATATATGAAACTAGTAAAATGGACTGTGTTCATCTTATTTGCAAATACTAGCTAATTAA
TTTAATTTCGCTGAATTTTTCTTCTAATTTTCAATGACGGGCCTGAGAAAAATGACTCAGACTTGAGAATGCAGC
ATTAGTCATAGAACTTGAGTTCAAGCCAACCAGAGATTAGGTTACAGAATATTCTTACAGAAGAAATTAACCACC
TGATGGAAAAAAAAAGGGGTTTGCTAGAACACCTTCTAGAGGTCCTTCTAGGGTCTTCTGAATTTTTTTGATTTT
TTTAATCAAATTGGGCCCACCATTCATTGAGAGGTGGGCCCCAATTTGATTAAAAAAATCAAAAAAATTCTAGAA
GGACCCCTAGAAGGACCTTTAGAAGGTGCACAAGCATTTTCCAAAAAAAAAAGGGTAACGAAAAAAAGAGAAGAA
AGGAACAAACTTTTCATATATAAAGTCAAAAAGAAAGCTCAAATTGTATTTTTGCTTTTACAAAGAAAAAAGTTG
TAAACAAATATTCTCTCTCTCTAACCATCCTTATCATCTCTCAATCTGTACAATTATTTAAGGTGAGCTTTAATA
AAGAGGGCTAGCATCAAATTTAATATTAGT

>*Bpe*PIP2;5

ATGGCGAAGGACGTTGAGGTTGCAGAGCACCAAGGAGAGTACTCGGCAAAAGACTACCATGACCCTCCTCCAGCA
CCTTTGATCGACTACGACGAGCTGATCAAGTGGTCTTTTTACAGAGCTCTCATAGCAGAGTTCATCGCCACCCTT
CTTTTCCTTTACATCACAGTCTTGACTGTGATTGGCTACAAGAGCCAGACTGATCCCTCCAAGAACCCCGATCAA
TGTGGTGGGGTTGGCATTCTCGGCATCGCTTGGGCCTTCGGCGGCATGATCTTCGTCCTCGTTTACTGCACCGCC
GGTATCTCTGGAGGACACATTAACCCGGCAGTGACGTTCGGGCTGTTTTTGGCGCGGAAGGTGTCGCTCATCAGG
GCGTTGTTGTACATGGTAGCACAGTGTTTGGGTGCAATCTGTGGTGTTGGGCTGGTGAAGGCCTTCCAGAAGTCT
GACTACAACAGGTACGGAGGTGGGGCCAACGAGCTGAGTGTTGGGTACAACAAGGGCGTCGGATTGGGGGCCGAG
ATCATCGGTACCTTTGTTCTTGTCTACACTGTCTTCTCAGCCACTGATCCTAAGAGGAGCGCAAGAGACTCCCAT
GTTCCTGTTTTGGCACCACTCCCTATCGGATTTGCTGTGTTCATGGTTCACTTGGCAACAATCCCAATCACTGGA
ACTGGTATCAACCCAGCAAGAAGTTTTGGAGCAGCAGTGATCTACAACAAAGATAAGGCCTGGGATGACCAATGG
ATATTCTGGGTTGGACCTTTTATTGGAGCTGCCATTGCTGCCTTCTACCACCAATTCATCCTAAGAGCAGGAGCC
ATTAAAGCTCTAGGATCTTTCAGAAGCAATGCTTAAAAGAGCTTTTCCCCCCA**TAA**

>*Bpe*PIP2;5

MAKDVEVAEHQGEYSAKDYHDPPPAPLIDYDELIKWSFYRALIAEFIATLLFLYITVLTVIGYKSQTDPSKNPDQ
CGGVGILGIAWAFGGMIFVLVYCTAGISGGHINPAVTFGLFLARKVSLIRALLYMVAQCLGAICGVGLVKAFQKS
DYNRYGGGANELSVGYNKGVGLGAEIIGTFVLVYTVFSATDPKRSARDSHVPVLAPLPIGFAVFMVHLATIPITG
TGINPARSFGAAVIYNKDKAWDDQWIFWVGPFIGAAIAAFYHQFILRAGAIKALGSFRSNA*

***Bpe*PIP2;6**

>**FXXK01000484.1**:42681-48008 *Betula pendula* genome assembly, contig: Contig483, whole genome shotgun sequence

>**Bpev01.c0483.g0002.m0001**

Chr*Bpe*_Chr8 4,856,8604,858,776(1)::
complement(join(4856860..4856979,4857091..4857231,4857470..4857764,4858471..4858611,4858613..4858776))

>*Bpe*PIP2;6

GTTTACTTAAAATTAATGTGATTTTTAAAATTATCTTAATATCAAAATTTAATAATAATATATTATAAATTTAAT
GATTATTTTAAAAGTCACATTAATTTAAGTGTACACAAAAAAAACATATAAATGACATGTATCTTTACTCATTTT
TCAATTTTTTTTTGGTACTTTGCAAATAAGGATAATTCATAGTACTTCTTCTAATTTAATCCTCCTTAAATTATG
TGTATTGCAAACGTGACTCTTACATTGCGCTAATGATAAAGAGCAGTTCAGGCTAATTTAACAGTGAATCAGTCT
GGGCATGTGAACTGTAACTTTAGTGGGTCTCATGATTAAAAAAAATTATTTTTTATTTATTTTTTTATCTGATTTA
GCCCAAATTAAAACACTATTTATTTCAGCCTAAACTGATGTGAATCAGTGCTCCTTACATTAACATCTATAAAAG
TGAATACCCTTGCTCCACTAATTGTAGTTTTGTGTAGCGTGTACGGTGTACGTGCAGAATATCTCTTTCTTTTTC
TTTTCTTTTTTTTCTGAGATGCAGAATATCTCTCTCGATGCTGATGGCGCATTACTTAACAAGTCTCATGAATGT
TAAAACAACATTTTATTCAAAAGACGTATTCATTTTTGCCTTTTCAATTATCCCTTACCTTAAAAGGAAAGCATG
AAAAGCCCACTGCCCTGAGTTATTAATGACGTACAAAACTTCTGGGAATGAAAAGCAAAAAACAGAAAAGTAATA
GTCAAAAATTAAAAGGTAAAGCCCAGCCCAAAAAATTGTAAGAACTACAATAAAAAAACAGAAAATTAAGGAGCG
CCACTAAGTCGTTTTTTTTAAAAAAAAAAATTATTTACCACAACCAATGCTAATGAATTGATATCTACTGAGTTA
CAATAAATATTTTTCTCTCAAAGATATAATGGAATCCAAAAATCTTACCTGATTTAGTTGTATTTAGCACAAAAC
AAAACAAAAACATTTTCAAAATTTCTATTCTAACAAACAAGAGAGACTCGAATTTTTGGAAATTACATAATATTA
TGAAAGATTAAAGAATAGGAAATTATGTGTTCTTGAGACTTCGTACCATATTAGAGTATATACACAAGTGAATAA
GCATTGCTTGCTTTCAAACAATAACTTAGTGTGTATTGAGCTATAAATATAAAAGTAATGCTACATGTTGAATTT
TTTTATCTAATTCTATTTAACTATGTATATATGGCTTTTTTTTTTTTTTTTTAATGAGTTTACTTTTATATTTTT
AAGGTTCATCTTTATTTATATATACAAAATTAAATAAAATTAAACATATAAAATTACTCTAAATACAAACTTGAT
TTTCTCTTTTTCCCATATAGAATCTATTCTGCCAAGATAATATTGAATTTAAAACATTTTCCAACCTACCTACTC
TAAAAAACGTTTTCCAAGATAATATTTGTCCACATTATCAAAATATTAGTGAAATTTCAACTTCCAGAACCTACT
CGAGTATTTAGCGGAGGGCGAATCAAAAGGTGCCAGAGAAGCAGAGACATTTACCAAATGGTGGGGTCACAGAAA

GTGAAAGAAACTAAAAATTGGAGATGGGGCGGCAAAGCTAAGCTTATTAATTAACGGACAAAGCTTACTCAACTG
GAAACACCCATATATATATACCCCATCTCCTCTGTTTCTTCCACACCAATACAGAATTTCTCACCAAACCACTCA
CTCATCTCTCTCCTCTCTCCCTCTCATTCACCCTAGTTTGCGCGAGTGCT**ATG**GCGAAGGACATGGAAGTTGGAG
GGCAAAGTGGGTTCTCCGCCAAGGACTACCACGACCCACCACCGGCGCCGCTGATTGATGCAGAGGAGCTCACAA
AGTGGTCCTTCTACAGGGCTATCATTGCCGAGTTCATTGCCACGCTCTTGTTCCTATACATTACTGTCTTGACGG
TAATTGGATACAGAGCCCAGTCCGACACAACCAGGGGCGGTGACGACTGTGGTGGCGTTGGCATTCTTGGCATAG
CCTTGGCCTTCGGTGGCATGATCTTCGTCCTTGTTTACTGCACAGCTGGTATTTCAGGTTGGTCCTCTGTCGCAT
ACACAACTTCAACTTCTTTTTCATTTAAAATTCGAAAAACTTTACCAAATCTCTTTCAAGTCTCTTCAAATGACC
TCAATAGTTAGAAGAAATCTTAATTAATGTATCAATTTTTCTTTTTTTTTATATTTACTGTTAAAATTTTTCTT
TAAATTCAATAAAACAATATGTATAATAAAGATTCAAATTTTGGGTAATATTACAAGTTCAGTTAAATAGTGACC
AATACTTAAATTTTTACAATTTTTTTTTTTAAAAAAAAAGTATTTTTAAATTTTTGACACTTCTCCATTAAAATT
TAACAACAAATCTTAATAGATAAAATTAAAAAAAATAAAATATAAATATATTAAATTAAGAGGTTTTAAACTTTG
AATTACTATTTTAAAACGCGTAAAAATTAAAAAGAAAAATTTAAAATTCGTTTACTAATTGGATAGAAAGTGTGG
GCGGCAATTCATGTCTTATTCTATTACCTTTCTATCATAATATAATATAATAGTTGTGTTGATTTGTATCGAGTT
CGTGTGTGGGTCGAAAATTACGCTAGGATAAGTCTTCTATAAACATCAAAATATTTTGTACAAGTTATGTGGGAA
CTTCCAATTTTCTATAATAAAACAAAGAAAGAAAAGAGCGATGTAGATTTGTAGCATATGGCTAATAAATGGTAC
CAAACTGTGCAGGAGGGCACATTAACCCGGCGGTGACATTCGGGCTGTTTCTGGCTAGGAAGGTCTCGCTGGTGC
GAGCCATTTTGTACATGGTAGCTCAGTGCTTGGGGGCCATATGCGGATGTGGGCTGGTGAAGGCATTCCAGAGAG
CTCACTACACCAGGTACGGTGGTGGCGCCAACGAGCTCTCCGTTGGATACAGCAAGGGCACTGGCTTGGCCGCCG
AGATCATTGGTACCTTTGTGCTTGTCTACACAGTCTTCTCCGCCACTGATCCCAAGAGAAGCGCAAGGGATTCTC
ACGTTCCCGTAAGTACCCATTTTTTGAATTTAACTTAAAAAGGGAATAATTCATAAAATTCCTCCGAATTTTAAC
ATATTTATCTTTAATTTTTATTTTTTATTTTTTCAATTTCATCGATCTGTGCAGATTTTTCGTTAAATCCTATTA
AAATTATCAAAACACCAATTTTTTGAATATGTGAATAAGTCACTTAAACAAATCAATCAGTTTTAGGCTTTAATT
TAATTTTAATTAATCTTGTAGGTATTGGCACCACTTCCAATTGGATTTGCGGTGTTCATGGTTCACCTGGCCACC
ATTCCAATCACCGGAACCGGTATTAATCCTGCCCGGAGCTTCGGAGCTGCAGTGATGTACGGCAAGACCAAGGCA
TGGGATGACCAAGTATGCACCTACCCATCTCTTTTTGGTTCCTTTTTCTCTCTGATTTATGTTACAACTTATACA
GTAACTGGTGCTAACTTAAAAATTTGATTTGATTTATGAAAATTTCAGTGGATTTTCTGGGTCGGACCATTTATT
GGCGCTGCTATTGCTGCATTCTATCACCAGTTCATTTTGAGAGCAGGCGCTGCAAAAGCTCTAGGGTCCTTCAGG
AGCTCATCCAACATA**TAA**ACTGAGCCAAAGATATTGTGGGACTCTGCTTGCCAATTGCTTCAAAGAGCAATGGGG
GTGTGCAACAGTACTGGAATTTGTACATTGTTAAGTTTCTGCCGAGGATGTCCTTCGTCGTACTTTTGTCATGTT
CTGGCAGCACTCATTTTAAATGTGCGCTAATGTTGTTGTTGTCTCCTCTTTTCCTTTGCATTTCTGGTCTTTTGC
TTTTGTTCTGTGTACTCATGTGTTATGGTCGAAATGATCAGCATGTACGCTTTGTTTTGCTTCCTGTCTATCAAT
CTTACGACTTTATTTTAGCAAAGAAGTATAGAGGGGTCCATCAATAAGTCACGGGTGATGCGCAAACTAACATCT
ACTTCATAGTCTAAGGAATAATATGCGTAAAAGTAATCACAACAGCTTTTTTATAATTCATTCTCTAAATTAAAG
GAAAACAATAAAAAAAAAAAAAAAGACTTACATCAGCTTTGTTATTAATTTCTTAAACTTTGAGTTTGTTACAATT
CAATAGTTTTAATCCAAAATTATCCATCTCTTCTTTTCTTTTTGTCTTTTATTGGGAATTGTATTAATATTTATT
AAAAAAAGTGAGGTAAGTAAAGAATTTATATTTGTAAAAAATAATATTTTAATGGTGTAAGAAAAAAAAATATT
ATGAAGTTGGTTTGAATTGAAAATAAAAGTAATTTTAATTCATAAATTAAAGAAAAATAAAATAAAAAAGCTTCT
GAATGTGAATGCTCTCATACATAGCAAAGTTTCTTCATATATAATGTGGGCGGGCAATTTGAATTGTTGGGTTAA
TTACACTCAACCTCGTTGGTAAAAGAAGTCTTTTGTTAGCCGAACTCATTAAAATAGATGGAATCTCGATCATG
TGCAATACACGAGCATTTTTCTTTTAGATTTAATATGTCTCTTTTGCCCTTGTTATGTATGTTGTAATTCCTACA
TTTAGCATGGGCTTCCAAGGAATATCAAATAAACAACTCCAAGTGTATATTCTGATACATCACATTAACAAAACA
TTTTATGGCTTCGCCAATTTTAGTT

>*Bpe*PIP2;6

ATGGCGAAGGACATGGAAGTTGGAGGGCAAAGTGGGTTCTCCGCCAAGGACTACCACGACCCACCACCGGCGCCG
CTGATTGATGCAGAGGAGCTCACAAAGTGGTCCTTCTACAGGGCTATCATTGCCGAGTTCATTGCCACGCTCTTG
TTCCTATACATTACTGTCTTGACGGTAATTGGATACAGAGCCCAGTCCGACACAACCAGGGGCGGTGACGACTGT
GGTGGCGTTGGCATTCTTGGCATAGCCTTGGCCTTCGGTGGCATGATCTTCGTCCTTGTTTACTGCACAGCTGGT
ATTTCAGGAGGGCACATTAACCCGGCGGTGACATTCGGGCTGTTTCTGGCTAGGAAGGTCTCGCTGGTGCGAGCC
ATTTTGTACATGGTAGCTCAGTGCTTGGGGGCCATATGCGGATGTGGGCTGGTGAAGGCATTCCAGAGAGCTCAC
TACACCAGGTACGGTGGTGGCGCCAACGAGCTCTCCGTTGGATACAGCAAGGGCACTGGCTTGGCCGCCGAGATC
ATTGGTACCTTTGTGCTTGTCTACACAGTCTTCTCCGCCACTGATCCCAAGAGAAGCGCAAGGGATTCTCACGTT
CCCGTATTGGCACCACTTCCAATTGGATTTGCGGTGTTCATGGTTCACCTGGCCACCATTCCAATCACCGGAACC
GGTATTAATCCTGCCCGGAGCTTCGGAGCTGCAGTGATGTACGGCAAGACCAAGGCATGGGATGACCAATGGATT
TTCTGGGTCGGACCATTTATTGGCGCTGCTATTGCTGCATTCTATCACCAGTTCATTTTGAGAGCAGGCGCTGCA
AAAGCTCTAGGGTCCTTCAGGAGCTCATCCAACATA**TAA**

>*Bpe*PIP2;6

MAKDMEVGGQSGFSAKDYHDPPPAPLIDAEELTKWSFYRAIIAEFIATLLFLYITVLTVIGYRAQSDTTRGGDDC
GGVGILGIALAFGGMIFVLVYCTAGISGGHINPAVTFGLFLARKVSLVRAILYMVAQCLGAICGCGLVKAFQRAH
YTRYGGGANELSVGYSKGTGLAAEIIGTFVLVYTVFSATDPKRSARDSHVPVLAPLPIGFAVFMVHLATIPITGT
GINPARSFGAAVMYGKTKAWDDQWIFWVGPFIGAAIAAFYHQFILRAGAAKALGSFRSSSNI*

TIP – Tonoplastic Intrinsic Protein

***Bpe*TIP1;1**

>**FXXK01000279.1:**16272-18583 Betula pendula genome assembly, contig: Contig278, whole genome shotgun sequence
>**Bpev01.c0278.g0002.m0001**
Chr Bpe_Chr8 6,808,526-6,809,382 (1)
::join(6808526..6808909,6809008..6809382)

>*Bpe*TIP1;1
CCACCGTCCTTGAGATCTTCGGGGCCACTTGATTTATTTATGGGTGGCCAATAACTATTGCCATGTGTCATGTCA
GCATCAAGCACGACAAAGATCCAATGCATCGCAAATTCACTCCCTGCTTATCCCCATACCTCATTTCTTTTTCAT
ATATAATAATATTTCATGTCTCAATACGCTATATATAATTCGAAGATTATTTCGAAATTAGTTTTTTTAATTTAC
CCTATAACAGTTTTTTTTTTTAATTCAATATCTGAAAAACAGGAACAAAGGAGATTATATAAGAGCATCTTTGTC
ACTCTTTAAGCGATTTTATACAAGAACAGTAATACAAGTCAATTCACTAAGATTTTTTAAAATCAATATGTGCAA
AATCTTACATGACGGCCACTGATGACATCATATTTTTAAACGATAAATTATAATTTTGTTAAACGTATAACTAAA
TTTTTTATACCGTAAATTTAAAATATTTCAAAACCGAAAAATCAAAAAATACATTAATATACAAAAGTTATTCGA
TCTTTCGAGAGGCCAAGCAAATCAGACAAAAAAAACACCACTATACAGCACGTTACGTGGCTCTTATCCTTGCAG
CACACGTGGCGATATTTCAGCAGTGTAATACCCAGTTGCTTTGCTGTACGAACAGGAATGGCTGGTAGATGGTCT
CCCCCAAAAATTGAAAAAAAACCAAAAACAAGATCACTATTCAAGTATTCACAGCCTGTCCACCAAGCACGTTCT
AAAATTATTATTACTTGCTACCGGAAACCGGCTCCAGCCGGTATATTAGCGCTCGTCACGTTCAATATAAACCCA
CATGCTGAGAAGCGGAGAGCAGTACCAACAACCAAGGCTAAAACGCAGTAGTGAGTGTACGTAGCTACAACAACT
CATCAAACGACC**ATG**CCGTTCAGGAATATTGCCGTCGGGAGGCCGGAGGAGGCCCGCCACCCGGATACACTGAGG
TCGGCGTTGGCGGAGTTCATTTCGACGCTCATTTTCGTGTTTGCCGGGGAGGGCTCCGGCATGGCCTTCAACAAA
CTCACCGACAACGGCTCCACCACCCCGTCTGGGCTCGTGCAGGCTGCCCTGGCCCACGCTTTCGGTCTTTTCGTC
GCCGTCTCCATCTCCGCCAACATCTCCGGCGGCCACGTCAACCCAGCTGTCACCTTCGGCGCCTTCATTGGTGGT
AACATCACCCTCCTCCGCGGCATCGTCTACTGGATCGCCCAGCTCCTCGGATCCACCGTCGCTTGCTTGCTTCTT
AAGTTCGCCACTAACGGCTTGGTGAGGCTACCCGTTTTCTGTTAGTTTGTTACAGTTAGTCTGACACTTAATTTT
GTACATTTAATTATTTAATTAAAATTAAATTTGTACTTTCTCAGACCACCAGTGCTTTCTCTCTGTCGTCGGGAG
TAGGAGTGTGGAACGCCTTCGTTTTGGAGATCGTGATGACTTTCGGTTTAGTCTACACAGTCTACGCGACGGCCG
TTGATCCAAAGAGGGGCAGTGTGGGAGTTATAGCCCCTATCGCCATCGGTTTCATCGTGGGCGCTAATATTTTGG
CGGGTGGGGCGTTCGACGGGGCTTCGATGAACCCGGCGGTGTCGTTTGGACCCGCCCTGGTGAGCTGGACATGGG
AGAACCACTGGGTCTACTGGGCCGGGCCTCTGATCGGAGGCGGGCTCGCGGGTCTTGTGTACGAGTTCTTCTTCA
TCAGCCACAATACTCACGAGCAGCTGCCCACCACAGACTAC**TGA**TGAGCAAGGGAATCTGAAAAGAACAGTGGTG
GCACGGCTTTTCTCTGCTGTATTTACTTGTTTATTCGAGGGTTGTTCTTGTGTTTTTTTTTTAATTAAATTGGTC
ACCGTTGATCTTTAATTATCACCATCATGATCATCTAGCCACTGTTGTTTCTCATCACTGAATGTTATGATTGTA
ATTCTCAAGTTGGGGTGTGGTTGTGTTTATCCTGCTCTGCGTCGGTTGTTTGATTATTTTTTTTAAAAAAAATTC
TGTTCTTTTCTCCTTCTTATTAACTTCAATTTCAACGTCCTTTTTTTTTTTGGATTTTTCCCCTGTTCCGTATTT
CAATTATTGAATTTTCTTTGTCCGGTACCGGAAGCATCTAGCACGCCGTACGGTTGAGCTGTAAATAGAATCACG
TACATGCATGTCCATAGAAATTACTGTAGGTCGACCGTCTGGTTAATGAATCTGATCACCTGCCTCATCGGGGGC
TGTGAAATGGAGTGTACCCATTTCATGATCAAATTGGTGTGTTTAACTTAGTAAGTTATG
>*Bpe*TIP1;1
ATGCCGTTCAGGAATATTGCCGTCGGGAGGCCGGAGGAGGCCCGCCACCCGGATACACTGAGGTCGGCGTTGGCG
GAGTTCATTTCGACGCTCATTTTCGTGTTTGCCGGGGAGGGCTCCGGCATGGCCTTCAACAAACTCACCGACAAC
GGCTCCACCACCCCGTCTGGGCTCGTGCAGGCTGCCCTGGCCCACGCTTTCGGTCTTTTCGTCGCCGTCTCCATC
TCCGCCAACATCTCCGGCGGCCACGTCAACCCAGCTGTCACCTTCGGCGCCTTCATTGGTGGTAACATCACCCTC
CTCCGCGGCATCGTCTACTGGATCGCCCAGCTCCTCGGATCCACCGTCGCTTGCTTGCTTCTTAAGTTCGCCACT
AACGGCTTGACCACCAGTGCTTTCTCTCTGTCGTCGGGAGTAGGAGTGTGGAACGCCTTCGTTTTGGAGATCGTG
ATGACTTTCGGTTTAGTCTACACAGTCTACGCGACGGCCGTTGATCCAAAGAGGGGCAGTGTGGGAGTTATAGCC
CCTATCGCCATCGGTTTCATCGTGGGCGCTAATATTTTGGCGGGTGGGGCGTTCGACGGGGCTTCGATGAACCCG
GCGGTGTCGTTTGGACCCGCCCTGGTGAGCTGGACATGGGAGAACCACTGGGTCTACTGGGCCGGGCCTCTGATC
GGAGGCGGGCTCGCGGGTCTTGTGTACGAGTTCTTCTTCATCAGCCACAATACTCACGAGCAGCTGCCCACCACA
GACTAC**TGA**
>*Bpe*TIP1;1
MPFRNIAVGRPEEARHPDTLRSALAEFISTLIFVFAGEGSGMAFNKLTDNGSTTPSGLVQAALAHAFGLFVAVSI
SANISGGHVNPAVTFGAFIGGNITLLRGIVYWIAQLLGSTVACLLLKFATNGLTTSAFSLSSGVGVWNAFVLEIV
MTFGLVYTVYATAVDPKRGSVGVIAPIAIGFIVGANILAGGAFDGASMNPAVSFGPALVSWTWENHWVYWAGPLI
GGGLAGLVYEFFFISHNTHEQLPTTDY*

***Bpe*TIP1;2**

>**FXXK01000397.1:**206000-211000 *Betula pendula* genome assembly, contig: Contig396, whole genome shotgun sequence
>**Bpev01.c0396.g0015.m0001**
Chr Bpe_Chr13 17,015,493-17,016,481 (1) ::
join(17015493..17015625,17015770..17016020,17016107..17016481)

>***Bpe*TIP1;2**
AAGGTCGTATATAGAGAAATGGAAATAATATAGTTCTATAGAGAAAAACAAACAAAACGGGAGTCCCCTTTTTAT
CTGTTGTCCTTTGATTTTTATAGGCAACTTGTTGTTGGCCATGTGGCACGCCCCGATTGGAGGATTCTTTGTCGT
AGTATTCTCTAGGCCTTATGATCCCCGCTTATCATAAATTTGTACTGAGATTGCGGGAATCAATGTTGACAACCT
AAGGGATGAGTTTTAGTGCAGTGCTTGGTGCACTCAAGTTCATTGGGTGATGGGCCAAGCATTCGGGTTGGCATG
CCTCATAGGGTTGCTAGGCCCAACAAAAGGTTTTTTTCTTTAAAGGGTTGGAGAAGAAAATCAATTGTGGCTAG
CACCAACTTGCAGAGGATTGCTCAGGAAAAGCTTAAACGGCGAAAACCGCATGTGCTTCTTCAAAATCAGTTGAA
TTATAGCTTAACACCCACCTTACCAGAACATCAATGAGACATAAAGATTCCTATATTTGAGAGTCAAACCCATAT
CATTGCATTATCCAAACCTAATGAGAATGCCCTGAGACCATCGAACCACCACCCTTGCCCAACAAAAGGTATTGT
AAGGACTTCCAAGTTGCAATCCAATCACATGAACTGTGTTTCAAGTGGCTGAAATCAAGTGGTTAAAATTAAAGC
AAAACATCTATTCCCTGTATGGGCACACAGCTCCATATAACAATATCATAGGACCGAATACACCACATCATCCCC
TGCGTCTCTTCATGCACTAAACAAAATTAAGGATAATTAAGTGATGATCACTGTTCATGATAATGAAGCCACCTC
AACTAACCACGGAGGTCTACATCAGAGCCGCATAAACAATCCAATGGTCGGTCAAGCCAAGCCGCAGCAAACAAT
CATTGGTGGACCCACCACCACGCCACAACCATTTTGGTCGGTGATTAGAATTTCTTAACTATGCGATCATCCGTC
CACGTGGCAAGCTGAGAACAAAACGGAGGCCTGGCCTAAAACCTTTCAGTGACAAGGAAAACGAGCCCTTATTTT
TCTCATTCAAAACAAACTGTCACCGGTTAGAAACCGGCTATTCCGGTTGTCCAAACGACAGTTAACCTTAGCCTT
TCCTGTATATAAACTACCTCCATGTAAAGCTAGAAACCAAGGCAAGAAGCAACAAAAAGCTAGTGCAATTTGGAT
TTCGAGTCTAAATTGAGATTTTAGAAAA**ATG**CCGATTAACCAAATTGCCGTTGGACACCCGGCCGAGTTTGGCGA
TTCCGGTGCTCTCAAAGCGGCCCTGGCTGAGTTCATCTCAGTGCTCATTTTTGTGTTTGCCGGTGAAGGCTCTGG
GGTGGCTTTCAGTAAGGCCCTGACTGACCCTTTTTGTTTCCAACTTCTTTGGAGCCTAAGTCATAGTTTTGTTTC
TTTCTTTTTGTTGTTATAATTAAGATAACTGAGAAACTAAACTGATAATTGAAATGTTGAATCGATGAATGTTTT
GGCAGACAAGCTGACAGATAATGGCTCGACAACACCGGCCGGCCTTGTAGCCGCAGCCTTGGCTCATGGCTTTGG
ACTGTTCGTGGCAGTGTCAATTGGTGCAAACATTTCCGGCGGGCACGTGAATCCTGCTGTTACATTCGGCGCCTT
CCTTGGCGGGCACATTTCACTCATTAGGGGTCTTTTGTACTGGGTCGCCCAGCTTCTTGGATCTGTGGTTGCTTG
CTTGCTTCTTAAGTTTTCTACCGGTGGACTGGTAAGGCCTTCACAGATATATATAGATGTACTTTCAATATATTT
TGTTAGATTTAGATTTGTGTTTAAATCAATTATTTGATGCAGGAAACACCCGCGTTCTCTTTATCTTCTGATGTG
AGTGTATGGAATGCGGTAGTGCTTGAGATTGTGATGACATTTGGGTTGGTCTACACCGTGTACGCCACAGCCGTG
GACCCTAAGAAGGGAAACATAGGGATACTTGCACCCATTGCAATTGGTTTGATTGTGGGCGCCAACATTTTAGCC
GGTGGTGCCTTTGACGGTGCATCCATGAACCCAGCAGTCTCCTTCGGCCCTGCTGTCGTCAGTTGGTCATGGGAC
AACCACTGGGTCTACTGGGTAGGCCCACTCATTGGTTCCGCCATCGCCGCCATAGTCTACGAGCTCATCTTCATC
AGCCCAAACACCCATGAACCGCTCCCAGCCACAGATTTC**TAA**GCAATAAAGACCTCTTTCTCTGTTCTCGGTTAT
GGAAAATGGGTTTCTTTTGGTTTCAGCTATGGGTGTTTTGGTGTTGTTTGTACCCTTTTGTTTTGTCTTTTTTTT
GTTTTTTTTTTTTTTTTTTCTATTCAATACAAGGGGAAGGGAAAAGGAAAATTAGGTCTTTACCACTCCTGTAC
CCTTCTATTGTTAATTGTTGATTCTTGTGTAACTGTCTTGTATTCCTGCAACTCATTTTGATCAGTAATGTCCAT
CTGTGCTCAGTTTAAGAATTTTCCTTTTTGTCCCTTTTTAGTTTTTGAAAAGTTTTAGGTGTAAAGGGGTACCCC
GTGCCCACCATGACTTGAAAAAAAAATTAATTAATTTAAAAAATTAATTAATTTAAAAAATAAAAAATAAAAAAA
AAGGTTGGTGGGTTTTAGTAGTTTGGATCACTAATAAAATTGCCATGTTAGCAGCATATTCCTTAGGTGCCATTC
TGCCCTATAAACCATAATGTAATATATCAAATAAGAAGAAGATTTGAATAGAAACAAAAAAATAATAATAATTTT
TAAATTTCCTCGCCAGCACTCTTAACCACCCCAACAATGCCCAGCCCACTGATTTGGCTTCCAGCCACGACACGA
GCACTCTTTAGAGTCAATGC

>***Bpe*TIP1;2**
ATGCCGATTAACCAAATTGCCGTTGGACACCCGGCCGAGTTTGGCGATTCCGGTGCTCTCAAAGCGGCCCTGGCT
GAGTTCATCTCAGTGCTCATTTTTGTGTTTGCCGGTGAAGGCTCTGGGGTGGCTTTCAACAAGCTGACAGATAAT
GGCTCGACAACACCGGCCGGCCTTGTAGCCGCAGCCTTGGCTCATGGCTTTGGACTGTTCGTGGCAGTGTCAATT
GGTGCAAACATTTCCGGCGGGCACGTGAATCCTGCTGTTACATTCGGCGCCTTCCTTGGCGGGCACATTTCACTC
ATTAGGGGTCTTTTGTACTGGGTCGCCCAGCTTCTTGGATCTGTGGTTGCTTGCTTGCTTCTTAAGTTTTCTACC
GGTGGACTGGAAACACCCGCGTTCTCTTTATCTTCTGATGTGAGTGTATGGAATGCGGTAGTGCTTGAGATTGTG
ATGACATTTGGGTTGGTCTACACCGTGTACGCCACAGCCGTGGACCCTAAGAAGGGAAACATAGGGATACTTGCA
CCCATTGCAATTGGTTTGATTGTGGGCGCCAACATTTTAGCCGGTGGTGCCTTTGACGGTGCATCCATGAACCCA
GCAGTCTCCTTCGGCCCTGCTGTCGTCAGTTGGTCATGGGACAACCACTGGGTCTACTGGGTAGGCCCACTCATT
GGTTCCGCCATCGCCGCCATAGTCTACGAGCTCATCTTCATCAGCCCAAACACCCATGAACCGCTCCCAGCCACA
GATTTC**TAA**

>***Bpe*TIP1;2**
MPINQIAVGHPAEFGDSGALKAALAEFISVLIFVFAGEGSGVAFNKLTDNGSTTPAGLVAAALAHGFGLFVAVSI
GANISGGHVNPAVTFGAFLGGHISLIRGLLYWVAQLLGSVVACLLLKFSTGGLETPAFSLSSDVSVWNAVVLEIV

MTFGLVYTVYATAVDPKKGNIGILAPIAIGLIVGANILAGGAFDGASMNPAVSFGPAVVSWSWDNHWVYWVGPLI
GSAIAAIVYELIFISPNTHEPLPATDF*

***Bpe*TIP1;3**

>**FXXK01002331.1:**93891-100322 *Betula pendula* genome assembly, contig:
Contig2330, whole genome shotgun sequence
>**Bpev01.c2330.g0012.m0001**
Chr *Bpe*_Chr5 3,465,120-3,466,550 (-1) ::
complement(join(3465120..3465494,3465873..3466123,3466418..3466550))

>*Bpe*TIP1;3
TCTTGTTAAAAGAGAGTCTAATCCTAGAACCCACATAATATTTAGGATAAGCTGCTTGGTAGTAGTAATCTTATT
CATCATCTGAAAAAAAATCTTGACTAATGCCACTTGCTCATTCTTTTTTTTTTTTATTCTTTTTTTGTAGTACAA
GAAAAAAATATTGGTGCAATTGCAAACAGTATTCAAACTTCATCTTAGAGCATCCACATTAAATTCTTCAAATTT
TAAAGAACAACCCTAAATTTTCTATTTTAAAGAACCAGTGAAAAACATTCTACACCTATATATCCTTTCTTTTGG
AGAATGGATGATTTTTTTTTTTTATTCTTTCTTTTAGGCCTAAAGAAAGAATAATTAAAGAGAGAAGTAGAGTTTG
AAGAGAGAACTAAATAAAATAAGAGACAAAAGATCTATAATGATTCTTCATATATTTGAAGGATGAGCAAAAATT
GGATGTAGGGTGATTCTTTATAATTTTTTTTGAAAAAATTTGTCTTGAGGTTCGGATGTAAATGATCTTACAACT
CAATCCTGATATTACTGGATAAAGATTTCTTTTAAACTTGGTAAATGAACTTCCTTTAAATAGTCTATAAATATG
TTATATATCTATTTGCAGGTAAAAATCACACACATACACACACACATATATATAATTGCAATAATAAAGTTAAAA
GAAATTTTATGAAAAAGTCAAGTTCTTTTCATATACAAAAAGATACATGCATATCATTTTTATAGATTTGATCCG
AAAAAGTTTCTCCAACTTAATTTAGAAAAAAATTCTATCCAATATTACCTAGTTAGGATCAAGATTCTCTAAAAT
TTTATCATATTGTACCATGTAAATTTTTTCAAATCTTAACAATTATTTTCAAATTAAATTACATAGTTTTTGTCA
TATCACTCAAAAATATGATAACTATTAAGATGATAAATGCTGAATTATTAAAAATGACGAATATTGGGCACCAAA
AAAAAAAAAACTGATATAATAAAATTTTAACCACTAAATTTGAAGAAAATATTAGAGGATTGGAATAAATTTCCT
AGAAAATCTCTTGATCCATCTATGATCTACCTACCTACTTTTCCGTAATTTCATATACCGATTGCATGCACACCA
AAAGGCACAAGATTCAAAAACGTGCACAGTAGACCACCGCATGATGGTTTCTATATTAAGAGGTGTGGAAGCCAG
TGGGGCCATTAGTTAGAGCCGAAGACAACCTAACCTCCATGGTCCCACCAACATACTACAAAAACCCCCCGACCC
TTAAACGCACATGTTTAAATATTCGATGGTCGGTTCGTCCCAATCAAAAAAATCGCCGCCCATATCGGCGCCATG
AGGTTTAATTGGCCGAACATCACGCGAGGGCCCATCGTGAGTCACTGGTCGGCGATCCTTTGATTTCTATTCCTA
TCCTCGCCGATTCAATCGTAGCTAAATTCGATCACAACCACACACGTGGCATCAGAGGACGTACTCGAGGCCCCA
TTCCCTGGCTGTAAAGTGACCGACATGCAGCTTCGAGAAAAGCCCCGCCTCGTTGGTCTCACGGTGAATGTCTTT
ACTGGGAACGGGTTAGACCGGTTCCCTAGCCGTTATTTTAGGTATATAAATAGGAACTCCCACAAAACACATCTA
TATATTTATGAGCTTTTGACAAAATCAAGTTAGAGAAATAAGCAAAAGAGACTTCCAAAAACTTTCGACTCTCAA
TCTCAAGAGCAGCTGAAATTCAAGAAA**ATG**CCGATTTCTAGAATTGCAATTGGTAATCCGGAAGAGGCGAGCCGA
CCGGACGCGTTTAGAGCCGCATTCGCTGAGTTTTTCTCTATGCTTATTTTTGTCTTTGCTGGAGAAGGCTCTGGC
ATGGCTTTTAGTAAGATCTCAATTTCAAAAACACATCCTTTTTTTTTTTTTGAAGTGAGAAAAAACACATCCAAT
TTAATATAAGATTAAGTAACCCAATAGATTTTTGGGCCTAAAAATTACGTTCGACTGTTATATTCTAATGTTTTT
CTTAATTTTGGTTGTAAAAAATTTCCTGATTTGTTTATTACAAGCTTTGTGACATTACATGCTTTGGTCACAAAT
GATGACACCCCACCCACCACCCAAAAAAGTGTTGCTTTCTTGAAATTTGCAAGGACTAATTGGTGGATGTTTTTG
GCAGACAAGCTTACTGATAATGGGTCAACTACACCATCTGGGCTTGTAGCTGCAGCGTTGGCTCATGCATTTGCT
CTTTTTGTGGCTGTTTCTGTTGGTGCAAACATTTCTGGGGGGCATGTAAACCCTGCTGTGACTTTTGGTGCTTTT
ATTGGTGGAAACATTACTCTGTTGAGGACCATTCTGTATTGGATTGCACAGTTGCTCGGTTCAGTTGTAGCTTGC
TTGCTTCTTAAGTTTGCCACTGGTGGACTGGTAAGAATAGGGAAATGAGAATTCACATCGTAGACTAACCAATTT
AATGCACTTTTTTGTCTTTGTCTATGATGAAGACTGCGTTTAGCATTGCAATTTTGCATGTTTATAAAATGGATC
ATTTAGGAGTCTACTTTTAAAAACTTGTGATTTGAAAACGTAAAAAAATATGCATTTTAAATTCTAGACAAATGA
GTGCGTTTTTAAAAACACAAATTTAAAGGTTAAATTGTAATTTTAAATGCAATTGTATTTTTTTAAATTGCGTTT
TAAATTGCAAATCCAAACAGCCCGGAAGCATCGACTGAAAAAGGGTTTTGGGATAAAACATAATGGTTGGTGCCT
AGCTAACTTGAATTTGAACTTGATCCACTGCAGGAAACATCAGCATTCTCCCTATCATCTGGCGTGTCTGTATGG
AATGCGGTAGTTTTTGAGATTGTGATGACCTTTGGGCTTGTCTACACTGTGTATGCCACGGCAGTGGATCCAAAG
AAGGGGAATGTAGGGACAGTTGCACCCATTGCAATTGGTTTCATTGTGGGTGCCAACATATTAGTTGGTGGTGCC
TTTGATGGTGCATCCATGAACCCAGCAGTCTCCTTCGGCCCTGCCGTGGTTAGCTGGTCATGGACCAACCACTGG
GTCTACTGGCTTGGCCCAATGGTCGGCGCAGCCATTGCAGCAATTGTCTATGACACCCTCTTTATTGGCGAGCCT
ACACACGAGCCACTTCCCACTAATGATTTC**TAG**GAATATTGATTTAAGCCTCTCCCTTTCTCTAGATTATTGAGA
GAATAAAGATGGGGAAAGAAATGCGATTGTTCTTGTCATAGTCTGGGGAATGGAATTGAATTTCATGTGTACTTT
TGTTGTAGGTGTTCTTGTTTTTTCATTTATGCTCTAGGAAAGGGTGATCGAGCTTCTTGAATGTCTTGCTAAATC
AGCGAATGAATTGTTTGTTTTGAACTTACAAAAAAAAAAAAAAATTGTTTGTTTTGCAACTTGCTTAATAACTGCG
TTTTGTTCTGAACAATGATCAGAGAAAACAGAAACTAATTGAAGTTTTGTGTAACTAGCAGCCGAAATTTTGATG
GATCCGAGGAGGGGTAAAACAACCTTATTATAACGGGATCTCGATCCGATAAAGACGGACTGTTTATAAGCTAAA
GATACACCTAATCTCCCAATTACTATGAATGTAGATAGGGGTTCCAATAGAAGTCCTTCCATTTTCATTTGTAAT
TGTGAACTCAATATTGGATCAATAAAAGTAGATTATATCAAGAAAAAGAACAAGTTCAAAGAGAATGGCTGCCAA

AAAACAAAGAGATAAAAAAGAGGGAAACTACTTAGGTTTTTCTATAGTTTCAAATAGGTTCATGTTAACTTATCG
TCTAATTAATTAACGATATGTCATGTCAGACTAATCAATTATTACTACATGACTCTCGTTTGACATGTCATATGC
CAACTATACATGTTACATGACACAGTTTCTTTAAATTACAAATATACATTTTTTTTTTTATAAAAAAAAGTTTGA
AAAAAATTCATTATTTAGGGTATATTAGTAATTTAAAGTAACTATGCAAAGTTGGCAATGACGTATTGAACGATA
GCCACATGGTATTAATTGATGAGTCTGACATGACATACTGTTAATTAAATGGATGATAGGTTGACGGAATGACTT
ATTGAAATCAAAGGAAAACTTAAAAACTTAATTATCAAAATTGGAAATCTAAGGATCAAATTGAAGTCGCAGGAA
AATTTAGAGACTAAATTTGATTTGATTTGATTTTTTAATTTTTAATTTTTTTTTAAAAAAAAACAAAAGTTGTGT
GAACCCATCTCTCACCAACTGGCCGAGGCGCAAATGCATATTTGTATACGAACATAAAGCCAAGCACAAAAAAGT
GAAAACGATATGGTACACAATGAAGATTGGGATGACCATGAAGTTGGTTTTTGTTTTTTGAGTTCAATCTATTTT
TTTTTTTTATGGATGACTTGACACTTGTTGATGTGGCACTAACATTGAGTGTTGAAATAAATGTTGGTGTGTCAA
ATGAGATCCACGTGGCATATGACCAACTGTTGACTTTGACGTGAAGTGACAGAAGAACCTCTTTAAAACTTAGGC
>*Bpe*TIP1;3
ATGCCGATTTCTAGAATTGCAATTGGTAATCCGGAAGAGGCGAGCCGACCGGACGCGTTTAGAGCCGCATTCGCT
GAGTTTTTCTCTATGCTTATTTTTGTCTTTGCTGGAGAAGGCTCTGGCATGGCTTTTAACAAGCTTACTGATAAT
GGGTCAACTACACCATCTGGGCTTGTAGCTGCAGCGTTGGCTCATGCATTTGCTCTTTTTGTGGCTGTTTCTGTT
GGTGCAAACATTTCTGGGGGGCATGTAAACCCTGCTGTGACTTTTGGTGCTTTTATTGGTGGAAACATTACTCTG
TTGAGGACCATTCTGTATTGGATTGCACAGTTGCTCGGTTCAGTTGTAGCTTGCTTGCTTCTTAAGTTTGCCACT
GGTGGACTGGAAACATCAGCATTCTCCCTATCATCTGGCGTGTCTGTATGGAATGCGGTAGTTTTTGAGATTGTG
ATGACCTTTGGGCTTGTCTACACTGTGTATGCCACGGCAGTGGATCCAAAGAAGGGGAATGTAGGGACAGTTGCA
CCCATTGCAATTGGTTTCATTGTGGGTGCCAACATATTAGTTGGTGGTGCCTTTGATGGTGCATCCATGAACCCA
GCAGTCTCCTTCGGCCCTGCCGTGGTTAGCTGGTCATGGACCAACCACTGGGTCTACTGGCTTGGCCCAATGGTC
GGCGCAGCCATTGCAGCAATTGTCTATGACACCCTCTTTATTGGCGAGCCTACACACGAGCCACTTCCCACTAAT
GATTTC**TAG**
>*Bpe*TIP1;3
MPISRIAIGNPEEASRPDAFRAAFAEFFSMLIFVFAGEGSGMAFNKLTDNGSTTPSGLVAAALAHAFALFVAVSV
GANISGGHVNPAVTFGAFIGGNITLLRTILYWIAQLLGSVVACLLLKFATGGLETSAFSLSSGVSVWNAVVFEIV
MTFGLVYTVYATAVDPKKGNVGTVAPIAIGFIVGANILVGGAFDGASMNPAVSFGPAVVSWSWTNHWVYWLGPMV
GAAIAAIVYDTLFIGEPTHEPLPTNDF*

*Bpe*TIP2;1

>**FXXK01000666.1:**158007-164214 *Betula pendula* genome assembly, contig: Contig665, whole genome shotgun sequence
>**Bpev01.c0665.g0011.m0001**
Chr Bpe_Chr5 13,209,768-13,211,363 (-1) ::
complement(join(13209768..13210136,13210701..13210948,13211237..13211363))

>*Bpe*TIP2;1
TCACCATCAAAGTTAATGATTTTTTTTATAATGCTACAAATTAAAAAGATTATTACGACAAAGAATATTGTTTTT
ATTTTTGAAGTTGGCTTTTTTTTTTTTTTTCCAATAGATGATTTAGAGCTTAATGATATAATATTGATATTAGGT
GTTAATATGAATGCTAGTGTGTCAATTGAAATACACGTAACATATGACAAACTATTTATTTTGATAAAAAATGAT
AGAATGACATATTTAAAATCTGGATAAAATTCAATGACCATATTCTTGAAATTAGAAAGTTAAAAACTATATTTA
AATCAAATAAAAATTTAAGAGCTAAATATAATTTTTTCTTAAATATTATAAACAAACTCCATAAAATTATAAGAT
TCTAGATGTAAAAACGGAAGAGCAACCAAAAAAAAAAAAAAATTTGCCATGATTTGTCCCAATTCATGAAAAACA
AGCAGCAAAAAAAATTGTCAAAAAAAAAAAAAAAATTAACTATGCCCCTCCCCATAAAACTTTCTGGCTCCTGATC
ATACATACATGATACATAGGGTGGAAATCTAAAATCACCCTTTAACTCATCATGCACGCAAAAAGAAGAGAAAAA
GATCGAGTAAAAAACCCTTACGATGAAAGTGGTCATGAAAGTCAAACAATTAACGGGATTGTTTTCCAAATAAAA
CCAACAGGCTAAAAAGGTTATTATAGAGTAAGATTCCTCTTGATAATCGGCCACCTCCTATTGAATCCTATCCAC
TCATTTTTCAAATGCGCCACAACCTCTTGCCGATATTTGGAACATGGTCGGTGCTCCTTGTGGCCCATGAGAGCG
AGTCATGCCATGCCATGCCATCCATGCATGTCGATCTCTTTTCTTTATCCATCCATGATATAATATCCCTTTTTC
TTTTTCTCATGTCACTCAACTATCTAGTCACATGGTGGAATCATTGATGAGCTTCCCGACATTTCCATTAAAGCA
TGTTAGTGACAGCTAAGGGTACAGAGAAAAACCTGATTGGATATGGTCTTCATTTTCCATATATATATTCCTTTC
TATTTCCAAACAAATATATACTACCGACAATAACAATACAATATTATGAACTTAATATTATATTCACACACGTCG
GTCTCTACTCTCTAGTCTCTAGTACTATATATATCATGCGACCTAGAAAAATTGCATTTGTGCAAAAATATCTCT
AACAATATCCTTATCCTCTTAATCAATTAGCAGCACTACTAGCTTCTCAGGCC**ATG**GCCAGGATCGCCTTCGGAA
GATTCGATGATTCGTTCAGTGTGGGCTCTTTCAAGGCTTATCTTGCCGAGTTCATCTCAACCTTGCTCTTTGTTT
TTGCTGGTGTTGGTTCCGCTATAGCCTACAGTTACGTTCTCCATCCATCCCTGCCTCCTCCCATACACCACCCAC
TCGATGCATCATGCATATTCAATTTTTTTTTTTTAGTAACATTAATTGAAGTGTTTCACCCACTGGAATCCCTGG
CCGTACGTGCAAAAACTAGATAGTAATTAATTTTCACAAGGACTAATTACGTACTAATGTAAATTAATCATGTCA
GTAAGCTATATATGGCCGTACAAAATTGTTGTTTTTCCCTCCTTTACAATATTTATTCTAACATTTTATTATTGT
TTGTTTTGTGGGTGACAGATAAGTTAACATCGAATGCGGCACTTGATCCATCGGGGCTAGTAGGCATTGCAATTT
GCCATGGGTTTGCTCTGTTTGTTGCAGTCGCTGTTGGAGCCAACATCTCCGGTGGCCATGTTAACCCAGCTGTCA

CCTTTGGATTGGCACTCGGCGGCCAAATCACACTCCTCACTGGCATCTTTTACTGGATCGCCCAGCTTCTTGGCT
CCATTGCCGCATGCTTCCTCCTCAAGGCAGTCACAGGCTTGGTAATTGACGTTCTTCAAAATATATCAATAATAT
CTATGATTAACTTTTGTTTTTTTGGGTTAAAAACACTTGAGCCTTTTAAACTATTAATTATTTTTAAACTACAAT
TTTATTGTAATTAAGTTATTTTTTTGTCATTCTTATAAAATTATCTTTATTTTCTTAAAACAAAATTAATTAATT
AAAAACCAAAATAAAAAAATCAAAATTTGTTTGTTTAGGTTTTTTTTTTTTTTAATTTTAATTTTGATTTTTGA
TTTTTGATTTCAATTTTTTAATTAATTAATTTTTTTTAAGAAAATATAAGTATTTTTGAAATATTGACAAAAAAA
TGACTTAATTACAATAAAATTGTAATTTAATATATCTAAGTATCACAATTGTCGATTCATCGGACTTTGTGAAAA
TTACTTCTAGTTTAGAAAGTTTTAAATGTATTTAATCCCGGCCCTTTTGTTATTTTGGCACCCTTAACCTAGCTA
CTATTATGAACTATCTATCAAGGGAGTGTTAAGTACACAAAATCACTGAATTAATTAATTAATTAATTATGCTTT
TTCAGGCAATTCCCACCCATAGCGTTGCGTCAGGAGTGGGAGCCATTGAAGGAGTAGTGATGGAGATCATCATCA
CCTTTGCTTTGGTTTACACGGTGTATGCAACAGCAGCCGACCCCAAGAAGGGTTCGCTAGGCACAATAGCACCTA
TTGCCATTGGTTTCATTGTTGGTGCAAACATCCTGGCTGCCGGCCCATTCTCCGGTGGATCCATGAACCCAGCCC
GCTCCTTTGGGCCTGCAGTGGCCAGCGGTGACTTCCATGACAACTGGATCTACTGGGCTGGGCCCCTCGTTGGTG
GGGGCTTAGCCGGCCTTATATATGGCAATGTGTTCATGTACTCTCAACATGCACCTCTAACCAATGACTTC**TGA**T
CGATTTCCTTCAAATCCTTGTGTTCTCACTAGTCCACATGTGTTTCTATTAATAAAGGAGGAAAAGCAATTTTTG
CTTTTCTTTCCTTTCTTTTTCTTTTTCTTTCTTTTTTTTTTTTTTCTTTTTATTTTAGGTTGATCTTGTTTTGTCC
TTGCATGTAAAACCTTGTTGGGTTCAAATGTTGCTTTATGAAAATGGATGAAGTTGGTGATGTGCAGCTACGATC
ATTGTTATATATAAGTGTATGGTTTGCTCTTAACGTCTTGTATCTGTTTTGGAGGGGAGGATGGCCCATCTTTGC
AATAATGCAACTAACTCCTTTGGACACACGCAAATTATGGCTTCAACTTCTTTTCTTTTCTTTTTTTAATCTGAA
AGTAATTCTCTTACAACATTTTTTATTTTATTTCTCTATCTTATTTAATTTACTGCAATGGTAGTCTTTTGCGCA
ACATAATAGATAAGAAAAAGGAAAGAAAATTGGTATAGCTCAATTTAGTTGCGGCTTGTGGTGTTTGAGGAAGGA
GAAATGTTCTTTTTTTTTTTTCCTTTTTTTTTCTTTTTTGCATTTTTCCAGTATTTTTAACTAATATAGTATGCTC
AATCAATCCTTAGATCAAATCTTGATTTCCTTAGATCAATCTTAATTAAAAATGATAAAAATATGCAACGGTAGC
TAGATTGAGTCTTGAGAAAATGTTAGAGAAAGCAAAATATTAAATGTTCCATTTTCAGTTGATGTGGCATACATT
GCTAAAAACACGCATTAGCACCAAAATGTCAAACAAATTAATTTATATTTAGGGAAATGCTAGAATATTTACTCA
AATTTTTATCCCATCATTCTAAATATTACAATCACCATTAGATTTATGGATTTATGTAGATCTCATATGTGAGAC
CCGCCCATTCAATGGTGATGGTAATATTTAGAAGGATGAGAAAAGGATTTGGATAAATATCCTAACATTTCCCTT
>*Bpe*TIP2;1
ATGGCCAGGATCGCCTTCGGAAGATTCGATGATTCGTTCAGTGTGGGCTCTTTCAAGGCTTATCTTGCCGAGTTC
ATCTCAACCTTGCTCTTTGTTTTTGCTGGTGTTGGTTCCGCTATAGCCTACAATAAGTTAACATCGAATGCGGCA
CTTGATCCATCGGGGCTAGTAGGCATTGCAATTTGCCATGGGTTTGCTCTGTTTGTTGCAGTCGCTGTTGGAGCC
AACATCTCCGGTGGCCATGTTAACCCAGCTGTCACCTTTGGATTGGCACTCGGCGGCCAAATCACACTCCTCACT
GGCATCTTTTACTGGATCGCCCAGCTTCTTGGCTCCATTGCCGCATGCTTCCTCCTCAAGGCAGTCACAGGCTTG
GCAATTCCCACCCATAGCGTTGCGTCAGGAGTGGGAGCCATTGAAGGAGTAGTGATGGAGATCATCATCACCTTT
GCTTTGGTTTACACGGTGTATGCAACAGCAGCCGACCCCAAGAAGGGTTCGCTAGGCACAATAGCACCTATTGCC
ATTGGTTTCATTGTTGGTGCAAACATCCTGGCTGCCGGCCCATTCTCCGGTGGATCCATGAACCCAGCCCGCTCC
TTTGGGCCTGCAGTGGCCAGCGGTGACTTCCATGACAACTGGATCTACTGGGCTGGGCCCCTCGTTGGTGGGGGC
TTAGCCGGCCTTATATATGGCAATGTGTTCATGTACTCTCAACATGCACCTCTAACCAATGACTTC**TGA**
>*Bpe*TIP2;1
MARIAFGRFDDSFSVGSFKAYLAEFISTLLFVFAGVGSAIAYNKLTSNAALDPSGLVGIAICHGFALFVAVAVGA
NISGGHVNPAVTFGLALGGQITLLTGIFYWIAQLLGSIAACFLLKAVTGLAIPTHSVASGVGAIEGVVMEIIITF
ALVYTVYATAADPKKGSLGTIAPIAIGFIVGANILAAGPFSGGSMNPARSFGPAVASGDFHDNWIYWAGPLVGGG
LAGLIYGNVFMYSQHAPLTNDF*

*Bpe*TIP2;2

>**FXXK01000121.1**|:1235422-1242022 *Betula pendula* genome assembly, contig: Contig120, whole genome shotgun sequence
>**Bpev01.c0120.g0064.m0001**
*Bpe*_Chr14 4,812,457-4,815,151 (1) ::
join(4812457..4812583,4813368..4813579,4813642..4813664,4814779..4815151)

>*Bpe*TIP2;2
TTTTTTAAAAAAAAAAAAAAATTACTCTTATAATCCCCTTAAAAATTAAATTTTGAGCCCTTAAATTTTGTATTT
TTTTAGTTTACCCTCTAAACTTCAAATGCTAGTTTCACCCCAAATCGGACATAATGATTAATTGGCATCAGACAA
ACCTTAGTTAAATATTTTTCATATAAAAGAAACAAACGAGGCATCACACCAAATCCATTCCACAGTTCCACTAAT
CAACCAAGCCTTTATAACTAAATCGATTCCAATGATTATAGTTCTAAATAATTTTCTTGGAGAACTTTGAAGACC
GACCAAATTTTATTTTAATATTATCCTTAATCCATAGATTAATGAGGTTGCTGAAATCATACAGATATAACATTA
ATATTATCGTTAATTAATAGATAATTTGCAATTATTTCTAAACATAGTGGATCCATACATGCTTATCTGTCCCTA
CAAGACCATGCTTATCTCCCAGATGTTTTAAAATTGTCGTTGTCTGTCCATTAATTAGTAGTTAAGTTGGTTGTC
TTACGCTCATAATGCCTATAGTTTATCAAAACAAGGCGGTGGGTAGGCACCAAAAGCCAATCATACAACGCCCAT
TTATTATTCATGGTCGGTGCCGGCCATGGCCCTTTCTTCATATATAGCCTCTCGATCTGTATGTAGCACTAAGCC

ACTAACTATATAAACCATGCATGCACATTTCTAAGGCCTATCTAATTAAGCAGATCTCTTTAACGACGACTCTTG
AGCTGATCCGTCGTCTTTCTTTTTCTAGTCAGCGAAA**ATG**GTGAAGTTGGCTTTTGGTCATTTTGGAGAGTCTTT
CAACGTTGAGTCTCTCAAGGCTTATTTGGCTGAGTTTATTGCCACCCTTCTTTTCGTCTTTGCAGGCGTCGGTTC
CGCCATTTCTTACGGTACGTAAGTGCGTCAAACATGATTCAACATATCGCAACGACAATCACATATTGCACGGAC
AGAAATTTTCTCTAAGTTTGGTTAATTGAAAGTTTTTTTTTTTGTTAATTTAATTTAGTATATAATACTGTCAA
GTGTTTATTGTGTTAAAGTATTGATTAAATGATTAAATTTATCTGTTTCTATCAGCTTAAGCTTTTGAATAATC
GGTGATTTAACATAGTATCAGAACTAAAGGTTCTGAGTTCAAATATTGATTCTGTTATTTACTTCTCATTTCAAT
TAAGTATTATGAGTGTTATAGTATTGATTAAATAATTAAATGATTAAATTTATTTAATTTTAGATCAAACTATAA
TTTTAAAAGCCATGTAAGAGCGTGGGAGTAGGTGTAGCATTTCTTTTTTTCTTTTCTTTTTTTATTTAAATGGCT
GTAATAAGAAGATATTTAAAACACGTTACGTCAACTGGTGTGATAAAAATGTATGAAAAGTAGCATTACTAATTA
TAAATAATGTGTTTCTCACATGCTAAGGAAACATAATAAATTTATAGACTGAATTAAAGAAAATTTCTCCTATTC
AATTTAGAGAAAATTTGTGTGGTGACATGCACCTAATAAAACACGTAAAAATTTGTATTTATATGGAGAATTTAC
TAAAAATAAAAAAAGTCAAAACAAATGGTAAAAAAAAAAAATTATTTACAAAAAGAACGAATTCATAGCTAGAAA
TATTGTTTTTGTTGAGAATGATCACTCGGTTATCTTCGATCTGTACAGGTAAGCTCACGTCAGATGCAGCCTTGG
ACCCTGCAGGCCTTGTAGCGGTGGCGGTGGCCCATGCCTTTGCACTTTTTGTGGGGGTGTCCATTGCGGCCAACA
TCTCCGGTGGCCATTTGAATCCAGCTGTCACATTTGGATTGGCACTGGGAGGCAACATAACCATCCTAACTGGCA
TTTTCTATTGGATAGCCCAATGCCTTGGCTCCATTGTTGCTTGCCTCCTCCTCCAATTCGTCACTAATGGCCTGG
TACATTTCTTGGGAAAATTAGAATATTTTATTACAAATAATTTGGTCATAAAATTTAATGAATAGATTGGTGTAT
TTGTTGGCACTATTAAAAAAAAAAAAAAAAAAAAAAAAAGAGAGCGATTTTTGGAAAATAATCTCTTTCTTGAAAA
GTAGGCTCTAATTGTTTGAGCATAAGGTTATGCATTTTAGCTAATCAAAAATCATTATTTTTTTCTTTACGTATT
AGTTTTTTCAATAAAAACTCTAACAGATTTTTTATTTTATTATTTATTTCTTTAAATATTATTTTCTAATATATA
TATATATATATTTTATTTTCTTAAATCATTAAGAGATAGGAAAAAGAAATGATTAAAAAAAGAAATACATAAGCG
GATTGGTGCTTACACAGTTACACTCCAATTAAAAAAAAAAAAAACACTGAACCAACAACTACCAACGATAGAAGAA
ACAATCACACAACCAGCTACAAGATCAGCATCAGACAATCTGCATAAACAAAACTACAACTAACACCTATACAAG
CCAAGTCAAAACACATCACAGAAGAAATAAACACCAGGAGAAAGATTAACCCAAGATCTGTTGTAAGAGGTTCCA
CATTTTACTTAGCTCCGGATTCTCAGCAGTCAACTTAAGCCATAATGCTTACTTAAAAGTAGCTAATCAAAAGCA
AAATATGTATTTTAGATAAAGGAGAAGACAAGGTCATGGATTTGATTTTTGCCTCATATTAGCTAAATATACACA
ATATAAAAGTTTTAAACAAATGCTCTTAAAACTCTTTATTTTCTGTGCTCAAAATAAAAACATTAAAAAACAATA
CAAAAACAACATGCTCCTCAAAACACACAAACATTTTTCATTTTTATGTCACATAAGGACACTTTTTCAAAAAAA
AAAAAAAAAAAAAAAAAAAACCCTCCACCAAAATACATTAACAAATAGAGTGATAATTTTTGTCAAATAATAGAG
ATTAAAAAAAATATCTTTGAGGTTTCCACCTAAAATTTATTCTTTGAAAGCCAAAATAGAAGAAATTGACGCTTA
ATGTGTATATATATATATATATCGATGCTTATACAACAAATCTTGGTGTTTGTTAAGTTACAAATTATGGTAACC
CAGGTTTTTTACTTGACCTTGAATTTGGCAGAGCATCCCAACACACGGAGTTGCTTCAGGAATGAATGCAGTTGA
AGGCGTGGTGATGGAGATTGTCATAACCTTTGCACTTGTCTACACTGTCTATGCCACTGCTGCTGACCCCAAAAA
GGGCTCACTCGGCATAATTGCACCTATTGCAATTGGGTTCATCGTCGGAGCAAACATCTTGGCTGCTGGTCCATT
TAGTGGCGGGTCGATGAACCCGGCCCGCTCATTCGGTCCGGCCGTTGTTAGCGGAGACTTCTCCCAAAATTGGAT
CTACTGGGTTGGTCCCCTTATTGGGGGAGGGTTGGCTGGGCTGGTATATGGAGGCATTTTTATTGGCTCCTATGA
GCCTCTGCCACCCTCTCAAGACTATGCC**TAA**AGTAAAGAGACTTCTTCTGGGATTTGGGTTTCTTCTATCTTTG
GGTTTCATATTGTGCTTCTCCATGTCCAGCCCAAAAAGATATTGTACGTGTTAATAAAATTGGAAATGATGAATC
AAATTGGCTTTCTGTTGATGTCATTTTTTTTTCTTAAAAAAAATTGTTAAATATTGTGCTTCTCCATGTCCAGCC
CAAAAAGATATTGTACGTGTTAATAAAATTGGAAATGATGAATCAAATTGGCTTTCTGTTGATGTCATTTTTTTT
TATGAAAAAAAATTGTTAAATATTCCTGTCTTAAAATTGGGCAATGGGCCCAATTTTTTCTTTTTCTTTTCTTTT
TTTTTTTAAAAAAAATATATATATACACTGAAATAGCAAGGCCTTTCACATGAATGCCAAAACAAATTAAAATCT
AGAAACAAGTGAAAACCATGAATGTCCATCTTCCAAGTTTCAAGTAGGGTGACTGTTGGTTAGAGCTAGCAATTT
TTTATATAATTCGTGAATTCGAAATGAATCTAATATGAAATTAGTGTGTTTAGGTTGATGAGTCTGACCCGTTTA
ATTAAATAAGTCGGGTTATGATTAACTTATATAATCTTATATCTAGAGGTGTAAAATGAGCTCAACAACTAGTGA
ACTTAACTCAGTTAAGTTTTTGACGGAATTTGACAGCATGGTGTAATTTGCTATTTATGCATAACCTTGGAAG

>*Bpe*TIP2;2

ATGGTGAAGTTGGCTTTTGGTCATTTTGGAGAGTCTTTCAACGTTGAGTCTCTCAAGGCTTATTTGGCTGAGTTT
ATTGCCACCCTTCTTTTCGTCTTTGCAGGCGTCGGTTCCGCCATTTCTTACGGTAAGCTCACGTCAGATGCAGCC
TTGGACCCTGCAGGCCTTGTAGCGGTGGCGGTGGCCCATGCCTTTGCACTTTTTGTGGGGGTGTCCATTGCGGCC
AACATCTCCGGTGGCCATTTGAATCCAGCTGTCACATTTGGATTGGCACTGGGAGGCAACATAACCATCCTAACT
GGCATTTTCTATTGGATAGCCCAATGCCTTGGCTCCATTGTTGCTTGCCTCCTCCTCCAATTCGTCACTAATGGC
CTGAGCATCCCAACACACGGAGTTGCTTCAGGAATGAATGCAGTTGAAGGCGTGGTGATGGAGATTGTCATAACC
TTTGCACTTGTCTACACTGTCTATGCCACTGCTGCTGACCCCAAAAAGGGCTCACTCGGCATAATTGCACCTATT
GCAATTGGGTTCATCGTCGGAGCAAACATCTTGGCTGCTGGTCCATTTAGTGGCGGGTCGATGAACCCGGCCCGC
TCATTCGGTCCGGCCGTTGTTAGCGGAGACTTCTCCCAAAATTGGATCTACTGGGTTGGTCCCCTTATTGGGGGA
GGGTTGGCTGGGCTGGTATATGGAGGCATTTTTATTGGCTCCTATGAGCCTCTGCCACCCTCTCAAGACTATGCC
TAA

>*Bpe*TIP2;2

MVKLAFGHFGESFNVESLKAYLAEFIATLLFVFAGVGSAISYGKLTSDAALDPAGLVAVAVAHAFALFVGVSIAA
NISGGHLNPAVTFGLALGGNITILTGIFYWIAQCLGSIVACLLLQFVTNGLSIPTHGVASGMNAVEGVVMEIVIT

FALVYTVYATAADPKKGSLGIIAPIAIGFIVGANILAAGPFSGGSMNPARSFGPAVVSGDFSQNWIYWVGPLIGG
GLAGLVYGGIFIGSYEPLPPSQDYA*

*Bpe*TIP3;1

>**FXXK01001027.1**|:34394-36563 *Betula pendula* genome assembly, contig: Contig1026, whole genome shotgun sequence
>**Bpev01.c1026.g0003.m0001**
Bpe_Chr7 20,005,317-20,006,689 (-1) ::
complement(join(20005317..20005703,20006202..20006452,20006560..20006689))

>*Bpe*TIP3;1
AAATCCTAGAAGCACACTACATAGCTCCCCAAGCATTTTAAATTCGAGAGAGGGGTAGATGGGAAGCTAGAGACC
TTCGAGGCAAGTTTAGTTGCAAAAAGTTTTACTTAGAAAGAAGAGATTGACGATTAGAAAACCTTTTCATCAGTA
GTCATGCTTAAGTCTATTAAGATACTTTTAGCCCGTTGCCGCTCATCTAAATTTTGAGATTTGGCAAATGAATGT
CAAAACATCCTTTTTAAATGGGTGACTTAACGGGGATATTTCTATGATGCAGCCAGATTATTGTTTTGTAGCTGA
GAGCCAAGAAAGTATGGTATGCAAATTGCAAATATCCTTTTATGGACTTAAGCAAACATTTAGATCATTGAACAT
TAGTTTTGATAAGACAATTAAACAGTTAAGTTTTGAACAAAATCTCAATGAACCATGTGTGTACAAGAAGTGTCA
AGACAGAGTAGCAACATACTTGGTGATGTATATTGATGATATCCTAGTCATTGGGAATGGTGTATGGACATTATC
AACAACAAGGATCTGGTTGTCTAATCAGTTTGATATGAAAAACTTGAGAGAAGCAAGTTATATCCTAAAGATCAA
GCTCTTGTGAGATCGCAAAAATATGATGATAGACTTATCCTAAGATAGATACATAAATAATATCTTGTCTGGATT
TAGCATGCTATACTCCAAGAAGGGATTAATACCCTTTAGGCATGAAATACCTCTTACTAAAGAGTAGTGTCCTAA
GACACCTCAAAAGGAAGAACAAATGATGACTGTTCCTTATGCTTCGATTGTAGGAAGCCTCATGTATGCTATGTT
ATATACTAGATTATGTAAAATGTAATATAATGATTCATAAGTATCTAATAATAATATTAATTAGAGCATATAATG
GTTAAATATTAAGCAACCTATATAAATATAATATATATTACGTAATTACTAATATACATACTTTGATACTCATAT
ATATATATATAAAACCCATTTTTAATAATATATATACCAAGCTAACGAGTTGAGTCGGATGAGCTAGTCAGGTTG
AGGTAGTCAGGTTAAGCTTTATACGAGCTAGGCTTGCGAGCCTTAAACAAGCTGAGCGAACGGCCGGATCGAGCT
TTAAACGAGTTAAGCTCACAAGCCTAGATATGTATTACTTACTATTTGAGCGTGCGGATTTCTACAGTAATAAAT
GAGTTTGTACAATATCCTATTCAAATTCGGATTGGACTTCAAGTGAGTTGTGGAACGGACTTTTTTATTTAAGCC
CTATGTATTGAGAAGGCAATGTGCCGAAACCGAACCAAAAGCTTCCTAGCTAGTAAAGCATGTATTAATTGGTGC
TGGACCTAGATTTTAGGTAGCGTGGGGGGTTAACCATATCTTCAGTCGAGGATGAAGTTAACCTTGTGTCCAAAC
GCATCCACTTCTGGTGATGATAGAGTTGGGTACGCATCGACTTCTAGTGATGATAAGAGTTGGGTGTACGGAGAA
TACTTTCAAAAGAAGAAATAGATAGACACTGTTCCAGCTGGAATTGGAATCAATAGTTAACACATCTACGTGTCA
GCCTTGGCGCACGAGGTGTCGCCAAGTGTCCGTATAACTCCATCCCCCATTTGGCCGATTTATTCAATAACCTCC
ACCTTTCAGCCATGCAAACACTCTCATCCACCTTGCTTTCCTAATATCATATCCTTCTCGTCACCATTTTCATCT
CCTAAACAACCACACTAACAAACTGGATATTGCTTCGAATTAAAGAACTAAAAAGGTTTCAGAAACTGCAAAAAA
GAAAAGAAAAAAACAGGGCTATTAATCTCTTGCCACC**ATG**CCCCGGAGATATGCGTTTGGGAGGGCTGAAGATGC
CACCCACCCCGACTCCATGAGAGCCGCCTTATCAGAATTCATCGCCACTTTCATCTTTGTCTTTGCCGGCGAAGG
CTCTTTACTAGCTCTCGGTTTGTCCATCGCTCTCTACAACATGCAATCCTATATTTAATTTATGGTATTAATTTG
TAGTTGGTGATGGTGATGGTGATGGTGCATGTGATTTCTGTTTTGGCAGGGAAGATCTACAAGGATACAGGCAGT
ACTCCGTCGGAGCTAGTGGCCATCGCACTCGCGCATGCATTCTCCCTCTTTGCAGCAGTGGCAGCCAGCCTCAAT
ATATCCGGCGGTCATGTCAATCCTGCCGTCACCTTTGGCACCCTACTCGGCGGAAGGATCTCCGTTCTCCGTGCC
GTCTTCTACTGGATTGCCCAGCTTTTGGGTGCTGTTGTGGCTTCACTTCTGCTCAGGCTTGTCACCAATAACATG
GTACAACATGCCCACTTAATTTTTTTTTTTTTAATAACAGAGTATCTTCGAGACTTCCACACGGGTCAGGTAAAG
CTCGAAGCTAAGTAATTTCAAAAAATTGTTTGTACTTAAAAGATTAAACCTTTGACATCATAGGCGAAACCAACC
CTTGGCTTGGGTCCCTAGCCACCCAAAATTTTAAAAAACATCATAAAAAATTTGAAAATTTCTTCTAAATTTAGT
GTATTTTTATGAAATAAACCCCAAAAATAATTTTTACGTCCAATCACTTTTTTTTTTTGATTTTGTCACTCCTCT
CCTAAACTCCTAGTTAAAATCTTAGTTCCACTCCTTTTTGACATGATGTTTCTCACGAGTCACCAGAATAAAGTG
ACTTATCACTCGAGTTAACCTTAGGCCGGGTTGCAATGTTCCGAATTAATTTGGTTCAAAAAAACACTTTGGTGA
ATTTAATGTTATTATAGCATGTTATGATGTTTATGGTGTTGGTTTCAGAGACCAGTGGGGTTCTACGTGACGTCA
GGCGTTGGGGAGTGGCACGGACTTATATTTGAGATCGTGATGACATTCACGCTGGTCTACACGGTTTTTGCGACC
GCCATTGATCCCAAACGGGGTAGCTTGGGGACGATTGCGCCGTTGGCAATCGGTTTCATCGTCGGAGCGAATATT
CTGGCGGGTGGGCCGTTTGACGGAGCATGCATGAACCCTGCCAGGGCTTTTGGGCCGGCTTTGGTCGGGTGGAGA
TGGAGGAACCACTGGATCTACTGGGTCGGCCCACTTTTAGGAGGTGGGCTTGCAGGCCTAGTATATGAGTACATG
GTGATACCCACGGAGGTTCCTCATCACACCCACCAGCCCTTGGCTCCAGAAGATTAC**TAG**TTCTTCTCATCCCTG
TTTGTTTGTAATAAAATTGCAAGTGGTTTGGCCTTTGCTTTTGTTTGCTTTCTTTTTTAAATATGTTTTGGGTTC
CTAGCTTCTGAGTTTGTTCATTGTCTGCAAGTGTTGTACCTAGTTACTTGTTAATAAAATGACAACCTAGTTGTT
CATTGTCTCCCATGTAATTTCTTTTTACCCTTTCCAATGTTTTGCTTAGAGAAACAAACTGTTAATTTTACGTGA
GGTTAAATATACAAAGGCCATTTGGGTGTAGTAAATAAATGTGGTTTTCTTCCGTCATGAAATGATTGTCACCCC
AATCCGTCCATCAGACAGTATGGTGACTTGACATCATCTAAGTTTTCATCCCTGCTGCTGTATTACCATTAGACA
GAGTGATCTCCACCCGTGACCAAACACAATCTTCTAGCCCACTAGACTAGCTATGTTCAGATATTGAAAACTCAA

>*Bpe*TIP3;1
ATGCCCCGGAGATATGCGTTTGGGAGGGCTGAAGATGCCACCCACCCCGACTCCATGAGAGCCGCCTTATCAGAA
TTCATCGCCACTTTCATCTTTGTCTTTGCCGGCGAAGGCTCTTTACTAGCTCTCATCTACAAGGATACAGGCAGT
ACTCCGTCGGAGCTAGTGGCCATCGCACTCGCGCATGCATTCTCCCTCTTTGCAGCAGTGGCAGCCAGCCTCAAT
ATATCCGGCGGTCATGTCAATCCTGCCGTCACCTTTGGCACCCTACTCGGCGGAAGGATCTCCGTTCTCCGTGCC
GTCTTCTACTGGATTGCCCAGCTTTTGGGTGCTGTTGTGGCTTCACTTCTGCTCAGGCTTGTCACCAATAACATG
AGACCAGTGGGGTTCTACGTGACGTCAGGCGTTGGGGAGTGGCACGGACTTATATTTGAGATCGTGATGACATTC
ACGCTGGTCTACACGGTTTTTGCGACCGCCATTGATCCCAAACGGGGTAGCTTGGGGACGATTGCGCCGTTGGCA
ATCGGTTTCATCGTCGGAGCGAATATTCTGGCGGGTGGGCCGTTTGACGGAGCATGCATGAACCCTGCCAGGGCT
TTTGGGCCGGCTTTGGTCGGGTGGAGATGGAGGAACCACTGGATCTACTGGGTCGGCCCACTTTTAGGAGGTGGG
CTTGCAGGCCTAGTATATGAGTACATGGTGATACCCACGGAGGTTCCTCATCACACCCACCAGCCCTTGGCTCCA
GAAGATTAC**TAG**
>*Bpe*TIP3;1
MPRRYAFGRAEDATHPDSMRAALSEFIATFIFVFAGEGSLLALIYKDTGSTPSELVAIALAHAFSLFAAVAASLN
ISGGHVNPAVTFGTLLGGRISVLRAVFYWIAQLLGAVVASLLLRLVTNNMRPVGFYVTSGVGEWHGLIFEIVMTF
TLVYTVFATAIDPKRGSLGTIAPLAIGFIVGANILAGGPFDGACMNPARAFGPALVGWRWRNHWIYWVGPLLGGG
LAGLVYEYMVIPTEVPHHTHQPLAPEDY*

*Bpe*TIP4;1

>**FXXK01000922.1:**34590-41021 *Betula pendula* genome assembly, contig: Contig921, whole genome shotgun sequence
>**Bpev01.c0921.g0003.m0001**
Chr Bpe_Chr9 15,031,065-15,032,014 (-1) ::
complement(join(15031065..15031442,15031546..15031784,15031888..15032014))

>*Bpe*TIP4;1
CATTCTCCCCCTAGCCAATACTGTGCCCATTTGCTTTTTTTTTTTTTGTGTTTTTTTCTTGACTTAAGCATTGGA
GGTGGTTTTTAGTGGTCCAGCTTTTCTGCTCTTTTGTCTTTCTTGCAAATATTTTGGACGGAGAGTTTGATGTGT
TATCACTGTATCAACAATAATGAATCAAAAGACAAAACGACCTTAACCAGTCAAAAATATCTTCTCCACCACTTC
TTTGTCAATGACAAAGAAGTTCTCCAATTTTAAAGTAAATATCTAGATATTAATGTAAACTTCATATTTAGATAT
TAATGTAAACTTCATATCTAGATATTAATGTAAACTTCAATGACAAAGAAGTTTTCAATCAAATATGAAGTTCTC
CAATTTTCTTGAATCCTCAACAACCATATTTTCAGCCAAAAAAAACAGTAAAGAAAACAAAATATTATTTTCCTT
TCTGAGCGAAGAAAAAAAAAAACAGAAGACTATTGCATTTCTATTTTTTTAAAGAAAACAAATGACCAAAGTCCTT
CTCATCCCAAAATTATTTCCGAACGACGTCATTGGCTACTGACGTAGTCTTCGTTTACTACAAAAATAAGCATTT
TATCAGTACTTATTTGCCGGCGCTTATGTTAAAATACCGACATTTGTCTTATTACCGATACTTGTTTTCTGGCAC
ATCTTGCACAAGTGCCTGGAAAGATGTGTCGGTAAAAGTAATTTTTTTTGTAGTCTTTCAGTTTAGTGGAAACTC
ACAGATCGGGGAAATTAAGAGTTGAAAGAAAAATTATGATTTATTAGTTACTTCTCTAAGGTTTAATTTAGAATT
AAAGTGAAGCCCTGAGAATTTTTACTCCAAAATTAAAATTTTTCATGAAATTTTGGGTTTTCCAATACTCATTGC
TCGTATATTTGCATGGATGGACCAACTAAGTGCGGCTAAATTGGTTAGAATATTTCCTAGCTCCGTGGAGAACTA
AGCAATATATGAATAGGTAATTAGTTTTTGGCCATATCTTTTGACCATAAATTAAGCTTACAAACGTTGCTAGCT
AGTCCATGGGCATTGGCGTAATTTAATTAGTCAACAATTATTTTATTGCATGATCGATAAGATGCGATCACATGC
AGTGCGTCATCATCCAAAAAACAAATGCCATTATTACTAGTAAAAATAATAGTGCAACCTAGTAGTGCCGATATT
ATATATAGCATATGCCAACGCAGCAGCAAAGGCAACCCAAAGTTGAAGCTTTTTAGCAACTAATTAAGTTTAAGG
AGTAGTACTCTCTTTACTCTTTAGGCGTTGCCTTCTAAAAGGTACATATTTTTTGGTC**ATG**GCGAAAATTGCCTT
GGGAACCACCCGAGAGGCCACACAGCCTGACTGCATCAAAGCCCTTGTCGTCGAGTTCATTACAACCTTCCTTTT
TGTATTTGCTGGAGTCGGATCCGCCATGGCAGCCGGTAATTATTCATTTTACTTTTTAATTAATTACTTTCGTTG
AAACTATATATATATATATTTTTTATTATAACCAAATCGTGTGCATGCATGGCTTTGATGAACAGATAAGCTAGATT
CAGGTTCGCTGGTGGGGTTGTTTGCTGTGGCTATCACACATGCACTTGTTGTGGCGGTGATGATATCCGCCGGCC
ACATATCCGGCGGTCATCTTAACCCGGCAGTCACGCTTGGCCTCCTCGCCGGCGGCCACATCACCATCTTCCGCT
CCTTTCTTTATTGGATCGATCAATTATTAGCAGCTTCAGCCGCTTGTATTATTCTTTACTACATTACTGGCGGAT
TGGTGAGTCAAATGGCAATGGCTCTTTTTTTTTGTTTTTAAATTGTATTGATAATAAAAAACTTACTTTGTGTTT
TTTTTTTTTGGTTTTGTTTTGTGGATGCAGACTACTCCAATCCATTCACTTGCAAGTGGGGTCGGGTACTTGCAA
GGGGTAATATGGGAGGTTATTCTGACCTTCTCCTTGTTGTTCACTGTGTATGCTACCATAGTGGACCCCAAGAAG
GGGGCTCTTGATGGATTGGGCCCAACTCTTACTGGGTTTGTTGTGGGGGCCAACATCTTAGCTGGTGGGGCTTTC
TCAGGGGCCTCAATGAACCCGGCACGGTCCTTTGGACCCGCTTTGATAAGCGGGGACTGGACTGATCACTGGGTT
TACTGGGTCGGCCCACTCATTGGTGGTGGCCTTGCTGGTTTTATCTATGAAAATTTCTTCATCGTCAGATCTCAT
GTCCCTATTCCTACTGAGGAAGAACGTTAC**TAA**TTGTCCCACTCTCTCTAAAGTTTATTGAGCCTTTCTCTAAGT
ATTACTTTTTTTTGTAAGTTTTCTTTATGTATTCTAGTGCTGCCTTTTCTTGTTTCCCATTTTCACCTATGCATG
GTATTATTATCTATCGGGAGCAAATGCTTCTATGAGATTCTATTGTGCAACAGCAAGAGTTCTTAATTGGATTAT
CTGGGAAAAAGAATTAACATTACTTTATTTTCTTCTAGATGACTTTGCGAGTGCTTAAACGTGTCAGGTCTCGTT
ATTTAATGTTAAGGTCAACATCAATGGCAAATGCACACTTGAATTTTTATGTCTTTTTTTGGTACAAATCAAATA

GGCAAACCAACTCACAAACACTAACAAAATAATAATAATAAAAAAAATCCATAACCCAAAAGAAGACACTAAAAT
TTTTACCTGCAAAACCTTTCAATGCAAAGGGAAAAATTACAGGACATAGTTCAGTTAAATCTTCTACAATGAACA
ACAAAGGGCTTACAAATATCGTCCCCTAGAAGATTTTTTAGAGGCGCTACCAACATATCAAAAATAGATATATTC
TTGGGTTACAACATCAATTCATCAGCTTAAGGTGGATTCCAATAAGAACAACAAAGAAAAATCTCAAAAAGTGGG
>***Bpe*TIP4;1**
ATGGCGAAAATTGCCTTGGGAACCACCCGAGAGGCCACACAGCCTGACTGCATCAAAGCCCTTGTCGTCGAGTTC
ATTACAACCTTCCTTTTTGTATTTGCTGGAGTCGGATCCGCCATGGCAGCCGATAAGCTAGATTCAGGTTCGCTG
GTGGGGTTGTTTGCTGTGGCTATCACACATGCACTTGTTGTGGCGGTGATGATATCCGCCGGCCACATATCCGGC
GGTCATCTTAACCCGGCAGTCACGCTTGGCCTCCTCGCCGGCGGCCACATCACCATCTTCCGCTCCTTTCTTTAT
TGGATCGATCAATTATTAGCAGCTTCAGCCGCTTGTATTATTCTTTACTACATTACTGGCGGATTGACTACTCCA
ATCCATTCACTTGCAAGTGGGGTCGGGTACTTGCAAGGGGTAATATGGGAGGTTATTCTGACCTTCTCCTTGTTG
TTCACTGTGTATGCTACCATAGTGGACCCCAAGAAGGGGGCTCTTGATGGATTGGGCCCAACTCTTACTGGGTTT
GTTGTGGGGGCCAACATCTTAGCTGGTGGGGCTTTCTCAGGGGCCTCAATGAACCCGGCACGGTCCTTTGGACCC
GCTTTGATAAGCGGGGACTGGACTGATCACTGGGTTTACTGGGTCGGCCCACTCATTGGTGGTGGCCTTGCTGGT
TTTATCTATGAAAATTTCTTCATCGTCAGATCTCATGTCCCTATTCCTACTGAGGAAGAACGTTAC**TAA**
>*Bpe*TIP4;1
MAKIALGTTREATQPDCIKALVVEFITTFLFVFAGVGSAMAADKLDSGSLVGLFAVAITHALVVAVMISAGHISG
GHLNPAVTLGLLAGGHITIFRSFLYWIDQLLAASAACIILYYITGGLTTPIHSLASGVGYLQGVIWEVILTFSLL
FTVYATIVDPKKGALDGLGPTLTGFVVGANILAGGAFSGASMNPARSFGPALISGDWTDHWVYWVGPLIGGGLAG
FIYENFFIVRSHVPIPTEEERY*

***Bpe*TIP5;1**

>**FXXK01000478.1:**207000-215500 *Betula pendula* genome assembly, contig: Contig477, whole genome shotgun sequence
>**Bpev01.c0477.g0022.m0001**
Contig477 209,208-211,230 (1) ::
join(209208..209337,209424..209674,210865..211230)

>*Bpe*TIP5;1
ACACATTCCACACAAAGAGGCATCTCGAGAGATAGAATAACAAGCATATAAGATCTATGTGCAATTTCAACAGAA
CATTGACTAGAAATTAACGCAAAAAATAAGGTCAATTAAATATACTAAACTGTGAAAATAAAACAAAATAAAAGA
TTCAAAGAAAACAAGTTACACCTACAACCAACTGTTGGGGATTCCACCTTAGAGAATTCCATAGACATCCACCTC
TGCTTGGTTGCAGAGAAACTCGAAGACAGTGAAAGAAAATAAAAATATTAAGCGCTAAGTTTGTCACATTTTGCT
TTCATGGTTACCTGTGGGCTGTGGGCCTGTGGCTAGAACTTTGTCTGAGATCTGCAGTCGGCTTGTTTGGCCGTC
GATTCGCTTGATCTCTCAGACTCCGTTAGCCTGATAATCATTGATACGGAACTGAGGCCTAAACGGAGCACCAAA
ATGGTGAGAAAGGGACTAGTTCTTTTTTTTTTTTCTCGAGAAAGGGACTAGTTTACGCGAAGTTGATCCCTTTTC
TTTTCTTTTTTGCTTTTTTTTTTTGGTTAGCCTCCGCGTGTTTCGACGTATTAATGTCAAAATATTTTTGATAGA
AAATAATTTTAGAAAAAAATATTAATTTTGTAATGTTTGCTGATACTTTGAAAATGATTATAAGGTTTTAAGGAT
GGTTTTACATTGAAAATTATTAATATTTTCATCTGAACTTATATAATTAATAATTTTATTCATAATAAAATATAA
TAAAAATTATAAGATTCCATTTAATAATTTAATAAATGGATTTTTTTTGAAAAATAATAAAACTTTAAAAATATC
CACATACATATATATTAATGTTTTTTTTTTTTTTGTAAGTGTATAATATTCAATTTAAGTCAACATTTTTTTCTC
TTATTACCATAAAATTTTCTTTACTTTTTACTTACAAAATATTTAAATCTACTTTGATTTTTATATCATATCTAC
CGCACGTGACACATTTTTATTTGAGTCTACGCAAAAAAAAATGGAACAATTAACACTTCTACTATTAAAAGATGAC
TAGCAATTAGGATAAAATGACCTATTTGTCAAATTGCCAAAACTCAAAGTTATAATTGTCAAAATTAAAAACTCA
AACTCAAATTTTATTTATCTCAGAATGCAAAACTTAAAGAGATTTTAGAGCAATTTTCCTTTCTTTTTTTTATAAT
AAAGAAGATGGTAAAAAAGTGAGAAATTATATTTGACAGGGTGGGCTCGGTCGGTGGGCCGAACCCGGACGATTG
TGCAAAGAGAAATACAACCGTATAAACCACACCCCGCGACGTGGCATAGCGGTTAATGTTTGAAATCCAAAGCCC
TGCTGAAACGTTGCAACCGAACCCCTCCTTCGAAGTTTGGAAACTGCCCCAACTACGCATTATATATCAACAACT
TCCATTCTCTCACTAGAGTATATAGCGAGAAAATTTTTAAATAAACCACAAAATCCGATCAAATTGTTTGATAGT
CTTTCACACTCTGAAATCAAGTAATTCCTCGTTCTTATTGGCAGAAAA**ATG**GGTCCAACAATGGTAGCCGCCCGT
TTCCAACATTCTGTCACTCCTAATGCGCTTCGATCATATCTCGCGGAGTTTCTCTCCACATTCTTCTACGTCTTT
GCGGTTGTGGGTTCTGCCATGTCATCGCGTGAGTTTTTAATTTTCTTTGGAGAATTTTTGCATACCATTTTTGTT
TGTTTTATTAAGTGGTCGTTGGAGTTTGCGATTTGGTAGGGAAATTGATGCCAGATGTTGCGTCGGATGCATCCA
GTTTGGTCGTGGTTGCCATCACCAATGCCTTTGCATTGTCGGCAGCCGTGTATGTTGCAGCGAACATCTCCGGTG
GGCATGTGAATCCGGCTGTCACATTTGGAATGGCGGTCGGAGGCCACATTAGTGTCCCAACCGCTTTGTTTTACT
GGCTTTCGCAGATGCTGGCCTCTGTTATGGCTAGCCTTCTCTTGAAGATGACCGTTGTTGGACAGGTAAAAGAAA
ACCTTCACATTCCTAGTCGTCTTATATTAATATATTTGACAACATCAAGAGCACCTTTAAACTAGACAAAGAAAA
AAAAAAAACATCTCTAGATAAATTCACTTTTTGGCTTCCGTTGTACCCCAGTTTCATGAAAAACATCATCAGTAG
TAAGCAGCCATGCATGCAGGTAGGGTAGGTGAGCAATTTTTTATACAACTTGCAAACATGACACGAAAATAATAT
GAAATTAATGGGTTAGAGTCGAAGTGTTTAACCTGTTTAATTAAATAAGTTAAGTTATGATTAACATATATAGTC
TTATGTAATCCCAAGGGGTTGGGTCAAGTGGTAAGGGTCTTGATTTTTGTGGTAGTCTCATGAGGTGTAAGGTTC

AAATCCTCTTGGGTGCAAACAATTTCTTGGGGCCAGCCCGCCGGCAAAGCCAGAGTATTATCCGATCTGTATGGA
GGGGGCGCTTTACACAGGTCTGAAGTTTACCTGACAGGGGGGTTGGTTTACGAAGTGACCTGCTTTGGAGGGTTG
GAGTATTACCCGATTTGTGTGGAGGGGCACTTTACACAGGTCCGAAGTTTACCTGACAGGGGTTGGTTTACGAAG
TGACCTGCTTTTGAGGGGTTCTTCGTTATAAAAAAAAAAAAATATAGTCTTATATCCATGTTTCGATACAAGCCG
AACTCGACACACAATATATAGGACAAACAATTTTTTACACAATTTGTGAACCCTACAAAAAATTAACAGGTTAGA
GTTGATAGGTTTGAACCGTTTAATTAAATGAGTTAAATTATAGTTAATCTTTATAGTCTTTTACTTATGGACGAA
ACTAGAAAGTTAAGTTTTAGAAGACTAGATTACATGAAATTTTTTTTTGAAGGGCCAAAATAAAAAATATGACCA
AAAATGTGTATTTATTAACAAATTTAAAGCTTAAAAAAGACAAATTTAACAAAAAAAATTTGCTAGAGGGGTTTG
GAGGGACCCATGCTCCCCTCTGTTCCCCCTCTAATTTTTTCACTACTTTATTTACCTATGTCTCGTTACGACCTA
AACCTGCATGCTTTATGAAACTAGAAATTTTTATATTGTGTAGTGGATCAATTGAATAGAGCTGTTGATTGGCAT
AGAAATATTTATAAATCGTGAGAAGTTATTCTAAAATTATATTTGAGTTCTGCAGCATGTTCCAACCTACGTTAT
TGCAGAGGAAATGACTGGGTTTGGAGCATCCCTGATGGAAGGTGTCCTAACATTTGCTCTAGTCTACACTGTTTA
TGCTGCTGGGGACACCAGGCGTGGCACAATGGGAGCCATTGGACCCATAGCAATTGGGTCCATAGCCGGAGCCAA
TGTGTTGGCAGCCGGGCCCTTCTCCGGCGGATCAATGAATCCGGCGTGCGCCTTTGGATCGGCTGTCACCGCCGG
CAGTTTCAAGAATCAAGCAGTTTACTGGGTTGGACCATTGATCGGTGCTGCAGCTGCAGGCCTTGTCTATGACAA
TGTTGTGTTCCCTAGTTCTCAAGCACCGGAGGGAGTTGGGGTG**TAA**TTCTATGCGATATTTACATTTTCTTTTCT
TTTGTTTCATCAGATTTGTATTTTATTAAGCTGTTTAACTGAATTCCTGAATGACTAATGATTATTTAGATGATC
TTTGCAATTCATTTTTTATCCTGTTTAAGATATAAGACAAAGAAGAGTATAGAAAGGGGTGCATCCCCCTCAAAA
ATTTTATAGATCTCCTAAAAATATATACTGTATGAAGTAGACCCTTAAAAATTAATTTCTTCCCTCTTTTTTATT
TTTATTTTATTTATTATTATTATTTTTTATAAGTTTTGCCTCCCACACTCAAATTTCTTGTTCATTGGACTTTCT
AGAATTCGGGTAAAGAGTTGTGAAACATTCGACATATCATTATAATTATGATGTTAAATGAATAAAATGATAGCA
ATGAACATTATCATGAATAGCTCTCTAGATGTCATCACCTGCCTTGTTTCAAAACAATGAATGAGCAATGTTTAT
AAAATAAATGTTTATACAAATCATACAACTTGCATATGTAACAGTGAAAATTAGTTATTAGATCAGCATTTATTA
AGAAAAAAATCATATTTAACCCCTAAGTTTTTAATTATCTGATTTGAATTTAGTTAGAGTTTATAATTTTAAGAA
TATGATCCTTAAATTTTGCCTAGATTTTAAATAGGCCCCTCTATCACTTTTTCGTCAAAATGAATGGTTTGTCAC

>*Bpe*TIP5;1
ATGGGTCCAACAATGGTAGCCGCCCGTTTCCAACATTCTGTCACTCCTAATGCGCTTCGATCATATCTCGCGGAG
TTTCTCTCCACATTCTTCTACGTCTTTGCGGTTGTGGGTTCTGCCATGTCATCGCGGAAATTGATGCCAGATGTT
GCGTCGGATGCATCCAGTTTGGTCGTGGTTGCCATCACCAATGCCTTTGCATTGTCGGCAGCCGTGTATGTTGCA
GCGAACATCTCCGGTGGGCATGTGAATCCGGCTGTCACATTTGGAATGGCGGTCGGAGGCCACATTAGTGTCCCA
ACCGCTTTGTTTTACTGGCTTTCGCAGATGCTGGCCTCTGTTATGGCTAGCCTTCTCTTGAAGATGACCGTTGTT
GGACAGCATGTTCCAACCTACGTTATTGCAGAGGAAATGACTGGGTTTGGAGCATCCCTGATGGAAGGTGTCCTA
ACATTTGCTCTAGTCTACACTGTTTATGCTGCTGGGGACACCAGGCGTGGCACAATGGGAGCCATTGGACCCATA
GCAATTGGGTCCATAGCCGGAGCCAATGTGTTGGCAGCCGGGCCCTTCTCCGGCGGATCAATGAATCCGGCGTGC
GCCTTTGGATCGGCTGTCACCGCCGGCAGTTTCAAGAATCAAGCAGTTTACTGGGTTGGACCATTGATCGGTGCT
GCAGCTGCAGGCCTTGTCTATGACAATGTTGTGTTCCCTAGTTCTCAAGCACCGGAGGGAGTTGGGGTGTAA

>*Bpe*TIP5;1
MGPTMVAARFQHSVTPNALRSYLAEFLSTFFYVFAVVGSAMSSRKLMPDVASDASSLVVVAITNAFALSAAVYVA
ANISGGHVNPAVTFGMAVGGHISVPTALFYWLSQMLASVMASLLLKMTVVGQHVPTYVIAEEMTGFGASLMEGVL
TFALVYTVYAAGDTRRGTMGAIGPIAIGSIAGANVLAAGPFSGGSMNPACAFGSAVTAGSFKNQAVYWVGPLIGA
AAAGLVYDNVVFPSSQAPEGVGV*

XIP – Uncharacterized *X* intrinsic proteins (XIPs)

*Bpe*XIP2;1 (original version)

>**FXXK01001578.1**|:125258-129233 Betula pendula genome assembly, contig: Contig1577, whole genome shotgun sequence
>**Bpev01.c1577.g0027.m0001**
Bpe_Chr3 19,966,597-19,968,432 (1) ::
join(19966597..19966637,19966768..19966855,19967620..19968432)

>*Bpe*XIP2;1
CAATGGAAGTCCAATGAGGCATCTGTCCAACACAGATTTCCAAATACGACCACTAAAGCTACACTGAAAGAAGAG
ATGATTTCTACTCTCTAGAGAGCCTCTGCAAAACACACATAGCACATCTCTCTAAAAGCCCCAACTAGCCATCCT
CTCACCAGTAACAAGACGATTTTGGACAGCATACCATAGAATGAACGCCTGCTTAGGGACGTAGAGTGGGAACCA
AATGAGCTTCCACCAAGGGACAACCGGTTTCTTGTCTCTAATATAGTCCCAAGTTTCACTACAAAGATACTTACC
AGTCTTAGAAATACTCTATCCAACCTAGTCAAGTTCACCCAACGGAACAAGGGAAAGCTGGCTTTGGATGCAAAC
CAGTGTCTCTGACCTAGCAGGAGGCCAAATCCACTGGTTATCAAGAATAACAGAAGAGAGTTTTGCATTAATATG

GCTGCCACTATCATATATGGGCCTAAACCCATATTTATCCTTAAGGACTCCATCCGGGTGCCACAGGTCAAGCCA
AAGAAAAAATTTTACTTCCATCCCCAACCGTGAATCGAATGAGGCCCCTTGCAATCTCCTTGAGTTTTAGCAGTTT
TCTCCATGTCCAAGAGCAATTTTGACGGATTTTCACAGCCCAAAAACTCCTTCCTTTGAGCACATAGGCTTCAAT
CCAAGCAACCCATAAAGATCTTGATTTCACAAAAAGGTTCCTTGCAATCTCCCTGATTTCACAAAAAGGTTCCAT
ATTATCTTTTGAGAATCTTCATAAAAATAATAATAATAATAAAAAAAGAGCTTGAAGATAACATTATAGACCCAT
GAGAGATTGTGGATCTTACCTAATTTCTAGAACTTCTGGTTCTCTCTCTCCAAAAAATCTAGCGCATGATTAAGC
AGCTTTCGCAACATGCCCTATTTAAACCATCCATAATAATTGGGTCATGATGATCGCAGCATGCCCTATTTAAAA
TGTTACCCTTGTGGTCCAGTGGGTAGTGGCTACCATTCTTTCATGGGCCTCCCATATTCTGTATAAGATAATTAA
GCTATTATTTTTTTTATTTTTATTTTCTGAGCAGATAATATATAAGCCCGATAGGTTGCTTGACATTGATGGACC
ATCCCCCTATTTATTATACTCCCCAACCTATTGGGTCAATTAAACACTTCATCTAGATTGTCTGCTTTACTATGA
TCAAGGGGCCACCGCACCTGTATTCACAGTTGACATTAAACACTACACTTCTGTGTAAGCATTTAATTCAGAATG
TCTTAATTTAGGGGTGGGGTGTAATATGTAATATTTTTTTGTCTTTTTTTTTTCAACAAAGGCTAATTTAAAGA
TTAGTTAAGACTGACATGTTAATATTTTAAAATAATTGCAAAATAAAAATATAATATATAACACTATTAAAATTA
ATTTTTAATAGTTATTAAATCTCATCACATCAACTTCAAAAAATATTTATAAGATATTTATGTCCATCTAACATT
ACCCTTTTGAACAAACTGCTCTGTCGAGTTTTGAAAGTCTGAGAAAAGGTCTTTGTAGGAAGCAGTAGCCGAAAA
TGTAGTTCAGGAAAAGCAGTTAGAAAAAAATAAAAGTTGACTAATATTATGATAGATATTATTATTATTATTATT
ATTATTATTATTATTATTATATATAGAATACAAAGGGTAAGCAAGAGAGCTAAAGAAGTAGTTGGGAGTTCAGAG
ATGGCTCAGAATGTGCAGAGAGTGGAGGATGAAGAAAGCTTGTACGTTGGAAGTAGAGTCCAACCTGTCCCCTTA
ACGCCATCGCAAGTACCCACATTTTTCTTCAATTCCTTAATTAATTCAGGGTGATACTATCATTCAAACTCATTT
CTTTTTCTAATAATATTGCAGGTCAGATCAACGTAGATTTGAGGAAGGGAATCGCAATACTAGTAAACTGAGTGA
GATTTTGGGCTTGGAGGAGTTCTTCTCCTTGAATGTTAGTATCATATTACATGCACTTAATAATGCAATTATTAT
TTTTGTTCTTTTGTTTTGGAAAATGCATTCATGGGAAATGGAGAGAACGGTATGTTTCATGGTTTAAATTAAATT
TTACATATTTAATTGTGTATATGTTGCGATGTTATTTAAAATTTTTAAATTATGTAGTCCATATACACTGTGAGA
TACAACAAATTACAATTTAGAACATGAAATTGGGAGAATTACTTCGAGAGAGGAGAGAAAGGAAATTATGACAAT
TAGAGTAGCCCCTTAGATTTATTGATTGAAGATCTTTTTTCATTAATAACTCATAAAAGAGTGGTTAAATACTCC
TATATTAATTACATTTAGGTTAAAAATACATAGATCTCCCTTGTGTCCTTTAACATTACACTCTTTAGTCCATGT
TTGAAAATTTTGTCAATATAACAAAAATAGACAATCTCATCCTCATGATATTTGACTGAAAGTCACTTTTAAAGG
GGAAAAAGGAAAAAAATCAAAATATAATATGCACAAAGAAAAGAGAAGGTACAAGTTTGAGACTTTTGGATCAAA
GAAAATGTTTATGAAAATCTTATAATAATATAATTAGGATTAAACTAGGCATTTTAATTTATTCTACGAGAAAAA
TTGTTAAGTTGGTTTGTTTGATTCGACATTGTAACAAATAAATTCATGTGGTATGCCTCCGTAACAAAATGATAA
TTCTGTATAATTTTGACCATTTTTCCAACATTATGTGATGAATCACAGGTTTGGCGAGCATCTTTGGCAGAGCTC
CTTGCTACGGCAATCTTTGTTTTTGCCATTGACACCATAGTCATATCCTCCTTTGAAACTAAAACGGAATCACCA
AAACTTATAATGTCACTCCTTATCGCTATCATTACCGCAATTCTCCTTCTCGCCGCCAATCCGATTTCTGGTGGC
CACATGAACCCTGCGATCACCTTAGCGGCCGCACTTGTTGGCGTCATTTCCGTTTCGCGGGCAGCCGTATACATT
TTGGCGCAATGTGCTGGTGCTGTGCTAGGTGCACTAGCACTCAAAGCCGTGGTAAACAGCACCATTGAAGACACA
TTTTCCCTTGGAGGCTGCACTCTCACTGTCATTGCACCGGGACCAAATGGGCCGATTTCTGTTGGGCTTGGGACA
AGCCAAGGCCTCTGGCTGGAGATAATATGTACATTTGTGCTTCTTTTTGCTTCAATATGGATAGCTCTTGATCAT
CGCCAAGCCAGGGCTCTGGGCAGAGTTATCATTTTCTCTGTCATCGGAATTGTAGTAGGCCTTCTTGTGTTTGTG
TCAACGACGGTGACAGGAGCCAAAGGCTACGCCGGCGCCGGAATGAACCCAGCAAGGTGTTTGGGCCCAGCAATT
GTTAGAGGGGGCCATCTCTGGAATGGGCACTGGGTGTTTTGGGCTGGGCCATCAATTGCCAGCGTGGCATTTTAT
CTCTACACTAAGATAATTCCAAGTCAGCATTTCCATGCCCAAGGGTATAAACATGATTTCTTGAACATTTTGAAG
GCTCCCTTTACATCAGACTCTGTTCACAAGAAG**TGA**AAAAATGGACAGGTGCATGCCTTGAGAAAAATGTGTAAA
T

>*Bpe*XIP2;1

ATGGCTCAGAATGTGCAGAGAGTGGAGGATGAAGAAAGCTTGTACGTTGGAAGTAGAGTCCAACCTGAAGGGAAT
CGCAATACTAGTAAACTGAGTGAGATTTTGGGCTTGGAGGAGTTCTTCTCCTTGAATGTTTGGCGAGCATCTTTG
GCAGAGCTCCTTGCTACGGCAATCTTTGTTTTTGCCATTGACACCATAGTCATATCCTCCTTTGAAACTAAAACG
GAATCACCAAAACTTATAATGTCACTCCTTATCGCTATCATTACCGCAATTCTCCTTCTCGCCGCCAATCCGATT
TCTGGTGGCCACATGAACCCTGCGATCACCTTAGCGGCCGCACTTGTTGGCGTCATTTCCGTTTCGCGGGCAGCC
GTATACATTTTGGCGCAATGTGCTGGTGCTGTGCTAGGTGCACTAGCACTCAAAGCCGTGGTAAACAGCACCATT
GAAGACACATTTTCCCTTGGAGGCTGCACTCTCACTGTCATTGCACCGGGACCAAATGGGCCGATTTCTGTTGGG
CTTGGGACAAGCCAAGGCCTCTGGCTGGAGATAATATGTACATTTGTGCTTCTTTTTGCTTCAATATGGATAGCT
CTTGATCATCGCCAAGCCAGGGCTCTGGGCAGAGTTATCATTTTCTCTGTCATCGGAATTGTAGTAGGCCTTCTT
GTGTTTGTGTCAACGACGGTGACAGGAGCCAAAGGCTACGCCGGCGCCGGAATGAACCCAGCAAGGTGTTTGGGC
CCAGCAATTGTTAGAGGGGGCCATCTCTGGAATGGGCACTGGGTGTTTTGGGCTGGGCCATCAATTGCCAGCGTG
GCATTTTATCTCTACACTAAGATAATTCCAAGTCAGCATTTCCATGCCCAAGGGTATAAACATGATTTCTTGAAC
ATTTTGAAGGCTCCCTTTACATCAGACTCTGTTCACAAGAAG**TGA**

>*Bpe*XIP2;1

MAQNVQRVEDEESLYVGSRVQPEGNRNTSKLSEILGLEEFFSLNVWRASLAELLATAIFVFAIDTIVISSFETKT
ESPKLIMSLLIAIITAILLLAANPISGGHMNPAITLAAALVGVISVSRAAVYILAQCAGAVLGALALKAVVNSTI
EDTFSLGGCTLTVIAPGPNGPISVGLGTSQGLWLEIICTFVLLFASIWIALDHRQARALGRVIIFSVIGIVVGLL
VFVSTTVTGAKGYAGAGMNPARCLGPAIVRGGHLWNGHWVFWAGPSIASVAFYLYTKIIPSQHFHAQGYKHDFLN
ILKAPFTSDSVHKK*

***Bpe*XIP1;1**

>**FXXK01001578.1:**101348-103631 *Betula pendula* genome assembly, contig: Contig1577, whole genome shotgun sequence
>**Bpev01.c1577.g0026.m0001**
Bpe_Chr3 19,989,890-19,991,672 (1) ::
join(19989890..19990015,19990881..19991672)

>*Bpe*XIP1;1
TCCTTGTTTATGTAAAATGAATATTCTTGCAAGCATATATTTGATCATAGTATGAATTGAAGTATTCTAAGACAG
ACATGCATCCTTGTTTATGTAGAATGAATATGCTTGCAGATTGCAATTGACTTCCACGCCTCACTGCAGCCATTA
AAAAAAATCAATTGGATAAAGCGTATAACTCAATATATGCTTTCACCGTCTTCTGGGTAACGCCAGAAACGTGGC
CACTTCAAAAAAAAAAATCTGTCGAAGCAGCCAAGTTTGGCGTTGGCTGGATTTTTCCCATTCTCTTCTATAAGT
TTCCATGAAATTAGATTCTTTGAATTGGTGGTAATTCTCTTCACTTTCTCTACAACTTATAAGGTC**ATG**GATATG
GTTGTCCCCGGACAAGGCGATCAGGAATCTCCAACATCATTTGAGAAATGCGAAGCACAAAATTTTAGAACAAAA
TTTCTTGTTTCCATAGGTGCCCATGAGTATTTCTCCCCAGAGGTATGCTTATTTATTTATTGTGTTCAGATATAG
TAGCTAGTTAGGTCCTTCCCGTTCAATTTCTCACAAATACTATTCCAATTTGCTCATGGGCTATCGAGTTGTAAC
AACAAAAAAAAAATTGTAATAAAGTTGTTAAACATGATTTACCTCCCTTAATTTTATATATTTTATGTGGAACAA
AAATAGATTTTAGAGAAATGATCTTTTTACATCTCTTTTTTAAATCAACTATTGAATTTGTGAGTCATATGAATT
CAAATATTAATTTAAAATAGAGATGTACATGATACGTATAAGAGATTATGTGTAACATGCGTGTCTCTAGATTTT
AGGAGAAACTACACTTACCTCTTAAACTACTATCAAATTTGCAATAACCCCCAAACTTCAAAAGGTTGCAATGTA
TATTACCAATAACTTAAGTTTTTTTGGTAGGGTACATTGTAACTTTTTTAAGTTTGGGTAGACATTATAAATTAG
TTAGTAGTTTGAGGGAGGTAAGTGTAGTTTTCTTTCTACTTTACTATTGGTCTAAAGATCATTCATTCAAATATA
ATCTCTACAATTATATTTCTTATCTCTAATTGTTCCACTTATGTTATGCCAATACTTAAGTACTTGATGGAGAAA
ATATATAGCAGCCTCCTAAACAATCACAATATTTTTATTTACACCCCCAAACTTTAAAAAGTGACATTAAGGCCC
TTTGAACTACTACCGAATGTCAAATTAAACATTTTTTGCATTTTTCTTCCCAAAATACCTTTGAAGTTACTTAAA
AAAAAAAAAACAATTTAAAAAAAAAGTTAAGGGGCTTAATATGAAAAGTGCTAACATTTTTTCATCTTGATTGTTT
CTTTCAGATGTGGAGGGGAGCCCTCACCGAGTTGGTAGCAACTGCCTTTCTTATGTTCACCCTCATCTCTTCCAT
CATCGCATGCTTGGACTCACAAGAGGTCGATCCCAAGCTTCTTGTCCCCCTCGCCGTCTTCATTATAGCCTTCCT
ATTCCTCTTGGCCACAATTCCTTTATCTGGCGGCCACATGAACCCCATCTTGACATTCATAGCCTGCCTCAAGGG
TCTCATAACTCTTGCCCGCGCTTCCATCTACGTTTTGGCACAATGTCTTGGCTCAATAATCGGTTTTTTTATAAT
AAAAAATGTGATGAACCACCAAACAGGGCACAACTATTTCTTAGGCGGATGCACAATATCTGCACTAAACCGAGG
GACTGCATTGGTTTTGGAATTTTGTTGCACATTTGTGCTGCTGTTTGTTGGTGTGACGGTGGCGTTTGACAAGAA
AAGGTGCAAGGAACTTGGGCTAGCAGTGGTGTGTGCTGTGGTGGCAGCGGCCTGGGCACTGGCTGTGTTTGTGTC
CATAACTGTTACTGGGCAGGTTGGTTATGCAGGTGTAGGCTTGAACCCTGCAAGATGCTTGGGCCCTGCATTGTT
GCATGGAGGGGTGTTGTGGGATGGGCATTGGGTTTTCTGGGTTGGCCCCTTTTTGGCTTGCATTGTTTATTATGG
TTACTCTATGAGCTTGCCTAAGGAGGGTTTGGTAGAGGTAGAGGGGGAGTATGACATTTCCAGGTTAGTTGGGGC
TTGTTTTGGGGAAAATGGCAGTCCAACTTCTCTTGAAGGAAAATTT**TAG**GTAATGTGTTTGGTTTGGTCCAATTT
CAATAGATCATGCAGTTACCATGTGAAATGATTAGGGGTGTAAATGTGTATGCGGTTACAGTATTGGCAATATTT
GCATCCGCATAATATAGTTATTAATTTTAAGTATCCGCATCCGAATCATGTAATTATTATCTACATCCACACAAT
TATTATCTGCTATTCGCATATGAGGTGATAAGCCTTTTTAAACCTTTAAGTTGTAATTCCTGCTTCTAATAAATA
CAAAAATGCATTTTAGTTTTTTTTTTTTAATTAAATAATCAATGCAATAGCCAGAGATAAGTACTTGATATTGTC
GATAGGTTCCATTTCTTTCTATATTGCGAGTGATAAAACTAAATTTATGGTCTAGACGTGTTTTTCTTTTTTAGT
AGTAAAATCTGCAAATTGTATGAACAATACGGACAAAATGTACATAAAGATATTTTGGGAGGAATAAATAATTCT
TACAAGATGTCTAAAAGCAAACTAAACGTAAAAATTTTATATTTCTAGAAATCTTATCAGATTCCTAATTGTTAG
AGTAATATTCTGGTGGTTTTCCTCCATGGGTCAACGATTTTAATAGTCACTGTACGGTTGGACTGAGGGGATGAA
ATACTTGC
>*Bpe*XIP1;1
ATGGATATGGTTGTCCCCGGACAAGGCGATCAGGAATCTCCAACATCATTTGAGAAATGCGAAGCACAAAATTTT
AGAACAAAATTTCTTGTTTCCATAGGTGCCCATGAGTATTTCTCCCCAGAGATGTGGAGGGGAGCCCTCACCGAG
TTGGTAGCAACTGCCTTTCTTATGTTCACCCTCATCTCTTCCATCATCGCATGCTTGGACTCACAAGAGGTCGAT
CCCAAGCTTCTTGTCCCCCTCGCCGTCTTCATTATAGCCTTCCTATTCCTCTTGGCCACAATTCCTTTATCTGGC
GGCCACATGAACCCCATCTTGACATTCATAGCCTGCCTCAAGGGTCTCATAACTCTTGCCCGCGCTTCCATCTAC
GTTTTGGCACAATGTCTTGGCTCAATAATCGGTTTTTTTATAATAAAAAATGTGATGAACCACCAAACAGGGCAC
AACTATTTCTTAGGCGGATGCACAATATCTGCACTAAACCGAGGGACTGCATTGGTTTTGGAATTTTGTTGCACA
TTTGTGCTGCTGTTTGTTGGTGTGACGGTGGCGTTTGACAAGAAAAGGTGCAAGGAACTTGGGCTAGCAGTGGTG
TGTGCTGTGGTGGCAGCGGCCTGGGCACTGGCTGTGTTTGTGTCCATAACTGTTACTGGGCAGGTTGGTTATGCA
GGTGTAGGCTTGAACCCTGCAAGATGCTTGGGCCCTGCATTGTTGCATGGAGGGGTGTTGTGGGATGGGCATTGG
GTTTTCTGGGTTGGCCCCTTTTTGGCTTGCATTGTTTATTATGGTTACTCTATGAGCTTGCCTAAGGAGGGTTTG
GTAGAGGTAGAGGGGGAGTATGACATTTCCAGGTTAGTTGGGGCTTGTTTTGGGGAAAATGGCAGTCCAACTTCT
CTTGAAGGAAAATTT**TAG**

>*Bpe*XIP1;1
MDMVVPGQGDQESPTSFEKCEAQNFRTKFLVSIGAHEYFSPEMWRGALTELVATAFLMFTLISSIIACLDSQEVD
PKLLVPLAVFIIAFLFLLATIPLSGGHMNPILTFIACLKGLITLARASIYVLAQCLGSIIGFFIIKNVMNHQTGH
NYFLGGCTISALNRGTALVLEFCCTFVLLFVGVTVAFDKKRCKELGLAVVCAVVAAAWALAVFVSITVTGQVGYA
GVGLNPARCLGPALLHGGVLWDGHWVFWVGPFLACIVYYGYSMSLPKEGLVEVEGEYDISRLVGACFGENGSPTS
LEGKF*

*Bpe*XIP1;2

>**FXXK01001578.1**|:129871-134095 *Betula pendula* genome assembly, contig: Contig1577, whole genome shotgun sequence
>***Bpev01.c1577.g0028.m0001***
*Bpe*_Chr3, 19961773..19962564) Genomic Location: 19961773-19962564

>*Bpe*XIP1;2
TTTGTTTATGGAAAAATACAAAGTAATTAAACAGTAGATATACAAATACATAATTTAAACACAGAAATTTAAACA
CATAAATAATTGGAGAACGAACTAACCGGACCGGTACCGGTTTGGTTTTTAAATAATGAGTTAAAATTCGGTTT
TTGCTTAAAAGTTATGGGTTCACGCCACGTGTCGCAATTCTAGGCACTTTGAATTGCTTAAAAATTATGGGTTCA
CGCCACGTGTCGCAATTTTAGGCACTTTGATGTGTGATTCAGCTATTATATATATATAGATTTCCTTATTCAATT
AAAAAAAAAAATAATAATAATACTTCAACCCTAACTCATTAATTTTCAACCCTAACCCATTTATGTTGAATTTAT
GAATCTTATCAAAAAATTGTTAGCCGTACTCCCTCCTATCTTTCAATCTTTAATTTTGGCACAATTATACGGAAA
TGAACCACGTACGTAAGAAGTTCCATCTGATAGTCTTTGGAATAAAGTCACAATCAAACTTTATATAGGAGCATT
TAAAGGAATCTTAACCATTCCATTTTATACAAGAGGTTATCATGCACACAAGGAGTTTTAAGATAATTTTTGTAT
TCAAGCATCAATTTGATATAAACATAAACCTCAAAAACTAATTTAATAATCTTCCCTTCATATTGTTGTAATATT
TACGATATCAAGGTGCAGCAGTGTAGATATTTGGCCTCACATTTGGTTATTAGTAACTGCATTAATTTATTTAAT
CCTTGTTTATGTAAAATGAATATTCTTGCAAGCATATATTTGATCATAGTATGAATTGAAGTATTCTAAGACAGA
CATCCTAGCTTGTTTATGTAGAATGAATATGCTTGCAAATTGCAATAGACTTCCACGCCTCATTCCAGCCATTAA
AAACATCAATTGGATAAAGCGTATAACTCAATATATGCTTTCAACGTCTTCTGGGTAACATCACGGCCACTTCAA
AAAAAAAAAAATTCTGTCGGAGCAGCCAAGTTTGGTGTTGGCTGGATTTTTCCCATTGTCTTCTATAAGTTCCCA
TGAAATTAGATTTTTTGAATTGGTGGTAATTCTCTTCACTTTCTTTACAACTTATAAGGTC**ATG**GATATTGTTGT
CCCCGGACAAGGCGATCAGGAATTTCCAACATCATTTGAGAAATGCGAAGCACAAAATTTTAGCACAAAATTTCT
TGTTTCCGTAGGTGCCCATGAGTATTTCTCCCCAGAGGTATGCTTATTTATTTATTGTGTTCAGATATAGTAGTT
AGGTCCTTCCCGTTCAATTTCTAACAAATATTATTGCAATTTGCTCATGCGCTATCGAGTTGTAAAAAAAAAAAA
AATAGTAATAAAGTTGTTAAACATGATTTACCTCCCTTAATTTTATATATTTTATGTGGAATGGAAATAAATTTT
AGAGAAATGATATTTTTACATCTCTTTTTTAAATTAAACATTGAATTTGTGAGTCATATGAATTCAATAATTGAT
TTAAAATAGAGATGTATAAGAAATTATGTGTAACATGCGTGTCTCTAGATTTTAGGAGAAACTACACTTACCTCT
CAAATTACCATCAAATTTGCAATAACCCTCATACTTCAAAAGGTTGCAATGTATACTACAAATAACTTAATTTTT
TTTTTCTTCGTTAGTATTATTCTAATGGAGGGATAACTAAAAGGGTTTGGTCGATTGGTAGGGTACATTGTAACT
TTTTTAAGTTTGGATGGACATTATAAATTAGCTAGTAGTTTGAAGGAGGTAAGTGTAGTTTTCTCTCTACTTTAC
TATTGGTCCAAAGATCATTCATTCAAATATAATCTCTACAATTATATTTCTTATCTCTAATTGTTTTACTTATGT
TATGCCAATACTTAACTACCTGATGGAGAAAATATATAGCAACCTCCTAAACAACCACAACATTTTTATATACAT
TCCCAAACTTTAAAAAGTAACATCGAGGCCCTCTGAACTACTACCAAATGTCAAATTAAACATTTTTTGCATTTT
TCTTCCCAAAATACGTACCTTTGAAGTTACTAAAAAAAACAATTTCAAAAAAAAAATTAAGGGGCTTAATATGAA
AAGGGCTAACATGTTTTGATCTTGATTGTTTCTTTCAGATGTGGAGGGCAGCCCTCACCGAGTTGATAGCAACTG
CCTTTCTTATGTTCACCCTCACATCTTCCATCATCGCATGCTTGGACTCACACGAGGTCAATCCCAAGCTTCTTG
TCCCCCTCGCCGTCTTCATTATAGCCTTCCTATTCCTCATGGCCACAGCTCCTTTATCTGGCGGCCACATGAGCC
CGATCTTCACATTTATAGCCTGCCTCAAGGGTCTCATAACTCTTGCCCGCGCTTCCATCTACGTCTTGGCGCAAT
GTCTTGGCTCAATCATCGGTTTTTTTATAATAAAAAATGTGATGAACCACCAAATAGGGCACAAGTATTTCTTAG
GCGGATGCACAATATCTGCACTAAGCCGAGGGACTGCATTGGTTTTGGAATTTTGTTGCACATTTGTGGTGCTGT
TTGTTGGTGTGACGGTGGCGTTTGACAAGAAAAGGTGCAAGGAACTTGGGTTAGCAGTGGTGTGCGTTGTGGTGG
CAGGGGCCATGGCATTGGCTGTGTTTGTGTCCATAACTGTTACTGGGCAGGCTAGTTATGCAGGTGTAGGCTTGA
ACCCTGCAAGGTGCTTGGGCCCTGTGTTGTTGCAAGGAGGGGTGTTGTGGGATGGGCATTGGGTTTTATGGGTTG
GCCCCTTTTTGGCTTGCATTGTTTATTATGGTTACTCTATGAACTTGCCTAAGGAGGGTTTGGTAGGGGTAGAGG
GGGAGTATGACATTTCCAAGTTAGTTGGGGCTTGTTTTGGGGAAAATGGCAGTCCAACTTCTCTTGAAAGAAAAT
TT**TAG**GTAATGTGTTTGGTTTGCTCCAATTTCAATATATCATGCAGTTACCATGTCAAATGACTAGGGGTGTATA
TGTGGATGCGGTTGCAGTTATTAGCAATATTTGCATCCGCATAATGTAGTTATTAATTTTAGATATTCGCATCCG
AATTATGTAATTATTATTTGCATCCACACGGTTATTATAGTTATAATCTGCTATCCGCATATGAGGTGATAAGCC
TTTTAGACCTTTAAGTTGTAATTCCAGCTTCTAATAAATACAAAAAGGCATTATAGTTTTTTTATTTTTTTTAGT
AAATACTCAATGCAATAGCCAGAGATAAGTACTTGATATTGTCCATAGGTTCCATTTCTTTCTATATTGCGAGTG
ATAAAACTAAATTTATGGTCTAGACGTGTTTTTTTTTTTTGTTAGTAGTAAAATGTGCAAATTGTATGAACAATA
TGGACAAAATGTACATAAAGATATTTTGGAGGAATAAATAATTCTTACAGGATGCCTACAAGCAAACTAAACAT
AAAAATTTTATATTTCTAGAAATCTTATCAGATTCCTAATTGTTGAAGCAATATTCTGATTGTTTTTCTCCGTTG

GTCAACAATCTTAATAGTTATTGTACGGTTGGACTGAAGAGATGAAATACCTGCAAAACAAAAAGATTGTCACAA
AGTTTTTAACTGGAGCCGAAGACACTCCAATGATTAAGTCAATACAGTTCATATTTGGAGTGTAAGAATGGCTTG
GTTATGGAGTGAACAAGCCCGTGTGTTAGAGAACATAAGAAAGAGAGAGAGAGAGAGAGAGAGAAGGAAAGCTCT

>*Bpe*XIP1;2

ATGGATATTGTTGTCCCCGGACAAGGCGATCAGGAATTTCCAACATCATTTGAGAAATGCGAAGCACAAAATTTT
AGCACAAAATTTCTTGTTTCCGTAGGTGCCCATGAGTATTTCTCCCCAGAGATGTGGAGGGCAGCCCTCACCGAG
TTGATAGCAACTGCCTTTCTTATGTTCACCCTCACATCTTCCATCATCGCATGCTTGGACTCACACGAGGTCAAT
CCCAAGCTTCTTGTCCCCCTCGCCGTCTTCATTATAGCCTTCCTATTCCTCATGGCCACAGCTCCTTTATCTGGC
GGCCACATGAGCCCGATCTTCACATTTATAGCCTGCCTCAAGGGTCTCATAACTCTTGCCCGCGCTTCCATCTAC
GTCTTGGCGCAATGTCTTGGCTCAATCATCGGTTTTTTTATAATAAAAAATGTGATGAACCACCAAATAGGGCAC
AAGTATTTCTTAGGCGGATGCACAATATCTGCACTAAGCCGAGGGACTGCATTGGTTTTGGAATTTTGTTGCACA
TTTGTGGTGCTGTTTGTTGGTGTGACGGTGGCGTTTGACAAGAAAAGGTGCAAGGAACTTGGGTTAGCAGTGGTG
TGCGTTGTGGTGGCAGGGGCCATGGCATTGGCTGTGTTTGTGTCCATAACTGTTACTGGGCAGGCTAGTTATGCA
GGTGTAGGCTTGAACCCTGCAAGGTGCTTGGGCCCTGTGTTGTTGCAAGGAGGGGTGTTGTGGGATGGGCATTGG
GTTTTATGGGTTGGCCCCTTTTTGGCTTGCATTGTTTATTATGGTTACTCTATGAACTTGCCTAAGGAGGGTTTG
GTAGGGGTAGAGGGGGAGTATGACATTTCCAAGTTAGTTGGGGCTTGTTTTGGGGAAAATGGCAGTCCAACTTCT
CTTGAAAGAAAATTT**TAG**

>*Bpe*XIP1;2

MDIVVPGQGDQEFPTSFEKCEAQNFSTKFLVSVGAHEYFSPEMWRAALTELIATAFLMFTLTSSIIACLDSHEVN
PKLLVPLAVFIIAFLFLMATAPLSGGHMSPIFTFIACLKGLITLARASIYVLAQCLGSIIGFFIIKNVMNHQIGH
KYFLGGCTISALSRGTALVLEFCCTFVVLFVGVTVAFDKKRCKELGLAVVCVVVAGAMALAVFVSITVTGQASYA
GVGLNPARCLGPVLLQGGVLWDGHWVLWVGPFLACIVYYGYSMNLPKEGLVGVEGEYDISKLVGACFGENGSPTS
LERKF*

***Bpe*XIP2-like** (original version)

>**FXXK01002938.1:**21257-23881 Betula pendula genome assembly, contig: Contig2937, whole genome shotgun sequence

>Bpev01.c1577.g0027.m0001

>BpeXIP2-like

TCTCTTGAAAGCTGGTTCATATGTAGGGCCGTTTCAATCTAATGATTCACTATTTTCTCATAATTTTTTATATCC
TCACTTCAAAATTACAAAATATTGTCATTCACCATTTGTTAAGAGTATTTTTTTGTTAAAAATATATTATTTCAA
TCAAATCCCAAACACTCCATCTTCTCATCTTTATTTTTATCTATCAACACTTATAGTTTTATAGTTTCTCTTTGT
TTTTGACTTTTAATAGGCTCTAAATTTATGCCGCTTTCAAATGTTTGTATTAGAATACACTCATATAAATACTGT
TAAAACATTTTTTCAGCTTTGGATCATAATAATCATATATTCAAAGATGCAACATTCCCGGTGTGTTCCCTATAT
ATATACCGTCTCCAAACGAGGTATCTTGTATCATAATACCTCATCTGATGATGGACCAAGCCTTCATAAGTTTCA
CTTCTTGTGAAGATTCGTATTATACCCAAAATACAACTCCTCCTTGAGGGATAAACAGCTAGAAATGCGTGAATT
AAAAATCACACCATCATCACAACTTATCCTGACATGGTCTTTTGATTTCTAGATGGTGAAATCTCATGCTTGGTC
GACCTTGGTTCTGGTGTGAGATAGGCAAGTTTTGTTGACCAGACAAGTGATGGTGAAGAGATACTCAGTGGAACT
AACGGCTTGTTGGTGTAAGGATTGATATCTACATATCAAGATAGTAATTATCGGACTTGACTTGTTATGTCTATT
GTGATACATTAAAGGTATTATGCGAATGGGTTTAAGATTATTTAAGTATGTAATATTTGGAGTTGGACTACGGCA
ATCAAAAATGAGGTAGCAAGCCATTATTAGAGATTGCATAAAATAGAAACTGAATATCATTGATTGGGAGTTCAT
AGCATGATGCAGACTCACAGAAATGGTCGCATCCGCAGGAGAGTATTGAGTGAAAGACATGGAGCGCTATGATAA
TATGACGTGTGAAACTGAATAAGTGAATCGCCTACCACCTATAGTCCACTCAACGCCAGCGAAAGTGTGCGCATT
CTGGAGTTGTCAATAACCGGGTCAGACCATGTGGAACTGTATAAGGGCTCTCTCGTTTTTATTTTTAAAATACTA
GCAAAATTGGGGGAGGGTAGGATTTTTTTATCTCGTTGAAGTGTTGCAATTCTTTCTATTTTCTTATACTTTTCT
TAAATTTGTAAATTGCTTTATTTGCAGCCAGAAATTACGAATCCTATACAACGTAAAAGATTCGAGGAAGGGAAT
CAGCAAGTTTTAAATACTAAGTACAACTGAGAGTGTTTGTATGAAGCGGCTTGGAGGAGATTTCATGTTCCTCAC
GATGAAATGTTAGTATCTATACTATGAAATTACGAGCATACTAACATAAATCTCTGACATATTTATTTATTTATT
TCTTCCATTAATTTGTGTATTTGAGGAAATGAATTCTAGGAAAGGCTTAGTGGAGCGAAGAAATGTTTCTCTATT
TGGGCAGGTCTGGACAAACAAAAAGTTTATGTAACAAATCTGTGTATCAGGCAGTAATATAATTGCGGACGGTAT
GAGTTCATAAAATTCTGTATAAAGATGATGTTGACTCTGTGGATTCAACATGGCTCGTACTGCGTATGATGGGGA
TGCCAAATATTAGAGATTATGTATCACCGAAATAAAATTGTAAATTTGTATTTTTGTTTGTGGAGGAGAGTGATA
GATAAATTGTGTATACAATTAGATAGAGTCCCTTTTATTTATATTGAATTGAAGATCTATTTTTATCATTATATT
ACTCACAAAGTATGAGTGAGTTATATATATCATTACCCTATTTACCAGAACTGTGTGGGAATTTAATTGACTTTA
AATTGAATCTTCGCCTTTTCTTTGTTAAACAATAAAGAAAGTAGAATAAAGGTTGCGTGATGCAGCCACCTGAGG
CATTACGGGCTCATGATCGTATCGCCCATCCTTCTCTTGGAGTGTAATTCCGCAAAATGCATCGGACAGGCTCCT
TCATGTCAGTGGTATTGGGTCTCTCGGCTTTTCACTGGTACCCACTGTCGGCGCGTGTCTCTCACGCGCCGGTGA
GTGGTCCATTTGCACCGGCCACTTTCCTCCGCCCAGCCCTCCGCCGTTGTTTGCTGTTTACTGTTTTTTTAAATT
TCCTTGTAATTTGATGTTGTTGTGTAATTTGCTGTTGTTGTGTTGCTCAGACTCTCGGTCTGGGTTGAAACCCCC
TTTTTAATTTTTTCTCTTTATTTTGTTCAGTTTCCTCCGAAGCTGGATGAGATTAAAGTTTTTTAGTCCTGCTTG

GTTTCTTAGGAAGTCAGATTAGATCTAAGGTCTTAGGAGTTTTCGAACTCCCGATGGTTTTATCCATCAGATTGC
GCCGCGGCCTTCAAAAGCTGCATTCGCAGATTACTTTCTCCTCCTTGGGTTTTTTCCCTTATTGTGAAGGTGCTA
GTGGTTTGTCTGATTAGGATTTTTCAGTTGAGCAACGTCTTGGTAGTTCTTGCTTTTAATTTTCTTGTAAGGTTT
TAGCCTTATGTTTCTGTAATGGTTCCCTTTAATATATGTGATGATGCACTTACAAAAAAAAAAAAAAAAAGAAAA
TTTTGTCGATATAACATAAATAGACAATCCCATCCTCATGATATTTGACTGAAAGTCACTTTTAAAGGGAAAAA
AGAAAATCAAAATATAATATGCACAAAGAAAATAGAAGGTACAAGTTTGAGACTTTTGGATCAAAGAAAATGTTT
ATAAAAATCTTATAATTATATAATTAGGGTTAAGCTAGGCATTTTAATTTATTCTATGAGAAAAATTGTTAAATT
GGTTTGTTTGATTCGACATTGTAACAAATAAATTCATGTGGTATGCCTCCGTAACAAAATGATAATTCTGTATAA
TTTTGGCCATTTTTTTTGACATTATGTAATGAATCACAGGTTTGGCGAGCATCTTTGGCAGAGCTCCTTGCTACGG
CAATCTTTGTTTTTGTCGTTGACACCATAGTCATATCCTCCTTTGAAACTAAAATGGAATCACCAAAACTTATAA
TGTCACTCCTTATCGCTATCATTACCGCAATTCTCCTCCTCGCCGCCAATCCGGTTTCTGGCGGCCACATCAACC
CCGTGATCACCTTAGTGGCTGCACTCGTCGGCCTCATTTCCGTTTCACGGGCAGCTGTATACATTTTGGCTCAAT
GTGCTGGTGCTGTGCTAGGTGCACTAGCACTCAAAGCCGTGGTAAACAACACCATTGAAGACACATTTTCCCTTG
GAGGTTGCACTCTCACTGTCATTGCACCGGGACCAAATGGGCCGATTTCTGTTGGGCTTGGGACAAGCCAAGGCC
TCTGGCTGGAGATAATATGTACATTTGTGCTTCTTTTTGCTTCAGTATGGATAGCTCTTGATCATCGCCAAGCCA
GGGCTCTGGGCAAAGTTATCATTTTCTCTGTCATCGGAATTGTAGTAGGCCTCCTTGTGTTTGTGTCAACGACGG
TGACAGGAGCCAAAGGCTACGCCGGCGCCGGAATGAACCCAGCAAGGTGTTTGGGCCCAGCAATTGTTAGAGGGG
GCCACCTCTGGAATGGGCACTGGGTGTTTTGGGCTGGGCCATCAATTGCCAGCGTGGCATTTTATCTCTACACTA
AGATAATTCCAAGTCAGCATTTCCATGCCCAAGGGTATAAACATGATTTCCTGAACATTTTGAAGGCTCCCTTCA
CATCAGACTCTGTTCACAAGAAG**TGA**AAAAATGGACAGGTGCCTTGAGAAAAATGTATTCTGTAAATCTGTTGCC
ATGATGTAATAACTCAGCATGTATCAGCCTGTGATTATGATGTGTTTACGTTTTCTAATGAAAAACCAATTACCC
CATATTAGTTTTGACTTTTTTTTTTTCTTTAATTGAAAGGGAAAAAGGCTACAAGATCTGTTGGGAAGAGAAAAT
GTGAGATCCTATAGAAGTGCTTCCGTGGTGGAAAGCGGCCCACTTAGCTAAGACATGTGCACGATAATTGACACA
TCTAAAAACGTTCAAAGCATTCTAGCTATGAAATGATACTAAATCTAAACTAATGTTCGAAATTACATTAGCAAA
ATTCAAAGAGAAAAAAAGAAGGGGCTGATTTTTGACTTGCATGGAAAATGTTGTGACAAAAGGAAATTTAAAATT
TTCAAAGAAGTAGAAAATAGTAAAATATAAAATAAATTAATCACACAAGACACCAAGATTTAAGTGGTTCGGCGT
AACAATATATTCACTGGCAGAGGCGATCCAGAAGAAATTCACTAATAAAAGATGAAGTACAAAAGAGTAATAGAG
AGAAAAACACTTAAACTCAAAGACCCAATACATCCAAATCTCACTTTTACGCAAAAGATAAAAAAACAACAAAAG
GGTAATCTCTCTTTCTCTCTAAGTGCGCGTGGCACAACAAAGCGATCTATTAGTTGCCGTGCTTG
>BpeXIP2-like
GTTTGGCGAGCATCTTTGGCAGAGCTCCTTGCTACGGCAATCTTTGTTTTTGTCGTTGACACCATAGTCATATCC
TCCTTTGAAACTAAAATGGAATCACCAAAACTTATAATGTCACTCCTTATCGCTATCATTACCGCAATTCTCCTC
CTCGCCGCCAATCCGGTTTCTGGCGGCCACATCAACCCCGTGATCACCTTAGTGGCTGCACTCGTCGGCCTCATT
TCCGTTTCACGGGCAGCTGTATACATTTTGGCTCAATGTGCTGGTGCTGTGCTAGGTGCACTAGCACTCAAAGCC
GTGGTAAACAACACCATTGAAGACACATTTTCCCTTGGAGGTTGCACTCTCACTGTCATTGCACCGGGACCAAAT
GGGCCGATTTCTGTTGGGCTTGGGACAAGCCAAGGCCTCTGGCTGGAGATAATATGTACATTTGTGCTTCTTTTT
GCTTCAGTATGGATAGCTCTTGATCATCGCCAAGCCAGGGCTCTGGGCAAAGTTATCATTTTCTCTGTCATCGGA
ATTGTAGTAGGCCTCCTTGTGTTTGTGTCAACGACGGTGACAGGAGCCAAAGGCTACGCCGGCGCCGGAATGAAC
CCAGCAAGGTGTTTGGGCCCAGCAATTGTTAGAGGGGGCCACCTCTGGAATGGGCACTGGGTGTTTTGGGCTGGG
CCATCAATTGCCAGCGTGGCATTTTATCTCTACACTAAGATAATTCCAAGTCAGCATTTCCATGCCCAAGGGTAT
AAACATGATTTCCTGAACATTTTGAAGGCTCCCTTCACATCAGACTCTGTTCACAAGAAG**TGA**
>BpeXIP2-like
VWRASLAELLATAIFVFVVDTIVISSFETKMESPKLIMSLLIAIITAILLLAANPVSGGHINPVITLVAALVGLI
SVSRAAVYILAQCAGAVLGALALKAVVNNTIEDTFSLGGCTLTVIAPGPNGPISVGLGTSQGLWLEIICTFVLLF
ASVWIALDHRQARALGKVIIFSVIGIVVGLLVFVSTTVTGAKGYAGAGMNPARCLGPAIVRGGHLWNGHWVFWAG
PSIASVAFYLYTKIIPSQHFHAQGYKHDFLNILKAPFTSDSVHKK*

***Bpe*XIP1;3**

>**FXXK01002938.1:**28824-31870 Betula pendula genome assembly, contig: Contig2937, whole genome shotgun sequence
>**Bpev01.c2937.g0004.m0001**
Contig2937 27,828-30,813 (1) :: join(27828..27953,30022..30813)

>BpeXIP1;3
ATTTGATCACAGTATGAATATTTGTTCTAAGATAACCATCCTTGCTTATTTCGTCATTAGAAAATGATCAGTTGG
ATAAAGCATATTACTCAATATATGCTTTCATTACTTAACAAAAAATATATATATGCTTTCACTGTCTTCTGGGTA
ACGCTAGAAACATCATTGACACTTCCAAAAACATCAGTGGAAGCAGCCAAGTTTGGCGGCGGCAGTATTCTCTTC
TATAAGTTTCAATAGAAAATTCTATAAGAGTCTAATTAATAGGTTTTTTACTATTAATATCACCCGACAACAGCC
AGAATGCTCTCAATATTGGTAGTACTCATGGTCAAAATTTTAATTTTAATTTTTTTTTTTTTTTAAAAAAATGAC
CCACGACTGGGTCACGATAGAACAGACCCACAGCGGCATGCCCTCTCACTACAGCTAGCTAGATGCACCATTCTC
CCCAATTATTGTACGTAGACATTGCTAGCCTCTTTCCCACCATATCTTACTTAGGCCGCTGATTACACTGAACCC

AACTTCATAAAGAACATACTCCCACGTACGAATGCTAGAAAGTTGGATCGATCTAAGGCTTTGGGGGCATAAACC
GTTTGGCTTGTGGGACAATTCTCAACAGTATATGCATGTGATGTCAGGATTTTTAAGACTTTTTAAGGGAAAATG
CATATGTGCTTTCCATTGTAATTAATATGCGTTTGAATTTATCCCAAGTCCAACAACTTGAGAGAAAGTTGATGC
GGTGGTATAAAAACAGCGGAGCATCCTAAAACCAAACATATACATCAGCCTAGTGGGATTCCATCATATAGACAA
CGAAATTCTCCTCAGATTCTAACATCAAAATTATAGTAATAAAGTTGCTCAAGCTGGAGCTGTCTTTTTCGCCCT
CATATTTCTAGAAGTAATTAATATGTTGCCAGCCCCTCTTTATGTTCACGTGTCAGGTTTGGATTGTATTAAAAT
ATATATATGTTAACTATAATCCAATTAATTTAATTAAATGGGTCAAATCTCTCAACTCTAATCCGTTAATTTTGT
ATTGAATTCATGTTGTGTAAAAAATTGTCAACCCTAAATATCACGTGTCAGGTTTGGGTCGTGTTGAGACATATA
TATAACATTATATAGATCGATTAATCTTAACCCATTTCATTTAATTAAACAGATCAGATCCTTTAACCATAACTC
ATTAATTTTATGTTGAGTTTATGAATCTTATCAAAAAATTGTTAGCCGTAGTACTCCCTGGCCTCCTATATATCT
TTCAATCTTTTTGGCACAATTCTACGGAAATGAACCACGTACGTAAGAAGTTCCATCTAATAGTCTATATATGGA
ATAAAGTCACAATCAAACTTTATATAGGAGCATTTAAAGGAATCTTAACCATTCCATTTTATACAAGAGGTTATC
ATGCACAAGGAGTTTTAAGATAATTTTTGTATTCAAGCATCAAATTGATATAAACGTAAACCTCAGAAACTAATT
TAATAATCTTCCCTTCATATTGTTGTAATATTTACGATATCAAGGTGCAGCAGTGTAGATATTTGGCCTCACATT
TGGTTATTAGTAACTGCATTAATTTATTTAATCCTTGTTTATGTAAAATGAATATTCTTGCAAGCATATATTTGA
TCATAGTATGAATTGAAGTATTCTAAGACAGACATCCTAGCTTGTTTATGTAGAATGAATATGCTTGCAAATTGC
AATAGACTTCCACGCCTCATTGCAGCCATTAAAAAATCAATTGGATAAAGCGTATAACTCAATATATATGCTTTC
AACGTCTTCTGGGTAACGCCAGAAACATCACGGCCACTTCAAAAAAAAATTCTGTCGGAGCAGCCAAGTTTGGTG
TTGGCTGGATTTTTCCCATTCTCTTCTATAAGTTTCCATGAAATTAGATTCTTTGAATTGGTGGTAATTCTCTTC
ACTTTCTTTACAACTTATAAGGTC**ATG**GATATTGTTGTCCCCGGACAAGGCGATCAGGAATTTCCAACATCATTT
GAGAAATGCGAAGCACAAAATTTTAGAACAAAATTTCTTGTTTCCGTAGGTGCCCATGAGTATTTCTCCCCAGAG
GTATGCTTATTTATTTATTGTGTTCAGATATAGTAGTTAGGTCCTTCCCGTTCAATTTCTCACAAATACTATTCC
AATTTGCTCATGGGCTATCGAGTTGTAAAAAAAAAAAAATAGTAATAAAGTTGTTAAACATGATTTGCCTCCCTT
AATTTTATACATTCTATGTGGAACAGAAATAGATTTTAGAGAAATGATCTTTTTACATCTCTTTTTTAAATCAAC
CATTAGATTTATGAGTCATACGAATTCAATAATTGATTTAAAATAGAGATGTACATGAGATGTATAAGAGATTAT
GTAACATGCGTGTCTCTAGATTTTAGGAGAAACTACATTTACCTCTCAAACTACCATCAAATTTACAATAACCTC
CAAACTTCAAAAGGTTGCAAGGTATACTACCAATAACTTAAGTTTTTTTTTCCCTTTGTTGGTATTATTCTAATG
GAGGGATTGTTGCCTAGATAATCAATCCTAGAGAGAAAGGAGGGGGGGGGGTGGATAGGGTTGATAACAAACTTT
TTTTTTAATAAAAATTATATTGAATTAAGAATTTAGAATAATATAAGAAACAGTATAATGCAGAAAATATAAATA
TGAACACAATAATAAAAAGATAGGAAGAGATTCAAACTCTGTGATTTATCGCGATTTGGTCTACATGACCTACGT
CCACTCCCCAAGCACACCACTTGAGATTTCAATCCACTAATCCAAACTCCTTGCAGGTAGAGTTCAAACCTTTAC
ACACTAAGAGTACACCACTCTTCTTCACAAGGTGAAAAATCTCCTTCATACCTCACAAGGGTCACACCAACCCTC
TTGACACAATTAACTGTGGATAGAATTTGAGATTTCTTTCACAATGACAATTCTCCTTTTTGAAAACGATATACA
AATGAAGCTTTTACAATAATATCTCTCACTAGAACTTTTGTATGAATTGAACTTGAAGGGCTCAAAAAAAATTGA
ATAGATGAAAAGAAAGAATGCTTTGTGTTCTAAGCTTGTTTGTTTCTCACTTGATTGATTGTAAGAAATGAGAGC
TAAGGTTACGTATTTATAAGTCTAGCAAGAATATAACCGTTGGAGAGAAGTTTAAATTTTTAAAGTTGCAATCTC
ATTTTCAAAATCAGAAATTGTTGCTAGCTAATTTTTTAAAGTTACAATCAGATTTTTAAAGCCAGAAATTGTTAC
TAGCTGATTTTTCAAAGTTGCAATCAGATTTTCAGATATAATAATTATTGCTAGCATATTTTTCAAAGAAACCTA
AGTCCAATGCATTTTCGTATTTTAAAAACTCATGTATGAAATGTATGAAGGGTCCTAAGGTCATTCTAGAGTAAG
GAGCCTCAAGAGTTTAATCATATAGGAGTTTTACAAGAATACCTTAATAAAATAAATATTCTTTAATCTTGAGTT
CTTTTGTACTTAAGGACTTCTTGCTTTGAATTCTAAATGCCTTTGATTTTTTTGGTCCTTTGAGGATGTATGCTT
GAATGTTTCTTGCAACTATCATACTTGAATTAAATCCATTAGAACAACAAGATAGAATTTGTTATCATAAAAAAT
ATTTGCAACATAATTGGATTGACGCCATAGGGCTAACAGGGATAACTAAAAGGGTTTGGTCGATTGGTAGGGTAC
ATTGTAACTTTTTTAATTTTGGATGAATATTATAAATTAGCTAGTAGTTTGAGGAAGATAAGTGTAGTTTTCTCT
CTACTTTACTATTGGTGCAAAGATCATTCATTCAAATATAATCTCTACAATTATATTTCTTATCTCTAATTGTTT
CACTTATGTTATGCCAATACTTAAAGTGCCTGATAGAGAAAATATATAGCAACCTCCTAAACAACTACAACATTT
TCATTTACACCCCCAAACTTTAAAAAGTGACATCAAGGCCCTTTGAACTACTACCGAATGTCAAATTAAACATTT
TTGCATTTTTCTTCCCAAAATACCTTTGAGGTTACTAGAAATAAAACAATTTAAAAAAAAAATTAAGGGGCTTAAT
ATGAAAAGAGCTAACATGTTTTGATCTTGCTTGTTTCTTTCAGATGTGGAGGGGAGCCCTCACCGAGTTGGTAGC
AACTGCCTTTCTTATGTTCACCCTCACCTCTTCCATCATTGCATGCTTGGACTCACACGAGGTCGATCCCAAGCT
TCTTGTCCCCCTCGCCGTCTTCATTATAGCCTTCCTATTCCTCATGGCCACAGTTCCTTTATCTGGCGGCCACAT
GAGCCCCATCTTCACATTCATAGCCTGCCTCAAGGGTCTCATAACTCTTGCCCGCGCTTCCATGTACGTCTTGGC
GCAATGTCTTGGCTCAATAATCGGTTTTTTTATAATAAAAAATGTGATGAACCACCAAACAGGGCACAAGTATTT
CTTAGGCGGATGCACAATATCTACACTAAGCCGAGGGACTGCATTGGTTTTGGAATTTTGTTGCACATTTGTGGT
GCTGTTTGTTGGTGTGACGGTGGCGTTTGACAAGAAAAGGTGCAAGGAACTTGGGGTAGCAGTGGTGTGTGTTGT
GGTGGCAGGGGCCATGGCACTGGCTGTGTTTGTGTCCATAACTGTTACTGGGCAGGCTGGTTATGCAGGTGTAGG
CTTGAACCCTGCAAGGTGCTTGGGCCCTGCGTTGTTGCAAGGAGGGGTGTTGTGGGATGGGCATTGGATTTTCTG
GGTTGGCCCCTTTTTGGCTTGCATTGTTTATTATGGTTACTCTATGAACTTGCCTAAGGAGGGTTTGGTAGGGGT
AGAGGGGGAGTATGACATTTCCAGGTTAGTTGGGGCTTGTTTTGGGGAAAATGGCAGTCCAACTTCTCCTGAAGG
AAAATTT**TAG**GTAATGTGTTTGGTTTGCTCCAATTTCAATAGATCATGTAGTTACCATGTGAAATGACTAGGGGT
GTATATGTGGACGCGGTTGCAGTTATTGGCAATATTCGCATCCGCATAATCTAGTTATTAATTTTAGGTATCCGC
ATCTGAATCATGTAATTACTATCTGCATCACATGGTTATTGCAGTTATTATCCACTATCCGCATATGAGGTGATA
AGCCTTTTTAGACTTTTAAGTTGTAATTCCTGCTTCTAATAAATACAAAAAGGCATTTAGTTTTTTCTTTTTTA

GTAAATAATCAATGCAATAGCAAGAGATAAGTACTAGATATTGTCGATAGGTTCCATTTCTTTCTATATTGCGAG
TGATAAAACTAAATTTATGGTCTAGACGTGTTTTTTTTTTGTTAGTAGTAAAATGTGCAAATTGTATGAACAATA
TGGACAAAATGTACATAAAGATATTTTGGAAGGAATAAATAATTCTTACATGATGCCTAAAAGCAAACTATACAT
AAAAATTTTATATTTCTAGAAATCTTATCAGATTCCTAATTGTTGAAGCAATATTCTGATCATTTTTCTCCATGA
GTCAACGATCTTAATAGTCAATGTATGGTTGGACTAAAGAGATGAAATACCTGCAAAATAAGAAGATTGTTAAAG
AGTTCTCAACCGGAGCTGAAGATACTCTAATGATTAAGTCAATACAGCTCATATTTGGAGTGTAAGAATGGCTTG
GTTATGGAGTGGAACAAGCCTGTGTGTCAGAGAACATAAGAAAGAGAGAGAGAGAGAGGGAGAGAAGGAAAGCTC
TTGTGGAGAGCTCCTGAGAGAAAGAGGAAGAGAGAGAGAGAGAGA
>BpeXIP1;3
ATGGATATTGTTGTCCCCGGACAAGGCGATCAGGAATTTCCAACATCATTTGAGAAATGCGAAGCACAAAATTTT
AGAACAAAATTTCTTGTTTCCGTAGGTGCCCATGAGTATTTCTCCCCAGAGATGTGGAGGGGAGCCCTCACCGAG
TTGGTAGCAACTGCCTTTCTTATGTTCACCCTCACCTCTTCCATCATTGCATGCTTGGACTCACACGAGGTCGAT
CCCAAGCTTCTTGTCCCCCTCGCCGTCTTCATTATAGCCTTCCTATTCCTCATGGCCACAGTTCCTTTATCTGGC
GGCCACATGAGCCCCATCTTCACATTCATAGCCTGCCTCAAGGGTCTCATAACTCTTGCCCGCGCTTCCATGTAC
GTCTTGGCGCAATGTCTTGGCTCAATAATCGGTTTTTTTATAATAAAAAATGTGATGAACCACCAAACAGGGCAC
AAGTATTTCTTAGGCGGATGCACAATATCTACACTAAGCCGAGGGACTGCATTGGTTTTGGAATTTTGTTGCACA
TTTGTGGTGCTGTTTGTTGGTGTGACGGTGGCGTTTGACAAGAAAAGGTGCAAGGAACTTGGGGTAGCAGTGGTG
TGTGTTGTGGTGGCAGGGGCCATGGCACTGGCTGTGTTTGTGTCCATAACTGTTACTGGGCAGGCTGGTTATGCA
GGTGTAGGCTTGAACCCTGCAAGGTGCTTGGGCCCTGCGTTGTTGCAAGGAGGGGTGTTGTGGGATGGGCATTGG
ATTTTCTGGGTTGGCCCCTTTTTGGCTTGCATTGTTTATTATGGTTACTCTATGAACTTGCCTAAGGAGGGTTTG
GTAGGGGTAGAGGGGGAGTATGACATTTCCAGGTTAGTTGGGGCTTGTTTTGGGGAAAATGGCAGTCCAACTTCT
CCTGAAGGAAAATTT**TAG**
>BpeXIP1;3
MDIVVPGQGDQEFPTSFEKCEAQNFRTKFLVSVGAHEYFSPEMWRGALTELVATAFLMFTLTSSIIACLDSHEVD
PKLLVPLAVFIIAFLFLMATVPLSGGHMSPIFTFIACLKGLITLARASMYVLAQCLGSIIGFFIIKNVMNHQTGH
KYFLGGCTISTLSRGTALVLEFCCTFVVLFVGVTVAFDKKRCKELGVAVVCVVVAGAMALAVFVSITVTGQAGYA
GVGLNPARCLGPALLQGGVLWDGHWIFWVGPFLACIVYYGYSMNLPKEGLVGVEGEYDISRLVGACFGENGSPTS
PEGKF*

NIP – Nodulin-26 like intrinsic proteins

*Bpe*NIP1;1

>**FXXK01000146.1:**160668-172878 *Betula pendula* genome assembly, contig: Contig145, whole genome shotgun sequence
>**Bpev01.c0145.g0009.m0001**
Chr *Bpe*_Chr2 8,012,612-8,018,807 (1) ::
join(8012612..8012740,8013083..8013304,8016009..8016206,8016467..8016528,80
18606..8018807)

>*Bpe*NIP1;1
TCTGAACTGTTCATTTAAAATATATGTTCCAGCCTAATGGTTGGAAGAATAGGGGAAATGGGTTACTAAAGCCAA
ATTAAAAAAGGAAAAGAATGAAGTTTGTGGGTGGTTGGTAGTAGTTTAGTTACATCCAAATAATTGGTTTTGAGT
GATCGAATCACCCCTTTGCCAATTTTGAGTGGCCAAATTACCTCATTAATTATGAAAGTGATTTGGCTACTTCCA
ATTGTTGGTTTGAAGGTGGCCAAATCACCTCTTAGTCACAAGAGATGGTTCAACTCTTATATATATACATTGTCA
TAATCTAAACTACTCATTTAAAATACGTATCTTGACCCAAGGGTTTGGAGAATAAGGGAAGGGGTTGTTGTTACT
TAATGAATTGTCTAATTAAGGTTCTATTTGTTTCGTTATAAAATGATTTATGAAAACTATTTTTTGGTGTTTTGT
GCGACAGAAAATAATAGTCAAATCGTAAACATTTTCAATTTCATTATAAAAGCTTATTTAATTTTCGTAAAATGA
TTTCATTTTTTTTAAACCATAAACTATTTTCTAAATTTGAATTTTTCTTTATCAAAACACCAGAGTTCGCCGAAC
TATCACCAGACCCTTGTCGAACTTCGGCTAGAAGTTGCTAAAAGGTGCCAGTCATTTTTCTCCATCGCTCATTTT
TCATATGAGGCAAACGATGAAAACTATTTTTTAAAAAAATAATTTTGTTAAAAATATATTTCTATAAAAAACATT
TTAGATTGAAATAAAAGAAAGCCTAAGCCATAAAGGGCCAAAAAAAAAAAAAAAAAGTCAAAAGGTAGTAATGATG
AGGAAGAAAAAATACGTGTTCCCAAATCTATCATATGTTTCACAATTTTTTTGTGAACCAAATTCAGTATACTCA
TGTTGAACAGCTTAGGATACCATGCCGACAGGGGTTTGATAAAGGCTCCTGGCAAATTTAATTAGGTCCCTCACA
AGCAATTTATCCATATGAACAAGCATCCCCTTTTCTCATCTCCCATGTAAACAAAAGAATACGCAGAAAGCTAAA
TGTTTTTTTTTTGTGTCACTCAAATAATTATATAACTTTTTAAATTATAATTGAATTAAAATTTAATAAGGATCT
ATTACAAATTTGTGTGAACGATTAAGAGAAACTTTGGGTGTGTTTGGTAAACTCTTTATTTTTTTTTATTTTTTG
AATAGTAATAAAAAATTATTGATGTAATATAAAGTAAAAAGAATGTTGTGAATTTTTTTACAGGAAAAAATGAAA

ATAATTATGTTTTTTAGTAAGTTTTTTATTTGAATAGTAATAAAGAGTTATTGATGTGATATAAAAAGTGAAAAA
AAAAAAATTGTTAAAAAGTGATTAATAGAATATAAAAAGTGAAGTTGATTGTTTTTAGAAAAATAAGAAAATGAGA
GGAAAAAATGTTTGCCAAACCAGGCCATGTCATTTTTAAGTGACACAAAAATATATTTAACATTACTCAAAGAATA
TACATTATTGAATAAAGTTAATGAATGAAACAAATGTCCTTTTGGTAAAGAGTTTTATTGTGAGTTTTTTTGTTT
GAATATTGATAAAAAGTTATTGATGTAATATAAAGGCGAAATGAGATTTGAATATTTTGTGAGATAAAAAGCGAA
AAAACTATTTGGTAATAGGAAAAAAAAAATAAATTGACTTATTTTGAGAAAAATGAAAATAAGGGAATAGTGTGTT
GCCAAACACACCCTATAAAGACTCTTGAGGTGTAGAAATTTAGTACAGTTATAATTACTATCAAATGACATTTAT
CACGTTAACTATTAAATAATTAAATTTACTTGTTAAATTTTATTATTTAATTTTTTTAACTCACGTGACATGATT
GTCAAGTAAGTTCATAAAAAATATTATAATAAAAAATTGTAATACTTCAAATATTTCTCACGAAATAGGCCACACTA
CAATACAATAAAAGTTAAGACACCAAATTTTGTACATGTGAGGCATTTGGCATGCATTAATTAGCAAATTATTGT
CCATTGTTTTTCGTATCATTTTAAAATTTTCCATAATTTAGTTCTGACTGAACTTTTTCAATATTAATTGTCAAA
AAAAAAACTTTTTCAATATTAGTAATGTGAAAGCGACAATATTATTATTATTTTTTTGAAAGGCAACAATTAATC
TATGTAAAATGGGTCACTAAAATAAGTCGAAGAAAAAGTATACATATTCTTTTCAAACGAGTATTTAATTTGCAA
TGTTCATTTTAAACTATCAATTGCGCCAACATCTTCTAAACTCTAAAACTTTGCAATGTATATATTTTATACCTT
AAAAAGATAAAAATGCCATAAAACATTTTCAGAAGACAAATGCTCTTATAAATTCGAAAAAAAAAAAACCCACAA
TGGTAGAAAAACATACGGTGTGGTAAAAAAAAAATGTCATAAAAAAAAAAAAAACACTTCACAAGCAATATTTTAA
AGTAATGCTATATTCGATATTTTATCTTATAATTATCATCAATAATAACATGACACAATTAACTAGCCATTGTAT
TAATCATTATTAAATAAAGAAAATATATGATTGTTGATTTAGAGTTCTACGCCATTATTAACATGAATGTTACTA
ACATGTTTAGGCCCATCAAGCAAAAAGGACCCATCCTTTCAGGATTTAAAGGTGAATCCGTTAAGATAAAGAAGG
CAACAAAGTAAGACCAAAAATCAACAAGAGTGCATCTTAAATCCACTTGACCCACCCCACCAGCCAAGTCTCCAA
CAAATCCAAAATTAACGATTTTTTCTCAACTACATCCAATTCCAAATGACAAAACTACCTGGTCTTATCTTCAAA
ACCCCCATTCAATCACTAATTTCATGACTCTAAACCCCTCAAAGTTTTAATTCTCAGATCATAATTAATTGCTTT
TCAAAACA**ATG**GGCATAGAAGAGCTAGAAATTTCGAAGGTTGAAGAAGGTGTTGGCACATCCGCCGCCACCAAAA
CCAATTCTGATACAGTTACTGCCTTTTGTTCATCAGCTTCAGTAGTAACCATCACACAAAAGGTAAGTGAATAAA
AAAATATCAGTGCTCCTACTTCCTACTTCCTAGGATTTACTTTAAATATCTAATTAACCACCATAATACTTATAA
TAAACCATTATAGCCAACAAAATAGAAAAAGAAAAAACAAAACAAAACCATTTTAGCCAACCATCCTTGATCGAA
ACGAATGCTCTTCGGTCTTCGTACAATTTTTTTGCAAATCAACAATTAAATTTGTAGAACTCATATGAAAGATTG
TAGATGAACCGTAGAATGATTAATAGCATATCTCATTTACTTAAATATTTTATAATGAGGTTTTTGAAATAAGTG
TCTCTATTTTGCTGGTTTTTTATTTGCAGTTGATTGCAGAAGTGATTGGTACGTATTTTGTGATATTTGCTGGTT
GTGGCGCAGTCGCTGTCAATAAGATCTATGGGTCGGTCACATTTCCAGGCATATGTGTGACATGGGGTTTGATAG
TCACGGTCATGATTTATACGGTTGGTCATATTTCTGGAGCCCACTTTAATCCCGCGGTCACCATTACTTCTGCCA
TCTTTCGTCGCTTCCCTTACAAGGAGGTCAGTCTTATAAATGCTGTATTCAAATTCTTGCCTCTATTGAAATTTA
TTTATTAAACTAGTTGGAACAGCACGTGCATTGCACGTGCTTGGTTCTCTTTGTTGTATATCACTCTATAAATTA
AAAAAATATGATATACTAAAAATAATTATTACATTTTAGATAAATTATTAATGAATTTTGAATCAAATAATACTT
TTTAATTTAGATCAAAAAATGTGTATTTAAAAGTATGAAAGTGGAAGTAGGATATTTCTTTATTTCTAATTATGA
TGTGTATCATGTGTAGCCTTGTAACTTTGCTGTTGACTTTAACTTTCAAAATAAATTTGACTAACACAATGACAT
AGAAAGAAAAAAAATTGAAATGGGTCTAAATACACACCCATCAAAATCTAAATTTCAAATCATACCAATCTAACAT
TATTTTCGTAGTCTATAATTAGTAGGTATAACAAAACTAAAACTAAAACTAAAAAAAAAAAAAAAGGTAGAGTTT
TTACCTTATTACAACCTATGGCCGGAATCTTTCATTGGCTTCACCACATTCTTCTTATTATTCTTCTTCTCTTCC
TCACCATCTTCTGAAACTCCCGAGTCCCCAATACAACAAAACATTTAAGGGATGCCTTGCTTTTCTCATAGAAGT
CATTTGGCTGAATATGAAATCGTATCAAAGAGTGAGATATATTTGAAAAAATTGTGAACAAAAATTATGATTAAA
GAAGATGAACCAAACCTTATTTGCTCTTTACAGTGTTTGTAGATGGTGGAGAAAAAAATTGAACTCACATTACAT
TGTTTACATTTTTACCAGGTTTCATCTGATCTTATTTGTTTTTTTTAGTAATTTTTGAAGTTGGTTGTAAATATA
ACTGTATCCATGATTATCTAATTGTAACTGTCTTTGTTTATCTAGTTATAACTGTTTCTCTTTATCTAGTTGTAA
ATAGTAATTTTAAAATTTAAAAAAATTATGAAAAACAAAAATAACCCTGTCTGATTTAACAATGAAGAAGAAAGA
AAAAAGGTAAAAACGTAAAATTACATTATCAAATAAAATACACTATTTACGAGGCATTCACACTTTTTTATATAC
CTTTAGGTAAGGAAATCTTGACTTTTTAAAATATGTATTCATTCATTTTTGTCCTAAACTTGAGCTATTCTTCCT
GCAAATACAATAGCTCTCTTGTATACGCTATTTATTCATTTGAATATCATGATGTAAAGTCTAATCATGTATATG
ATGTTATATAATTTAAATTTTTTAAAAGATGTGTTTAAATTGACATCATAAACATCATTAAATATTTAACTTAAA
TTAATACCATAAAATAAAATAAATATTATTTATTTCATTTGAAATTAAAAAAATAATGTACCGTTAAATTGGAGC
ACATATATATATATATATATATATATATTATATATATATATATATGCTCAATAGAAAAGAATTAGACCATCCTTGAG
TATAGACTGTATAGTCCTTGTTTGGGCTCCATCTCTTCAAGTTGACTGGGCTGGTAGGTTTTTGGTTTGGACCAT
ACGAAACCCACCAAGGTTTTCCATTGACCCTAAAAACTCATAGGTATAGAAATTTTTTTTAAACCCGGTTTAAAG
GAAATTCTTTTAACCAATTATCTAAAAGTGTTACGCGTCTTTTAACAGGTGAGAATGACATGTTTTTTTAATAAT
ATGTGCTTTTCAAATATTTTGGCCTCAAAAAGTCACCTATCTTATCCACCAATTTTGCAGTGAGAAATTGTTGAA
AAAATATAAACATAAGAACGTGAAATTCAATGATATATACCACTTGAGAACTCGCGAACTATGATCTTTTGTAAG
TGTTCTACGCTATTTATTGATGCCTCTCATATTTGTAATGTTGAAAGAACTTAAACGGTGATGTTTTATTACAAG
AACAAAACAAATAAGCCTACAACCTAAATGCAATTTGAGAGAAAAAGATAAGAGTTAATTGAGAGAAATTTAGAA
TTTCGTTACAACTGGAAATTGGAAAAGGCCTTCTTCATGGCCTCTAAAAATGATTAAATGAATTAATACCCATCC
AATGGGTTATTTATAAACAACCAAGAGTTGTGAATCAAGAGGTACAACTCTTGGTAATGGTTGGACCAACATAGT
GTGAGTATACTAAGGTCCAATTACTACCATAAACAAATATATTTTTCTGTAAATTTGATACCTAGACAAGATTAA
GAACAATTATGTCACAAGGGATAATAATATTTGTTGATTAATATCTACTTTTTCGCCCAAGCATAAAAGGATAAG
GAAACAATGAGAGACTGGTCCACCTAGAAGTAACCCTAAGCCTAATAGCACCTTATTCGCTAGGGTATGAAGTAA

TCATAATAGTACTTTATTTGATAGGGCACCAACCAATGGACCTGTAACTTGGGAGATTCATAGGCCCTCCTTAGA
ATTGATCAAGTCATAACTTAATTCCAACCCCACTAGAGATATAATGATACGAACAAAAATTAGTGGGCATGAGTC
GTCCCATTAATACGTGTCCATGGAAATTGAACTCATATTTTTTGTGAGCTCCTGTTTGTTCTGTTGCTCATATAA
TTCTCCGCCTTACCAAACCATAATGGGAGTAAGCCTCTTACCATTTCGTTAAACTCTTTTGATGATTTGTTGATT
AAACTCAATTATTACTCCCATAAACTGCAGGTTCCTCTGTACATAGCGGCTCAGCTAATGGGATCAATTCTTGCT
AGTGGCACATTAGCTCTTGTGTTTGATGTAACTCCTGATGCTTACTTTGGAACTGTACCTACTGGATCAAACTCC
CAATCCTTGGTTCTCGAAATTCTCTGCTCCTTCCTTTTGATGTTTGTCATCTCCGGCGTCACCACAGATAGTAGA
GCAGTATGTAATCCAAATATGCCTTGGAATTTTATATTTGCAAAAAGAAAAAAAAAAAAGGAGTACATTTTCA
ATTTTTAGGCTACAACACATGAGCGCAAGTAATTCAAAATGGGCTTCTGATGGGCCATTGCTTTTTTTTTTGGCA
CTTTTTCTATTTAAACAAATAAAGGATGCTACTTGTGAAATTCGTGATATTTGTGACAATATTTTTGGTTTGTAT
GTCAATAATCCCATTCAATATTTTTTTTTTTTGGACAGATAAGTGAAGTAGCAGGGATTGCTGTTGGTATGACCA
TATTATTAAACGTCTTCATTGCTGGGTAACTCTCTATTTTGTGATAATGTTTGGGTACCAGTTTTATTTAGACTG
ATGTAGCATATTATGTGGATGGGTCCAAGTAATTAAATGATGTGTTGCATTTGCATATCAGAATGTCACAAAACT
TTTTAAATGATAAAGATTTTCCTCGACATAAAATAAGGTCCCATGATATTTAATTAGAAAGGACTCACACTACAA
TTTAGATTTTATGTTTTTTTTTTCCTAGTTTATAAACTATACTATCCATTATGTAACAAAAGAAAAAAAAAAGGG
ATAAAACTTGGACCAAAACCATACGAAAGAGGATGTAATTTCTTACAAGGAAACCCATTCTTTATTATTATTTTT
TTTTTTGAGCAATACAAGGTAAGGGAGGGCCATTCTTGATTCTTTTATCGGTAAAATTGTCTACTCTATAACAAG
GTCCATGTGGACATATCATAAAATTTTCATCTTAGACTTATATTATAAGTATCACATTAGTTTGTAAACCTAGCA
AATGTCACGTGACAATCAACATTTTAGTAATTGATATGATAAGTATCATGTGGACTGTCACGTCAGTTTGTAAAA
ACTTATAATAAAAGTTGTAACACCTATAACAATTCTTCGTTTGGCAATCACTAAAAACGTTAAAAAGAAAAGAAA
AAGGCAATTGCCATTCAATTTACAATTTCTGAAAGCTCTTTTTTTTTTGGGGAAATTTCACAAACCCCCCCTGAA
TTGTCATTGCTTTTGCAATCCCCCCCTCAAACTTCAAAACCTCTCAATTTAGTGTATTTAAGTTTCAATTTTTTG
CAATCCCCCCCCTCCGTTAGGATTTTTCGTTAAATCCTAACTGAAGGGTGTTAAATTACCAAATTATCCTTAATA
TTTTCAATTTTCAAATTTTTTTTTTTTTAAAAAAAGTGACCCACGTTGGGTCACCTGACCCAACGTGGGTCAGCC
GCGACCCACGCAGCGTGGGCCAACCGTGACCACGCTGGGTCACGTTGTGACCCAGCCGTGGGTCACCAGACCCAC
GGCGGGTCTGGTTGTGACCCCGCCGTGGGTCGGTGACCCACGGCTGGGTCACGGCGTGACCAACGCTGGGTCACG
GCGTGACCAACGCTGGTCACGGCGTGACCCAACGCTGGGTCACGGCGTGACCCAACGCTGGGTCACGGCGTGACC
CAACGCTGGGTCACAACGTGACCCAACGCGGGTCGCGACGTGACCCAAGCTGGGTCACGCCGTGACCCAGCCGTG
GTTACCAGACCCACGGCGGGGTCACCACCAGACCCCGCCGTGGGTCTAGTGACCCACGGCTGGGTCACAACGTGA
CCCAGCTTGGGTCACGTCGTGACCCGCGTTGGGTCACGTCGTGACCCGCGTTGGGTCACGTTGTGACCCAGCGTT
GGGTCACGCCGTGACCCAGCCGTGGGTCACCAGACCCACGGCGGGGTCTGGTTGTGACCCCGCCGTGGGTCTGGT
GACCCACGGGCTGGGTCACGGCGTGACCCAACGCTGGGTCACGGCGTGACCCAACGCTGGGTCACAACGTGACCC
AACGCGGGTCACGTTGTGATTGTGACCCAAGCTGGGTCACGCCGTGACCCAGCCGTGGGTCACCAGACCCACGGC
GGGTCTGGTTGTGACCCCGCCGTGGGTCTGGTGACCCAGGCTGGGTCACGGCGTGACCCAACGCTGGGTCACAAT
CACAACGTGACCGCGTTGGGTCACGTTGTGACCCAGCGTGGGTCACGGGTGACCCACGGCTGGGTCACCCGTGAC
CCAGGGTCTGACCCAAACAATTTTTTTTTTTTAATAATTTTTTATTTGAAATAAAGGTAATTGTAATTTTTTAAAA
AGTTAGGGGCATAAAAGTTATTTCACCCCTCTTGATCGTCTATCTTAACAGAATTGACTAACGGAGGGGGGATTG
CAAAAAATTGAAACTTAAATACACTAAATTGAGAGGTTTTGAAGTTTGAGGGGGGGATTGCAAAAGGCATGACAA
TTCAGGGGAGGTTTGTGAAATTTCCCCTTTTTTTTTTTGGTGTATGATGGTAACACAGTTTAGAATGAATAATTGC
AGGCCGGTTTCAGGAGCTTCAATGAATCCCGCAAGGAGCATACGGCCTGCTGTTATAAAGCATGTGTACAAAGGA
TTGTGGGTGTATATAGTAGGGCCAATTATTGGAACAATTGCTGGAGCATTTGCCTATAATCTCATCAGATCCACA
GACAAACCACTTCAACACTTGCCCCGGAGTACTGGATCATTCCTGAGACTC**TGA**GAGCTACTACGTACAACTAAA
TTATTGCAGGCTACTTCCAGGACCCCCCTCTATTTCCCGCAAGAACCATTTTGTTATCTTTGTGAGTTAACCCAA
GACCCTGCAAATCGTTTACAACAATTACTGTTTTTTTTTTTTTTTTTTTAGATTTTGATTACTAATCTTTCTT
CATATAGAAGCTTCTGCTGGTGCTTTTTTTGAGATGAGAACAAATTTATTTGTAGAAGTTTGTAATAGGGGAAAA
ATTACTTTTTACCCCCTAATGTTTGGGGCGATTTTCAATTTAGCCCCCAATGTTAAAAAATTCACAATGTGCCCC
CTCAATGTTTAAAAAAAATCAATTCGGCCATTCCGTTACCTAAAACCGTCTAACTTGCCGGATTTACTGTTCATT
CGAATTTTTTATTATTTTTTTTTACTGTTCATGGTTAACCGTCTAATTTTTTTTTTTTAATTTTTTAAATGGGTAA
ACCGTAAACAGTAAAAAAAATAAAAAATTCGAATGAACAGTAATCCGGCAAGTTAGACGGTTTTAGGTAACGGAA
TGGCCGAATTGATTTTTTTTAAACATTGAGGGGCACATTGTGAATTTTTTAACATTGGAGGCTAAATTGAAATCG
CCCCAAACATTGGGGGGTAAATTATAATTTTCCCTTTGTAATAATTATATTTAGTCATTACAGAATAAACGGGAT

>*Bpe*NIP1;1

ATGGGCATAGAAGAGCTAGAAATTTCGAAGGTTGAAGAAGGTGTTGGCACATCCGCCGCCACCAAAACCAATTCT
GATACAGTTACTGCCTTTTGTTCATCAGCTTCAGTAGTAACCATCACACAAAAGTTGATTGCAGAAGTGATTGGT
ACGTATTTTGTGATATTTGCTGGTTGTGGCGCAGTCGCTGTCAATAAGATCTATGGGTCGGTCACATTTCCAGGC
ATATGTGTGACATGGGGTTTGATAGTCACGGTCATGATTTATACGGTTGGTCATATTTCTGGAGCCCACTTTAAT
CCCGCGGTCACCATTACTTCTGCCATCTTTCGTCGCTTCCCTTACAAGGAGGTTCCTCTGTACATAGCGGCTCAG
CTAATGGGATCAATTCTTGCTAGTGGCACATTAGCTCTTGTGTTTGATGTAACTCCTGATGCTTACTTTGGAACT
GTACCTACTGGATCAAACTCCCAATCCTTGGTTCTCGAAATTCTCTGCTCCTTCCTTTTGATGTTTGTCATCTCC
GGCGTCACCACAGATAGTAGAGCAATAAGTGAAGTAGCAGGGATTGCTGTTGGTATGACCATATTATTAAACGTC
TTCATTGCTGGGCCGGTTTCAGGAGCTTCAATGAATCCCGCAAGGAGCATACGGCCTGCTGTTATAAAGCATGTG
TACAAAGGATTGTGGGTGTATATAGTAGGGCCAATTATTGGAACAATTGCTGGAGCATTTGCCTATAATCTCATC
AGATCCACAGACAAACCACTTCAACACTTGCCCCGGAGTACTGGATCATTCCTGAGACTC**TGA**

>*Bpe*NIP1;1
MGIEELEISKVEEGVGTSAATKTNSDTVTAFCSSASVVTITQKLIAEVIGTYFVIFAGCGAVAVNKIYGSVTFPG
ICVTWGLIVTVMIYTVGHISGAHFNPAVTITSAIFRRFPYKEVPLYIAAQLMGSILASGTLALVFDVTPDAYFGT
VPTGSNSQSLVLEILCSFLLMFVISGVTTDSRAISEVAGIAVGMTILLNVFIAGPVSGASMNPARSIRPAVIKHV
YKGLWVYIVGPIIGTIAGAFAYNLIRSTDKPLQHLPRSTGSFLRL*

*Bpe*NIP1;2

>**FXXK01000231.1:**15000-21500 *Betula pendula* genome assembly, contig: Contig230, whole genome shotgun sequence
>**Bpev01.c0230.g0002.m0001**
Chr Contig230 17,343-19,985 (-1) ::
complement(join(17343..17562,18062..18123,18938..19135,19480..19704,19842..19985))

>*Bpe*NIP1;2
GTATTTTTAAAGCATGTACATTTATTTTTAATAACATTAATAAAAAATATGTGTTTTTCACATGCTTTAAGAACA
CATGTTATTTTACTAGATTAGTTTGTAAGAATTATCTACAACTTAGTTTATAGCTAATTTATGTCCTTAGCAAAT
AGGATTTTTTTAAAAAAAAACAAAAAAAACTTGTTGGTCTTGTTAAAAGTAGAATTCATGTATTGCTATCCTCAT
AAAATATTAAGATTAGAACATTTTTATCCGGCTATTTAAAGTAGGAATTTTCTTATTTTAGAGAGTCAAAATTTA
TTTTAGACCATTTTGTTGCTTTTATTCGGCTCCCTACTTTGAAAAAAAAAGTAAGGAACATGAAATGCCATTTCA
CTTTCCACTCTTCTTTAAACTAATGAAATATTAATTTTTCTTTTTAACTGTTTGCTTTTTCCAATTTTTTTTCTC
TTCTTATTTTTTAAAAATATTATATTTAAATAAAAAAATGTGAAAGTATACCTTTAATTTAAAAAGTCAAATGTA
GTGATTAAAAAATAAAGTATTTAAAATAAAAAAAATATAAAGAACCGAATAAAAATGTTTACTCAAATCCTCAAC
TAAGATTAATCTTAGGCTGAAGTCTTCCCTTAAGAAGGCAAACTCCAATAGCATTAGACTAACGGTAAACACCTC
ATGGGTACCCGTCCACGCAGGATCTATAATAGTGTTTAGGGCACTTTTTACAAGGCATTCATGTCCCTCTGTATT
TCCGGTGGGTTGTTGAAGCAGCCATCAATGCCTTTCCCTGTTCACATCCTCTAAACATCTCTTTTTTCAACGAAC
GGTATTTACTATGCTTTTCATATTTTAAAATTAAAACAAACACATTTGGTCGTCTCTTTCTTTATCCCAAAAGCC
CATTCTCTCTCTTTCTCTCTCCATCCTTTTTGGCCCAATAAATTGGAGTTGTTCAGCTCGGTTCTTTGCCAAAAC
AGAGCAATCTCCTTTCTCTGATTTTTCACCTCTGTTCCTTGTCTCTTACCTTCTTTTCGTGTCAAGAGCG**ATG**GC
AGAGAATTCCGGAAGTAATGGAAGCCAATCGATTACTTTGAATGTTAAAGAATGTGAGGCCAATCACAACCAAGC
CTCCTCTGCCTCCAAAACCAACCATGGCGGAGGCTTCTGTGTCTCTGTTCCTTTTATGCAGAAGGTTGAACCACT
CTAAACCCATCTATTTATTTGTTCTTAAATTTGAAATTTGATAAACAAATCTTATTTAAGAATGTTTTTGGGTCT
ATATATTCAGCCAATTTAACATGAAAAAGCGGAAATTTTTGTTGATGACAGTTGGTGGCAGAGGTGTTGGGGACG
TATTTCTTGATCTTTGCCGGTTGTGGGGCGGTGGTGGTGAATTTGGGGAATGACAAGGTGGTGACTGCGCCGGGA
GTATCAATAGTTTGGGGACTCGCTGTGATGGTTCTGGTTTACTCTGTTGGCCACATCTCTGGCGCTCATTTTAAT
CCTGCGGTCACCATTGCTTTTGCCACTTGCAGAAGATTTCCATGGAAGCAGGTAAACTACACCGAAGAAACTTTC
TCATGCTTGGGTGCACTGCAAGAAAGTGCATTAATTAACATACTTTTTGCAGTTTCTAAACAACCCACTTTCCTT
TCTGAGAAAAATCTTCAAACTTGTTTTAGAAGGCATGTTATTAACACCCATATCCAAATGGGTATGTAAATTCAT
GTATTTACAAAAAAATTACTTCAATTTTGGTGAATTTTCAAGGTATGATAAAAACCCATGACGCATATCATATGC
CTTCTCCCAACCAATGCACACTGTATTCCAACCAAATTTGTCTTTCTGATGTAAACTTCTTTTTTTTCTTTCTTTT
GTCAAATTTATATCTTGTAGGTGCCAGCTTATGTATCAGCTCAAGTCCTGGGATCAACAATGGCGAGCGGAACCC
TTCGGTTGATATTCAACGGGCACGAGAACCGGTTTGTAGGAACAATTCCGGCTGGGTCTAACTTGCAGTCTTTTG
CGCTTGAGTTCATCATCACATTCTACCTCATGTTTGTCATATCTGGCGTTGCAACTGACAACAGAGCTGTTAGTT
CTTCACCCTTTTATTCGTTTAATTTTGTTAAATCACATGTTATTTCAAAAAGTTAAGTTTGACACGCCATATATT
TAATTAAAATAAGTGGTGAAATATAGAGTTAGAATTTGAGCTCAGACTTCTGCTATGATATTGGTAAAATATAGA
GTCAGGATTTAAGCTCAGACTTATGCTCTAATATCGGTGAAATATAGAGTCAGAATTAGAGCTCAGACTTCTGCT
ATGATATAGGTGAAATATAGAGTCAGGATTTAAACTTTGGTGTCTTTCAGCATAGCAGTAGGGTCAGGAACATTT
TGGATCAAAGAAATGGGTGTAGAATTGACTTCTTTTAGTAATCAGCCCGAGACAAGAATTAATAAAAAAGAGATT
TCATAGCCCTTCAACACACCAAGCCTTCTTGAAAAAATAGGAGTTAAAAGTCACCCGGAGGACCAGAAGCTTTGT
TGGCCTTTTGAGAGAAAAACTCATCTCTAATTTTCTGATGCTATGATATATGTTAAATTACCAGTCATATGTTTG
AAAAGTGGTGAATTAAATTATTTAATTAACATTCTAACAAATTTTTAATTATAATTGCACAACAATTTATAATTA
GCCAATGTTATGTGGTTGGTTGATTAGTTCTCCTATCTTTATTGACCTATTGCATCGAGAATCTTATGAGATCAA
GCAATCTGTCTGATATATATATCCAAGCGTGCTGAGTTATAATACTAAGATACTTTTTAAGCTTATCTGTGCTGG
CAATTATGTATGAATTCTTCTACACCGTTGAAATTAATATATTTTCTGTCTATGCAGATTGGAGAGCTTGCTGGC
CTTGCTGTTGGGTCTACTATCCTGCTTAACGTCATGTTTGCAGGGTAATAGCTTAATCTAATCTGATTTTAATAT
ATTCAACACCGATCATCTTTACACCATTTTTTATTATTATATATATTCATGTTTGTACATATTTTAAGCAAAACG
CTTGGAATGCTACCACTTTTATTACAACTTCTTACCAACTTGGATTGGGACAAGTTGGTACTTATCTGTCCAAAT
AAATTTTGGACTGTTTTGTCATTTATCCGGACAGGTGGGTCTCTTAAAGACTTATATTTTCTAACAATCTGGACA
GCAAAATTGATGAAATCTCTATCCTACCAACTCAAGTGGCAGACTGTTACGTGGTAGTTCACAATTTAACAACTG
ACATGATAAGTGTTATTTAGACATGCACATGACCGTTCATCTTTTAGTGACTGATGCGATAAGTGTGACGTGAAC

TGCTAAATCAGTTTATAAAGAACGTGTAATAAAAATTGTAGTACCCTTTGCAACGCTGGTATTCTAACTATGTCG
GGATGCTATTTTTGACAGGCCAATTTCAGGAGCATCAATGAATCCAGCAAGAAGCCTGGGGCCTGCAATTGTGCA
CAACAAATACGAAAGCATATGGGTATACCTAGTGGCAACGACTCTTGGGGCTATAGCGGGTGGAATGGTGTACAA
TATAATCAGGTTCACGGACAAGCCGTTGCGCGAGATCACCAAGAGTGGGTCTTTCCTGAAAGCGTCAAGAAACAA
CAACTCTGTC**TAA**TTAACTATATATAATTATTATATTATGAGATAAAAAAAAAAAGGCAAAGTGTTTCCTCCACG
TTTTGTACTTGTTTGGTAATTGCTACGTAGGATGTGCTACTTTAGGCTTAAAATGTGTAAGGAGGAACCCACATG
TCTGTCTATATGTTATGATCGAATGTAGTCTCTGTATCTTAACAAACCTTTATAAATGAAAGTTTGCTTCATAGT
TATAGTTGGTGTTTGGATAAGGGTTTTTTTTTTTTTTCTTAATTTAAATTGGGTGGAAAGAAATTCTTAAAAAA
AAAAAATGCATGTGAGAACTTACTCCAAGGACAAATAGATATCTGTTTAATATATCAAATTTAGATATTTGTACC
ATGGTAGTCGTTTTTCTATTTTGTTTTGTTGTTTTCTTTGGGTTAAAGCTTTTCAGCCGGTGTTTATTTCCTTTC
TTTGTGCTGAGATGTCTCTGGTTTTGTGGGATTTTGTATCATCAATTGTTCCTTTTCATTCTTTTTAAGTGCATG
TTCTCTTGGTGTAAAACGTTTTTCTCCTTGTTGTAGTTGTTGTTTAGGTTTAGGTAGTTTGTTTCGGAGAACTTT
GCCTTTGTTAACCGCCCTTTGTGGCGGTATCAATTTTCTTGATGGGTCGGGTCATTTACTTGACCCATTCCCAAT
GGTTTTAGTTTGTTTTTGCATTGTTTGTAAGTTGTTTTGGGGACTACTATTTGGGTTGGCCACCCTTTAATTTTA
TTTTTTTTCTATGTAATTTCCTCTTTTCCTTTACCAGGATTGAATAGAAATAAAAATAAAAATAAAAATAAAAAG

>*Bpe*NIP1;2

ATGGCAGAGAATTCCGGAAGTAATGGAAGCCAATCGATTACTTTGAATGTTAAAGAATGTGAGGCCAATCACAAC
CAAGCCTCCTCTGCCTCCAAAACCAACCATGGCGGAGGCTTCTGTGTCTCTGTTCCTTTTATGCAGAAGTTGGTG
GCAGAGGTGTTGGGGACGTATTTCTTGATCTTTGCCGGTTGTGGGGCGGTGGTGGTGAATTTGGGGAATGACAAG
GTGGTGACTGCGCCGGGAGTATCAATAGTTTGGGGACTCGCTGTGATGGTTCTGGTTTACTCTGTTGGCCACATC
TCTGGCGCTCATTTTAATCCTGCGGTCACCATTGCTTTTGCCACTTGCAGAAGATTTCCATGGAAGCAGGTGCCA
GCTTATGTATCAGCTCAAGTCCTGGGATCAACAATGGCGAGCGGAACCCCTTCGGTTGATATTCAACGGGCACGAG
AACCGGTTTGTAGGAACAATTCCGGCTGGGTCTAACTTGCAGTCTTTTGCGCTTGAGTTCATCATCACATTCTAC
CTCATGTTTGTCATATCTGGCGTTGCAACTGACAACAGAGCTATTGGAGAGCTTGCTGGCCTTGCTGTTGGGTCT
ACTATCCTGCTTAACGTCATGTTTGCAGGGCCAATTTCAGGAGCATCAATGAATCCAGCAAGAAGCCTGGGGCCT
GCAATTGTGCACAACAAATACGAAAGCATATGGGTATACCTAGTGGCAACGACTCTTGGGGCTATAGCGGGTGGA
ATGGTGTACAATATAATCAGGTTCACGGACAAGCCGTTGCGCGAGATCACCAAGAGTGGGTCTTTCCTGAAAGCG
TCAAGAAACAACAACTCTGTC**TAA**

>*Bpe*NIP1;2

MAENSGSNGSQSITLNVKECEANHNQASSASKTNHGGGFCVSVPFMQKLVAEVLGTYFLIFAGCGAVVVNLGNDK
VVTAPGVSIVWGLAVMVLVYSVGHISGAHFNPAVTIAFATCRRFPWKQVPAYVSAQVLGSTMASGTLRLIFNGHE
NRFVGTIPAGSNLQSFALEFIITFYLMFVISGVATDNRAIGELAGLAVGSTILLNVMFAGPISGASMNPARSLGP
AIVHNKYESIWVYLVATTLGAIAGGMVYNIIRFTDKPLREITKSGSFLKASRNNNSV*

*Bpe*NIP2;1

>**FXXK01000282.1**:659099-670105 *Betula pendula* genome assembly, contig: Contig281, whole genome shotgun sequence

>**Bpev01.c0281.g0063.m0001**

*Bpe*_Chr2 4,593,561-4,596,959 (-1) ::
complement(join(4593561..4593786,4594005..4594066,4594153..4594347,4595128..4595352,4596819..4596959))

>*Bpe*NIP2;1

ATGAAACTATTCTGTATATTTCTATGGTGATTTAACTGTGCACAAATCAGGCATATTTATAGCTGAATGAGACCG
GTTAAGATATAAAATATAATAAACAAGAAATCAGAGGATTAAAACAAAATATCACATAACAATATGAAACTATAC
AGAGAAATAAATTAACATTTGATTCGGTCTATAAATGGCAAATGATCATAACATAATCGAATTTTAATATCAGGC
CAACAACAAACATATTCTGAATATATATTATAACACGTCACCTTAAACTCATTGTGGTAACGTAGAAGCCAACTT
AAATTTGGAAAAAAGATCATAGAAATGTCCAAGTAGATGTGCTTTGATGAACACATCAACAATTGATCAACAGAT
GCAATAAGACAAAGGCAAAGAGTTCACTACAAGAGATGATGGCGCATAAAATAATAATCGATTTCATGTGTTTGG
TGTGCTCGTAGGCACACCCATGTTCTGTAGGTGCTAGTCTAGCCAGAATAGCTCAAAGGTGATGTTAGTATGTGT
TGGTACAATACTCAGTCTCAGTACCATAGCGAGCAACAAACAATATAACACCAAGTAGAAAGCTATAAATAATGA
TGGAGTAGTGACAACCATGAACTCATGATGGAATGACAACCAATGAGATCACTTGGGCAACTAACTAGCCAATTG
ATTTGAAAGATGCATGTTTGGAAATTCACAAGCTTACCACCTCATCCAAGAGGCTAACTAGAGCATTAAAATGTT
TGACTGTTTCGCCTAATTATGAAAATTATGATGGGCTGATAGAGTATCAACATCATATTCTAGCATATACATAGA
TCCTCATCACAAATTAATTTACACAATTTTAGATTGATATTCTATATCTAGTCATTTTTGTTTTCGACAAATGAA
CATTTTTAATGAAGAGAAACAATTAACACATCATTGAGTCTCTTTTCAAGCAAAGCTCAAACCTGTATAAGGTTT
TCACACTTTTTAGGTCAGAAGTCGAATATAGGCTGTAAGGTTAGGAAGCTTTTTTATTTCATAACTGGATTCCAA
TTCTCTACAATGACCGAACAAGTTTGGGCCTAAGAAATCAAAGGGACATGAAAGGTGGGTTGAGATAGGCCCAAG
TTCCAAGAGGCTACGTAAATTGTGTTAGAGATACTAGCAAAAAATGTGTACATTTATGTATAACTATAAGGCATA
TGTAAATGCATATAACTCTTTGGATTATTCGAACAAAAAAAAAAAAACTCTTTGAATCCAAAGTTTTTTTTTTT
TTTTTTTTTGTGACTTAGTAACCCAGGGCACTTTACCTGCATTTGCAAGCAATGGAATTCGTCCTCAGTCCTCA

TAAAAATATCCTGTGGCATATGCGAGCCGAAAGAGATCAAGGTAGGAAAGGTGTTCGGATATTACTCAAATAATT
AAATTTCACACTTTCTATCTACTTCTTTTGTTTTGAGGACAAGTCCGAAGAGACTTTCGACTGAAATAGCTTTTT
TCCGGTACATATCATCTTTAAATATTAATGATATTTTATAAGAAAATAATAAACAGGAAAAAAGGAATATTATAA
CAAGATATTTACAAAGGACAACATTTCACTTCAGAACATATATACTGTGTTGCCAACAAAGAGTGAGGCCCAGAC
CCCACCTTTGCTTTCCAGGATTTATTTCCTCCAAATTATGTTGATTTAAACATTGAAGTCATCTCATTAGCTCTT
TCCGATAAGCTTTTTTATTAAACTTTTATCTCCTTGTAGGTACTTTTGATCATTACCTCGACATCCAATTGACTG
TTCTAACAGTTTGATACCATCTGTAGAAACAATAATTGTTTCTTGACAAAAAACCAAAGTGCCAACCACATGATC
GGAGAGTGCTCGTCTCAATGATGGTGCAACCTCTAACCCACCTAGCGTGAGAACCTCTTAAGACCAACACATCAC
GGCTATTCAGGTTCATCTCTTGCAACTTTTATAGAACATGAACATCTCCTACAACTTTCTCATTAACCATATCCG
CATTTTTTAAAACCGTTATCCGCATTCGCATATGCCGGTAGTTAATATCCATCGATATTTACACTACTGACCCGA
ATTGTGTATATATGAATTTAAATCAATCTCTAAAATACTATGAGTAGTTAAGGGCCGATGCTTTCTAAAAAATAA
ATGTTAAATACTACACCTCCATCCTAATTCAATTCTATTGCGCACTAATGTAGCAGTTCCCGCAAGTCCTTGGAT
CAATCATTGCAAATAAAAATATCGGTGCGATAAGAGTGTAGAATATAAGATTATTCTTTCTAGAATTGAATTATA
TGCTTTTCCCCTAACAAGTGAGATTATAAAAGGGTCTGAGCTCCTGCAGCTTAGGCACTCGACAATATCTTTCAA
AAGTTGATTCTCTTTTTCGTCTTTCACATTTCGTCCCCATCCAGAAA**ATG**GCGACAGATCCGAAACCAAAGAATT
TGGCTGAGGCAAATGAGTTCGTCTCATTGGAAAACCCAATATCCCGCAAGCCAAAACCAGGTCTTCTGAGGGCAC
TACTTGAAGAACATTATCCACCTGGTTTTCTCAGCAAGGTAAGATAAAGCTTCCAATAGTCCAACCACATCACTT
CATGTCCTCTCTACAACCAAAAAGGACACATTTTTAGTTTCTCAAGCTTGCTGGTTTCCTTTGAGGCTTTCCTTT
TTTCTGTTCCTATATTTTTTTTTAAGAAATTATATGTCAGGCAGCATCAGCCTTCATTTTTTTTTTTCTAATTGA
ATAAGGAAAAGGTTACATGGGAGGATCCTTACCACCCTTGATCCGAAAGGAAGAGAGAGTGGAGGATCCTAAAAC
CGAAAAAGGGGTGAGCTCCCCAACAATAGACCCAAACAGAAAAAACAGGCAGAAATAAAAACACAAAGTAGGAAA
TTGGTCAAACAAGTGACCCGGTCCTCTCAAGGGGCCGCATAACGCGGTTACAGAAGCAAAATGGGCACAGATTAA
GGCAAGCCTATCTGAACCCCCGCTAACCAACTGCAACAGCCACCAGAAGGAGGCTGATGCAGTGAATGCACTGTT
CAAGTCCAAGTTCAGGACTGAAGTAGAGAGAGGGACCAACACTAATAACGGATCAAAAGTCTCCAAAACAAGATC
TAACAACAACATATTAGGGAGACAATAACCCAATACAAACAACAACAAAATTACAAGAAAAAATACAGAAAAGTA
ACAAAAAATCCAGCAAACAGCAGCAATGGCGGGGGGGAAACGGCTGGAGCTCACTGGCAGGCACGTGAGATCCAC
GCACCGGCGAGTGAGATCCACTCTCCGGCGCGTGAGATCCACGCTCCAGCGCGTGGCCGTCGGAATGATGAGCGG
CTGATGGCGACGGAAGCAGACGGCGACGATGAACACTGTGGGGTGGCGCGTGACATGCTGGGTCTGGCAGAGGAA
GGTAGATCAAGTTTTTAAAAAACCCATCTTCGAACCAAGACAGATCCGGCCATCAACACGGTGGATTGCGCGGTG
GAGACGGTGTATGGCGCGGTGAAGATGGCTGACAGTGCTCCCGTGCAGTGGTGAGGATCGGCTGCTGAAAGGGGA
AAAACACCAACAAAAAAAGCAAAAAAGAACAAGAACGAAAGAAAAAACACGACGAACGGAGGAGAAGGGAGGAGG
GAGAGATGAGGAACCATCCCTCCTCCCTTCAAGGCACCACGAGCAACCGGACAAGCCCTCACTAAGGAGGTGGAA
AAGCCTTACTCTCTTAGAGAGAAGGTGGAGACTCTCTCTCTAAAGAGAGAGTATTTTTTGAAGGGTGATCAGCCT
TCATTTAGTAATTAAAACATTGATCTTACAAAATTCCCCATGCATATGTTTCATATCTTTGTCCAACATCCGGCA
TGCATGACTCATTTATCTTTTTGAGAGAAAAGTAATTCAACGGAAACGAAGAAAACTATAGAAGAATGTACCATG
TAATATATATGAGTCAGAGTTATACTCTAGCAAGGTTTCGTCAAGAACATAACTTTATTTGCGTAACACGATATA
ACAGGTGGTGGCAGAGACTATAGCGACCTATCTTCTGGTGTTTGTGACGTGTGGCTCAGCTGCTCTTAGCGCAGC
TGATGAAAACAAAGTCTCAAAACTGGCAGCCTCCGTTGCTGGAGGGCTCATTGTGACCGTGATGATCTATGCAGT
AGGACACATCTCCGGCGCGCACATGAACCCGGCCGTCACTCTAGCTTTTGCAGCTGTCAGACATTTTCCATGGAA
GCAGGTACATGAGCTTCTGAAAATCCCCATGGTCGAAACTAGGCCCTTGGAGGTTTCCTTTTTTGGCATTACATA
CTGTGCTCTTAGGTTCCATCCATGGACAATGTGCTGTCAAAATTGTTATTTGTCTTTGTCATAGATAAGAGTGGC
AATTCGTGTTAACATGTTGAATTCAGTCGTGTCGAGGCATAAATATAAGACTATATAGCTCAACCCTAACTTAAC
CCATTTAATTAAACGGGTCAAACTCTTCAACTATAACCCTCTAATTTTATATTGAGTTCGTATAGAGTTTGCAGG
TTATGTTAAAAATTTTCCACCATAATGTCATGTGTCGAATTCAGGTTATGTTGAAATATATATATTAGACTATAT
AAGTTATACGGGTCAAACTTTTAACCTAACTCTTTAATTTCGTGTTGAATTCCTGTCAAATTTGCAGATCATATC
AAAAATTGACAATCTTAATCATAGACTCATATATATATAATATATATATATATATATATATATATATGTTACATA
GAACAATACAAATTTTCTTCGTCATTTACCCTCAGACAGTCGCATGAAAATAGTAATGTCCGTTGACTCCTATAG
TTTTTGTTCCAAACATTATCTCCCATATTAAAAATTCAGGACTTCAGACAATTTTATCAGATATTTTACAAAGTA
ATAATTGAAGCATCTTCTTTTGCCTTAAATCTTAAAGAGAATTCAAAGAAGGATAAATAATGCTTATGCGATCAA
GTAACAAAGGAATGAACGAAACATTTTCTTGCAGGTCCCACTTTATGCAGCAGCTCAACTAGCCGGAGCTATTTC
TGCTGCATTTACACTGCGTGTAATATTGAACCCAATAAAGGAACTCGGCACTACATCACCAACCGGGACAGAAAT
TGAAGCCCTAGTCATGGAGATAGTAGTTACATTTACTATGATGTTCACCGCTTCAGCTGTTGCAACGGACACTAA
AGCTGTAAAATTGCATTCACATTCCAAATTTTACAAACCTCTTCAAAATGTTAATGTACTGATCATTAGAAAATG
TCTCCACTTCCCCAGATAGGAGAGCTAGCGGGTCTAGCAGTTGGTTCTGCAGTATGCATTACATCCATCTTGGCC
GGGTAAGTAAAATTGACTCATAATTATTTACCTAACCCATGAAAACTATTCCCTACATTGGCCAAATCCTGGATT
AAATAGAACTTCCATGCGAAGCAAGGACCGAAATTTTACTAAGCTTCATCATATAGGGTAAATGCCAGGTCGGGG
AAGACTCAACCTGGAAAACCCACCCATTGACCAATAATGAATACTAACAAAGTTGTCTAGTCGTACTCAGACCAA
TATCAGGGGGATCTATGAACCCAGCGAGAACAATAGGTCCAGCAATTGCTAGCGCATCCTATAAGGGGATTTGGG
TGTATTTGGTCGGACCAGTGACTGGAACGCTTCTAGGGGCATGGTCTTACAAACTGATTCGGGTGTCGGATGAGC
CTCTTTATCCAATGTCGAAGGAGGGGCACAGCAAAGAGATGGTGAACAAATATCCTAACAATTCTGTG**TGA**AAGG
ATGGTTGTGCACACCATGGGGAAATTGTGTCAAGCTTCCCGTATCTGGAATTCCCACAAGTCTTACAACTTTATG
TGCAAGATCTTGTATATACTTTTGTTATTAGCTTTCTATATCTGTTAGTAATGTGAGACCACCTGTGTGATAGTT
ATATTGATGGAACAGTATGTAGAAACGATTTAACCCAAGCATAATTAATTTCTCAGCCCCAAAAAATTTAAAGCC

AAATCTAAGGATAAGCACAGACCTCAAGTCCGATTCAAGTCGTGAAGCTTAATGATAATTCAAAAACACACCATA
AGTCTGTCGATTTTAGACTTACAACACTGACACACAACAGAGTGAAACTCTTTCTCCTCCCTACTTTTGGGTATG
CCCTAACCATTTTTTAGCCGGAATAATATGAAGCCTGGACTCATGGCAACATCAATGCAATTTAAAGGAACGTGT
CAACAAATGCCAAGGCATTATTAACACATCAATTTAATCTTTATATTTCTACTATATAAAATACACATCAATATT
TTAAAAAATTACATATGTAAGTGATATGTGCAGGGTCATAAGAACCAAATAAGTAATTGATCGTGGACCATTAAC
TGAGGAATGTCACCTTAAACAAAGTGCATCATGAAGCAGCTTTTTCACACACCATAAAACTC

>*Bpe*NIP2;1

ATGGCGACAGATCCGAAACCAAAGAATTTGGCTGAGGCAAATGAGTTCGTCTCATTGGAAAACCCAATATCCCGC
AAGCCAAAACCAGGTCTTCTGAGGGCACTACTTGAAGAACATTATCCACCTGGTTTTCTCAGCAAGGTGGTGGCA
GAGACTATAGCGACCTATCTTCTGGTGTTTGTGACGTGTGGCTCAGCTGCTCTTAGCGCAGCTGATGAAAACAAA
GTCTCAAAACTGGCAGCCTCCGTTGCTGGAGGGCTCATTGTGACCGTGATGATCTATGCAGTAGGACACATCTCC
GGCGCGCACATGAACCCGGCCGTCACTCTAGCTTTTGCAGCTGTCAGACATTTTCCATGGAAGCAGGTCCCACTT
TATGCAGCAGCTCAACTAGCCGGAGCTATTTCTGCTGCATTTACACTGCGTGTAATATTGAACCCAATAAAGGAA
CTCGGCACTACATCACCAACCGGGACAGAAATTGAAGCCCTAGTCATGGAGATAGTAGTTACATTTACTATGATG
TTCACCGCTTCAGCTGTTGCAACGGACACTAAAGCTATAGGAGAGCTAGCGGGTCTAGCAGTTGGTTCTGCAGTA
TGCATTACATCCATCTTGGCCGGACCAATATCAGGGGGATCTATGAACCCAGCGAGAACAATAGGTCCAGCAATT
GCTAGCGCATCCTATAAGGGGATTTGGGTGTATTTGGTCGGACCAGTGACTGGAACGCTTCTAGGGGCATGGTCT
TACAAACTGATTCGGGTGTCGGATGAGCCTCTTTATCCAATGTCGAAGGAGGGGCACAGCAAAGAGATGGTGAAC
AAATATCCTAACAATTCTGTG**TGA**

>*Bpe*NIP2;1

MATDPKPKNLAEANEFVSLENPISRKPKPGLLRALLEEHYPPGFLSKVVAETIATYLLVFVTCGSAALSAADENK
VSKLAASVAGGLIVTVMIYAVGHISGAHMNPAVTLAFAAVRHFPWKQVPLYAAAQLAGAISAAFTLRVILNPIKE
LGTTSPTGTEIEALVMEIVVTFTMMFTASAVATDTKAIGELAGLAVGSAVCITSILAGPISGGSMNPARTIGPAI
ASASYKGIWVYLVGPVTGTLLGAWSYKLIRVSDEPLYPMSKEGHSKEMVNKYPNNSV*

***Bpe*NIP4;1**

>**FXXK01001163.1:**60000-71000 *Betula pendula* genome assembly, contig: Contig1162, whole genome shotgun sequence

>**Bpev01.c1162.g0002.m0001**

Bpe_Chr1 16,645,942-16,653,541 (-1) ::
complement(join(16645942..16646158,16646421..16646482,16651141..16651338,16653042..16653263,16653431..16653541))

>*Bpe*NIP4;1

CAATATGGTATGTACTATGCTCAAAAGGAAGAGAGTAATTGTTTGTGCCAAAAAATATTAGGCTTTAGCGAAGCT
ATTCAGTAGTGTGAATGGCTCAATGTCATTTTTTTTTTCTTCCAATGGTTTCTGAAGGCTTAGGGAGTTATCTTG
TGTTTTTTTGCTGAGTGTGAGAGCATTACACATGTTAGTTGTGAGGGCGATAAGTGGTTGGTCTAATTAAGACCC
ACGAATTTGCGGGCGGAAGGAAGAGGACTGACGCAATTAGTTTGTTTTTCATGGTGGCTCCTTGGTCCACTCAGA
ATTGACATCCTTCCATAGCCTAACTGCAAGCGTATGTGTATTGAAATTACAAGGGAGATGTAAAAGGCTGAAGGA
CAATTATAAACTGATAGACTTGTATATGGGTCACAGAATGCAAAAGGGTACTTTTTTCTTTCTTTTACTTTTTGA
AATATTACAAACATTTTACATACAACAAAAGCCATGAGTCATTGACAAGGTAACAATGAAGTTTTCCTATTTGTC
AAATGTCAGCAGTATAGCTTCATAAGTGAAAAATGCATACACATGGCATGTGAACAAGTAAAAATTGTATTTGTC
AATGATGCAAAGACTGGGCTTGGAGGTGTCTACCTCAATTGATAAAATCCATGTTTTCAAACAACATCCTGAAAT
TGAATCCTGCATATATGTATAATTACGAAGGTTTAAGAATAGAAAGAACTGCCTCCGGTTGCCTAATCCATAGAC
CCAAAGATCTCTAATTCGAGTCCAAGGGGCCCAGCTGCATGCCACACTTGGGTGCTCGATGGGCTAGTCTTAATA
ACCATTATTGGACCAAATACTATATAGCATCCCAGGCGTTATGCGTCTTATATTTTGAGTGTACAACGGGCGGTT
ATTCAACCGGAAAAAACCGGATAACCGGAAAACTGGGAAACCGGAAAAAACCGGAAAACCGAAAACCGGAAAAAA
CCGCAACCGGATAACCGGATAACCGCTAGGCCGGTTGCGGTTGCGGTTTTTTATTCTGAAAAAACCGGTTACTAA
CCGGATAACCGGGTAACCGGTTTAATATATAATATTTTATAAAATAATATTTTATAATTAATTTAGACTAAACTA
AAAAAAATAAGGATATCAGTTGAAAATGATGTCTCATAAATATGTATTTTAAAGGTTCTATTGAAAGTTTATCTT
GGAATTCACACCATAATCAGGTAATATTGAAATTTCAGAGGCGAAATTTTTATATGGGGGAAGAATTTCATGCC
CTGCACAAAACACGTGGCATACTTTTTTTTACAAACAACTGACTTAAGTAAATGTATGTATGTAATATTTTAAAC
ATATATAAACTGAGGAGGCCTTATAAAACCTTAAAGAAGCCCTAGGGTGGCCATTTTTGCTTGCCCACAACAACC
CTAGAGAGAGAGAGAGAGAGAGAGAGAGAGAGAGAGAAGGAGAAGGGGTTTTTCTTGGAATTTTGTGTTTAAAGGGC
AAATAGCTCCTACCTTTAAGGTAAGAATGTTTGATATAGTCTTGATTTGTGTTATGTTCCATGCTATAAAAGCTC
CCTACAAATGCGCATAATGATCTTTGTGGAGAAGCCTTGGTGGGTCAAAGGCCTGTGCACACTCGGAAACCTCTC
TATGTTTGACATTTTGTCTCCATCATTGTTTTGGCTCAGTGTCACACACACATCCGTAAACAACTCCCAAAAGCA
GGAAAACCATCAATCGTTATTTTTGTTTAACTTTTAAATTTCCTTCCATGACTTCCGAATCGACTTCATTTCTAT
CATACAAGAGAAGGATAGATTCTTGTGTTATTATCATATCATCTTACTAATTATTTTATTAGGTGGAGA**ATG**TCT
TCACAAGACATCACAAAGGTGGAAGAAGGGAATAGCTCTTCTTCAAACAACAATGGAAATTCGGGGGTGTGCAAT
TCATCTGAGATAGTTCAGCTCGTTCAAAAGGTAAACAAACTTTTTCAGTGACTCACTCAGGGTAGCACAATTTTT

ATTATCAACTATTAAAAAAAATAAAAATGTAGGCATGAAATTAATTATTGTGGACAATTACATATTGATATGATG
CTCATATCCTCCTCCTCAATTTTTTTCATTTATTTTACTTTTTTCAGGTAATAGCTGAAGTGATTGGGACATACT
TCTTAATTTTTGCTGGGTGTGGCTCGGTCGTGGTCAATAAGATATATGGTTCAGTATCATTTCCCGGGATTTGTG
TAGTATGGGGTTTGATTGTCATGGTCATGATTTATTCTGTTGGTCATATCTCCGGCGCCCACTTTAATCCGGCAG
TCACCATCACTTTTGCAATCTTCAGACGTTTTCCCCTCAAACAGGTAGGTTAAATCCATCCAATAACAATTCACT
ATTAAAGGAAATGTGCTAGTATTTGATGAAGCTTTTCAGATAAAGTGCTTGACACAAAAATAGAGCTTAAAGCAC
CAAAATAAGTTTTCTTTAGATTTTTCTTAAATATAAGAAACTAAATTATTTTTTGTTTTTTTATTCAGATAATCT
GTATAATAGTTTTCCTTATATTTTCTCTATGCCATTTAAATATTCTTTAAATTAAATAATAAGAAAAAAAAAGAG
TAAAGAGAAACCATTTAAATATCATTTTAAACAATGTTAGAGAAAAAAAGAAAGTTTTTATATTAAAATAACGTT
AATGTAACCACTTTTGATTATCTAGATTTAAGGTACAATTCTTGGTTCCCTTAGTGTTTTCTTAGTATATGGAAC
AACAAGAGAATTTGTTTAAAAGGTTTTTTAGGAATTTTTCTTACATTTAGAAAATGAAATAGGTTTTAGGAAAGC
TACTGTTAGCGCTCTTATAGATTGATTAGTAATGAATACACAAATAATTAATATATATGTATATAACCACAAGGA
GTTAGCTCAAGTAGTACGGGTTTTGGTCTTGGTGACATCTCCGTCAAGACTAAAGTTTGAATCCCCTTGAGTGTA
AACAAGTTCTTGGGGCTACATCAGCGGCAAAGCCGTAGACTTACCCGATCCGTATGCCTTAATCATGTGAAGTGA
GCGCTTTGCACGGATATGGGTTTGCCCCACAGGGGTAGGTACATATAGGAGTGTACAAGCGGAATGATTATTAAC
CGTTAATAATCGGATAACCGAAAAATCGAAAAATAAAAAATAACCGCAACCGGATAACCGCCTAGGCCTATATAT
ATGTGTGTGTGTGTGTGTTTCAATTAGCACATCCGCATCCCACAGAGGAATGGAAGAAGTTCCTAACCATCGTG
CATGGCCTGTGTTGGAATTTTTTGAATATATCTTGTGGTAGCTTGTTAAGTTCTATATGTTATTTTTATGTTATT
TATATTTTATGAGAATATAAAATAGTTCTAAAAAAAATTAAAAAGTGAGTTTAAAATAGCTCAAAAAAAGGCCTA
AAAAATGAAAAGAGAGTTTAAAAGATCCCTTATTTGAAAACCGGTTACCAGTTTAAAACCGCATTAAAACCGGTT
AGTAGCAACTGCAACCGCAACTAGTAGGGTTGTCACGATTGCGGTTGTTTAAAATTAAATAACTAGGGAGCCTAG
TTGCGGTTACTATTAGTTGGAAATAACTACAACCACAACCAATTGTACACCCTTAGGTACATGTAGTGGCCACCC
CTTGGACAAGTTTTCCGTCGTTAAAACATATATATATATATATATATATGTGTGTGTGTGTGTGTGTGTGTGTGT
GAAAAACTAGAAATGGTCACTAAATTTTGTTAACTAAAACTATTTTGTTATTAATTTGCTTACAATAGGACTCCC
TTACATGTGCTCGAAAAAAAATATATTGATAGCCCAGAAAAGAAAAAGAAAAAAAAAAGACAATAATTAATAGCT
TTAAAAACATTATTTTTGCATTTTACGTTGTCACGTAACAATGATCTTATATATATGAGTCAGAAAATTTAGTTT
ATAAAAAATTATAGTTTAGCGTTTATAAAAAAATGATCTTATATGAGTCAGAAATTTTGGGTTTATAAAAAATTAT
AGTAAGTTTTTTCTTTTCGCAGGTGCCTCTATACATTGTGGCGCAGATGATAGGATCAACTCTTGCTAGTGCCAC
GTTATGCGCTCTTTTTCCTGTAGATAAAAAGTCTTTCTTTGGAACAGCACCGGTTGGATCCAATTTTCAAGCTCT
CATTATTGAGATAATCATCTCCTTTCTTTTGATGTTTGTTATTTGCGGCGTTGCCACCGACAATAGAGCGGTTAG
TAACTCTTAACCACAATTGCTTGCTTGTTCATTGTGTTTAATTGATCATTACATATATATCTTTCAATTTTTCCA
ACCGATGATTTCATACTTTCTAGTGAAGATTTGAGGCCATCAGTTTTGTGTGGATTTTTATAATTGTTTAATCAA
GAGGTGTTTCACGTTGATTGAAAAAAAAAAAATGGAGAAGATGAACCAAAGAATAAGAAAATTCTATTAAATGGTC
ACTAATACACAAAGATGGTACAAGTACAACACACACAAAAATCACTCTCTCACACTCACAAAATAAACATTTGCA
CACTTCACACACTCTCGTAAGAGCATTCATAACAACTACTCTTTAATTACTTTGCTTATCTAAATACAAGAAAAA
TTTAAATAAATAAATAAAAAACCACAAAAAATTACTTGTAGCAGCTTTCATATAGGTTCTTTAAATGTTGGGTAT
GCTATAGTGTTACGCAATGTATAGGAAAGCTTTTTAGGGAAGCTAGCTTTCCTATCCATTATTTTAATCCAAAAT
ATTTCTCTTTCATCTTTCCTCTCTCATCTTTTTGCCTTTCCTTGGAGACTATGTTGGGATTTTATAAAAAAAAAA
AATGAGGGTAAGTAGGGAATTGATATTTTGTAAAGAAATAATATTTTAATAGTATCAAAAAATAAGAAAAACTAT
TATGGAATGTATTTGGATAGAGAAACAAAAAGTAGTTTTATTCCTTACATTTAAGAAAAATCAAAAGGAATGTGC
TATGAATACTCTAAGAATACTAATAGCAGCTTTCCTAAATCCTATTACATTGCCTAAATGTAGGAAAAACTCATA
AAAAACAAACTGAAACAGATTCTCTAGATGTTTCTTAAACTAAAGGAACACCAAAGAAACCAAAAATGATACCCG
ATATCTATGGTATCACTTATGCTTCCCTTATTATTTTAATATAAAAATGTTTTCTCTTTCCTCTCTATTTCCTCT
AATATTTATTTGAAACAATATGTAAATGTCTTTTTCTTTCCCCTAACATTTAATTTAAATAATTTTTAAATGGTA
TAGGGAAGGATAAGGGAAGCTATTGTAGAGTTTATTTGAATAGAAAAGTAAAAAGTTGTTTGATTCCCTACATTT
AAAGAAATCTAAGGGAATCTGTTGCTAGTGCACTAAGAACATTTCCAATAGATGCTCTAAAACCTCCATATTGCG
TATATTGAGCGAATATATAGTAAAAATCACGTCCAACAACTTATTTTCTTGTTATCTAAAACATATATTTTGTTT
TTTGATTAGCTACTTTTAGGCAAGCACTTTAACTTATATATTCTTTTTTAATCATTTCTCTCTATTCTCTTTTCT
CGTTTAAGAAAACAATAAAAAAAAACTAAAAAATAATATTTAAAGATAATATAGAATGGAATAGAAAATTTATTG
AACTTTGTATTAAAAAAAAATAATAGGTAAAGTAAAAAAAAAATTGATATTTTTTTATTAGCTGCAATGCATAAT
ATTACTGGAGATGATCTAAGTGTGCCCATCACACTAAATTACATAGCTCTCACCAAAAACTACTTAGAGCACTCT
CATTAGGCAGCGTGGGTGTAACCAAAATGTTAAAAGGGGTCTAAAAAGTTCTGCATCACAAATTGCTCAAAATAT
TTGGTGAGTGTAAAAAATTTGCAAGCAGGACTATAAGTAGCATTGGAAATAATTTGACATGATAAATAAGAGCTT
TATTTTCTTTACCTAGATCAACATATATATTTTTAAAATTATGACCGTTATGAACAGACAAACTTTCAAAGAGAT
TATTGTTGGAAAAAAAAATAAAGAAATATTTTTAGAAAGAATATTTAAATGAAATTGTGAAAGTGGATAACTAAGA
TGCTGAGCCTAATCTAACTGCTTTGAAAGGTAGGTAGCTAAAATAGAAAAATTTTATTTTTTTAGCTAAAATAGA
GAGTAAATTTGTTGATACTGATGTGAATGCTCTTAACTCAAAAAAAAAAAAAAAAATGAAGAGGAGGAGGGAGGTC
ATCCATTGCCCCCACTAATAGGTGGTGGGACCCCACGGAAGACCTAGTTGGGTTGCCCCATCCACTTATGTTGAC
ATTGGTTGAAAGGGGCATGGGCAGGTTTCCCTATTCACCCACACTAAGAGGGTGGTGGTTGGCCACCCACCAAGC
CACGAAGGGTGTGGGTGGGTTGCACCCACGCTGCCATTGGCCAAGGGTGTGTGGGTTGATGACCCACCCACGCCT
GCTGAGGGTGGGTGGGTCTCCTCACCACTTCATGGGTTGCAACCCACCCAAGCCCTATTTAGGAGAGGGGGTGTG
TGTGGGTTGCAATCCACACCTTTTGCTAGTGGGCTTATTGTAACACCCCGATCTTATATTGGAAGATGTGGAAT
AGTCTGCATATAGTAAGTGGTTTATAAAGAGAGTCATAAGTGTTAAGTCTCATATTGCTTAATTACTAGTTAAAA

CTGTACATTATAAGTCATTCTAGAAAATCTTTAAAATTGACTAGTCATTTTAGGGTGATAGCGTAAAAGGACTAA
TCAATTTTGGAGTTTTTCTAAAATCACTTATAAAGTCCAGTTTCACCTAGTAACTAGGCAATGTAGAACTTAGCA
CTCATAACTCTTTTTATAAATTACTCACTCTATATGGCATATTTCTCATCTTCCCAATATAAGACTGAAGTGTTA
TACTTACTTCCTTCACTTACAGCTTAAAATGCATTTAGCCCTTCTCTCGCTTCCGTACTCGGTACTCAAGCACGA
CATCTATGAAAGCAGCAATGGGAGAACCGATGGTGAACAGGAAAAGGGATGACCACCCGGTCGCTAGGAAAAAGC
AAGCCTATGAAGGAGCAGACCAGTGGGGTCGTTTTTCAAGCAGAATATAATAGGTTGGTGGGTGGATTGCCACAC
ACACGATAAAGGGAGGGGGTGAGGTCGCCCCATCGGGATGACCACCCGATCGTTAGGAAAAAGCAAGCCTATGAA
GGAACAGACCAGTGGGGTCATTTTTCAAGCAAAATAGAATAGGTTGATGGGTGGATCGCCACACACACAATAGAG
GAGGGAGGTGAGGTCACCCCATACTCCGACTAGTAGAGAACATGGGTCGCGACTCTCTACCTCTGACGGCTTAAG
CGGGTCAATTTTCACCTGACTTTAAAAGCAATTCCAGCCAATCAAGGAGCTAAGAGAGAGAGAGAGAGTCAATTT
TCACCTAACTTTAAAAGCAATTCCAGCCAATCAAGGGGCTACCAAAGGAAGCCCCAAGAGAGAAAGAGAGAGAAG
GAAAAGAATGAGAGATCTAGAGAGGGGCGATGAGAGAAAAATGTAGAGAGAGAGGAGGAAAAGAGAGACGTGACA
GAGTGGGGGAGAAAAGAGAAGAGGGGAGGTCAATTAGCGGTGGTTGGAAAGAGAGAGGTGCATGGTTCTAGTTTT
TCAACACTTCCCAAACTATTTATGATGGTCCATGAACTTATCAGGCTCTTTTATATCGTCTTTCTCATGATAGGC
AATGTTAATATATTTGGGATTTGATTGCCTTAGCCAGCTTGATCTCTAAAGTTATGGTTCTGAGGTCTCTTCTAT
TTCTTGATGTTGACTTGGATAAAAATCTGCTAGCTAGAGCATCTAGATTTACCCTTCCTTTTCAGAGGCTAACAG
ATCAACCTCCATCCGTAAAGATCGAAACTCCTCATTCTTTAACTTGTTCTAGTTGTGCTCTATCTTCTTCAGGTT
GCTCTTGTAGCTTGATTTCCATTTCATTAAAATCTGGCAGCAACACTTGTCTTGAATACCACCAAGATCGATGAA
GTGTCGTCAAATACAACATTAATTCATGATGTCATGTATACAATGTCTTCCTCTCATCATAACCCCTCAAAAGGC
ATCGAATTATCTTTTCATCATACCTTTAACATCAAACATGCTTTGAAGGTAATCATTAAGATTTTGATACCTTTG
ACGACATATTTCTATTTGATACCTTTATGGTCGATGATAGGTTCTTGTGTTCATGGAAGGGTGATTGGGATTGAA
TTGCAAACGAAGTTGGATTTGAGTGAAAAAAGTATATCTCATTTTCATTGGGGCAAGAGATACCACAAAACTCGA
CTTGGGGCGAGAGAATATCACAAATTGCCAAATAGTTTTCTCCCCAATTCCTTCATAACAATATAATCTTTAAAT
AGGCTACAATAATTGATAAGAACTCCAATGATAAAACTAATCTTAATCTTAATTTTAAAACAACATAATCCTAAT
AGTCGAAGTTGATCACTTTTTCTTCTGGCTAAGATTGTTAATCAAACCTCTTCGCAAATGATACTTGTAGATCCC
ATTCATCTTCATTGTCATATGCTTAATTGGTGATCGTTCGATGTCACCATGTTTCTTTTTGCTAACTTTTTCTTG
GTTTAAAATCTCAAGTTCTTTGGCCACAATTATAGTATTTAACACTCTGATTTTGACGATTATATTCCCTTGAAT
GTTGCTTATCTCTTTGCACGTTATTAATTTTTTGAGTTCATCCTGATTGAGAATTCCTTCTCTGTTGACCTTTTT
TCTTGTCATCTTTTTTAAATTCGGATCTTCATATTTTTATTTGGTAAATTAAGTTGAAAAAATTCCCCCTCTTT
CAGATTGGAGAATTGGCGGGAATTGCCGTGGGGATGACAATAACCTTAAACGTGTTTGTCGCCGGGTACGTAATT
TCCCATCCCTACTTCAACTTGAAGCCATATTATATATAGGAGAAAAATGTGTGATTTATCTTATTTGTAATTAGA
TTTTTGTGGTATTAAAATTGAGTAATTTTAGGGACTATTTTTTTATCATCCTAATTGATGTGGCTTTTAAAATCA
CAATCGAATTAAAATTTAACAGTGATACATCTCAAATTCAATGGTTTTTTCTGAAAAATGATTAACACACTGGGT
TTCCTGATTATTTATATATGATTCCAGGCCAGTAAGCGGAGCATCTATGAACCCTGCCAGGAGCATTGGGCCTGC
TCTTCTCACGCACATGTACAAGGGACTCTGGATTTACATACTTGGGCCCCTCGCAGGGACCATACTTGGTGGTTT
CGCCTATAATATGATTAGATTCACCGACAAACCACTCCGGGAGTTAACCAAGAGTGGATCTTTTCTCAAAACTTG
TCCAAAAAATGGTTTC**TAA**AACTTACCAACCCTTTTCATAACTTTCCTTTAGCCCAGTGCAGCTTACACCCTTGT
TATGGTGCTTGTGGCTTACGTTAGGTTAGAGGAGGAAAACATTAAAATCAACATTAAAACTTGTTTTATCATTTA
ATTCGTGAATTGTAGGTTTCTATTTTTTTTTTCTTAATTTGCCTCATATACACATAATTCTACCTACATGTTTGT
AATACGCAATTTTTTGCCTCTTGACATGTATCTGCTACTGTCTTTGAACCAAATGATACAAGATTGCAATTGCAA
TTCACAAGTTTTTCGCATTACAGTTGTACTATATATGCAGTCTCTCTCTCTCTTTTTCTCAAGCATAAAATAAAT
TGTTTCTTAACATACTTGAATCTAGCTTCAACTCAATCACCTGCTAGCTAGAGAGAGAGAAGCTAAGGGGAAAAA
ACACTACACCCATTTACGAGCAAAGAAAAAAAGGAAAGCTAATTAAGAAAGGAAACAATATATGTGAGAAGAAGT
GGGTCTTTTTTGAATGAGCAATTATAGAGAGTCACAGAGAGAGAAATGTGAAGAGACGTGTTCTAAACTTAGACG
TGTCCCATACGTTAGTGCAATTTTTTTTTTTTGAAAAAAAAAATAAAAAATTGTGCATGGTTTATGAACATCGAT
TTTTTATTTTGATACGAACAAAACCTAATTTTTAGGCTGATGACTTTTCCAAGATTCCTACTCACAAATGTGATT
CCAAGGATCCTAAAATAATCTTTTGTTTAGTGTCTCTATCATGTGTTCGACAATTTGTTGCAACTCCACATGAAG

>*Bpe*NIP4;1

ATGGTCTTCACAAGACATCACAAAGGTGGAAGAAGGGAATAGCTCTTCTTCAAACAACAATGGAAATTCGGGGGT
GTGCAATTCATCTGAGATAGTTCAGCTCGTTCAAAAGGTAATAGCTGAAGTGATTGGGACATACTTCTTAATTTT
TGCTGGGTGTGGCTCGGTCGTGGTCAATAAGATATATGGTTCAGTATCATTTCCCGGGATTTGTGTAGTATGGGG
TTTGATTGTCATGGTCATGATTTATTCTGTTGGTCATATCTCCGGCGCCCACTTTAATCCGGCAGTCACCATCAC
TTTTGCAATCTTCAGACGTTTTCCCCTCAAACAGGTGCCTCTATACATTGTGGCGCAGATGATAGGATCAACTCT
TGCTAGTGCCACGTTATGCGCTCTTTTTCCTGTAGATAAAAAGTCTTTCTTTGGAACAGCACCGGTTGGATCCAA
TTTTCAAGCTCTCATTATTGAGATAATCATCTCCTTTCTTTTGATGTTTGTTATTTGCGGCGTTGCCACCGACAA
TAGAGCGATTGGAGAATTGGCGGGAATTGCCGTGGGGATGACAATAACCTTAAACGTGTTTGTCGCCGGGCCAGT
AAGCGGAGCATCTATGAACCCTGCCAGGAGCATTGGGCCTGCTCTTCTCACGCACATGTACAAGGGACTCTGGAT
TTACATACTTGGGCCCCTCGCAGGGACCATACTTGGTGGTTTCGCCTATAATATGATTAGATTCACCGACAAACC
ACTCCGGGAGTTAACCAAGAGTGGATCTTTTCTCAAAACTTGTCCAAAAAATGGTTTC**TAA**

>*Bpe*NIP4;1

MSSQDITKVEEGNSSSSNNNGNSGVCNSSEIVQLVQKVIAEVIGTYFLIFAGCGSVVVNKIYGSVSFPGICVVWG
LIVMVMIYSVGHISGAHFNPAVTITFAIFRRFPLKQVPLYIVAQMIGSTLASATLCALFPVDKKSFFGTAPVGSN

FQALIIEIIISFLLMFVICGVATDNRAIGELAGIAVGMTITLNVFVAGPVSGASMNPARSIGPALLTHMYKGLWI
YILGPLAGTILGGFAYNMIRFTDKPLRELTKSGSFLKTCPKNGF*

***Bpe*NIP5;1**

>**FXXK01001046.1:**25000-30089 *Betula pendula* genome assembly, contig: Contig1045, whole genome shotgun sequence
>**Bpev01.c1045.g0004.m0001**
Bpe_Chr11 981,169-985,394 (1) ::
join(981169..981384,984138..984557,984669..984730,985199..985394)

>*Bpe*NIP5;1
TGATAAAATAATTAAATAAAAAATTTAGTTTATTAATTTTTCTTAATAATCTTGAATTGTCATATTAATTTGTTA
ATGATTTTGTAGTGATAGATATAAATATCCTATATTTTTTGAAGTATATTATTTGATTGATACGTCATGGTCTTA
ACTTGTTCCCAAATACCATTGGAGAAATAAATAATGCATGAGTGTATGTGTGCTGGGCAGTTAGTCAATTACCTT
ATCAAACGCTTTAAATATTTGCCTAGAGAAAGATTAAATATGTGACATGTTTGGTTTAGTTGTTAAAAGATACTA
TATATCTTTTTGAAATTCTATATGTGGGTCTTGTATATTGAATGATGAATTTAAAAAAGAAATTGGTGAGACATC
GATGTGAGATAAAGGAGTAGTAGCATGTCTCTTAGTTTTTTAATCAATTCTCTTCTTTTTATTAATATTTGGTTA
AACAAAATTGATTATTTTGAGGCTCCCATCAAACGAGAAGTAATTTAATAATGAGTTCATTAAATATGCTTTAAA
TAGAATCTCTTTAGTTTACCAAAAAAAGTATTAAAATAGAAACACTCTTTAAGAAATTAAGATGACAGAAATAAA
CAAAAATTTATATATGTATATGTATAGGTATGTTTTAAGTACATATTTATTCTCTTAATTCAATAATCACTTTTT
CAATAATTGCGGACATTAATAAAGTACACACCAAACAACACCTTAATGCTAACTTGTGGGAGAGGAGATTCCTCC
TTTTTCTAGAGTATATTACGGTAAGATCTGTATTTTGATCCTTCCTTATCCAACGCTCAGTAATTAGAAAAAGAC
AAAAATTAATTTCTAAAAACTAACAATTTTTGGGAAACGTCTCATCGACTCTTGTTTTCTTATAATTATTGGTCG
GCGTATTATTGTATCATCTTGTTAGAAGATAATTTTTTTATTTTTTTATTTTTTTTCTGTGTCTTTCGGAAGAGCC
ATCAAATTAGCCAACCTCAAAAGTCAACTTGAAGAATAAGAGAATCCTGAGCACATGCTTCACAATTTTTTTTTT
TTGTAATTGTTGGATAGATATCAAGCAAGCCTATCGAATAAAATTGGACCGATTGGATTGAGATATTTTTACAAT
TTCAGAGAATTTTTTTTTATCTATATATTTATTACAATGGCTGATGGCAAACAATAGTAAAGTGATATGATTGGG
CTGTCATACCTTAATTGTACAGCATTATGGGGCCGTCTTTGTTTCTAGTCAATGCCTATGGTATGTTTATGATGT
ATTTTTTGTTTTGGTTATAAAAATGTTTTTAATGTGACATACAAGTTAAAGATATTTTATATTTTTATTTAACTG
TTTTATGCTGAAAATTGTTTGATAATGTTCTGATTTTTTTATAAAAGAATGAAAGTGGGTTAACAAACATGCAGA
TAGGCATCATTCCCTGTGTCATTAT**ATG**TACTATTTTTTTTTTTGGAGGAAAGTCTTTAGGGTTTATTTATTTATT
TTTGAGTGCTTTTTACGTTTCCTTGCTGAAGTGGAATTTAACTTTTTTTTTTTATTATTATTATTATTTAAAGGG
AGGGGGGGGGGGGAGGGGGAGAATTTAACTTGTGTGTAAAGGTTTATGTTTGTTAATGTATTTTCATGGGTATT
TCTTATTTTTGGCTTGAAAAAAATAACTTTGGGTGGATTTGATAAACGTTTTTGAGTTTTATTTATTTATTTTTA
ATAATAATAAAAAGTGATTAATACGATATAAAAAGTTAAATTGACAATAATAATAAATAATGATCGATAAAATAT
AAAAAGTTAAATTAATTTTTTTAGAGAAATAAGATGAGGGGAGGTAAAGATGTTGCCAAACCATGCCTTTATATT
TTTAAAAGTGTTTTACATTTTGAGTAATTTGTTAGTGTTTTACTTGTTAAGAAAAAAATGATCTTAGAAATTACC
AATATTTAGGACACTCTGACTAACTTAATTTTAATGAAAGACGGGTTTGGCATATTTAGGACACTGAGATGAACA
ATTTTGTTTTTAGTAAATAAAGTTAGTAATAGTGGTGGGTTAATTTGCAGCTTGGAGCAGAGTTTGTGGGAACTT
TCATCCTGATATTTATGGCGACAGCAGGACCCATTGTGAACCAAAAGTACAATGGAGCGGAGTCACTGATCGGAA
ATGCGGCATGTGCGGGGCTGGCAGTGATGGTCGTGATTCTGTCGACCGGTCACATATCGGGCGCACACCTAAACC
CATCCCTCACCATTGCGTTTGCTACACTGCGCCACTTCCCTTGGCTCCAGGTGCCCGCCTACATCGCGGCACAGG
TTTCAGGATCCATTTGTGCTTCCTTCGCTCTCAAGGGAGTTTTCCATCCCTACATGTCCGGCGGTGTTACGGTTC
CTTCCGTAACTGAAGGCCAGGCTTTTGCACTCGAGTTCATTATCACTTTTAATCTCCTTTTTGTTGTCACCGCCG
TCGCCACTGATACCCGAGCGGTACGTATATATATATATATATGACTGTTATCTAATCTTCTTCCATAAATTTTTT
TTAAAAAATATTCTTCCATCATCTTTCTTTTCTCATGGCCGGGTTCATCTTTCTAGGTGGGAGAGTTGGCGGGTA
TAGCAGTTGGAGCTACCGTTTTGGTCAACATTCTTGTTGCCGGGTGAGTAACTTCTTTTCTTTTCTCGATCGTGT
TATGGTTTTTTTTTTTTTTTAATAATATTATTTAGTAAATTGTAAGGCTGATTTACACCGTTAATAATATAACATT
CGTTTAACAGTCTTTTTTTTTTTAATAGGCTAACATAATTAAAGCTTAGTTTTTTTGGCCAGATAAAAAAATTAA
AAATATATATAGTTTTAGGGTTTTTTGTAGTCCGTAGAAACGTGTCCCCTCGCCCTCCCACTTCTTCTGTATTAC
AGTGTAATAGTGTATTGTAGGGTCAAAAGTTTAAAGCGACTAAATAAGTTCAGCAACTCTGTTAGCAATTGTATA
GGTGGTAGAGGGAAAGAGGCCGGTGGCAATTTTGTTGACATGCATTTTACTACTTTTACACCCAAAATTGAAAAA
AGAAAAAAAGAAAAGGAAAAAAAGGAATGTTTGCAATAATTGTTAGATTTGAACGTGGCAGGCCATCATCTGGTG
GTTCAATGAATCCCGTGCGAACTCTGGGCCCGGCAATTGCAGCAGGAAACTACAAGTCATTGTGGATATACCTGG
TGGCGCCCACACTTGGGGCCCTCACCGGTGCAGGAGTCTATACAGCGGTGAAGCTCCGAGAAGACGAGGTTGAAG
CACCGCGGCAGGTGAGGAGCTTCCGTCGCTAGGGGACGCGTGCCAAAGGGCCTAAAATTGCCGCTGCCCCTATA
ATTTCGTCGCCTTTGTTGTTTGGACTTTAGCTTTATGACATGACAGCTTGAAAGGTAATATAATAATTAGAAGAA
TAAAAAAGGTGTGGAGGAGATTTGACGATGTGACCGAAAGTCTACGCCCTACAATTTGAGTTGTATAAAAAAGTA
TGGTGTGTTGTCATCTCACGGGGTCTTCTCTCCTTTTTGGATGGAAGTCTCCTGTGATGTTAAATAAGCGCTTTG
CTATGAAACCAAGCAGCTTGTAATGATGTGTGTAGTGTGTACCCTTTGCTTTAATATGAAAGACACTGATGTGTG
TAGTGCGTTTGTAAACTTTGCTAATCAGTGAAAGTGCATGAGCACGAATGCCTTGTTTTATTTATGTTTGGTTGA

TCCCACTTTCTGCATTATAATACTCTTCACTTCTGTCCTTTATGCATTGCAACTTCGTATCCAGTTGGGCACAAG
CAAAACTTGGTTTGACCCATTTACTCAAAAGAGTAATTTGAGGTGTTTGGAATTGGGCCAACAGGGAAGTTTAGC
CCACTAATAATGGCAAGCGGTCTTGAATCAAAAGTGTTGAATCAAAAAGAGTGTAAATTCAAGTGCAGACTATTG
CAAGTAATAAATTATTAATAAATATGGTAAATATTAAGTAAAAATTTTATTATCGTGCAAAAAAGTTTGAATTGT
TTTTTCACCAAAAGGTGGAGCACATTAAAGTAAATAAACTAAAGTAATTAAAAATAGAGTTGGAGAAAAGTAATG
GAAAATATTA

>*Bpe*NIP5;1

ATGTACTATTTTTTTTTTTGGAGGAAAGTCTTTAGGGTTTATTTATTTATTTTTGAGTGCTTTTTACCTTGGAGCA
GAGTTTGTGGGAACTTTCATCCTGATATTTATGGCGACAGCAGGACCCATTGTGAACCAAAAGTACAATGGAGCG
GAGTCACTGATCGGAAATGCGGCATGTGCGGGGCTGGCAGTGATGGTCGTGATTCTGTCGACCGGTCACATATCG
GGCGCACACCTAAACCCATCCCTCACCATTGCGTTTGCTACACTGCGCCACTTCCCTTGGCTCCAGGTGCCCGCC
TACATCGCGGCACAGGTTTCAGGATCCATTTGTGCTTCCTTCGCTCTCAAGGGAGTTTTCCATCCCTACATGTCC
GGCGGTGTTACGGTTCCTTCCGTAACTGAAGGCCAGGCTTTTGCACTCGAGTTCATTATCACTTTTAATCTCCTT
TTTGTTGTCACCGCCGTCGCCACTGATACCCGAGCGGTGGGAGAGTTGGCGGGTATAGCAGTTGGAGCTACCGTT
TTGGTCAACATTCTTGTTGCCGGGCCATCATCTGGTGGTTCAATGAATCCCGTGCGAACTCTGGGCCCGGCAATT
GCAGCAGGAAACTACAAGTCATTGTGGATATACCTGGTGGCGCCCACACTTGGGGCCCTCACCGGTGCAGGAGTC
TATACAGCGGTGAAGCTCCGAGAAGACGAGGTTGAAGCACCGCGGCAGGTGAGGAGCTTCCGTCGCTAG

>*Bpe*NIP5;1

MYYFFFGGKSLGFIYLFLSAFYLGAEFVGTFILIFMATAGPIVNQKYNGAESLIGNAACAGLAVMVVILSTGHIS
GAHLNPSLTIAFATLRHFPWLQVPAYIAAQVSGSICASFALKGVFHPYMSGGVTVPSVTEGQAFALEFIITFNLL
FVVTAVATDTRAVGELAGIAVGATVLVNILVAGPSSGGSMNPVRTLGPAIAAGNYKSLWIYLVAPTLGALTGAGV
YTAVKLREDEVEAPRQVRSFRR*

*Bpe*NIP5;2

>**FXXK01001085.1:**70352-70951 *Betula pendula* genome assembly, contig: Contig1084, whole genome shotgun sequence

>**Bpev01.c1084.g0010.m0001**
Chr *Bpe*_Chr6 19,692,291-19,696,739 (1) ::
join(19692291..19692298,19692422..19692527,19694190..19694609,19695465..196
95526,19696562..19696739)

>*Bpe*NIP5;2

AGCGGATAATAACCGCATTTAATAACTGCAATAACCACGCAG**ATG**TAGTTAGTACTAATATGATGCAGATACCTA
AAACTGATATTTGTATTTTGCAAATGCAGCTACAAATATTACAAATAACTGCATCTACATATATACCCCTAACTT
TAACATCCCTTATCTTAACCCTAATTTAGCTCGTGAGTCAGCCCATGGTAATGGCATGGGCTTAGATATGCATAC
CA**ATG**ATATTGGGTCTCAATATCTTTGCAAGCATGGATCAATGCCTTCTGATTGAAATCATAAAAGTTTTAAAAT
TTATTTTCATTTTGAATGACATGCATAACTCAACGGTTTAGTTCATAACATGTTACATTCAAGGCATGGGTTTAA
ACCTTCTTAAATACAACTTGACATTTATTTTTAAAAAATAAATGCTAGATACTCCAATATATGAAAGGTTCGATC
TAGCTTTTATTTTATTTTATTTTATTATTGTTTTTTAATATTTTGGTTGAATGAGCAGGTCAACTTGAATGGGCT
TCGTTTGAGTTTAATGGAATGCTGCCTCCCTCTAACCTCCTTCCCGGTCCACTCAGTTGTATATATATATATATA
TATTTCCATTTGTTGCTTCTGCTTCTTGCAGACCACATCATTCGCATCATCCAGGCCAGGAAACCAGCTTCCGAT
CAATGCCGCGATGCAAGTGCTTTGGCGATCTTTCTGCACCTGATTTTTCTCTCATCCGAAAGGTACTCTCTCTCA
AACCAGTTAATTATAAACCGTGTAGCTTTTTTCTCCTTATTTTTTTTTTCTTAAATCCTTGGACTTGAAATTATA
TTTAGAATGGTGCAGTGACACCCTATTCCTTCCCTCCCTTCGTTGTTGTAGATTCTCCTTCATTTCCTGTAACTT
ATATATATATATATTCTTTCTTTTGTTGTTGTTCTCTCCATTTGTCCAAAAAAAAAAAAAAATTCATATGAGGCCA
TTATTTATGCAAACTGTGATTCCATAAGTACAAATATTTTTTTATTACGCTGAATGACTATTCCATTAAAAAAAA
AAAAATTTTATTAAAAATAAAAGAATGTAAAATTGATTAACCTTAAAATAGCCATTTTTATAGCCATAAAAGAAT
GACTATTTCAAGTATTCAAATATATGTATTTGTTAACACCAATGCTAGTTTATTCTATTAATTCCATTTTTAATA
AATGTTATTCATTTTTCCTAATAGAAAAACCATTACGTGTACAAAACATACTATTATTCAAAAATGATTTTTGTA
AACTTTCTGTGGCTAAATCAACTCAGTAGCATTGTATGAATGTTTATTGAAAAATCAATATAGTATGAACCCAAT
TTTTGCTTAAAACATTTAGTACTAAATGTTTATTTGGGTTTTTTTAAAAATATTTAGGGGACTTTTGGATTAAAA
TTTTGGCCTCTAGAGTTTCTGTCCATACACAAAATTCAATCCCCAAATCAGTCTTTGAAAATTCGTTTTGTTTAA
AAATAATTACCACAATACATATATTCGGTTTTTGGCCCTCATTAAAATTAGACTGGACCATTCATTAATGCTTTA
CCCCTCCCAATAAATTATTTAGGAAAAATACATTTAACCCGCCCATGAAGTTTGATTGTTATTTTAATTTTGTTT
CAAATGTTTAAAAATTGATAATGTACTTTTATCAAATTTCTAAATTTTGTAATGTAGTTCATCAGTTAGACAATT
CCATCTTCTTTGACGGAAAATATCTCACATACCAGTAACTCTTTTGAAAAATTCTGTCTTTTCTGAAGAAATTGT
CTAATGAATGAGATACATTGTAAAATTCAAAAATTTAGTGAAAGTACATTATTATTTTTTAAATATCGGAGGCAA
AATTAAAAAACAGGGTCAAAGTTTAAAAGGGTTAAATGTAGTTTTTTCAATTATTTAAGTAGGATGAGCCATTAT
ATTCCTCTAAGAGCCTGTTAGACATTAGCAGCAGCTTCCATTAAATTTCATTCAATTTTCTAGTGTAGGAAAAAC
TGTTAAAAAAATACAAAAGTCCATGCAGCAGCTTCCTTAAGTGTTCCTTAAACTATAGAAACATCTAGGGAACA

AAAAGTGGTTATCTTAGCATTATTTTAATATAAACAACTTTCTCTCTCCTTTCTCTTTCTTTTACCATTTGTTTA
AAACAATATTTAAATAATTTCTCTTTCTTCTCTCTTTCTTCTAACATTTAATTTAAATTATATTTAATTCATATA
GTGAAAGATAAAGGGAAGCTGCTATGCAGTGCATTTATATAGGAAACTAAAAAATAATTTTGTTTCTTACATTAA
AAAAAAATTTAAGGAAAGCTGCTGTTAAATGCTCTTAGGGATTGAAGACATACTATTTGTTCTAATGCATGCAGC
TTGGAGCGGAATTTGTTGGAACATTCATCATGGTATTCTCAGCAGCAGCAGCACCGATTGTGAACGAAAAGTACC
AAGGAGCAGAGACGCTGATTGGGAGTGCGGCTTGCGCAGGGCTTGCTGTGATGGCCATGGTTCTCTCTACCGGCC
ACATCTCGGGAGCGCACTTCAACCCATCCGTCACCATAGCATTTGCGGCGCTGCGTCACTTCCCGTGGGTCCAAG
TGCCGGGCTATTTAGTAGCGCAGGTTTCTGCCTCCATTTGTGCTGCCTTCACTCTCAAGGGAGTATTCCATCCAT
TCATGTCAGGTGGAGTTACCGTTCCGGCGGTAGGCATTGGCCAGGCTTTTGCACTCGAGTTCATTGTTACTTTCA
CTCTCTTGTTTGTTATCACCGCTGTTGCAACTGATACCTGTGCAGTATGTATTATTGATCACATTTTTGATTTTT
CCATATACTACTCATTTGTTATTATTCACTGAGTTTCACGTAAATTACTTATTAGAGAAAACTTCACTTATCCTA
ATGTTCGGCATTTTTGTAATCCTACTTTTGAAGTTTAAAAAGTTAGAATGCCGGCTTGCCATCTATTTAAAACTT
TGTAATGTCACCTCTCCGTCAAGATTTTTGACTACATCTTAACCGAGAATGTCAAAATCCCCAAAATAAAAAAA
TAGAACACATTCAGGCGTGGGTCCGATGATCCAAGGCCATTGAGCTTTTCTTTTTTTCCTTTTTGTTTTTAATTT
TTAATTTTTTAATTAAGAGTAGTTTTATTTTTTTATTAATTTTATAGGAGTATAAAGGACATTTCATCACTTTAT
CCTCGGATTAATTTAATTATAAGAGTAATTATATATGTCTACCAACATATCTATCTTGTGTCAACAAAAAATGAC
GTGGCTTTGAAAATCACTGTTGAATTTATAATGGATAATTATTGATTTTTGATCCAATATTAATTTTAAATACTA
CATCATTTTTTGTTGACACAATATAGACATAGAAGTTGACTTGTACGTATCATTACTCTAACCATAAATCCTAAT
GTATGATAGGGTTGACAATACAAAGTTTTGAAACATGTCAACCCAATATTTTAACTTTTAAACTTTAAAGATACG
ATTGTAAAAGTGCTGAAATATCGGGATAATTAAGTGAAGTTTCCCTGAAACTTATTTGAAGTTAAAATTAAATGG
CCTTGGAACCGATGCAATAGGTGTACCGAGGTTCTTCTAATATTTCTCGGGGCTAATTAAGTGTTTCATGGCAGG
TGGGAGAGTTCGCAGGAATAGCAATTGGAGGTACTGTTATGCTCAACATTCTTGTGGCAGGGTTAGTAACTAGTA
TTTCTTTTATCATTCATTCTTTATTTAATAAAGCCAATTCAATTCGTCATTTTCTCCTTTCGAGTGATAGTATCA
TCTGGTGAATACCAGGACTGGACAGGACCAGCTTCTAGAATAAAATTTCTGGCTCGCAAATATATGAACTGAAGT
ATTCCTGACCATGTAATTTCCTTAACAGCAAGCATGTGACATTATCATTATGCCCTTTATATTAGAAAAAATAAA
TAAATGAAAAATCCTCATAATCTCCATGGTCGTGGAGGGTGGCCATGGAGTTAGCTCTATTTTGGCCCATAGAGG
TCATCTCCATGTTCATGGCCATGAAGGGTGACCTCTATGGCTGTAGAGGTCCATGGCTACGGAGGTTGGATAAGG
TTTTTTCTTTTTTTTTTTGTCATATTAATTAGGAGGCATATATAATTACAATTTCACATGCATGCCTTCTGTTAT
CAATTTAGTGTAGCAAAAGAATAGTTGTTGACATGTTGTATATTGCAACATGGATAGCCTGAGGTAATGAAATTA
CAAAAAAGAAAAAAAATTGAAAACGTTTTTAAAACAAAATCGACCAAACTACAAAGTGATCGGTTTTGTTTGATA
ATCTTTTAAAGGTTTGCATTTTTACTTTTTTTGCAAAGAGAGAAATATTATTAACAAAAAACATATATAAAACAA
TTAAAATTACTTTTATCTTCATCTCATATCGCATCCAAAAAGTAAAAGACACCCTTCTAAAAGGCAATAGTACCA
AACAAATTCTTTATGTTTTCCTTTTTATTTCATTGACAAGTTATTATATATTGTCATATAGAGTTTTTCATATGA
TCCGCTCTCTTTTTCTCTTAATATATTTGATTATATATTTCTACAGTCTTGTTATTATATAAGCCACTCTTTGTT
TTTTTTAACGAATATGATTAATCTGTATTAAGCTGCTTAATGACGTTGATTACTAAACAATATAATAAATGGAAT
ACAAAGAAGATATATATATAGTGATTTCTTCTGTTGTTGACATAGCAGGACAACGACCGGCGCATCAATGAATCCCG
TAAGAACAATTGGGCCAGCCATCGCAGCAGGAACTTATAAAGGATTTTGGATATATATGGTGGCACCTATACTTG
GGGCCTTGGCCGGTGCAGGTACCTACACCTTCGTGAAGCTCGCAGAGGATGGCGTTGAAGGCCAGGATAAC**TAG**C
TAGGAGCTTAAAGTTAGCTCTAATGAAGATAAACATTAGATTTGTTGCAATTACGAGTGATGCTTAAGATGAACA
GTCAGATATGACACCTTGAAAATGTTCTGATAATGTCTGATCATATCGTTAATTAACGTAAATAATATGGTGATT
GCTTTTCTTGCATCTTGCTTGTTAAAAACATACATATGAATGCTAGATCATATTATAAATACGCATATATATTGT
GAATATATATGATCTCATGTTACATAAATTAATTAAATACCTACCTTAAGGATTTTCATGAAAAACCTACTTCAA
TGCAGTGGAAAAAATAAATCATCGGTTGCTTTTCTGTCACTCCTCTCGATTATAACATCATAAGAACTTGTGATT
GGTGGTTTACCATGCAAGCAAACCACACATTTCTCCTACAATAAAAGAATCTTAGAATATTTTTAGTTACGATTT
TCAAATGTGTTTTCGTGTTTTGATTTGAATTTCTTTATATATAATAAAA

>*Bpe*NIP5;2

ATGATATTGGGTCTCAATATCTTTGCAAGCATGGATCAATGCCTTCTGATTGAAATCATAAAAGTTTTAAAATTT
ATTTTCATTTTGAATGACATGCATAACTCAACGCTTGGAGCGGAATTTGTTGGAACATTCATCATGGTATTCTCA
GCAGCAGCAGCACCGATTGTGAACGAAAAGTACCAAGGAGCAGAGACGCTGATTGGGAGTGCGGCTTGCGCAGGG
CTTGCTGTGATGGCCATGGTTCTCTCTACCGGCCACATCTCGGGAGCGCACTTCAACCCATCCGTCACCATAGCA
TTTGCGGCGCTGCGTCACTTCCCGTGGGTCCAAGTGCCGGGCTATTTAGTAGCGCAGGTTTCTGCCTCCATTTGT
GCTGCCTTCACTCTCAAGGGAGTATTCCATCCATTCATGTCAGGTGGAGTTACCGTTCCGGCGGTAGGCATTGGC
CAGGCTTTTGCACTCGAGTTCATTGTTACTTTCACTCTCTTGTTTGTTATCACCGCTGTTGCAACTGATACCTGT
GCAGTGGGAGAGTTCGCAGGAATAGCAATTGGAGGTACTGTTATGCTCAACATTCTTGTGGCAGGGACAACGACC
GGCGCATCAATGAATCCCGTAAGAACAATTGGGCCAGCCATCGCAGCAGGAACTTATAAAGGATTTTGGATATAT
ATGGTGGCACCTATACTTGGGGCCTTGGCCGGTGCAGGTACCTACACCTTCGTGAAGCTCGCAGAGGATGGCGTT
GAAGGCCAGGATAAC**TAG**

>*Bpe*NIP5;2

MILGLNIFASMDQCLLIEIIKVLKFIFILNDMHNSTLGAEFVGTFIMVFSAAAAPIVNEKYQGAETLIGSAACAG
LAVMAMVLSTGHISGAHFNPSVTIAFAALRHFPWVQVPGYLVAQVSASICAAFTLKGVFHPFMSGGVTVPAVGIG
QAFALEFIVTFTLLFVITAVATDTCAVGEFAGIAIGGTVMLNILVAGTTTGASMNPVRTIGPAIAAGTYKGFWIY
MVAPILGALAGAGTYTFVKLAEDGVEGQDN*

***Bpe*NIP6;1**

>**FXXK01000045.1:**935000-945000 *Betula pendula* genome assembly, contig: Contig44, whole genome shotgun sequence
>**Bpev01.c0044.g0051.m0001**
Bpe_Chr5 8,366,764-8,370,948 (1) ::
join(8366764..8366994,8367790..8368014,8368159..8368353,8369537..8369598,8370744..8370948)

>*Bpe*NIP6;1

TGGCTGGTTTGTAGTTTGCTGCTCTTGTACTTTACCAAAAAAAAAAAAAAAAAAATCTGCCATCACCCAAGTATGA
CTTCCTTCCACCTGCAAACAATTTACCTACCACGCAATACACGCTCATCCAAAGATTGCTTTAATATCTAGAGGA
AAGGGTTAAAAATGTTATTACTAGCTAATTTACCTACCATGACTGCATCCAAGGGGCGGACCTACTTGTTAGGGT
GGGAAAAATTTTAAAATTCTTTTTAAAGTATACAGCTTTTATAAATTTGTTTCCTTAAAATTATATTTATGGCCC
TTTTTAAAAACATTTCTTTTAATTTACCCCATCTTCCAACATAGGAAACCAAAAAATTTTCTTGTTTTTTTACAT
GTCTAAGCAAAGTAGGAAATTTGTGTATTCAAAATGAATTTCTTTTTTTTGATATCTTTTAAAATAACTTTATAA
ACAAATAACTTTTGTATATAGTAATGAGTTAAGATTTAATATGAAATTAACACTACTAAAAAAAGAGTAGAGCTG
GGCGTCCGTTTTCTATTTTAAAAAAAAAATAAAAATTAAAAAAAAATGCTTTTTTCTATTATAAAACAGGCTTAAAC
TTGTCCAATTCTCATTTTGTTTTTTCTAAGGTTATGAAATGGGGATGAAATAAGCCTGAACCTGTTTTTTGTTAA
AAAAAATATTTTTCTTATTTTTTATCTCAATAAAAAATAAGAAAAAATATTTTTTTAACAAAAAACAGGTTCAGG
CTTATTTCATCCCCATTTCATAACCTTGAAAAAAACAAAATGAGAATTGAACAAGTTTAAGCCGTTTTATAATAG
AAAAAAGCATTTTTTTTTTTATTATTTTTTTTTTTCAATTAAAACGGACGCTAGGCGTCCGCTTGTGCGGACGCC
TAGCACGACTCCTAAAAAAATTACCTGTATGATGCATCTTTTTAGTGCTCGTGCAAGATGCGTCGAAGAAAAGGC
GTCCGTAACAAAATAAGAGTTGGTATAATATTTATTTTTTGTAGTGTAAATTTAAGACTTTTCTAGTTTTATATA
ATATAAAACTCGCACAAACACATATGAAAAAAAGACTATATGTACATCTCTTTGTGCTTAAATATTTATATAAGT
TAGTTCCCCACAGCACCGATCCTCATTTGGACCTGATTGCATCTCTCTATTCTTTTATAGGCACACAGATCGACA
CACTTAAATAGGGACTTCATTATTGGGAGAAGACATTGAATAGAAAAAAATTGAAACAAAAAGAAAAGAAAAGTA
GGATCCTTCTGTAGCATTGACAGGAAATCCATAATTCATGTCCCACGTTAAAATCCTCTTTCCAAAAAACCAAAT
TCGATCAAGACTCTCTGCAATGTCCCACCACCCACTTTCCTCATCTCCAGCACTTCCAACCATTAAACTTTCTTT
TCTCTTTTTTCACTTGTGATTCCCTAAGTCAACCACCTAGTCACTATAAATATCAGACACCTCAGTACTCTCTTC
AAAGCAAAGGGACAAAGAAGTGGAACTCAAAATTCAAAAGCTTCTCTCTCTTTCTCGAGCTTTGTTTTGTTTTTG
TTTGTATTATTTTAATCATTTTCAGTTTTGCTTTGGAGTG**ATG**GAAAATGAGGAAGTTCCATCAGCACCTTCAAC
GCCTGCAACACCAGGGACTCCAGGAGCCCCACTCTTTGGTGGGTTCAAGGCAGAGAGAAGTGGGAATGGTAGAAG
ATCCCTTCTCAAGAACTGCAGCAAGTGCTTCAGTGTTGATCAAGAATGGGCCTTGGAAGAAGGCACCTTACCCAA
ACTCTCTTGCTCTTTGCCACCTCCTCCCATTCCACTTGCAAAAAAGGTACGCTATGGATACAAATTTCAAACAAT
TTTGTTGCCTTCCACGTGGATCTCATTTATTCGAACAGGTTTAATTAAATAGTCAAAAATTTATTTGGACAAATA
AATTTTAGGTTGACACTTTTACAATATGTCATCCAAATTGTTGAATGAGATATGTTAGAGGTAAATCTTATAGTC
TTCTCCTGTCCAACTCCGATGGATCGCACAATTTCAATTGTTAAAGTATTGATTAAGTGGTTAAATTTACATTTT
TCTATCAGCTTAAGCTTTTAACTTAAGCTTTTGAGACAAGTAGTGGTTTAACATGATATCAGAGGCAAAGGTCCT
GAGTTTGAACCTTGACTCCGTCATTTACCTCCCATTTTAATTAATATTTCACGTGTTGGGCCTCATCTATTAAAA
GGGAGTTTGAGTTCACATGTGAGGGGGAGCGTTAAAGTATTGATTAAATGGTTAAATTTACCTCTTTCTATCAGC
TTAAGTTTTTAACTTAAGCTTTTGGGACAAGTAGTGGTTTAATATCAAGAATAAATTTATAAAGTTAAAAAAGAA
TTATTTCTCTTGTTGAATATGTAAATTTCACAAATTTAATGGTAAATTTGCAAGAGTTAACCGGAGATGACGATC
AGTTTGAAAAAGAATCCTATGTCAAAAGCTCAAAATCAAACTGACCCACGTTTCCTTTATGTGTTTATTTTCATG
GGTCATTTTTATGTCAAAAAAAGGAAAACGAAAACTAACCCATATTTCAATTACGGCTTATTGTTCACATCTTGA
TGCCAAAATTAATCAGGTTGGAGCTGAGTTCATAGGCACTCTCATACTGATCTTTGCTGGGACAGCCACGGCCAT
GGTGAACCAAAAGACACATGGCTCAGAAACCCTCCTTGGCTGCGCTGCTTCATCTGGCCTTGCTGTTATGGTTGT
CATTCTCTCGACCGGACATATCTCTGGGGCCCATCTCAACCCAGCTCTCACCATTGCCTTTGCTGCCCTAAAGCA
CTTCCCATGGAAACATGTAAAAAATTTCATTTGCCCTTTTCTTGCAATAATTCTAAAGAGTAATGTTATAGGAGA
CTGTTATACAATTTTACTGTTACGTTGTTCTAATCGTATATTAATTTACTAAATAGTGTTTTTTATTTTTATATT
AATTTGGCAGGTGCCTATGTATATTGGAGCACAGGTTGTGGCATCTTTTTGCGCTGCATTTGCACTGAAGGGGAT
TTTCCACCCAATAATGGGAGGGGGAGTCACTGTTCCTTCCGGGTCATATGGCCAAGCTTTTGCTTTGGAGTTCAT
AATTAGTTTCAACCTCATGTTTGTCGTCACAGCCGTGGCCACCGACACAAGAGCTGTACGTGATTTCTATCAACC
TTTTAGACTTTTAGTGTCATGATCTTGAGAAAAGAACAGAGTAAAAGAAATTTTGCAGTATATTTTTTTTTTCAT
GTACCACATTTTTATCTCAGGCTCATTTAATTAGATTAGTCTTTATTAAAAGCAATATTTAATAGTTGATAAACA
CTATTATATCAGTTCATTAAAATTAAAAAAAAATAGAGTGCAATATATAATATTTCTTTTTTTAAATAATATATCA
GTTCATGACATTTGACTAGCCAAATTTTAGGCCCGATGCATCCTTAAAAACTAAAAGAAATAAATATACAACAAA
TTAATCAGAATTACTTCTTCAACAAATTAATCCTCTCATTTTTTTTCACCATGTCAAACTGTGTATTGAAGAGAC
GTTTGCATATAAAAAAACAAAAGAGACGATTGATCAATGACTAAACAGTCATTTTCTTTTCCTTTCTATTGAAAA
TAGAAGACATCTACAGATCACATGCACTTAAGATTGTGGTTTCCCTCATAAGATAATATTTCTCCTCCTTTTTTT
TCTTTTTTTTCTTTTTTTTTTTTTTTTTTAAAAAAAAAAACATTTTTTGATCTTTAAGCTCACTTCCCAGGGATTT
TCCACGAGAGTTACTTCTCTTTTTTAAAAAAATTTGTTTACAAAGTCAAAGAGACAAGAGTCGGTAAATACTTGGC
AGCATGCAAAGTTCCATTTCCCATAAATATAATAAAATAGTACAAGTTGTCGATTTCTTTTTTTTTTTTTTTTTT

TCGTATATTACAAGTTATCGATTTCACTAGGCTCCAATATTCAAGGTAGAAAACATCCAGAGAAATAAAATAAAA
TAAGGAATTAACACGTAACTATAATATTCAGTTTATATATCTAATATTAATTATGAGCACCTCATAATAATTCAA
AGCTAAGCACTTTTTCCAAAGTGCCTATCTTCAAAATCAGAACAATTAAGGAATAATTAAAAAGAAAATTAATTT
TCGATAATTGGCTGGTACATATATAAAGTCTTAACTGGATATATATATAGTAGCCTGACGAACTATTTTGCGCAC
TTTACTGAAACCAGGGTATTCTTAAATTTTTTTGCATTTTGCAAAGAAATTAAGGGGGATGCACAGAGTGAACAT
AACGTGACACTAATGATAAAATATTCTGCATCATGCAGGTGGGAGAGTTGGCGGGAATCGCGGTTGGAGCAACGG
TCATGCTCAACATCCTCATAGCCGGGTACGTATGTCAGAAACTCCACTTGCTCTCAACTGAAGGTCTTTGAAACA
AATCATTATGAGTAAGAAGCAAACCACATAACATGGCTCATGTCCCCCCGACCAGGATTCCAAGTAATTTGTTGT
CATGACCCATGGTCATGTCAGAGAGTTTCAATAAAGTTTATTTGTCCAATTTCTTGAGATTTATTTAGAGCATTA
ACAGCAGCTTCTCTTAAATTTTATTCTCTTTCCTAGTGTAGGAAAAACTGTTAAAAAAACACAAAAAAGTCCCTG
CAGCAGCTTTCCTAAGTGTTCCCTAAACTATAGAAACACCAAGGGAACCACTTTTGGTTCCCTTAGCATTATTAA
AATATAAAAAACTTTCTCTCTCCTCTACCATTTGTTTAAAACAATATTTAAATGATTTGTCTTTCCTCTCTTTTT
CTTCTAACATTTAATTTAAATAGTATTTAATTCATGTAGGGAAAGATGAAGGGAAGCTGCTGTAGAGTGCATTTA
ACTAGGGAACTAAAAAATGGTTTTGTTTTCTACATTAAAGAAAAGCTTAAGGGAAGCTGCTGCTAATGCTCTTAG
ACATATAAACTTTATAGGAACTCTCTTATATTTACTATAAGAATTACTAATAATCTCTACAACAAAATTGTTAAA
GATTTCTATACCATGTCCCCTCTTTGTATCTTAAAGATTTTTAAAGACAAAGCATGTTGACTAGTAGCTAGGCTT
AACAGAAATTTAAATGTAAAAGTTTTGCCAATCGATCAGCTCACTCAAATGGCACTTTCTCCCATATAAGAGGAT
GAGGTCCTGAATTTAGGACTATTGAATGTATGTAAAACTTACCAACAAAATAATTTTTTTTTCTATTCTACCAA
CAAAAGTAGGTGTCCTGTAAATGAAATTAAAAACCAAGATGGGTATGGGGATTCGGGAACATAAAACACATAAAC
CATCATTCAAAAAGTAGGTGTCCTGTAAATTTTATGCTACATTTGTTGATGCTTTTTGGTGGTATTTTTTATTTT
TTTATTTTAAAAAAATATTCTTATGTGACATAAAAGGAGAAATATTTTTGAGTGTTTATGTTTTAAGTTGTTTGT
TAATACTCTAAATTTTATATGGTATTGTTTTGTGCTATCTTATAGGCCAACAACCGGAGCTTCAATGAATCCAGT
GAGAACACTAGGGCCAGCCGTTGCTGCAAACAACTATAAAGCAATATGGGTCTACCTCACTGCTCCCATCCTTGG
GGCACTGTGTGGGGCAGGAATCTACTCTGCCGTCAAGCTGCCGGAAGAAGATGGTGGCATTCATGAAAAGCCTTC
AACAACAAGTAGCTTCAGAAGG**TGA**CCGACCAACACCATCATCGCACCTACATTAATAACATCGAAACTCCAACA
GAATATAATGAAGGTGGATAAAAATGAGTATGACAACAAGATGAGAGACAATTTAACAATGAGTCTTGTGGGTAC
ATATATATATCACACTAATAAAATAGAGTAATGCTATTTAGTAGATTGTTATACGATTGTGATACAACTTGATGA
CGTGACTTAGATCAGCACATGTAAAAAAAAAAAGAAATAATATGTTGATTTTCAGTGTCATATCATCTCAATTAT
ATGTCAATTGTATAAAAGTCTACTAAATAGCATTACTCAATAAAATATCATCAATTAGCAGTATCAATAATACAT
ATTTAACAATCAAGCTATAATTTCCCCCATGTAACGGAAAGCTAATTATAGAGTATGAGGATTGAGAGATCACTT
TCATCTTCAAGTACATATTAATACCAGCACCAGGTAATAGAGATATAGTAGTCTAAATAGGTGGAAAACAAAATC
TCAGGTTTTTAATGCATTATCATTCAGGTCTATCTTTTCTTTCCACTGTATTTGTATCTCTTTTTGATCGTTTAA
AAAATGCATGAACATTTAAGATTGTATCTTTATGTGATATTTCCTCCTTTTTATGAATCTTTGAGCTCATTGTGC
AAGGACCACAACAATATAAAGATGGTTGGGAATCAGGTTTACAAACTTCCTCATCTCAACTAATCACTAGTAAAG
ACTAGCTAAAATGAAATGATTGAATCAGGCTTTTAAGCAGCAATACAATCAGTTTGCTTTTCCATTTTGTTTTCA

>*Bpe*NIP6;1

ATGGAAAATGAGGAAGTTCCATCAGCACCTTCAACGCCTGCAACACCAGGGACTCCAGGAGCCCCACTCTTTGGT
GGGTTCAAGGCAGAGAGAAGTGGGAATGGTAGAAGATCCCTTCTCAAGAACTGCAGCAAGTGCTTCAGTGTTGAT
CAAGAATGGGCCTTGGAAGAAGGCACCTTACCCAAACTCTCTTGCTCTTTGCCACCTCCTCCCATTCCACTTGCA
AAAAAGGTTGGAGCTGAGTTCATAGGCACTCTCATACTGATCTTTGCTGGGACAGCCACGGCCATGGTGAACCAA
AAGACACATGGCTCAGAAACCCTCCTTGGCTGCGCTGCTTCATCTGGCCTTGCTGTTATGGTTGTCATTCTCTCG
ACCGGACATATCTCTGGGGCCCATCTCAACCCAGCTCTCACCATTGCCTTTGCTGCCCTAAAGCACTTCCCATGG
AAACATGTGCCTATGTATATTGGAGCACAGGTTGTGGCATCTTTTTGCGCTGCATTTGCACTGAAGGGGATTTTC
CACCCAATAATGGGAGGGGGAGTCACTGTTCCTTCCGGGTCATATGGCCAAGCTTTTGCTTTGGAGTTCATAATT
AGTTTCAACCTCATGTTTGTCGTCACAGCCGTGGCCACCGACACAAGAGCTGTGGGAGAGTTGGCGGGAATCGCG
GTTGGAGCAACGGTCATGCTCAACATCCTCATAGCCGGGCCAACAACCGGAGCTTCAATGAATCCAGTGAGAACA
CTAGGGCCAGCCGTTGCTGCAAACAACTATAAAGCAATATGGGTCTACCTCACTGCTCCCATCCTTGGGGCACTG
TGTGGGGCAGGAATCTACTCTGCCGTCAAGCTGCCGGAAGAAGATGGTGGCATTCATGAAAAGCCTTCAACAACA
AGTAGCTTCAGAAGG**TGA**

>*Bpe*NIP6;1

MENEEVPSAPSTPATPGTPGAPLFGGFKAERSGNGRRSLLKNCSKCFSVDQEWALEEGTLPKLSCSLPPPPIPLA
KKVGAEFIGTLILIFAGTATAMVNQKTHGSETLLGCAASSGLAVMVVILSTGHISGAHLNPALTIAFAALKHFPW
KHVPMYIGAQVVASFCAAFALKGIFHPIMGGGVTVPSGSYGQAFALEFIISFNLMFVVTAVATDTRAVGELAGIA
VGATVMLNILIAGPTTGASMNPVRTLGPAVAANNYKAIWVYLTAPILGALCGAGIYSAVKLPEEDGGIHEKPSTT
SSFRR*

***Bpe*NIP7;1**

>**FXXK01000331.1:**40000-47500 *Betula pendula* genome assembly, contig: Contig330, whole genome shotgun sequence
>**Bpev01.c0330.g0006.m0001**
Chr Bpe_Chr1 34,485,381-34,487,761 (1) ::
join(34485381..34485563,34485825..34486049,34486688..34486882,34486981..34487042,34487584..34487761)

>*Bpe*NIP7;1
AGTTCTTGCTGACTGATGTGAAGTCGCCAGAAGTTCCTATCGGCCATTCCGGGAGGCTGCAATTTCCTAGCCACT
GCTATGCGCTGCCAAGAATTTCTATTGGCCGCCATCGGGCCGTCGAAAGCTCATGTTGGCCACTACAAGGTCACC
AAAAGTTCTTCGCTAGCAATAGTCGCGGGCTGTCAAAAGTTCTTGCCAACTACTGCAGGGCCTTTGAAAGTTCAT
GGCGTCAACTGCGGGTTCACCGAATGTTCATGGCAATTACTTTTTCAGAAGTGTTTTGTGTCTTATTTTTTGAAA
ATTATTTTTTTCTTTTAAAAAAGTTTTATAGCTAGTTTTGAAAAGTGAATTAATGGTGAGGGCTACTACATTTAA
AAATCTGTTTAAATAACTGGTATTTAAAAAGATCTTTTCCTGTTTGAGAAGCGAACATAGTTTTAGAAATGTTAC
CAAAACAGACCCTAAAATGTTGATGGTACCTAAGGTCTAAAGCCTTCAACCGATATGATACTTTAAAATATTTTC
GGTTAATATATTATGTATAATTTTTTTAGCTTTAATTATTTTTTCGAATTTAAATGGTTGAGATTTTCTCACATT
AATATTTATTACTTTTATTATTCCAGTAGTATTTAATTATCACCATAGCATTTATTATATTATATCATATTCTAA
ATTAGTAAATACTGATGTGACAAATTTTAATTGTTTAATTTTAAAGTAATGTCTGAGATTTAAGAATTATATATA
TAAAAATATTTTAGAAATTATTAGAGGATCTTGATCCAACCCTCAACGTACAACTCTTTTCTCTCCTTATATATA
ACTAGCTTATACTAACCCAACCCAATTGATCTAATAATATTATCCATCTGCAAAAAATAAGGTGCACTCTCAAGA
AATCAAGAGAAGAACAGGTGGCTGAGGTTTTTTTTTTTTTTCTAAGCAATATATGGAAGGGGAATTATAAAAGGG
TCTATTGCACTCTTGATCCTAAATTCCTAATAGATAAATTAAAAGAATGAGTGATCAACTGAAAATGTTTTCAAA
TACAAAGTAAAAAGCGGCTTATTAAGTAACCTGATATGCTCCAAATTAAGAACTTCTAGTATTAGGGATCTTCAA
AGCTTCCAACT**ATG**AAGGGAGTGAATTTGTTGTGAATATGTACAATGATGGGAGAGAACATTCAATCTGTGAACA
AATTGGACTTGTTGATGGCATGGATATTGATGATGAGAGAATTTCCTTCAAGCATAGCTGCACAAGGTAATTGCA
CCTATTAGAGACAACTAATTAATTAAGTTGCCAAAAGTGCTGCATGTGCCTCCCCTACAATATTAGCTTTAATAG
GAGGAAGCCGGTGAGAAGCTGCTAATTAAAATGATGTTTGTACGGTCCAGATGCGTCCTTTCGTTTTTTAAAACC
AAAACCATCACGCCGTTAACAAAACGGCAACACCAAAAAGTTAAAGTCTGTGAAACAAAACATAAATTGATTTTT
TTTTTTTCTTTCTTCTACGCAAACAGAGTCACGCTAATATTCAGCACTCCAATACTCTGTAAGTTCATAGGCTCT
CTCTGAATTTGGTTCTATGCTTTTCTTTTTCAGAAGTGTTTCCCTTTTATTAGTTTTTATGGATGCTATAGGTGA
AGTGTTTCCTTTTTGTTTTCTGTGTTGTCTGGAATGTGAGGTTATTGTTCTTGGAGTTTATGGTTTTTAGCTTTA
CTCTATCATTTTCCATCCAAGGATTTTAAAAGCACCTATAGCATCCATATTCCCTAAAAATTAATACAAGGGAAA
CACTTTTCTAAAGAAGAAAAGCACAGAAACCAAAATCAAAGAGACCCCATCAACTTACAGTTACAGTACTATTGG
CGTGCTGAATATCAGTTTCAAGCCTTCAAGCACTTTGAGAGAGTCCTTGAATGAGTTTCTCATTGAAGAAGCAAC
ATACATCTCCAAATCTTTTCATCAAAACCCCCAAAAATCCTGACTTCTTCTCTTGCTCTCCAAGACGAGCAGAGA
GTGTTGAAGGTCTGTTTGCGTAGAAAAAAAAAAAGAACAAAAGAACAAAAGAAAAAAGCAAGTCTGTTTGGCGTT
GCCGTTTTGTAAAAGGACGTGTTGGTTTTAAAAGGCAAAAGAATGCATCTGGGCCATACAAAAAAATACCGCAAA
TGTGCGGTAGTTTTCACTACCGCACATAAGCCGGATCCTCATCAGAGTAGCTAAGAATTGTTGTTGCGACAGCAT
ATATGGCTAGATCGAAGTTTGCCTTATAGAACCCGGGGAGGAGGCTAGGCTAGGGTTCCAAACTTCATTGCTGGA
GAGGAGTTACGCCAAGGTGAGAGATGGTTCCTTAATGTAAGGGTTATTTATTTCAATAAAGATGAAATTTATAGA
TAAGTTGATTCCTTGAAAATCACATATGATCCATTAATAAAAGCTAGCATGATTTGAAACTCACGGTAGTCCGGC
CCCTTCTTGGAATTAGGTTGTTCTTATTAATTATGAAATCCAAAATTGACCCACAAAAAATAATGTTTTTTTTTT
TAAAAAAAAAGGGAAAAAATAATGTTATTTGTGCATACATATATGTCTCATTTATATATATTATATTTACTTAC
GTTTGTTATGTCTGGTCACCTCACCCACTTATATTGTGTTTACTAGTCATGTTATTATGTGCCATCAAGTGTAAC
TACTTATTGTTAGGATAAGAGCCAGCTTAAAGGAGTAGGTAACTAAGGCCTCCGTTTAAGCGCCCCATGAAATAG
TACTGAGCATGCACATTACAAGAAAACAATTGCCAAAACGAAAAAAAAAAGGGGTAAAAATGAGTTTCAGCGATA
ATGTTTTAAAAAACACTACTAAAACAAGGATAAAAACGTCATTAATTATTTGTCAAATTTTCACTGGCGGTGATC
CGAAACATTCTTCCTTGGCACATAAATTATGTGCCAGGAAGTTGCCATACTCGACACCGGGAGACCTAGCATAAG
CTAAATGTTAGCAATTAAATGCGAAAAAGGTTTTCTTGGCGTTTAAATCATCAGTTATCTTAAATAATAAAGTTA
ATAGAAAATAACAAGGTCAATTATTTAATTGGCATTTTAAATATTCACACTCAAGTATGGACTAAACAACACATA
ATTTTTTTTTTCTAATTAATTATTTTAACTAAAGTAAAAAATAAACTGTACATGTCCAAACTTAAAATTTGAAGT
GGTAAAGACCTCCCTTCCTTTTAAAAAAGTTACATTGCCAAGAAAAGAATATGTAGTGCATGTACATCAATTTTT
TTATTTTTATTTTTTGTAATTACATAATATAGTGTACATAATTAATTATGACTTGGAAGGGATCCAGCAAGAGAA
GGAGGTTAAACCAAATAAGATTTGTTGGGGTTGGGGAAAAGATGGTTGATTTCTAAAATGAAAACAGTTAAGAAA
CGTCACCAACTTCAATTATCTCTCAAACTTCAACCCTACAAAAGAGCCAAAACTGTGTGAACAACAAACAACTCT
TCTATATTTAACCCCCCCTCTTGTTAGAACGCAATTAACCTAGCTAGAGTCAGCTTTTCTATTCTACATGTTCTT
TGCACCTCTTTAGCTTTCTGAAAATCATTAAAGACTCGGTATTCCTTCCTTGCGAGTGCATCAGAAATTTGATCA
TGAAAAGCTTGTTTGAAGAGCAACCACATCATCTTGATCACACTATAGACCATGCACCAACTAGCGGCCAATCTA
AAGATGATCAAGAAATGGGTGTTAATGCCATGTCAAAGAGTGATGTCTTGAAGAACTCACCCTTTAGCTGCTTTC
CACAAGGAATGGATCTAAATCTTGGACGCGTGGTAACAGATCTCTTACTAAGCTTAGTATGATTATCACTTGATT
TTACTGCATGAGCATGTGATGTCATTTCAAATCACACCCTGATTGAGTCAATGCTTCTGTAGCTTTAAACATTAG

GGAAAATTTTGCATATATACGAAGAAGTTTGATTGGAATGCCTTCAACCTTCATAGGAGCTGCTTCATAAAACTT
TTATTGACTCCTCCACTATAGACATTTTAGTTATCGTGCATGTGAGTGTCGTATTTACATATATGCAGGTTTTAG
CAGAACTGGTAGGGACTTTCATTTTGATGTTTTGTGTGTGCGGGATCATAGCGTGCACTCAGCTAAGTAAAGGCG
AAGTGGGTCTTCTGGAGTATGCTGCCACAGCAGGATTGACAGTGGTTGTTGTCATTTTCTCGATAGGCCATATCT
CTTGTGCGCACGTTAATCCTGCTGTCACAATAGCTTTTGCAACCTTTGGTCATTTTCCATGGTCCAAGGTACATA
ATTTTTCCAAATCTTCCTTCGAGATTTTAGTCATTTATTTTTCATTGCAGTTAAATATATATTAAAAAATTTC
AATCAATCACTTGAATTTTCATCACATTACAATTATACTCTTAAACTTAAAAACTTTCAATTTAATATATATCT
TTTAATTTTTTTTAAATCACCCATCCGTTAAGATTTTCATTAAATCTTAATGAAGGAGCGTCAAAATTTTCAAA
ATATCCTTCATTATTTTAGGGAAAAAAAAAAATTGCAAGGGTCTAAGCAGCCAACAACATAATATACACACAGGT
TGAATCTTTGAAGTTTATATATATAATATATATATATATATTATATTTTAAAAAATAGGGGTATTTCCTATTTAA
AAAAATAGGGGTATTTTAGGAATAATGACAGAATTTAATGGAAAACCCTAACCGCTCGAATGAAAAAAAATTGAA
ATTGATACACATTGAGAATTTTTAAAGTTTAAGAAATAATTAATAAAGAGGTGACAATTTAAAAAAATTGAGTGA
AGTTTTTCCTTAAATATTTCATACATTAAATCTCACCTATTAAAGAGGAGTTAGAGCATTTAGGCATTAAAATGG
AGTTTGTTTACTTATTGTGTGACTGTAACAGGTTCCGCTTTACATATTGGCACAAACGTTGGGTTCTGTGTTGGC
AACATGTATTGGACAATCTGTCTACGATGTGAAGTCGGAATTTATGACCACCCGGCCACTCCGCGGCTGCAGCTC
TGCCTTCTGGGTAGAGCTTATTGCGACTTTTATCATCATGTTTCTAGCTGCTTCAGTGACCTACCAACATCAATC
TGTAAGCATTTTTTATTCTCTAATTAATCTGTGAGTTGGATTAATTAGTTTTGATGGAGAGTCACGATATCCACA
TGATCAACTTGAAATATTTTGTAGGTAGGCCATTTGTCTGGCTTCGTTGTTGGCATAGCCATTTCACTAGCTGTG
TTAATCACAGGGTAGGTAAAAAAAACTCTTTACATGTGATTAGCAATTGCAAAGATCCTTTCGCGGTAAACTTTA
GAAAACCCTCATAACTTTCACGTATTTTCAAATGACCCCTTCAAAATTTTAAAACTCTCAATTTAAGCTATTAAA
GTTTTAATTTCTTCCCTCCAATAGGATTTATGGTGCCAAATTCACAAAATACTCATTTTTTTTAGGAAAAAAAAT
TATAAGAATTTAGGCGTTGGTCAGGATTTAACGGAATTTGTAAAAACATCCACGACTGAATCTTTGAAAAAAAAA
TTATAATTATTTTTTAAAAGAAATAACGGGTATTTTGTAAATTTTGACATGATTTAACAAAAAAATTTAATGGAA
TGGGAAAATTGAAAAAAAAAAAATTAAAAGTTCAACAGACTAAATTGAAAGTTTTAAAGCTTTGAAGGGATCATTT
TAAAATGCATGTAAGTTCATGGGGGTTTTATGAACTTTTCTGTTCCTTTTGTTCTCTTACTGGTTACTTTTCTTG
TATCAATTTTCTGGGTGTGCATTGTAGGCCTGTTTCAGGAGGATCAATGAATCCCGCAAGGTCATTAGGGCCTGC
AATCATATCAGGGAAATTCAATGACATATGGATATATATGACAGCCCCAACCATTGGAGCCGTGGCAGGTGCTCT
TCTCTTCCGAATTCTACGTCTTCAACAACGCCGCCCATGTAATCCCACTTCC**TAA**TTATTCCAGTCTAGTTGTTC
ATACTTAATTCGGCCCTTGGTTCAAGGCTTCAAGCTAGTGCACAAGAAAATTGTATTTGAAGATATTGCTGGATC
ATGTCAAGTAAATAAATGCCAAGTTTCGACTTCTTGGATGTCTTCAATGAGAAAGGTTGATCTTCTAAGTCTAGG
GTCACCTTAAGCTACAAACCTTAGCTAAATTGCAAATTCGGGTGAATTCCAAATAGTATAACAAGTTTTAAATTT
TGGGTTAAATCCAAGTGTCCATTTGATAATGTACTAAAAAAGTCATTAACCCTTAAAAAGTAAAGTAGCAATGTA
TAAACCAAAACTAATTTAAAAAGTGTTAGTTTTTAATTTGAGATAATTCGAAATTAGAGTGAACCAGTTGTATAA
TTTGTCAAACATCTTTTTTTTTATATAAATATAGAGAAATGCTACTTAACATTTTTTGTTGTCCATCTCCCAGCA
TTCTCTTTGTTTAATAATCCTCAGAAGATTCTACGATTTTCTTAAATTCTTAATGTTAATTTTAAATCAAACTGT
TGATATTAATTTTACCACATCAAAATTATTACTTTAATAATAGACTAAAATATGATAAATAGATTACAAATATTG
AATTGTAAAAATAATAAATGCTGATTGGACAAAATCAATGTGCAATTGGGCTCGACTAGTTCAGTGTTGACCAAT
GCAGCAAAATCCTTGTCCGCTGGGCTGGGCCTAACCCTAGTAATAGCCCACACTGGTTACCTTTAGGATGACAAA
ACGGCTTACCGTGTCGAGTTCGGATGAATCCCACAATAAGAGACGATAAGATGGTTCTGCGGAAGCTTGAATGTT
GGCCACTTTCGATGGTATTTGGATGGTGCCGTGGGTCTTGGGTTTCTTGGGCAAGCTTGAATCTTGGCCAC

>*Bpe*NIP7;1

ATGAAGGGACAACCACATCATCTTGATCACACTATAGACCATGCACCAACTAGCGGCCAATCTAAAGATGATCAA
GAAATGGGTGTTAATGCCATGTCAAAGAGTGATGTCTTGAAGAACTCACCCTTTAGCTGCTTTCCACAAGGAATG
GATCTAAATCTTGGACGCGTGGTTTTAGCAGAACTGGTAGGGACTTTCATTTTGATGTTTTGTGTGTGCGGGATC
ATAGCGTGCACTCAGCTAAGTAAAGGCGAAGTGGGTCTTCTGGAGTATGCTGCCACAGCAGGATTGACAGTGGTT
GTTGTCATTTTCTCGATAGGCCATATCTCTTGTGCGCACGTTAATCCTGCTGTCACAATAGCTTTTGCAACCTTT
GGTCATTTTCCATGGTCCAAGGTTCCGCTTTACATATTGGCACAAACGTTGGGTTCTGTGTTGGCAACATGTATT
GGACAATCTGTCTACGATGTGAAGTCGGAATTTATGACCACCCGGCCACTCCGCGGCTGCAGCTCTGCCTTCTGG
GTAGAGCTTATTGCGACTTTTATCATCATGTTTCTAGCTGCTTCAGTGACCTACCAACATCAATCTGTAGGCCAT
TTGTCTGGCTTCGTTGTTGGCATAGCCATTTCACTAGCTGTGCCTGTTTCAGGAGGATCAATGAATCCCGCAAGG
TCATTAGGGCCTGCAATCATATCAGGGAAATTCAATGACATATGGATATATATGACAGCCCCAACCATTGGAGCC
GTGGCAGGTGCTCTTCTCTTCCGAATTCTACGTCTTCAACAACGCCGCCCATGTAATCCCACTTCC**TAA**

>*Bpe*NIP7;1

MKGQPHHLDHTIDHAPTSGQSKDDQEMGVNAMSKSDVLKNSPFSCFPQGMDLNLGRVVLAELVGTFILMFCVCGI
IACTQLSKGEVGLLEYAATAGLTVVVVIFSIGHISCAHVNPAVTIAFATFGHFPWSKVPLYILAQTLGSVLATCI
GQSVYDVKSEFMTTRPLRGCSSAFWVELIATFIIMFLAASVTYQHQSVGHLSGFVVGIAISLAVPVSGGSMNPAR
SLGPAIISGKFNDIWIYMTAPTIGAVAGALLFRILRLQQRRPCNPTS*

SIP – Small Basic Intrinsic Protein

***Bpe*SIP1;1**

>**FXXK01000083.1:**21000-33500 *Betula pendula* genome assembly, contig: Contig82, whole genome shotgun sequence
>**Bpev01.c0082.g0002.m0001**
Chr Bpe_Chr8 23,172,529-23,181,063 (1) ::
join(23172529..23172825,23173422..23173694,23180914..23181063)

>*Bpe*SIP1;1
CTCTTTAGGATAACTCTTCTGCTATCAGCTAATTACTTCCCAGTTGGAGATGTGAGAGAGAGAGAGAGAGAGA
GGATTTGGCAACAAATCTAATAAAGAAGAAGATGTCAGGATCTTTTCAGTAGAAATACAAGCTTAGCTGGTTACC
TGTTTTATCTCATCGCAGTGGATTAACGGCTATATGCTTTTGAAACTACAACAGAAAAAGTAGCCGTTGTAGTTT
TCAACGGCTATATTTTCAAATACACGTTCTTATCCTTCTACTTTTTTTCTTTTCTTTTCTTTTCTTTTTTTTTT
TTTACCTGCGTTTAAATTTTAAGTGCTCGTTGGGTCGTTGGGCTGTGCAAACAAGCGGAGCGCCTGTGGACTATG
GGACTAAGAGTTACCTGGGCCAAACTATGGCCTAAAGATAGAACTCTCAACTCTACGTGGGTCAGTTGCAACCTC
TAAATTGATTCAGGTTGGATAAAGAACATACAAGATACCCCAAGACCCATCTATAAAAAAAGTTTAAAAAAAGTA
AAAAAAACTAAATAAATAAAACAAAAAAAAAAAAAGAAGAAAGATACCCCAAGGCCCGATTGATAGGGAGTCCACT
CTATTTGTGGTTTCCTTCTATTATATAACCCTTTTTCAATTTGCTTAGAAAAATAAAAATAAAAATAGCCAAGAC
CCCAATAAAATTCCAATTCATTTTTTTCTTTGTTTTCTTTATGTCTCTTCACTATTTTTAGTTGGTAAAATTAAT
CTTTTAACTTTGATGGATGGAACCCAAAAAAAAAAGGGGGGGTGAATTATCTTTTTGGAATAATTACTTTCTTCCA
ATTTCAAAAAGGAGTAATTATTTTTTAAAGTTGAAAAAAATGGGTAATTATCTTTTAACCCTCTAAACATTCATT
ATTTTTGTCATTATTTTTCAAAATTCAAAAATTTTTAATTTAGTGTATTAAACTTTTAATTTTTTGCAATCTCAC
CTTTCTCGTTAGGGTTTGAGTTAAATCAGACGTCAAAATGTAAAATAACCTTTATATCTATATAAATTTATAAAA
TTACAAAATTATCTTAATTAAATAATCATTTTTTAGAAATAAAAAATATGAATAGTTTGAGAATTTTTAAGACCT
CAATTAGGATTTAACAAAAAATACTAATAAAATGGATGACATTGTAAATTTTTAAAATTAGATACACTAAATTAA
AATATTTTAAATTTAAAGAGTGATTACAAAATTGATAAAAATTCAGGTGTGTTAAATAAATTTTTCCAAAAAATA
ATTAAAAAAAAAAAAACATTGTGTAACTCTATCTGTCAATTACAAAAACGGTCAGCCAACAAAAAAGACAAAACA
TTGAAGCTAGAATACTCAAAAGAAAAATAGCAAATTCAAATGTAACTGTCTCAACTCTCGATGTCTTATCTCTTT
GAACTTTCTCTGTTGTTAGCAATAAAACTAAATTCAAACTAATAAAAATGATACATATCATGTTCAATTTTTTTA
TTTTTTATTTAAAGAATGGTACGTATTAATTTATTTACCATTGGTACGAAGTTAGTTAGTTAATTAATTGTAGCA
GTTTGGTGGGCAAAAATGTCCCTACTGATTTAACCATACGGAGCCCTACTTCAAATGAAGATAGCGAGATAAATT
TTAGCAAGTACATACATGTATACTTAGCAAAAAAAAAAAAAAAGGGCACGTATGGTGTTGCTTTCGTCTCTGCCCG
ATTGCCACTACGGACAATACATATGCATATATAGATAAACAAACAAATAGAGGCCAATAAGTCAAGGCAAGAAAG
AGGAGGAGAGATCGGTGGTTGGTGGTGATAGATAGATCGAA**ATG**GGCGCGATAAAGGCGGCAATCGGAGATGCAG
TTCTGACATTCATGTGGGTGTTCAGCGCATCCACGCTGGGGGCAGCGACTACGGTGACAGCCAGAGCCCTCGGAA
TTGAAGGGCTGGTGTGGCCTTCTGTCTTCATCACTACCGTGCTTGTATTTGTTCTCGTCTTCTTCTTCACCTTGA
TCGCTAATGCCTTGGGTGGGGCCAGCTTCAACCCCACCGGCACTGCCTCCTTTTACGCTGCCGGCCTGGGTCCCG
ACACCCTCTTCTCCATGGCCCTACGCTTTCCTGCTCAGGTCTTCTCTTTCTCTTTTTCAATTTCACAATTTCATC
TAACAAATAACATAACATGCATGTCTCCTTTGTCTCTTTTCATCTTTTTGTTTCTCATATAGATCTGCTATTCTT
TGTGTATTCATAATTCTTATATTGATTGACTAAAAAATTCGCTGTTTCCTTTTTTTTTTTTTCTTTTCTTTTTTTT
CTTTGCTTTTTTTTTCTTTTTTACATGCACTTCGCTTTTGTTGGATTTCTCATCTGTTTATTTATGCAAATCTGCC
CCCCTACTTTTGGCATGCCGTTTTTTTTACGGATGTCTGCAAGCCAATTTTACAAAATGCCCTGCGCGAACCTTT
TTTTCCCCTGTCGCTCACTCTCAGTCTTTACTTGGTTCTTCTCTAAACCTCTAAGCATGACCATGACATGCTGTT
CGTACTTAGCTTTGTGAGGAATACAAATCTTGCTTACTTATAGTTTAATTGTTTTAATTTCCTAAAGGAGAACCA
AAATTACGCAGACCATTGACAAAAAAGGAAAAGAAAAGAAAACAGAAGAAGATGCAAATGTTTAGGTTGTTGAAC
AAAAACATTAAAAATACTATTGTCTGCTTGATAGGCAGCCGGTGCTGTGGGTGGTGCTCTGGCAATCATGGAAGT
GATGCCCAAGAACTACAAGCACATGCTTGGTGGGCCTTCTCTCAAAGTTGACTTGCATATTGGAGCCATTGCGGA
GGGGGTGTTGACTTTCCTCATTAGCTTTGCTGTCCTTGTAATCGTACTCAGGGGTCCTCGTAGCCCGATACTGAA
GACATGGTTACTTGCCATGGCGACAGTGGCATTAGTCATTGCGGGTTCTAATTACACTGGACCATCCATGAATCC
TGCCAATGTGAGTACATCCTTGCACCTTGTATTACATTCTCATGGCTGATTTTTTCACTGGTTTCTCTAAAAGTT
CCGTTATATGATTGTACGTCCTTTCTTTAAGCAATGTGAATGATATACTTTTCTTGACAACTATGCAATTAAGCC
TTTTCTCTTGTGTTTTTTAAGTGTTTTACTTAACATCCTCCTCTTCCCTCCTTTCACTTAGGTCATCACTCTTTA
ATCCAAATTTTATCAGATGATTACATTTAATATTAAGCAAATTTTGTTGTATATATGTTTGTTGGAAACAAATAT
GGCATACTTTGTTGTTACATGAAATGCATTATATCCATTGCTAATACTTAGTGGTACGCTAAATGTGACAAGGGA
TTAAATAATATTAGGACAGATTCAATGAATACCTTGTAGTTTCTTCCTTACTTTTTGTTCTTTTGCATGGCAGCA
CTTTTTGTCTATCTTTAAGAATTTACAAAATGTATCATTGATGAAACACATTGGAATAAAAGCATACTATATCTT
CTTGTCTATCTGATAGAGTAATAAATGTGATAAAGCCAACAATAATTTGGAGATAATCATTGACAAAGGAAACCT
AGCCCCTCTTTTATCCAACTGAATCAAGATCTAAAAGGGGAAAAAAAAAAAAAGAATCTTGGTGTCTACGTATTTT
TCCATTTCTGGTTGCCTTCTTTTCTCCCACTTATCTCCTTATATTATCTAGAACCTTTGTGAACTCACCCATCAT
AAAGTTTACGTACATTTTAGGTTGTTTACCTGTGCAGCACTTTAGCTGTCTTGATAGCTGGCCTGGTGTATGTGT

AAGGTTGGCAATGGCTGGACTTTTTAAATAACTACCATATTCTGCCCTATTGGGAAGGGAATGGTAAAGAACTTT
TTAAGCACTGATAGCGTGAAGTTAATGGGCTAGGCAGCTGTGAGATTTTAGTTAGTGACTTCCAATGTGTGTTAG
GTTCAAGATAGGGTAGGGTTTGAATCAGTCCTACTTGGCATACCATGGATTATAAGCACGTGTTACATAGCAATT
ACTTAAGGGCTTTCTTTAGAAAGTTCTTAGCCTTTAGAATTGTTAAGTACCATTGGACTTTTGTGCAAAGTTGCT
TTGCTTAGATTGACTGCTGCTTTATTTACTGATTCGACTGGAAGTTTCTTTTATCAACTTTCTGATATGGGGTGC
GAAACTCATCTCCAATGCCTGACAAGACAAGGCCTGTTGTGTAGAGGGATAGGCTGGCCCCACCTTTGCCCGTTT
GAAATATACTTTTATTATGTGGGGAGGATGTTATACAAAACTTCTAGCATAAGTTTTCTCTACAAATTGCCTCAC
TCATCATGTGAAAAATTTGTGGGCCAAAGAAAGATTATTGCCTAAAGCACTCACTCTTTCTTTCTAGATGAACCA
AGTAGATAAAGGAAGAAATTAGTATCTTCTCCTTTCCCTTCTTCTACCACAAATATGGGCTACAAAATTGAGTTC
TTCTCAATCCTTTTACATCACCATAGATCCATACCATGGAGACGGTTTTGATATTTTTGGGTTTGAAGGGATCCA
GGGAGATGGAAGTGTGGCAAAATAATATTATATTTTCTGCTAACCAACAACCAAGGAGAAATAGTCTTCGGTCTA
AGCGGAATGAAGATGGTGATAGTTTGGGGCATAGAGTCAATAGTTGGTGGCAATGGTGATGGTAGAATGGTGGCA
ACGGTTGATGATGATAGGTGATGATGGTAATGGTGTTGCTGTTGGCGATGACTGTGAGTGTTGACACTGATGGCA
ATAATGATAGCAATAATTGCAGTGAATAGGGGTAGGTGAAAATGAATATGGGAGCATTTGGTATAACTTTTATTT
AACATACTCTGTTAATGCCATCCTACCTATGGAACTTCTTTTTTTGAGATTTGTCGGGGAAAGGGAGGGGTGCAG
GGGGTTTGTAGTTTCAAAATTGAGTGTAGTAAATATAAGAACAAATTTCTAGAGTTACCTTATTGGATTGTGATC
TTAGGAACATTGAGGAAATTAAAATATGCTTATTGGAAAATATTTTTATCAGCAAATATAAGTCTGACCTAGTTG
AAGAATTTGGACCTACTGTTGATGGGACAGAATTTAAGGAGTCTCACCATTACTTTTTTTTTTTTTTTTTTTTTT
AAGTTTATGTAATATCTCACCCTTTTGGGACTTAGTTGCAGGAATTTCAATTCTGAGGTTGCTACCTAGATGCTT
GCTAAAGAAGCAGTTCTCTTGCAGAAGAATCATTGGGGCATGACAGACACTTGAATCAGATTGAGAGACATTTAG
GATGGTGAAGGAAAACATTTCCAATTTCAGATTTGTCCATGAATGGAGAAGTGATGAAAGACATGTTAGTTCCTA
AGTTTAGCTTAAGAAGATCATAGTACAATAACAATCATCATAGTACAATAACAATCACTAAAATCTTGATCCTTT
ATTCAAATTCAGAAGTTTAATGCAACCAAATCTTTATGGCTAGGAGTTTTCAAATTTCAACCATCCCATTTGCGT
AAACTCTTCTATAGATCTTCATATTACCTGATTGACGGGTTTAACCATTGCTTTAAGAGGTGGTTTTATGGTAAG
GAGTTGTCTCATAAGGATCAATAGAAGGAGACTTATCTAGCTCGTGTCATTTTGGGTTCTCTGGGGCTAGAGTTT
GTGAAGTTAAAGGAAACTTTGCTAGATATGCTATATACTATCATATATATATATATATATGTTTGTACCTCTCTT
TTTCATCTACGTTTTTCCCATAAATATAAGTGTTTGAAATTCTAAATTCTTATACTCTTCTTTGAGCAAAATTTG
AGCTATAGATTAACCTCATACCCAAGTTGTACCTCTTCCCTTTTCTGTTTGAGAGGTCTTGGAGTCTAGCCATGG
AGGGGTGGATTTGGGGACGGTTAAGGCTATTGCCACAGTGTAAGCCTTTGGTATGTAATGTTATTATTATTATTT
TTAATTTCTAGAAGCATGTGAGGCCATCCTCCTAAAAAAGATTCGTCTTGTTAATTGTAATACTCAATTCGCACA
TACGCCAGGCACCTTTTTTAGAAGAGCAAAGTGTCTAATTAATGATAAAAACCCTGATTTAGGCTCTGCACTTAT
AGCAAACCTTCTATATTTCTGAGACATGTATAATTGGAATATGCTTTGGATAACTGACTGTTGCAGGGCCCACTT
CTATTCAAAGAGTAATGATGGTTTGTCTTGTCCACAGGCTTTATAACCATTCTTTGGGTCCTTAAGCTCTGCAGT
GTTTTTTTTTTTTTCAAGTTCTGTTCCAATTTAATTGCATCATGATGCCCCTGATGCAAAAGATATGATGTTGGT
TTTTTGTTTTTTCTCCCTTAAAATATCTTTCTTTCCGAGCTTCTTGTTGTGTGCATTGCATTATTTGAGAATTAA
GTGCGATTAAAAAGTTAAAGCTTTCATATCTTTTCATGTACACCTTCTAGGATTGTGGCACTATTAGGATCAATT
TCTATGTCCCTTTACGCTATTCAAAGTTGGACATTGATAGAACTCATAGAGACCAATCTAGCTCATTGTTGCTTC
TTGCCTTATCGGAAAAGATGCAGATGTTTTCTACTGAGTTTGCTACCATCTCGGGAGAGGTTTAATTTTGTCAGC
TCGGCTAGGATAGGTGATTCTGCCATTTTCACTTGTGGCTTTAAAATGTTGGCATGTGTCCTTTGAGGAGCATCC
ATTTCTTTGTAGAGACCTCTTACCCTCTTACTGCAACGAAGACTTCTTTCTCATGGGTTTGTATATGTAATACAT
AAATCTAAATTTTTTTTTTTTTTTTTTTTGGGGGGGGGGGGGTGTGTGTGTGAAAGAGGGATCTCTCTTGAGGACT
TTGGCTTACGATTTTTTTTTCCCTCACAACTTGTTTAGTTTTCTTCTTATGAGGATCTCTCTCTCTCTCTCTCTC
AATTTGTTTTCAATAAAAAGAGATGTTTGTCATGCATGATGAAAGAAAATGCTGACAGGCTCCTTATTGACTATC
CTTGGAAGTTGATCTTTTGGCTTTGCAGATTAAAGATTTGTCCAGGCTTGAGCTTCTTCTTATTCTTCTAACAAT
CTCCCTCAATTAGTTTCTCTTAATATTCTGAACAATGTTATGTGCAATACTAATTGCTAACTCTTACTTAGTAGA
GTTCCATCAGTAAATGAACTACAATAACGAACTTTGAAAGATCTTAAGCCGTAGAAGCTCCTTAGACTCAATAAG
TTACGCAAAGTTCTTCTGTTGTACTATAACTTGCCCATAAACTATTTTTCCCGCTGTCTTTGTACTTCATCTTCA
AGATCTCTAGAAAAGTAAAGATGCCAAAGTAGTCTGTCATTCTACTTTGTAATAGAGTATCATACCATGGCTGCT
ACCATGCTGAAATTGTTTTGCTTGATCTTTTTGCTGACTTTGGGATTTTAACTTAGGAAAAACTTCACTTACCCC
CTCTAATTACCATGCGATTTGCAATTTCTTCCTAAACTTTAAAAATTTATAATGTGGGCTGGGTTGGAAACACCC
CTGCCTTTGGGGAGTCACGAGCCCCCACTAAAATTTTAGAAATACTACAAAAAATTGAAAATTTCTTCTAACTTT
TATGTATTTTTATGAAATGGACTCCCCAAAATTAATTTTTACCTCCAAAATTTTTAATTTTTTAGTTTTGTCCTC
CCCCTCCCCTCCTAATATTCAGTTCCGCCCTTGAATGTGGGATTCTAATCTATCAACCCATTTCAGTTACTCCCT
ACTATTAAATTTTTTCGTTAACTTAGACAACTAACATGTATAATGTTTGTTAAACCCTGATAAAATTTATAAAAT
ACAACTATGCCTTAAATTAACAAATAAAAAATTTAGAAAAACAAACAAAGAAACTAAAAAGACAAAAAAAAAAAA
AGTAACAAAAATCATTTTTTTTTTTTTTAAATAAACTGATCCACGGACACTCTATGGGTCAGCAAACCCATGGCGT
GACCGTGTGTCACCATACCCACTGTGTGGCTGTGGTTCATGCTAGACCGTGGACATGCTGTGGGTCACTCAATTT
TTTTTCCCATTTTTATTTAAAAAATGGGCTCTTTTTTTTTTTTGTCATTTTTACTTTCTCCGGTAGGATTTAGAGT
AATGTTAAATACTACACTACCATCCCACATTCATCCCACTATGCTGACGTGCAGGGCCAATTCTACCCTTGGCAA
ATTAGGCGGCCGCCTAAGGCCCCTTATTAATAAATATTAATGAAAATTTATTCCATAAAAAAATATTTAAGGCCC
TAAGAATTGTCAATATTCTCTAATTAAGGCCCCTAATTTGATAAAATCCTCAATTAATAAATATTAATGAAGATT
TATTCCATAAAAAATATTTGAAGACTTAATTATAGTCAATATTCTCTCGCCCAAATTATGGTAAATAAGTAATTA
AGAAAGACATTAATATCATTTGATTCTTTTAGAAACTTAAATCTCCAACATTCCATTCTTGAAACAAGTCTTAAA

AAAATTGATAGCATTAAATAAGGAATCAAATAATATATTAATATTGAAGTCATTCTTAAATAAAACCCATTCTGG
GGTAGAGTTTTTTGACTTTGTGATGAGCAGCCAAATTTTCATCATAATTTACAAGAAGCAGCTAATTAGCAATA
AATTATGATCTCAAGTCCCAACTGCCAACTGGATCCTTATTGTAAGATATGATTTTTTTTTTCTTTTCTTTTTT
TTTTCTCTAACATTGGGTTGCTTCTATCCACACGCAACATTCAAAAAAAATATAGAATTCATGATTGGAGTCCAA
CGTTCAAAAAAAAAAATTAAAAAAAAAAAAATTGAATTGAATTAAATGTTAAGAAAATGGAAAGCCTCTAAAAAT
ATTTTATTCAGTTTCCATTTTTTCTCTCTATTTCTCTATTTAAGACTTATTCAAATTTGAATATAATATAAAATT
AATATTTTTTTTCTAAAAAAGCCTCACTTAAACTTCTCGTCTTAAGCCTCAAAATGTATTAAGCCGACCCTGCT
GATGCGGCATTGTCAATCTATCATTGAATTTGTTTTTTTAACAAAGATTGATCCAATGGTGAATTGGCAATGTC
ACGTCAGTATAATGGAATTGTAGTGTAGTATATAGCATTACTTTAGAATTTAATAGAAAATCTTAACAGAGAGAG
TAATTGAAAGGGGTTGATAGATTAGAACTCCACATTGCAAAATGCATAGTAAATTAGGGGGTAAGTGAAGTTTCC
CTTTTAACTTATTATTGAATTCCTACTCCCGCCAATGAGCTCTAGCTTAATGGCACCTCCTCGCCCTCTAAGAAC
AAGAGGGTAATGTTGTGAGTTCAAGACTCATTGTGTGCGTTTGAGTTATCAATTTAAAAATATATATATATATAT
ATTGTCAAGCATTAACGATAAATGCCATCTTGTGATGGTGTTATTATTTTATCATTCCTGAACACCCATTGGAAT
GTTTTTTGTTTTTTGTTTTAATGATATCCAGCACACTTTCACGCTTTTCTACTTGATAAACTTTCATTATATAAC
TCTTAGAGTTCTGTTGGTTTTGCATTTTACCGTTTTATATCAAAGGTTGAGCTTCTTCAATTGAAAATTTCTTAT
ATTCTAATAATCTGCATGATTTTGCCCTGTTCATGTAAATAGCAGGTTCTTATTTCAATTTGACTTCCTACTTAT
CATATACATGCATACATACTATATATATACATGTGTGTGTGTGTAGAGAGATAGACAAACTTTTTGCTTTAGTAA
ATGTGGAACATAGGTAACTGATTTTCCAATATGAAGATGTGTGAACTGACCTAGAAAACTTGTTTGCAGTAATTG
CATCATTTATTTGCTTTCTTCTTATCTTTTGTTAAGTTAACGCCTGTGTAGAGATCTTTCAAGTTTTTCTGATTA
CTCCATGCTTGTATGGATATCTATATATGTTCACAAATGGAAGATTAAGATTTGAGGTGAGTTTTCTACTAAAAT
TATGGCTACTGAGGGTCTATATCACAATATTTCCTGACAATGGAAGCAAAATGGAAGGCGGGAGAGAGAAAGGGG
GGGTAGAAAAAGAATAATTTTTCTTGAACCAAAAAAATAATTTCTATTTTAATGGAAGCCTTATGGGATGTGCGA
ACTTTTACCATGTATTGTCTCTGCAGGCCTTTGGTTGGGCATATGTCAACAACCGGCACAACACATGGGAGCAGT
TTTATGTGTACTGGATCTGCCCCTTCGTTGGAGCAATATTGGCTGCTTGGGTCTTTCGGGCTCTCTTTCCCCCAA
CCCCAGCAAAGCAGAAGAAAGCC**TGA**AGAGGCACAATATCTTATCTGCTCGTCCATTTAGTAAATAAGCTTGATG
TTAGTAATAAGGACCTTTCTTTTTCCTTTTCTTTTCTCTCTTTTTTTTTTTTTTTTTCCTGGTGCTACCTTACT
AGTAGTATTGGGACTCTCTTCAGCTTATGGATCAATAATATAACCTGTCCAGAAATTAATATTTGCAGCCTGTAT
GCGAGGTTTGTCATTGACTAGTCCGTTTTTGTTGTGATGGTGATATGGTCTACTTTCATTTTTTTTTTTCTTTTC
TTTTTTATGGGCAAGTAGAAAGAAAGAAATTTAGATCACGGGGTCATGACTATCAACAAAGGGAAATAGAGAATG
CGGGTGGAGAGGAATGGAAATGTTTCTAGTAAAGTAATTGAAAGGCGACCCACCCCTCTACAGATAGCATGTGGT
CTGCTTTTTAAGAGAGCTACTTGTTCTCTATTCTACGTCTGTATTAGTGTCCTTGGTTCATTATTGGATTTCTGT
AAACTGTGGGATTTAACTCAGAAAAATAGTGAATGATATGCATGTACACACCCGAATGTTGCACAAGATAACTTG
GTGAGATGTCTCTGTACGGCCACGCGAATTGGGGTGGTTACTGGCTAGGGTACTATTCAAGGTACAAGGTTGCTG
GTTTGTAAGGCACACTGGACAAGCCTGAGAATTGTACAGGTGCATTTGATGCTGCATTCATTTACATTTACATT
TTCATATCTCATGGTTATGAGCTTTTTGCTTTGGCCTCATAAGTTTTCAAAAACATGGTATGATTTTTGTGGAAA
ATTCTAACCTAAAACGGATGATATTTCTGTTCAAGTATTCCAAGATGCACTCACTCTTTGTTTTGATGGGTCCAG
AATATGTTTATGGCCCGGAATGAGGAATGAGGTTATAAGGGGGATCTTTATCATTCTTGATTCGAAAGGAAGAGA
GAGTGGAAGATTCAA

>*Bpe*SIP1;1

ATGGGCGCGATAAAGGCGGCAATCGGAGATGCAGTTCTGACATTCATGTGGGTGTTCAGCGCATCCACGCTGGGG
GCAGCGACTACGGTGACAGCCAGAGCCCTCGGAATTGAAGGGCTGGTGTGGCCTTCTGTCTTCATCACTACCGTG
CTTGTATTTGTTCTCGTCTTCTTCTTCACCTTGATCGCTAATGCCTTGGGTGGGGCCAGCTTCAACCCCACCGGC
ACTGCCTCCTTTTACGCTGCCGGCCTGGGTCCCGACACCCTCTTCTCCATGGCCCTACGCTTTCCTGCTCAGGCA
GCCGGTGCTGTGGGTGGTGCTCTGGCAATCATGGAAGTGATGCCCAAGAACTACAAGCACATGCTTGGTGGGCCT
TCTCTCAAAGTTGACTTGCATATTGGAGCCATTGCGGAGGGGGTGTTGACTTTCCTCATTAGCTTTGCTGTCCTT
GTAATCGTACTCAGGGGTCCTCGTAGCCCGATACTGAAGACATGGTTACTTGCCATGGCGACAGTGGCATTAGTC
ATTGCGGGTTCTAATTACACTGGACCATCCATGAATCCTGCCAATGCCTTTGGTTGGGCATATGTCAACAACCGG
CACAACACATGGGAGCAGTTTTATGTGTACTGGATCTGCCCCTTCGTTGGAGCAATATTGGCTGCTTGGGTCTTT
CGGGCTCTCTTTCCCCCAACCCCAGCAAAGCAGAAGAAAGCC**TGA**

>*Bpe*SIP1;1

MGAIKAAIGDAVLTFMWVFSASTLGAATTVTARALGIEGLVWPSVFITTVLVFVLVFFFTLIANALGGASFNPTG
TASFYAAGLGPDTLFSMALRFPAQAAGAVGGALAIMEVMPKNYKHMLGGPSLKVDLHIGAIAEGVLTFLISFAVL
VIVLRGPRSPILKTWLLAMATVALVIAGSNYTGPSMNPANAFGWAYVNNRHNTWEQFYVYWICPFVGAILAAWVF
RALFPPTPAKQKKA*

***Bpe*SIP1;2**

>**FXXK01000388.1:**89824-92655 *Betula pendula* genome assembly, contig: Contig387, whole genome shotgun sequence
>**Bpev01.c0387.g0011.m0001**
Chr Contig387 91,110-91,829 (1) :: 91110..91829

>*Bpe*SIP1;2
ATTGAATCCCCCGGCCCAAGTATTAGATTGGGTCAAAGTCCACTTAGACTTATTAGTGCTTGATGGACTGCATCA
AAGCCTGAACTTGATAAAGTTGTAGCTTCGGAACCCAAGCCTAGTTTGAAGGTGGGCTACTCTCTTCGTAAATGG
CTTCAATCAAAGTCATCAAAGAACCAAAAGGAAAACTTTACAGAGTGTAGCCGCAAAGTTTTTTTTTAAAAAAA
AATTTCATATTTGTAAAAAATTTATAAGCCTTAAAAGGTGATATGTGTCCTTAACATGTGAGAAGTACATAATTT
TTTTAATAACATGTGTTTCTCATATTTTTTTAACAGCATTAATAAGAAATCACGTGCTTTTTATATGTTTAAAG
AATATATGTTGCTTTTAGAAGCTGATTTGTAGGATTTGTAGAAAATTTCTGTCATTTTTTTTGTTTTTGTTTTTG
TTTTTTCAGAAGACTTAGTTGGAAGGTATTTATTCATCTCAGTTTATTAAATTAACATTTGTTCTTAAAGCATGC
AATTTTTACATGTATTTTTTATTCTTACATCGATCAATTACATAAATTTTTTAAAAATATGTCAATTTAATAAAT
TAAAGCGGAAGAATTCTGAACAACGTATTTTCTAAGTCTGAACAAAGTCTCCGAGGCATGAACGATCTACTTATA
AGACCACGCATGGCATGGGCTTGAGGCAAGCAAATTAATTAATATTGACAATTATTTTATAGTCAAGATTTAGGT
GATATTATATTAGCAAACCATTAAAAAAAAATTAAATATAACAATATTAATCTTGACCATGAATCAAATGAATCT
CCTCCATTTCCTTCGAACCTTTGGTATGACTACGCCATTTGACAGACAAGTTTCAACCGTCAATTTAAAGACACT
TTTGATTGGCTATTGGCTATTGGCCCGTCACAGTACCCGGCACTTTTGCTCTACCATTAGCTCAAAAAATATCTA
CACGCACCTCTCTCATGTTTTTATTTTTTGGTAGGTATTAGGTAAGCACGTGAAAAAGGACAG**ATG**AGGCGGTCG
TTCTCAAGCACACAAAAAATGGCCTCAAATTTAAGCGTATAGGAATCTACAAAACCCACAAATATAAAAACAATT
AATTACACGCTTGGTGGCCCGTGCATCTCTATTTTTTGTTTTTTTATTTTTTCTTGGAAAGAAATGCTGAAATTC
CACGTCAGTAATTACTTTGTTGCAACTCATTATAGAAGAGGACAGAAGAAGATTGAGAGCAAAGATCAGAAGAGT
GGGCCGGCCGGATGGGTCTGATGAGGGTGGCGATCGGAGATGCAATATTGACATCGTTGTGGGTTTTCAGCGCGC
CGATGATGGGAATCCTTACACCCATCATAGCAACCTACCTTGGCGTACAAGCCAAGTCACTGGCAGGGCTCTTCA
TAACCACCCTCCTCGCCACCAGTCTTGTATTGATATTCCAGTTGATTGGCAAGGTCCTGGGCGGTGCCAGCTTCA
ATCCCTCTACCACTGTCTCATTCTACGCCGCCGGCTTAAAACACAACTCATCTCTCATCTCCATGGCCGTGCAGT
TTCCGGCTCAGGCCGCCGGCGGGCTGGTTGGCGCCAAAGCTATACTCCAAGTGCTGCCAAGCCAATACAAACAGA
GGCTTAGGGGACCTTCCCTGAAAGTTGACTTACTAACCGGAGTCATTGCCGAGGCGGCGTTGACTCTTGGGCTTA
GCTTTGCCATCCTTTTGATCATGCTCAGGGGTCCTAAAAATCCACTTGTAAAGGTGTGGTTGGTTGCTGTGGCGA
CTGTGGGATTGGTTGTTGCAGGGTCGGGTTACACGGGGCCTTCGATGAACCCGGCCAACGCATTTGGGTGGGCAT
TTGTGAATAATCAGCATAATACTTGGGAACATTTTTATGTTTACTGGATTGGTTCTTTCACTGGAGCAACTGTGG
CTGGTTGGATCTATCGGTTTCTCTTTGTTCTCCCAATCAAGCAGAAGAAAGCCTGAAGAAGCATTTGGATAAGCT
AGCTAGCTAGCTAGCTCCTACTGACTATGGCCATAAGTTTTTTCTTTTTCTTTTTTATTCTCAATGTTAAAACAT
TAGTAAAGTGAATAAATTTGCTGTTTCTCATTAGTTGCATTAAAGCGTATATTAATGGTGAATTCGAGCCCTGAC
CCTACTTTACCTCGAATTCGGAAGTTAAAATT**TAA**AAATATCACGCGTTAGGTTTAGTATTATTGAGGAGGGTGT
GACTCACTTACATCAAGGAAAATGTTTAAATAATTACATTCAACATTTTCACTATGGCTTAAGAAACAATCAAGA
GAGCAATTTTTATAGAGGAATGCTAGGTGTACAAAGGTTTTTGTACAAAAAATTTCTTACAAAATGAGGTGGCACT
AAATGATTGAGTATAAGAGAAATTCAAATTTTGAATTATCCAATTATTTAGTGCCACCTCATTTTGTAAGAGTTT
TTTGTACAAAAACTTTTGTACACCAATCATTTCTCATTTTTATATGGTCTTGGTACAAATCAAGTCTTTTATACC
ATTCAATAAAAGAGTAAACATCATCTTGAAACACAAAAATTAAATGCTACCAAAACATTAAATCTCCCCTCTTCA
ATCCCTCATCAACGGAAAAAAGTTTTCTTTCTCTCACTCATTTATCACTCTCTCAACTTTCTTGAGAGACAAACA
ACCATTGCCAGAGCAACCCAAGTCGGATGCCTCTGCCACTGCCGCCTCCACCACTACCACCGCCCAAAAAAACCA
TCACCACTGTCCAGAAAAACCCCACGGTCACCGCCTAGGCCAAATCCCATGGCCACA
>*Bpe*SIP1;2
ATGAGGCGGTCGTTCTCAAGCACACAAAAAATGGCCTCAAATTTAAGCGTATAGGAATCTACAAAACCCACAAAT
ATAAAAACAATTAATTACACGCTTGGTGGCCCGTGCATCTCTATTTTTTGTTTTTTTATTTTTTCTTGGAAAGAA
ATGCTGAAATTCCACGTCAGTAATTACTTTGTTGCAACTCATTATAGAAGAGGACAGAAGAAGATTGAGAGCAAA
GATCAGAAGAGTGGGCCGGCCGGATGGGTCTGATGAGGGTGGCGATCGGAGATGCAATATTGACATCGTTGTGGG
TTTTCAGCGCGCCGATGATGGGAATCCTTACACCCATCATAGCAACCTACCTTGGCGTACAAGCCAAGTCACTGG
CAGGGCTCTTCATAACCACCCTCCTCGCCACCAGTCTTGTATTGATATTCCAGTTGATTGGCAAGGTCCTGGGCG
GTGCCAGCTTCAATCCCTCTACCACTGTCTCATTCTACGCCGCCGGCTTAAAACACAACTCATCTCTCATCTCCA
TGGCCGTGCAGTTTCCGGCTCAGGCCGCCGGCGGGCTGGTTGGCGCCAAAGCTATACTCCAAGTGCTGCCAAGCC
AATACAAACAGAGGCTTAGGGGACCTTCCCTGAAAGTTGACTTACTAACCGGAGTCATTGCCGAGGCGGCGTTGA
CTCTTGGGCTTAGCTTTGCCATCCTTTTGATCATGCTCAGGGGTCCTAAAAATCCACTTGTAAAGGTGTGGTTGG
TTGCTGTGGCGACTGTGGGATTGGTTGTTGCAGGGTCGGGTTACACGGGGCCTTCGATGAACCCGGCCAACGCAT
TTGGGTGGGCATTTGTGAATAATCAGCATAATACTTGGGAACATTTTTATGTTTACTGGATTGGTTCTTTCACTG
GAGCAACTGTGGCTGGTTGGATCTATCGGTTTCTCTTTCCC**TGA**
>*Bpe*SIP1;2
MGLMRVAIGDAILTSLWVFSAPMMGILTPIIATYLGVQAKSLAGLFITTLLATSLVLIFQLIGKVLGGASFNPST
TVSFYAAGLKHNSSLISMAVQFPAQAAGGLVGAKAILQVLPSQYKQRLRGPSLKVDLLTGVIAEAALTLGLSFAI

LLIMLRGPKNPLVKVWLVAVATVGLVVAGSGYTGPSMNPANAFGWAFVNNQHNTWEHFYVYWIGSFTGATVAGWI
YRFLFP*

***Bpe*SIP2;1**

>**FXXK01000213.1:**133500-142000 *Betula pendula* genome assembly, contig: Contig212, whole genome shotgun sequence
>Bpev01.c0212.g0010.m0001
Chr Bpe_Chr8 12,136,640-12,142,650 (-1) ::
complement(join(12136640..12136804,12136967..12137227,12142366..12142650))

>*Bpe*SIP2;1
TCTTATTCCACCTTAATGCAGTATTTAAAGAGAAAAAATTACACATAAAAGAAGTCACAGTCCCACACGAACAAG
ACTCTCCTTTGAAACAGTCTCCTATCCGAAACAACATAAAGAAGAGGTAAAGTAGACTCCTACTTGAATAAAACA
TAATCCCTAAACCAAAAGAAGATAAACATAAGAAAAGTCCTCCACTCACGTGAGAGACCCACCACTAATTAATAG
ACTCAATAACATAAAGCAATAACATCAATATCAACTCTATCCAGTACAGTAGCTTCCAATAGCTTCCAATCTTGC
ATCCATAACAAGTGTATTGTTGTTTCCCTTCCCTGTTCTATTTGCTTATATATAAAAAAAAACTTATAAATAAAA
AAAAGATAAAAAAAGCTAGTTTGTTGTCTCCGCAAATTATAAAAAGATTTCTAAAAGTTTTAGGTTTAGCATTAT
GTATAAAGAATAAGCAAATAGTAATGTTATATAATATATTACTATTTTACTATATTGACGTGACATTGTTAATTT
ATTATTGATTTTTATTTTTATTTTTTTTATTATAAAAACTTATTTAAGAGTGAATTAATAAGATCATGTAAGCAT
AATAAAATATCGGTGAAATATTAATGTGTTATATAGTATTAATCATATGCAAAAGCATGTAAACAATTATCACTA
AATAATATAATTTGGAGGATTCAAGTGAGTAGTCAAGTATTGAGATTTCATTTCTTGGGTGAGACCCACATTATC
GAATAATGAATATGC**ATG**ATAGGGAAGAAGTAATAAGAAAGAGAGTAACTTACCAAAAAAAGAAAAGAAAAAAGA
AAAAAGAAAAAGAAAAAAAAGAAAAGAAAAGAACAAAGAAAAGAGGGAGTAAAACCAACGACAGCTTGTAAAAGC
AAAATTTGTCATTTTATTTTGCCCACAACGGACCCGCTAAAAGGCGAAAAACCGAGTCACAACGGACCCATCATC
ATACGACGGTACGGCACACCCGAAACACATGTCAGACATCGGACAACGATCACAACACGTCAAAGCTGAAAAACA
GAGGAAATTTCCTATGGTCTCACACTTTTCTGGAGAATCTTTCGAAAATAATTGTATCTCCATCGTAATCTATAA
AAAAAACTGTGTCTCCATGCGCAGAGAGAGAAAAGAGCGTGTCCAAGCCACCCAAATAGTAGGAGAAGAAGAAGA
CTCTTTCAGATTTCTCGTCTGAAAATGGCTTCGGCTGGTTTGCTGGTCTCAGATTTCATAATCTCTTTCATGTGG
GTATGGTCAGGGGTTCTGATTAAGATCTTTGTGTACAAAGTTTTGGAGTTGGGTCATGAACCCAGTGGCGAGGTT
ATCAAGTGCGCTCTCTCCATCGTCAACATGTTCTTTTTCGCGTTTTTGGGTAAGACTACCAAAGGTGGAGCCTAC
AATCCTCTCACCGTGTTCGCTGGTGCCATTTCTGGGGATTTCAAGCGCTTCCTCTTTGACGCTGGTGCTAGAATC
CCAGCTCAGGTTACCTCTCTCTTTGGTGCTTTCATTTGTTTGTTGGAACCTATGTTTTTGTTGGTAAACGGCTTA
ATTTGATTGAAATGTGAATTTACTTTGTTTTATTCGGGTGTTGAAGCACTGTCTGATTATCGAGTGGATTTGTTT
GATTGAAGCGCTAACTGGGTTTGCTTTATTTGCTTGTCTGGTTCGCGGTAAAGTGAAGGAAACAGGATTTTTTTG
ATTTATGTCATTTTATTTTGCCCAATACTGGAGTACTTGCCTTCAATCATGACAAATAATTGTATAAAGCTTACT
TGGGTGGATATCTAAAGTTTTTGGGCTCCAAAATAAAATTTTAATTGAAACTCCAGCTTGTTTTCTTTTTCAGAA
ATGAATTGGAGCATGATCTTGGTGTGTTTTTTCTTTTTAAAATTTATCTTTTGTTTGGAAGCTGAAACTTACCGA
AAAGAAGGAATGCGGAAATCTTAGTTCAGCTTTTTTATGGGGGCTTTCTGCTTAATTTGTTCTTTTGATTTATAC
TTGAAGTGCAAGATGTGAATTGGGTTTTGTAGTTTTGTTATAGTGAATTGTCTTGGTTGACGACTTCTCTTGGCT
TTTTTATTTATTTTTTTTGTTGGACTAGATTGATTGATTAATGTTCTATAGTTAGTTTGAAGTGTTGGTTCTCTA
ATTAAATTGCATCTGGCTGGCAATTGAATAAGCTTGTTGAGTAGTAAAGGATACAACAATTTGATGTTGTTAACT
CTCTTAAACGATGCAGAGAGATTAGAATGTCTTTATAGAATATTTGCTGTACAGAGGTTCGTTACAAATATAACT
CTACAACAACTGATCACGCGTGATGTATTTTATCACGCTTATCTTCAACTATACACTTTGAGTTTGAAATCTTGC
TATCATCATTTGAGTTTGAAATCATGATCTCATCTTTCCTGATCTGATTGTGAGAGGATGATGTTGTTATAGGCC
CCCTCAATTGTAGCGGGACAGGAGGCACACTAAGATTGTCACAAAGTAATGAAAACCATTGAGATACAAGGGACT
TGGTGAATATGTCAGCTTTTTGATCTTTACTTGAAATAAATTGAAACAGTGAATTTTTGGAAGCCACCTTATCTT
GAATAAAATGGAAATCGATCTCAATATGTTTTGTGCGTGCATGAAACATGGGATTGGCTGTGAGATAAGTAGCAC
CGATAACATAGGTGGAGTGGTAAGAAAAATGCCCAATTCCTTAAGTAGAGACTGAAGCCATTGAAGCTCAGCTGT
GGTGTTAGCCAAGGCTTGATATTTGGCCTTGGTACTAGACCGTGAAACAATTGCCTATTTGCGGGAACTCCAAGA
GATAAGGTTGCTGCCAAGATAGATGCATAACATCTAGTGGACCAACAATCATCGGGGCAACCTGCGCAGCCCGCA
TCAGAATACGAATGAAGTTGAGTAGATGATTGACGCTGAAGAAGTAAACCATGGAACATAGTATGCCGTAGATAG
AATTCTCTTCATCGCTGTCCAATGATTTATAGTTGGGTGTTGAAGAAACTAGCACACCTTGTTTACGGCAAAAGA
AACATCGGGGACGTTTGGGAATGGCTTGGGCTTGCACTGTTCACGTTTATTATTTTTTCAAAAAAACATTCCCAC
TTTTTTATATCACATCAACTACTTTTAACAACTTTTTTCACTTTTTTATATCATATCAAATATTTTTATTACATT
CATAATATCACTAAAAAACAAAAATTTTCACTTTCCAATACAAAAAATTTACAACATTCCCACTACTTTATATCA
CATCAAAAGTCAACAATCCCCAAACCACAATCCCAACCCTTTGCCAAACACCCCCATCAGGTCTTGTAAGTTAAA
GGCACTGCATGGATCCCACTATGCTATGGTAGAGAGAAGGATCTGAAAATGCTTCACCATCAAATTTTGAGAGTG
AGGTAGTGGAGGACATAGACGTAATAGGTTTAGATATTGCCATATTTGCACGTTGTAGCAAATCCAAAATATACT
TCTTTTGTGAAAGAGATAGCCCTTGCGAATCATGGTGGACTTCAACACCTAAAAAGTAATGGAGAGTGGCCCCAT
GTCCTTTATCGGAAAAACACCTTGCAGCTTGTAAATAAGAGAATTAAGAGCAGATGAGTTTGGGCCAGTTACAAT
TATATCATCCACGTATATGAGGATGAAAATTATAGTAGCACTGTGGCCATATATGAAGAGAGAAATATCAGTGCG

GGAACCCACAAATCCAAATTCCAATAGCTTTGAAGGTGAGAAAACCAAGCTTGTGGGGCTTGTTTGAGGCTATAG
AGGGGATTTCTTAAGGTGGCACACGTGATTTGGAAATGATGGATGAACATAGACCGAAGATTGAACCATGAAGAC
ATTCTTCTGCAAAAAGCCATGTAGAAAAGCATTTGTAACATCTAACTGTTTTATTCACTAGTTGGAGGCAACAGC
CAGTGAGAGAATAGTTCTAATGGTTGTGGGTTTCACAATGGGACTAAAAGTCTTCCCAAAATCAATACCGGGCTG
CTAATGAAACCCTTTGGCCACAAGACGGGCCTTGTAGCGATCAATACTGCCGGTTTCCTTTTGATATAGAAAACC
CATTTGCATCCCACTAGGTTCATGTTAGGACGTTGAGGAACTAATGTCCAAGTCCCATTCTGCAAAGAGCATTAA
ATTCAGTGTTCATGGCCTCTCTCCACTTCAGACTTTTTGATACAGCAGTGAAAAAAGTAGACTCCACATCAAGAG
ATTTCGAGCCCGAGGTGAAGGAAATGGTAATGTGCCATCCGTAAAACGATGTGGCTTGCAAATGTTGTTGCGCAA
CCATGTCTGCATATGATGGCAACTATTAGTACTGAAACTTGGGCCTGAGTGTTTATTATGAGACTCCACATCAAG
AGATTTCGAGCCCGAGGTGAAGGAAATGGTAATGTGCCATCCGTAAAACGATGTGGCTTGCAAATGTTGTTGCGC
AACCATGTCTGCATATGATGGCAACTATTAGTACTGAAACTTGGGCCTGAGTGTTTATTATGAAGGCAACCATGT
CTGCATATGATGGCAACTATTAGCATTATAGGAATGATTATTATGAAGCTTGGGCCTGAGTGTTTTGGTATAAGT
TACTGAAACTATTAGTACATAGTGAGATGGGATTTCAAATAGTACAAAGAATATATCATGTGATTAAATTAAGAA
TTCAAGGATAAGAGGTAGTGAATTAGAGTGACCAATTGCCCAATAAGCCAAGGACGGGCCGATTATCCATGTAGG
TGCACTGTTCTTGTGTTTTACTTTTGTTTATGCATGTTCTGGCTTAGTTCTAGATTATTTCCCGCTATGCAATAA
TTTTATGCACATGATCCCAACAGATCAGCGGTGACATGATTTGTAGCCCTTGTGTCAGGATACCAATTAAGATTA
CATGGGATTGACGTGCAGTTGTAAAGGCCTACATCGATGGAGCAGAGCCTTGCTGAAATGAGTAATCAAATATGT
GGTAACACCGTAGAGCTGTATGGCCAACTTTGTTACACTTGGCAAACAGGTCTGTTGTTGTTGTTGTTACCAGAG
AAAGCAGGATAACCCCCACGTCCAAAACCTTTGCCCTGGTTTCCATATGCACGATTGTTGAATGAAGGCCGTTGA
GAAGAGCTATTGGAACCAGTTTTCGTTGGACGAGTGTTGTTGTTGTAATGAGGCTTCTAAGAGGGTTTAGTAGCA
ACATTAGCATATGGGAACGTAGCATTTGCAGAGGCAGTACTGTGTTCAATCCACATTTCATGAGTTAATAAGTGA
GCAGAAAGCTCTTCAAGGGACATTTGGGTCCACACGAGTAGTCACAGAAGTGACAAAAGGATCAAACTGAGATCC
TAGTCCAGCAAGCAGGAATGACAAATGCTCAAAATCATGGAGCGGTTGTCCTGCAGCAGCCAATGTGTTGGCAAC
TTGAATTTCTGAAAATACTCAGAAATTGGGAGTGACCTTTCTAAATGGTGGCAAGTTGAAAATGAACTTGCATGA
TGCGTGCCATTGAATGAGAGGAAAACATCCTTTCAAGAGCAGCCATACTTCGGCAGATGTGGCATAAACCCACAA
CCTATGGGATTAAACTCTCTGAGAGAGACGATATTATAGCTCCAAGGATCAACTGATCCTGTTGAATCCAGAGTA
AATACTCAGGATTTGCAATAAGGGCAGGGGCAGCAGAGCTGGAAGCAGCCAACGGGTTGGGGATGGTTTGTGGTG
GTGGTGGGTGAGATCCATCAATGAACCTGTAGACATTTTGTCCAAAAGGTAAGGTAGGATGACAGCTTTCCAGGA
AAGATAGCTGTCCTGGGTTAATTTCACCGTGGCATGTTGGGAATTGTCGATGAAGGCAGGAGGAGATGATTTCAT
AATGGAGGTCGCAGGGGAAGAAGTGGTGGTAGACGACGTGGAATTGGCCATTTTGAAAAAAATATTAGACCTAAG
TCTTGAGTAGTTTCAATACCATAAAGGATAAAACAATTTGATGGTGTTAACTCTCTTAAATGATTCAAAGAGATT
ATAATGTATTAATAGAATATTTGCTGTACAGAGGTTTGTTACAAATATAATCTACAACAACTGATCATGTGTGAT
TTATCTTATCACGTTTATCTTCAACTATACACTGGGTTTGGAATCTTGCTATCATCATTTGAGTTAGAAATCTTG
ATATGTCTGTGATGAGTTTTAATATAATAAAAAATGTACTAAAAAAGTTGTCACATGGAGTAAAAGGAAAATGTC
TCTTTTTAATCATCTTTTCAACATGTCTGATCAATTTCTCATGCTATTGTTCTTACTGCTCATGCATCATGAAGA
AATGGGATCAGTGTCATTACGTACTTTTGATGAACAAAGCTACTGCAGGGCTGAGAGAGAAAATACTGAGTTGGA
CAAAGAATGATTATTGCTCATGTCCTGCAATTGTGAATTTCAAAGACAATATTGTTTGGAGTAGATAATTTAAGT
TTGCCAAAACTGTGTGCCATGAAAATGACTTGCAATCATTCAGTAGTAGCTGGTTCCTTATACCATGGAGGCAAC
TTGTTTTTCTTATAGTCATTGTTTTTGACCATATATTTTCTCTCCAGGTGATTGGATCTATTATTGGGGTTAAGC
TCATTATTGAGACTTTCCCTGAAATAGGACTCGGGCCACGTTTAAATGTTGACATCCATCGAGGTGCACTGACAG
AAGGTTTTCTGACATTTGCAATTGTTATAATCTCTCTTGGTCTGGCCAGAAAAATTCCTGGAAGTTTTGTCATGA
AGACTTGGATCTCAAGCGTCTCCAAGTTAACTCTTCATATACTTGGCTCTGATCTGACTGGTGGTTGCATGAACC
CAGCTTCTGTAAGTATCTACTTTTACTGATATATATATATATATATATATATGCACATATTTTTTTTTCCTATCATT
ACTTATATATATATATATTTATATGCACATATTTTTTTTTTTCCTATCATTACTTTAGCTGCAGAAACTTAAATT
ATAGAAATTGATTTATGCAGGTGATGGGATGGGCATATGCTCGCGGGGATCACATAACCAAGGAGCATATACTTG
TATACTGGCTTGCCCCAATAGAGGCGACTCTGCTGGCGGTATGGATATTTAGGTTGGTAGTCCGGCCAATAAAAG
AGGAGAAAGCAAACGTGAAGAGTAAATCAGAATGATGAAGTTCCACTTGGAATTCAAGAGTGGATTTATCCAATG
AATCTGTTCGCCTTTGAGGAGGTGAAAATGGTTAAAAGATTATTAGAGTTGAAGGAATATACAGTACTTAATTTG
TACATCAATTCCTTTGGCTCGCCCATTTGTAATATATCATTCATTGCATTTAGTAGATTCACCGGTATGTTCGTC
CCAAAACTCTCTCTACCCCGTCTGTGTTCATGCAAAAAGATAAATGTGTGTACCACTTGCACACATGCTTTTGCT
TACAAGTGAAAGAAATTAGTATCTTGCACTTAACGTGCTAGCATAATATGAAGTTTTTGAATTAAATACCATGAA
CTGCATATTGGGAGGTAACCCTTTTTTTAGAAGGATTTAGCGACGGCAAAGTTAAAATATTGTAGTATGTAACTA
ACACTAAGAACTGTCCCAAAAATCCAGAAAATGAGAGCTTGAGAATCTGT**TAG**GTGATGCACCTATTTTTGGAAG
CCAATGTTTACAACGATACGAGGTCTCCCTAGGAAGCAGGATCACAAAGAATGTTCGTTCTTATAGCATCTTTAG
TATTTAGATGTGGGCATGGCATATATCATGTAGTAGAACAGTGAACTTTTTCGAGTTCTCTCCTTACATAATTAG
AATGCGATAAAAGTATGGTGTATAGCATGCTAACAATGATAGTAAAGGCTCGTAGTGAAAACCATGTAATCGCTC
CATAATTCTCCTTTTTTTTTTGTGTAAATATTGATATAGGTGGTGGAATAAGGGAAATTTCTACCTGGTATCACT
GGGAGTGCCCTCTGGATTAACGTAATTTAGTTACAATTTTTATAGCAATGAAACTAAAATGAGTAGTAAACAGTG
TTGAGGGCTGATTTTTCGTGGGACTCAAGAAGAAGATTGACATGTGCTACAAAAATGAAAGAGGGCCCAATACGA
AAGGAGCTTCCCCAAGCGAGTGTCAACGGGCTAGCAAAAGAGGGGCTTACGTAGGCAACCGTCTAACCATTAAAA
GATTGTTGTTGCATGATCACAAGGCCGTTGGACGAATGTAATCCCCACATAGTAGCATTCATGAAAGCTTAAAGG
ATTGATGCTGCACGGTCAAAAGGCCGCTGAACGGCCATGATTCCGCCATATGACACAAGCAGTGGCTTACGGCCT
GAACAATTTGCAATTGTTGAGAGCCCGTGTAATACCTCCAAAAATAAAATAATTTTTAAAATAAATCTTTT

>*Bpe*SIP2;1
ATGATAGGGAAGAAGTAATAAGAAAGAGAATTTCTCGTCTGAAAATGGCTTCGGCTGGTTTGCTGGTCTCAGATT
TCATAATCTCTTTCATGTGGGTATGGTCAGGGGTTCTGATTAAGATCTTTGTGTACAAAGTTTTGGAGTTGGGTC
ATGAACCCAGTGGCGAGGTTATCAAGTGCGCTCTCTCCATCGTCAACATGTTCTTTTTCGCGTTTTTGGGTAAGA
CTACCAAAGGTGGAGCCTACAATCCTCTCACCGTGTTCGCTGGTGCCATTTCTGGGGATTTCAAGCGCTTCCTCT
TTGACGCTGGTGCTAGAATCCCAGCTCAGGTGATTGGATCTATTATTGGGGTTAAGCTCATTATTGAGACTTTCC
CTGAAATAGGACTCGGGCCACGTTTAAATGTTGACATCCATCGAGGTGCACTGACAGAAGGTTTTCTGACATTTG
CAATTGTTATAATCTCTCTTGGTCTGGCCAGAAAAATTCCTGGAAGTTTTGTCATGAAGACTTGGATCTCAAGCG
TCTCCAAGTTAACTCTTCATATACTTGGCTCTGATCTGACTGGTGGTTGCATGAACCCAGCTTCTGTGATGGGAT
GGGCATATGCTCGCGGGGATCACATAACCAAGGAGCATATACTTGTATACTGGCTTGCCCCAATAGAGGCGACTC
TGCTGGCGGTATGGATATTTAGGTTGGTAGTCCGGCCAATAAAAGAGGAGAAAGCAAACGTGAAGAAAAATGAGA
GCTTGAGAATCTGTTAG
>*Bpe*SIP2;1
MASAGLLVSDFIISFMWVWSGVLIKIFVYKVLELGHEPSGEVIKCALSIVNMFFFAFLGKTTKGGAYNPLTVFAG
AISGDFKRFLFDAGARIPAQVIGSIIGVKLIIETFPEIGLGPRLNVDIHRGALTEGFLTFAIVIISLGLARKIPG
SFVMKTWISSVSKLTLHILGSDLTGGCMNPASVMGWAYARGDHITKEHILVYWLAPIEATLLAVWIFRLVVRPIK
EEKANVKKNESLRIC*
