# Supplementary material for: Genome-Wide Identification, Structure Characterization, and Expression Pattern Profiling of the Aquaporin Gene Family in Betula pendula
Source: Int J Mol Sci. 2021 Jul 6;22(14):7269. doi: 10.3390/ijms22147269 (PMC8304918; doi:10.3390/ijms22147269)
Supplement: Supplementary file 1 [file ijms-22-07269-s001.zip › Supplementary Files Betula MIPs/FigureS10.pdf]

Figure S10

Analysis of raw data distribution for gene expression

Data with no mapping values (i.e. no AQP expression level) are highly suspected, for statistical reasons, of being actually not available instead of zero. Classically, mapping values are regularized-log transformed to be normalized using the 'bestNormalize' R package (Peterson & Cavanaugh, 2019, J. Appl. Stat. doi:10.1080/02664763.2019.1630372).

In the gene expression data we produced in *Betula pendula* aquaporines (AQP), the representation of quantile-quantile plot against theoretical Normal distribution reveals clearly that the absence of expression should not be confounded with a zero expression level. In fact, the absence of expression represents much more a lack of information on the gene expression than a definitive result of zero expression.

In the Figure S1 below, it appears clearly that the distribution of raw gene expression data obtained for AQP are flawed to an abnormal distribution (non-Gaussian, Figure S1A, Shapiro-Wilk normality test pvalue=1.94e-22) when values of genes lacking expression information are replaced with a zero. On the contrary, when these values are considered as non-available (no expression information available), then the distribution of AQP expression values is Gaussian (Figure S1B, Shapiro-Wilk normality test pvalue=1).

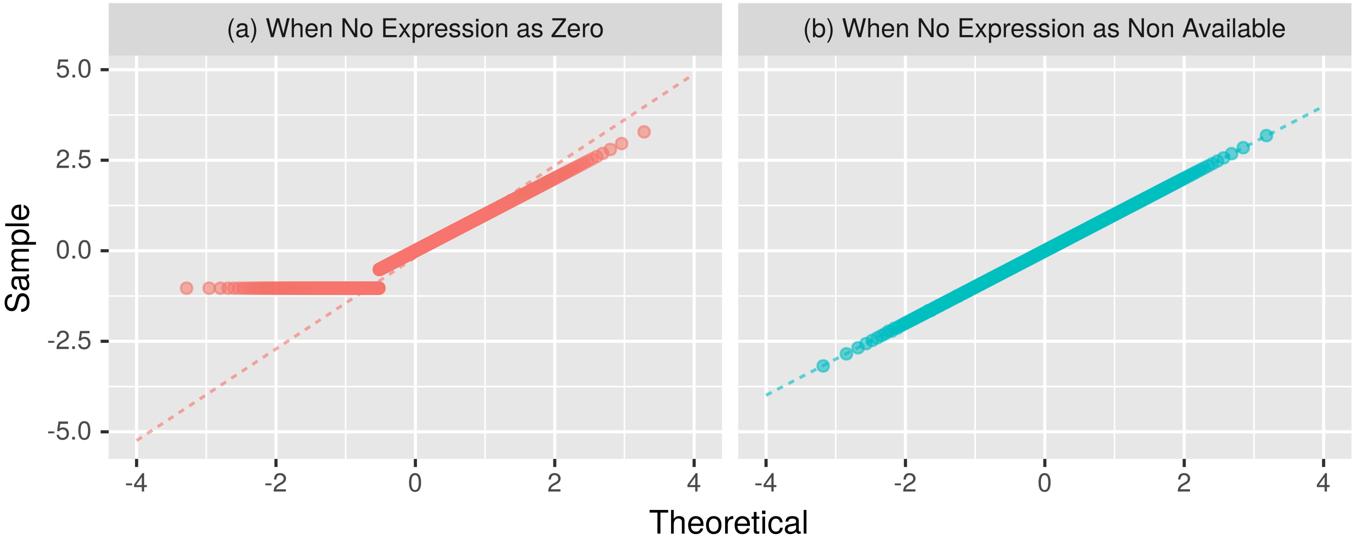

Figure S10. Comparison of raw data distribution of regularized-log transformed AQP gene expression in *Betula pendula*. (**A**) Quantile-quantile plot of regularized-log transformed values including non-expression values as zero expression. (**B**) Quantile-quantile plot of regularized-log transformed values considering non expression values as non-available information. Dashed lines show theoretical Gaussian distribution.
