# Supplementary material for: Genome-Wide Identification, Structure Characterization, and Expression Pattern Profiling of the Aquaporin Gene Family in Betula pendula
Source: Int J Mol Sci. 2021 Jul 6;22(14):7269. doi: 10.3390/ijms22147269 (PMC8304918; doi:10.3390/ijms22147269)
Supplement: Supplementary file 1 [file ijms-22-07269-s001.zip › Supplementary Files Betula MIPs/FigureS11.pdf]

Figure S11

(a) **Distribution and homocedasticity**

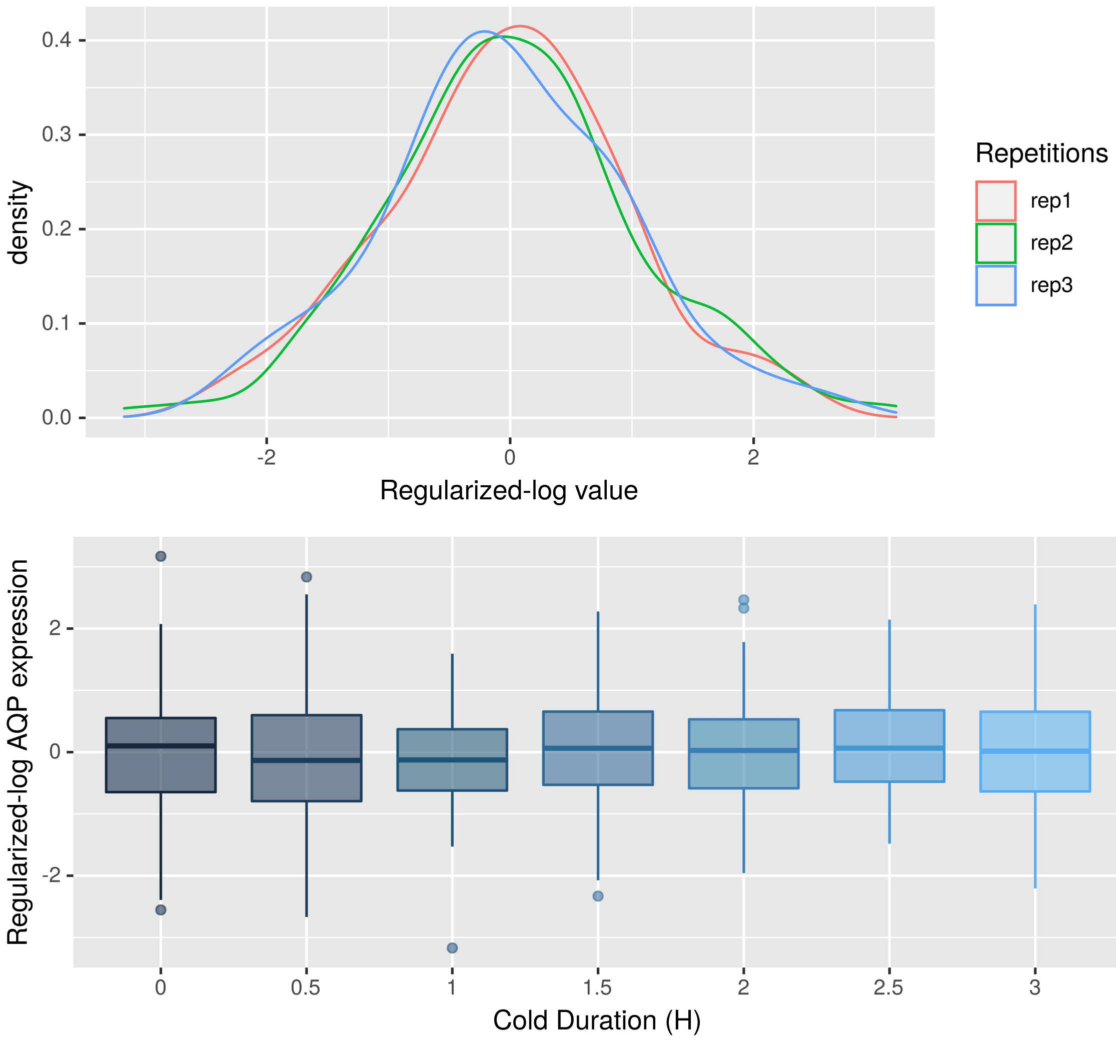

(b) **Analysis of variance**

term	df	sumsq	meansq	statistic	p.value	contribVariance
Hour	1	1.219	1.219	1.169	0.2802	50.4 %
rep	2	0.2273	0.1136	0.109	0.8968	4.7 %
Hour:rep	2	0.08567	0.04284	0.04108	0.9598	1.8 %
Residuals	388	404.6	1.043			43.1 %

Figure S11. Exploratory data analysis of Cold Stress experiment. (**A**) Normality of regularized-log AQP expression data and homoscedasticity. (**B**) Analysis of variance. The effect of Hour on the expression value is very weak (no significance), although it contributes half to the global variance. This is 43% of the variance which are not explained by the experimental design.
