# Supplementary material for: Genome-Wide Identification, Structure Characterization, and Expression Pattern Profiling of the Aquaporin Gene Family in Betula pendula
Source: Int J Mol Sci. 2021 Jul 6;22(14):7269. doi: 10.3390/ijms22147269 (PMC8304918; doi:10.3390/ijms22147269)
Supplement: Supplementary file 1 [file ijms-22-07269-s001.zip › Supplementary Files Betula MIPs/FigureS12.pdf]

Figure S12

(a) Distribution and homocedasticity

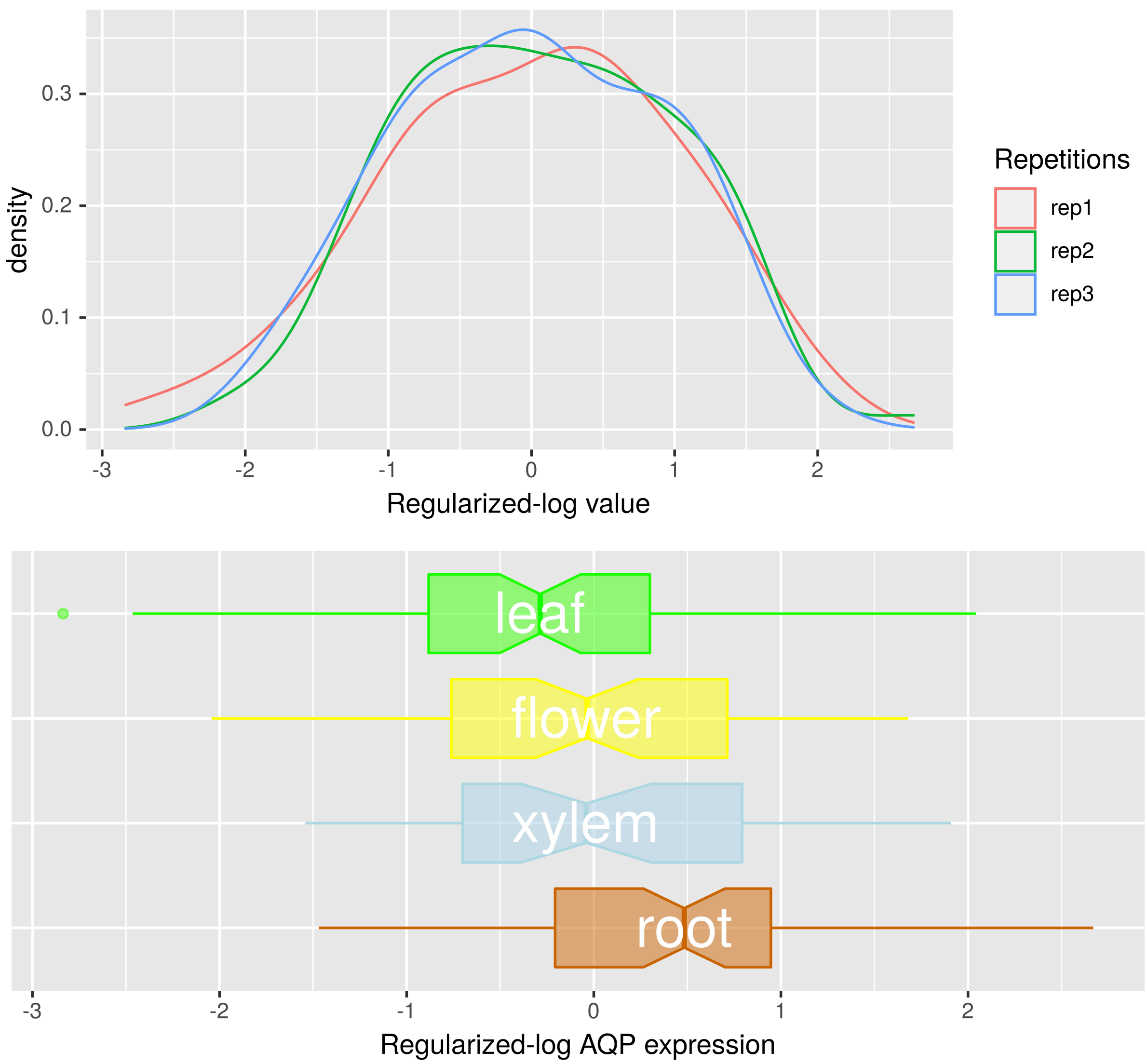

(b) Analysis of variance

term	df	sumsq	meansq	statistic	p.value	contribVariance
Tissue	3	17.22	5.738	6.242	0.0004195	83.7 %
rep	2	0.2507	0.1253	0.1364	0.8726	1.8 %
Tissue:rep	5	0.3554	0.07107	0.07732	0.9956	1 %
Residuals	252	231.7	0.9193			13.4 %

Figure S12. Exploratory data analysis of organ experiment. (**A**) Normality of regularized-log AQP expression data and homoscedasticity. (**B**) Analysis of variance. The effect of the organ is clear and significant on the general expression profile of AQP.
