# Supplementary material for: Genome-Wide Identification, Structure Characterization, and Expression Pattern Profiling of the Aquaporin Gene Family in Betula pendula
Source: Int J Mol Sci. 2021 Jul 6;22(14):7269. doi: 10.3390/ijms22147269 (PMC8304918; doi:10.3390/ijms22147269)
Supplement: Supplementary file 1 [file ijms-22-07269-s001.zip › Supplementary Files Betula MIPs/FigureS3.pdf]

Figure S3

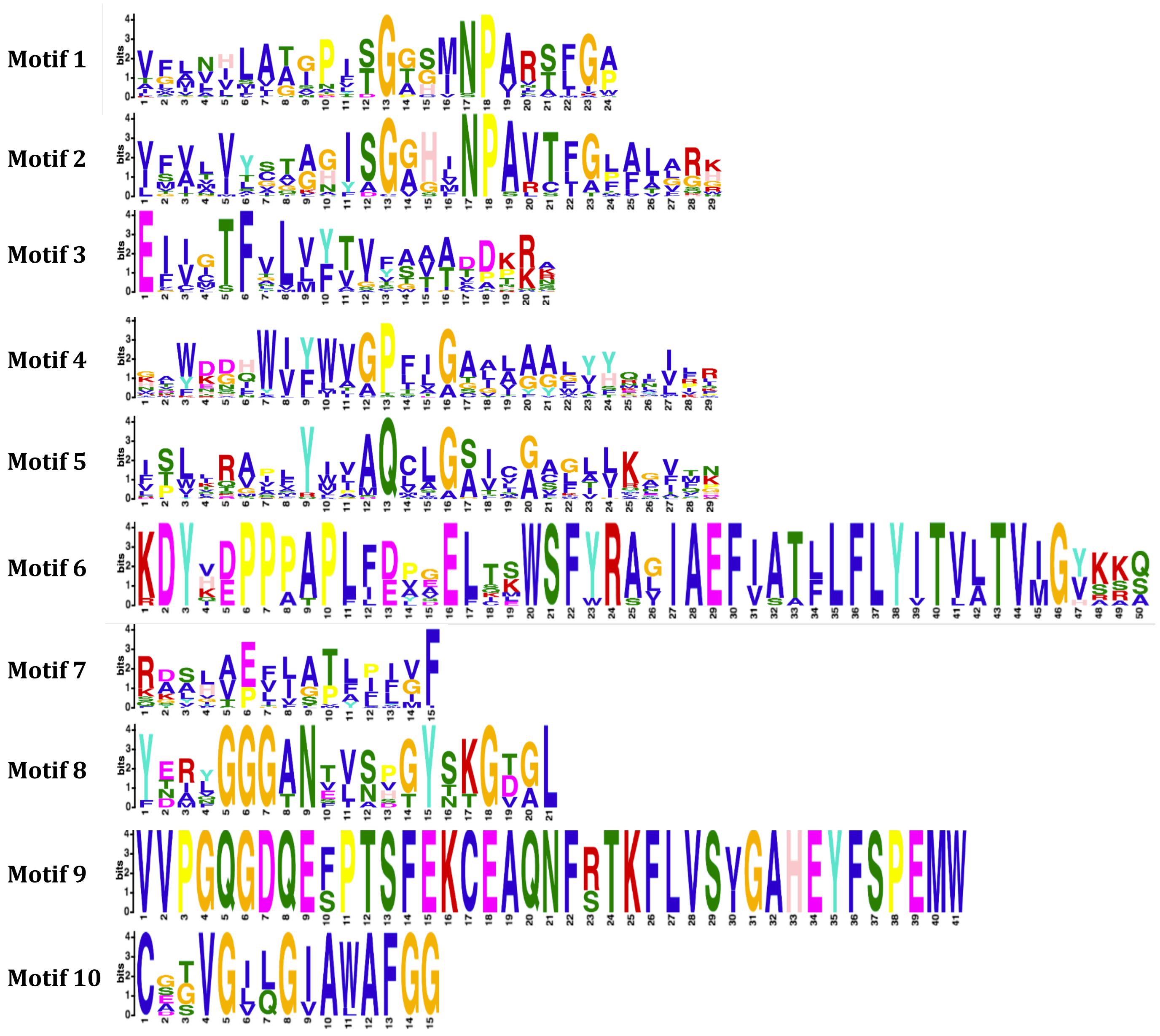

Figure S3. Amino acids constituting the ten motifs of the 33 full-length *Bpe*AQP protein sequences (**Figure 2C**). Motif analysis was performed with the MEME web server. The statistics of MEME motifs detected in the AQP multiple alignments are detailed in the next pages of this document.

Motif 1

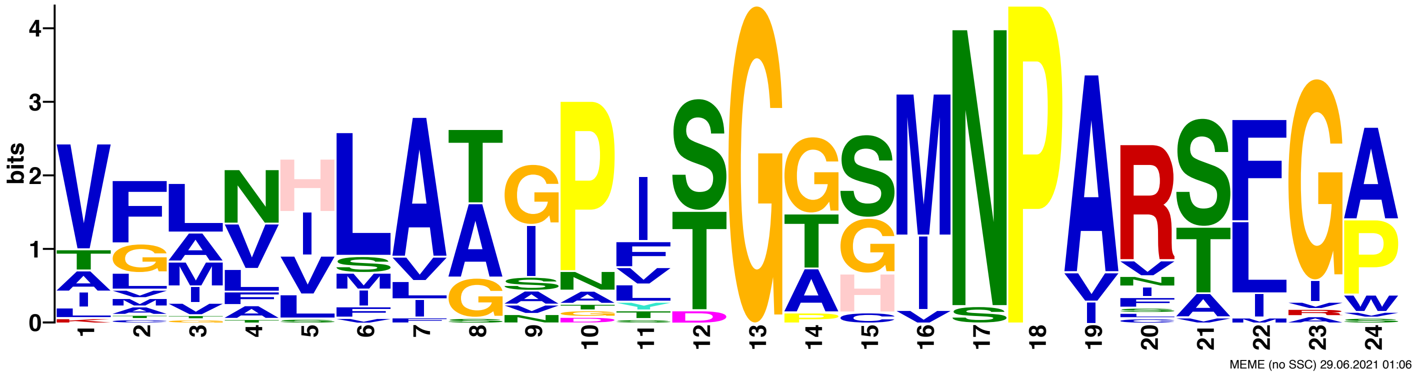

MOTIF 1

VFLNHLAAGPISGGSMNPARSFGA MEME-1,
width = 24 sites = 34 llr = 1338 E-value = 9.2 10^{-296}.

Simplified pos.-specific probability matrix :

```
     A  1121::7411:::2::::8:1::5
     C  ::::::::::::::1:::::::::
     D  :::::::::::1::::::::::::
     E  ::::::::::::::::::::::::
     F  :4:1:1::::2::::::::1:5::
     G  :2:::::24:::a43:::::::8:
     H  ::::3:::::::::2:::::::::
     I  1:1:311:3:4::::3::11:11:
     K  ::::::::::::::::::::::::
     L  1141161:::1:::::::::::4::
     M  :12::1:::::::::6::::::::
     N  :::4::::11::::::9::1::::
     P  :::::::::8:::1:::a:::::4
     Q  ::::::::::::::::::::::::
     R  ::::::::::::::::::::6:::
     S  :::::1::1::5::4:1:::5:::
     T  1::::::4::14:3::::::3:::
     V  61132:1:1:1::::1::11::::
     W  :::::::::::::::::::::::1
     Y  ::::::::::1:::::::::::::
```

Detailed statistics of MEME motifs detected in the AQP multiple alignments.
Simplified po(sition)-specific probability matrix represents in horizontal coordinate the position of each amino-acid in the motif and in vertical coordinate the relative occurrence of amino-acid variation in the detected motif. For instance, in the motif 1, the first amino-acid (left-most column in the matrix) is represented majoritarily by V (6), and minoritarily by A(1), I(1), L(1) and T(1). Capital letters are IUPAC's amino-acid one-letter code. **a** = all sequences show the same amino-acid. **llr** = log-likelihood ratio. Colons are meant for visual alignments of each column in the matrix when using a monospaced font. This statistical approach applies to all of the following 9 motifs.

Motif 2

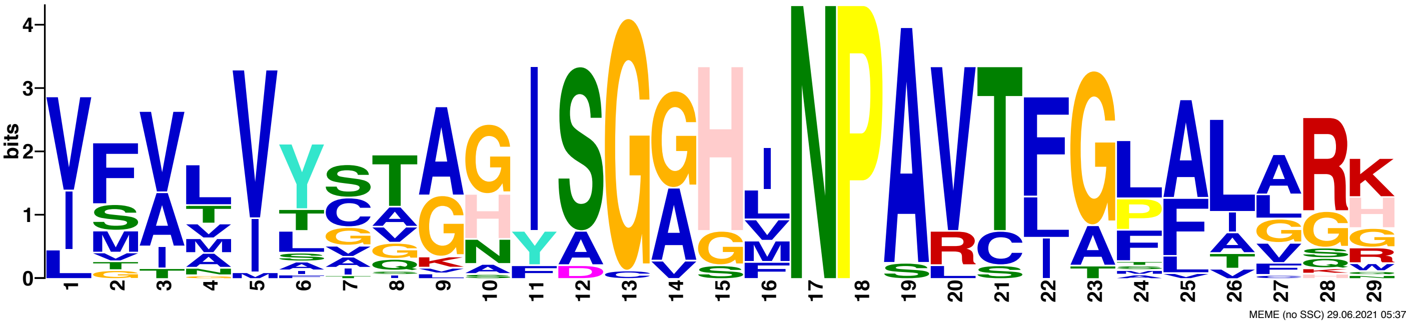

MOTIF 2

```
VFVLVYSTAGISGGHINPAVTFGLALARK MEME-2,
width =  29  sites =  31  llr = 1510  E-value = 2.8 10^-344.

Simplified pos.-specific probability matrix :
     A  ::31:11251:2:4::::9:::2:513::
     C  ::::::3:::::::::::::2::::::::
     D  :::::::::::1:::::::::::::::::
     E  :::::::::::::::::::::::::::::
     F  :5::::::::1::::1:::::5:23:1::
     G  :1::::2145::a52:::::::7:::222
     H  :::::::::3::::8:::::::::::::3
     I  3:1:3:1:::8::::3:::::2:::1:::
     K  ::::::::1:::::::::::::::::::3
     L  2::4:2:::::::::2:::1:2:4162::
     M  :2:1:::::::::::2:::::::::::::
     N  :::1:::::2::::::a::::::::::::
     P  :::::::::::::::::a:::::2:::::
     Q  :::::::1:::::::::::::::::::1:
     R  :::::::::::::::::::2:::::::61
     S  :2:::13::::8::1:::1:1::::::1:
     T  :112:2:4::::::::::::8:1::1:::
     V  51517:11:::::1:2:::8:::::12::
     W  ::::::::::::::::::::::::::::1
     Y  :::::5::::2::::::::::::::::::
```

Motif 3

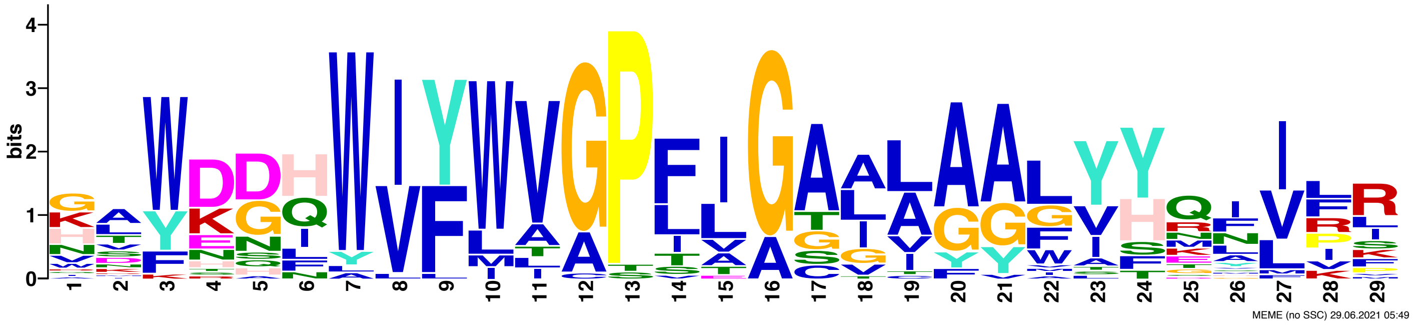

MOTIF 3

```
EIIGTFVLVYTVFAAADDKRA MEME-4,
width =  21  sites =  31  llr = 1150  E-value = 2.6 10^-255.

Simplified pos.-specific probability matrix :
     A   ::::::1:::1::44511::2
     C   ::12::::::::1::::::::
     D   ::::::::::::::::46:::
     E   a::::::::::::::::::::
     F   :2:::a:::5::4:::1::::
     G   :::4::1::::1:1:::::::
     H   ::::::::::::::::::1::
     I   :752:::::::1::11:::::
     K   ::::::::::::::::::442
     L   :::1::193:::::::1::::
     M   :::1::::2::::::::::::
     N   ::::::1:::::::::::1:2
     P   :::::::::::::::::22::
     Q   ::::::::::::::::::::1
     R   :::::::::::::::::::62
     S   ::::1::::::113::::::1
     T   ::::9:1:::6:11242:2::
     V   ::41::515:371:3:1::::
     W   :::::::::::::1:::::::
     Y   :::::::::5::2::::::::
```

Motif 4

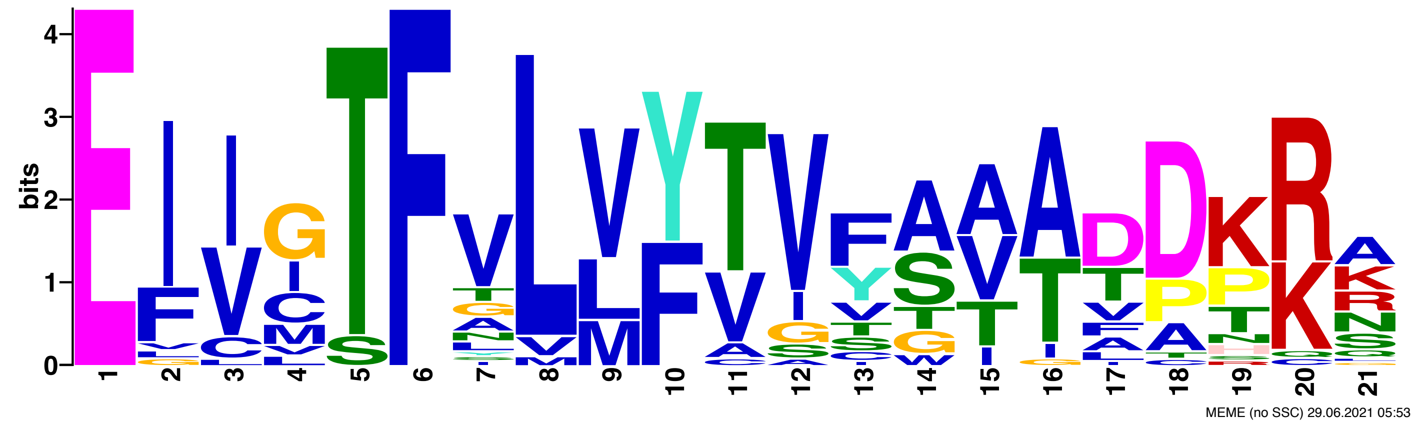

MOTIF 4

```
GAWDDHWIYWVGPFIGAALAALYYQIILR MEME-3,
width =  29  sites =  32  llr = 1445  E-value = 9.4 10^-312.

Simplified pos.-specific probability matrix :
     A  :2::::::::12::1263366:1::1:::
     C  ::::::::::::::::1::::::::::::
     D  :1:44::::::::::::::::::::::::
     E  :::1::::::::::::::::::::1::::
     F  ::1::1::4::::5:::::1:2:1:2:21
     G  2:::3::::::8:::811:332::1::::
     H  2::113:::::::::::::::::3:::::
     I  :::::2:5:11::15::21:::2::2411
     K  21:2::::::::::::::::::::1::11
     L  :2:::1:::11::23::34::4:::1221
     M  :::::::::1::::::::::::::1::::
     N  11:111::::::::::::::::::12:::
     P  ::::::::::::9::::::::::::::21
     Q  ::::13::::::::::::::::::3::::
     R  ::::::::::::::::::::::::1::23
     S  :1::1::::::::1::1::::::1::::1
     T  :1::::::::1::11:1::::::11::::
     V  11:::::4::7:::1::11:::2:::31:
     W  1:6:::9::8:::::::::::1:::::::
     Y  ::2:::1:5::::::::::12:55:1:::
```

Motif 5

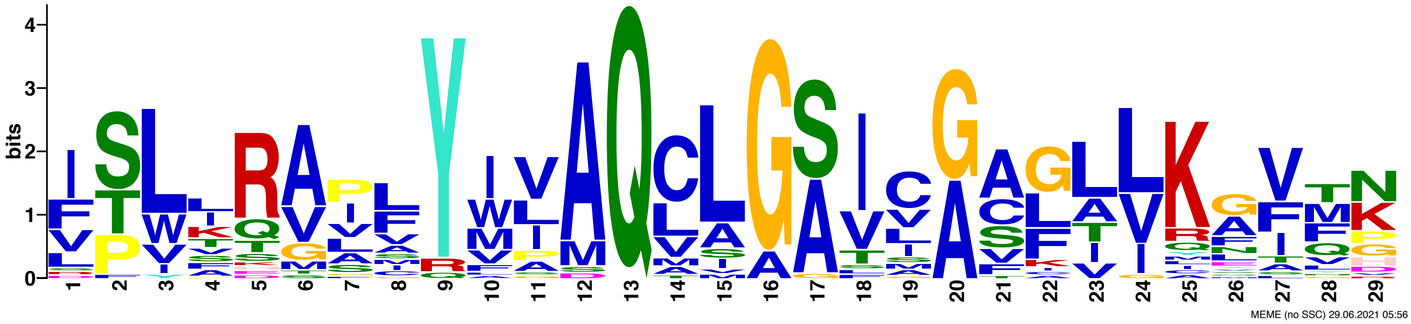


```
MOTIF 5

ISLLRAPLYIVAQCLGSICGAGLLKGVTN MEME-5,
width =  29  sites =  34  llr = 1433  E-value = 8.6 10-297.

Simplified pos.-specific probability matrix :
     A  :::1:511::18:1115:154:2::21::
     C  :::::::1:::::5::::4:2::::::::
     D  ::::::::::::::::::::::::::::1
     E  :::::::::::::::::::::::::1:::
     F  2::1:::2:1:::::::::::12:::122:
     G  :::::1:::::::::9:::5:4:::3::1
     H  ::::::::::::::::::::::::::::1
     I  4:12::21:42:::1::61:::12::2::
     K  :::1:::::::::::::::::1::7:::3
     L  1:62::13::2::27:::1::345:1:1:
     M  :::::1:1:2:1:1::::1::::::::2:
     N  :::::::::::::::::::::::::1::3
     P  :2::::2:::1::::::::::::::::::1
     Q  ::::1::::::::a::::::::::::1::1:
     R  ::::6:::1::::::::::::::::1::::
     S  :5:11:11:::::::1:5:1:2:::::::
     T  :3:11::::::::::::1::::1:::12:
     V  2:11:211:14::1:::22:1:13::41:
     W  ::2::::::2::::::::::::::::::::
     Y  ::::::::9::::::::::::::::::::
```

Motif 6

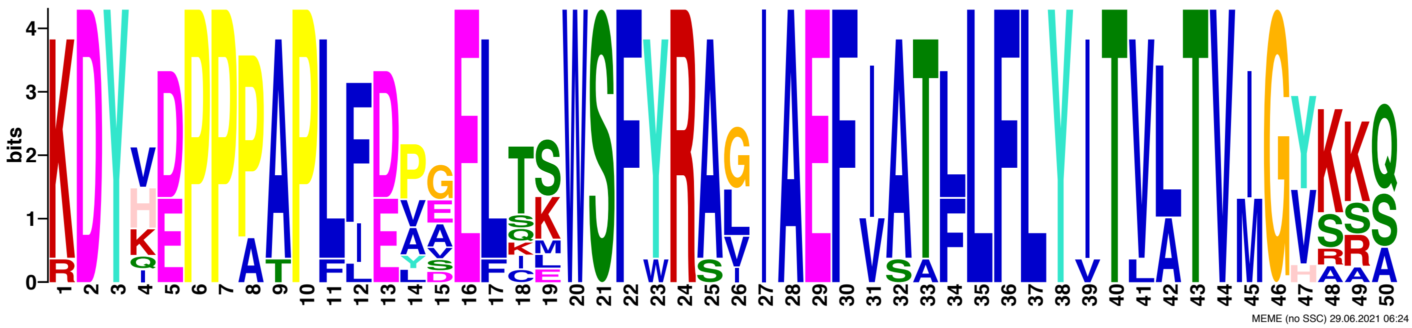

MOTIF 6

```
KDYVDPPPAPLFDPGELTSWSFYRAGIAEFIATLLFLYITVLTVIGYKKQ MEME-6,
width =  50  sites =  10  llr = 1223  E-value = 1.2 10-269.

Simplified pos.-specific probability matrix :
    A  :::::::29::::22:::::::::9::a:::91::::::::3:::::112
    C  :::::::::::::::::1::::::::::::::::::::::::::::::::
    D  :a::6:::::::6:1:::::::::::::::::::::::::::::::::::
    E  ::::4:::::::4:2a::1:::::::::a:::::::::::::::::::::
    F  ::::::::::17::::1::::a:::::::a:::4:a::::::::::::::
    G  ::::::::::::::3::::::::::4:::::::::::::::::::a::::
    H  :::3::::::::::::::::::::::::::::::::::::::::::1:::
    I  :::1:::::::2:::::1:::::::1a:::7:::::::9:::::6:::::
    K  9::2:::::::::::::13::::::::::::::::::::::::::::65:
    L  ::::::::::91:1::9:1::::::3:::::::6a:a:::17::::::::
    M  ::::::::::::::::::1:::::::::::::::::::::::::4:::::
    N  ::::::::::::::::::::::::::::::::::::::::::::::::::
    P  :::::aa8:a:::4::::::::::::::::::::::::::::::::::::
    Q  :::1:::::::::::::1:::::::::::::::::::::::::::::::5
    R  1::::::::::::::::::::::a:::::::::::::::::::::::12:
    S  ::::::::::::::1::14:a:::1::::::1:::::::::::::::223
    T  ::::::::1::::::::5::::::::::::::9::::::a::a:::::::
    V  :::3:::::::::21::::::::::2::::3:::::::1:9::a::4:::
    W  :::::::::::::::::::a::1:::::::::::::::::::::::::::
    Y  ::a::::::::::1::::::::9::::::::::::::a::::::::5:::
```

Motif 7

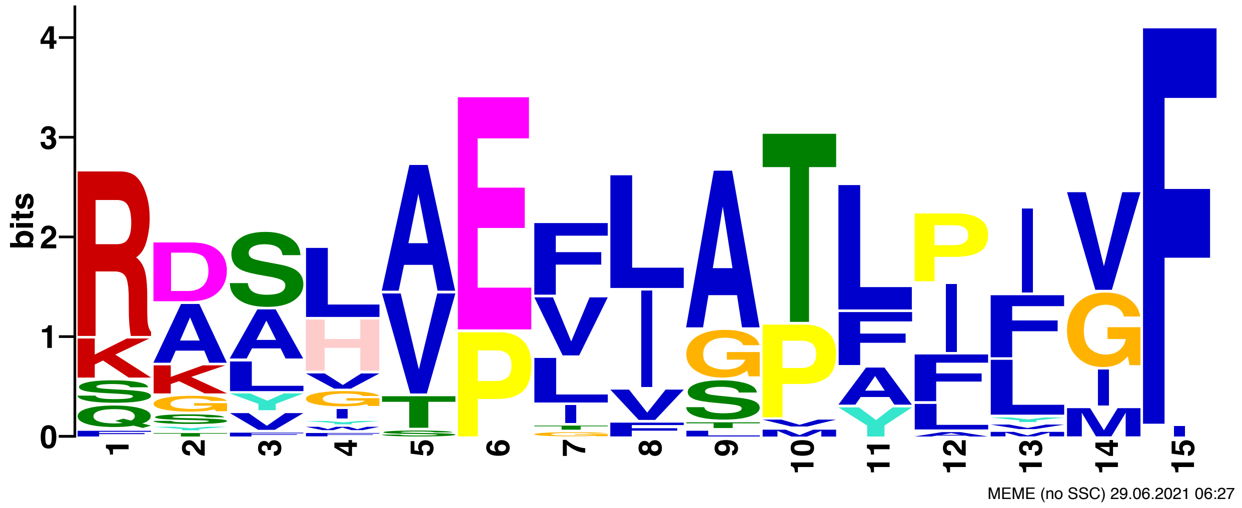

MOTIF 7

```
RDSLAEFJATLIIVF MEME-7,
width =  15  sites =  32  llr = 737  E-value = 3.7 10 116.

Simplified pos.-specific probability matrix :
     A  :33:5:::6:2::::
     C  :::::::::::::::
     D  :3:::::::::::::
     E  :::::7:::::::::
     F  ::::::31::223:a
     G  :1:1::::2::::3:
     H  :::3:::::::::::
     I  :::1::14:::342:
     K  22:::::::::::::
     L  ::24::24::513::
     M  :::::::::::::1:
     N  :::::::::::::::
     P  :::::3:::3:3:::
     Q  1::::::::::::::
     R  6::::::::::::::
     S  114:::::2::::::
     T  ::::1::::6:::::
     V  ::114:31:::::4:
     W  :::::::::::::::
     Y  ::1:::::::1::::
```

Motif 8

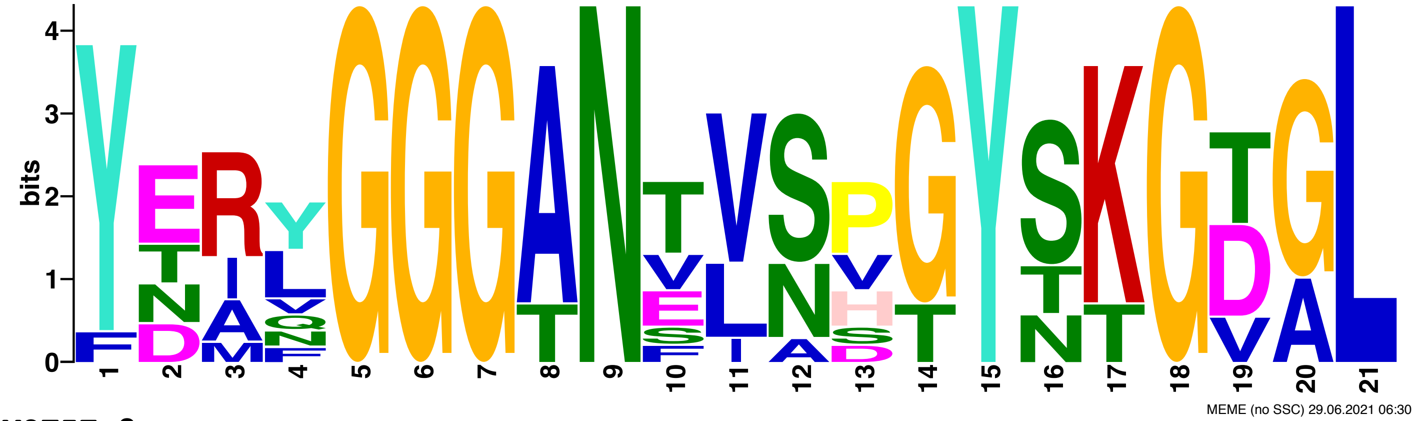


```
MOTIF 8

YERYGGGANTVSPGYSKGTGL MEME-8,
width =  21  sites =  10  llr = 451  E-value = 1.6 10-61.

Simplified pos.-specific probability matrix :
     A  ::2::::8:::1:::::::3:
     C  :::::::::::::::::::::
     D  :2::::::::::1:::::4::
     E  :4:::::::2:::::::::::
     F  1::1:::::1:::::::::::
     G  ::::aaa:::::8:::a:7:
     H  ::::::::::::2::::::::
     I  ::2:::::::1::::::::::
     K  ::::::::::::::::8::::
     L  :::3::::::3:::::::::a
     M  ::1::::::::::::::::::
     N  :2:1::::a::3:::2:::::
     P  ::::::::::::4::::::::
     Q  :::1:::::::::::::::::
     R  ::5::::::::::::::::::
     S  :::::::::1:61::6:::::
     T  :2:::::2:4:::2:22:4::
     V  :::1:::::26:2:::::2::
     W  :::::::::::::::::::::
     Y  9::3::::::::::a::::::
```

Motif 9

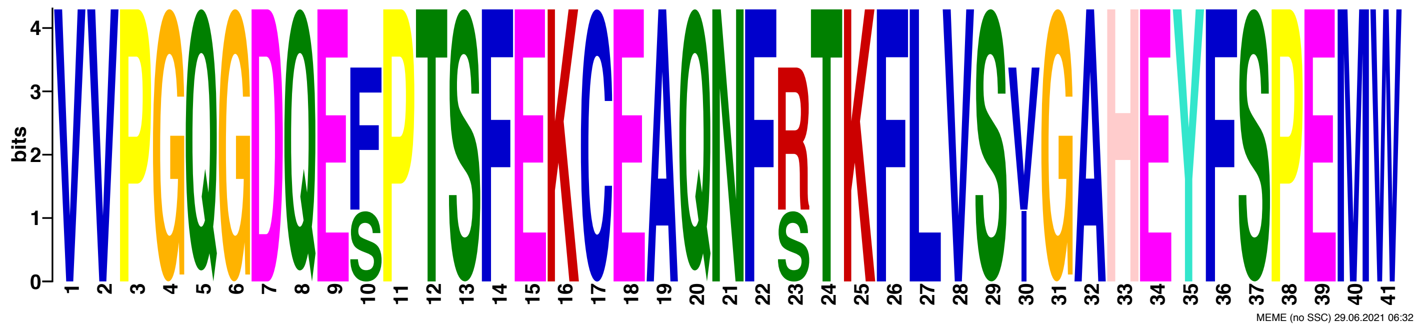

MOTIF 9

```
VVPGQGDQEFPTSFEKCEAQNFRTKFLVSVGAHEYFSPEMW MEME-9,
width =  41  sites =   3  llr = 371  E-value = 1.6 10-050.

Simplified pos.-specific probability matrix :
     A  ::::::::::::::::::a::::::::::::a:::::::::
     C  ::::::::::::::::a::::::::::::::::::::::::
     D  ::::::a::::::::::::::::::::::::::::::::::
     E  ::::::::a:::::a::a:::::::::::::::a::::a::
     F  :::::::::7:::a:::::::a:::a:::::::::a:::::
     G  :::a:a::::::::::::::::::::::::a::::::::::
     H  ::::::::::::::::::::::::::::::::a::::::::
     I  :::::::::::::::::::::::::::::3:::::::::::
     K  :::::::::::::::a::::::::a::::::::::::::::
     L  ::::::::::::::::::::::::::a::::::::::::::
     M  :::::::::::::::::::::::::::::::::::::::a:
     N  ::::::::::::::::::::a::::::::::::::::::::
     P  ::a:::::::a::::::::::::::::::::::::::a:::
     Q  ::::a::a:::::::::::a:::::::::::::::::::::
     R  ::::::::::::::::::::::7::::::::::::::::::
     S  :::::::::3::a:::::::::3:::::a:::::::a::::
     T  :::::::::::a:::::::::::a:::::::::::::::::
     V  aa:::::::::::::::::::::::::a:7:::::::::::
     W  ::::::::::::::::::::::::::::::::::::::::a
     Y  ::::::::::::::::::::::::::::::::::a::::::
```

Motif 10

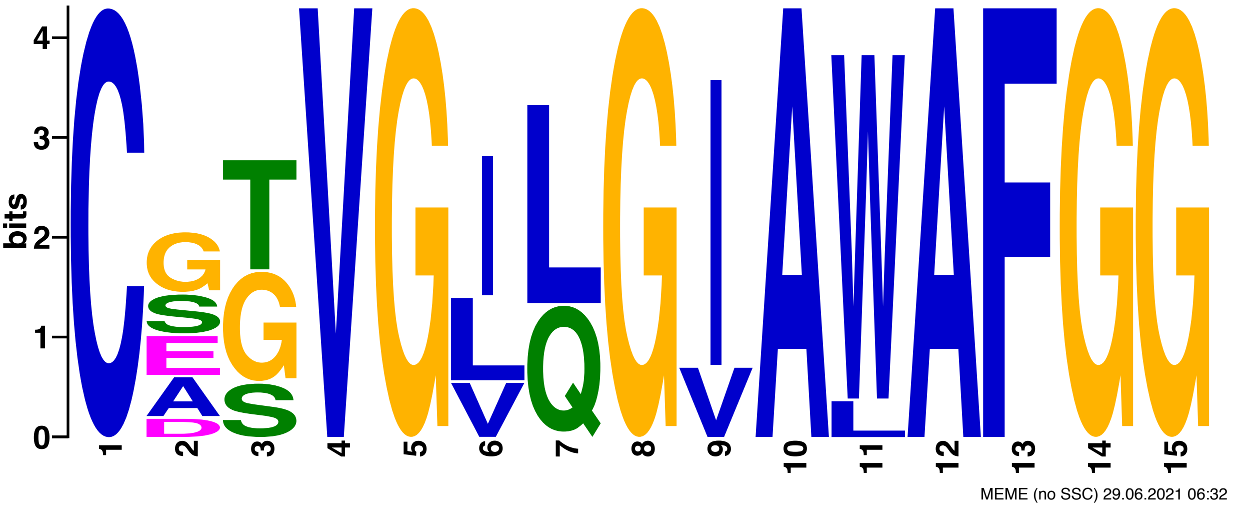

MOTIF 10

```
CGTVGILGIAWAFGG MEME-10,
width =  15  sites =  10  llr = 346  E-value = 2.3 10-48.

Simplified pos.-specific probability matrix :
     A  :2:::::::a:a:::
     C  a::::::::::::::
     D  :1:::::::::::::
     E  :2:::::::::::::
     F  ::::::::::::a::
     G  :34:a::a:::::aa
     H  :::::::::::::::
     I  :::::5::8::::::
     K  :::::::::::::::
     L  :::::36:::1::::
     M  :::::::::::::::
     N  :::::::::::::::
     P  :::::::::::::::
     Q  ::::::4::::::::
     R  :::::::::::::::
     S  :22::::::::::::
     T  ::4::::::::::::
     V  :::a:2::2::::::
     W  ::::::::::9::::
     Y  :::::::::::::::
```
