# Supplementary material for: Genome-Wide Identification, Structure Characterization, and Expression Pattern Profiling of the Aquaporin Gene Family in Betula pendula
Source: Int J Mol Sci. 2021 Jul 6;22(14):7269. doi: 10.3390/ijms22147269 (PMC8304918; doi:10.3390/ijms22147269)
Supplement: Supplementary file 1 [file ijms-22-07269-s001.zip › Supplementary Files Betula MIPs/FigureS4.pdf]

Figure S4. Manual analysis of the *Bpe*XIP2 group leading to the theoretical redefinition of a *Bpe*XIP2;1 used in this work.
This manual reconstruction took into account orthologous sequences from *B. platyphylla* and *B. nana* species, and related reads from the RNA sequencing used for the transcriptional expression analyses.
The original *B. pendula* sequences which were retrieval in the public databases (CoGe, NCBI) are : Bpev01.c2937.g0004 for a full-length sequence (named in this Figure *Bpe*XIP2;1), and Bpev01.c1577.g0027 for a truncated sequence (named in this Figure *Bpe*XIP2;1-like, "-like" in reference to a pseudogene sequence).
The redefined "*Bpe*XIP2;1" sequence used for this work was named *Bpe*XIP2;1.

```
                              10        20        30        40        50        60        70        80        90       100       110
                               |         |         |         |         |         |         |         |         |         |         |
BpeXIP2;1             MAQNVQRVEDEESLYVGSRVQPEGNRNTSKLSEILGLEEFFSLNVWRASLAELLATAIFVFVVDTIVISSFETKMESPKLIMSLLIAIITAILLLAANPVSGGHINPVIT
BpeXIP2;1             MAQNVQRVEDEESLYVGSRVQPEGNRNTSKLSEILGLEEFFSLNVWRASLAELLATAIFVFAIDTIVISSFETKTESPKLIMSLLIAIITAILLLAANPISGGHMNPAIT
BPlaty_Chr09G20962    MAQNVQRAEDEESLPDQRRFE-EGNRNTSKLSEILGLEEFFSLNVWRASLAELLATAIFVFVVDTIVISSFETKMESPKLIMSLLIAIITAILLLAANPVSGGHINPVIT
Bnana_CAOK010027221   MAQNVQRAEDEESLPDQRRFE-EGNRNTSKLSEILGLEEFFSLNVWRASLAELLATAIFVFVVDTIVISSFETKMESPKLIMSLLIAIITAILLLAANPVSGGHINPVIT
BpeXIP2;1_like        --------------------------------------------VWRASLAELLATAIFVFVVDTIVISSFETKMESPKLIMSLLIAIITAILLLAANPVSGGHINPVIT
BPlaty_Chr09G20935    --------------------------------------------VWRASLAELLATAIFVFVVDTIVISSFETKTESPKLIMSLLIAIITAILLLAANPISGGHMNPAIT
Bnana_CAOK010205141   --------------------------------------------VWRASLAEFLATAIFVFAIDTIVISSFETKTESPKLIMSLLIAIITAILLLAANPISGGHMNPAIT

                             120       130       140       150       160       170       180       190       200       210       220
                               |         |         |         |         |         |         |         |         |         |         |
BpeXIP2;1New          LVAALVGLISVSRAAVYILAQCAGAVLGALALKAVVNNTIEDTFSLGGCTLTVIAPGPNGPISVGLGTSQGLWLEIICTFVLLFASVWIALDHRQARALGKVIIFSVIGI
BpeXIP2;1             LAAALVGVISVSRAAVYILAQCAGAVLGALALKAVVNSTIEDTFSLGGCTLTVIAPGPNGPISVGLGTSQGLWLEIICTFVLLFASIWIALDHRQARALGRVIIFSVIGI
BPChr09G20962         LVAALVGLISVSRAAVYILAQCAGAVLGALALKAVVNNTIEDTFSLGGCTLTVIAPGPNGPISVGLGTSQGLWLEIICTFVLLFASVWIALDHRQARALGKVIIFSVIGI
Bnana_CAOK010027221   LVAALVGLISVSRAAVYILAQCAGAVLGALALKAVVNSTIEDTFSLGGCTLTVIAPGPNGPISVGLGTSQGLWLEIICTFVLLFASIWIALDHRQARALGRVIIFSVIGI
BpeXIP2;1_like        LVAALVGLISVSRAAVYILAQCAGAVLGALALKAVVNNTIEDTFSLGGCTLTVIAPGPNGPISVGLGTSQGLWLEIICTFVLLFASVWIALDHRQARALGKVIIFSVIGI
BPChr09G20935         LAAALVGVISVSRAAVYILAQCAGAVLGALALKAVVNSTIEDTFSLGGCTLTVIAPGPNGPISVGLGTSQGLWLEIICTFVLLFASIWIALDHRQARALGRVIIFSVIGI
Bnana_CAOK010205141   LAAALVGVISVSRAAVYILAQCAGAVLGALALKAVVNNTIEDTFSLGGCTLTVIAPGPNGPISVGLGTSQGLWLEIICTFVLLFASIWIALDHRQARALGRVIIFSVIGI

                             230       240       250       260       270       280       290       300       310
                               |         |         |         |         |         |         |         |         |
BpeXIP2;1New          VVGLLVFVSTTVTGAKGYAGAGMNPARCLGPAIVRGGHLWNGHWVFWAGPSIASVAFYLYTKIIPSQHFHAQGYKHDFLNILKAPFTSDSVHKK
BpeXIP2;1             VVGLLVFVSTTVTGAKGYAGAGMNPARCLGPAIVRGGHLWNGHWVFWAGPSIASVAFYLYTKIIPSQHFHAQGYKHDFLNILKAPFTSDSVHKK
BPChr09G20962         VVGLLVFVSTTVTGAKGYAGAGMNPARCLGPAIVRGGHLWNGHWVFWAGPSIASVAFYLYTKIIPSQHFHAQGYKHDFLNILKAPFTSDSVHKK
Bnana_CAOK010027221   VVGLLVFVSTTVTGAKGYAGAGMNPARCLGPAIVRGGHLWNGHWVFWAGPSIASVAFYLYTKIIPSQHFHAQGYKHDFLNILKAPFTSDSVHKK
BpeXIP2;1_like        VVGLLVFVSTTVTGAKGYAGAGMNPARCLGPAIVRGGHLWNGHWVFWAGPSIASVAFYLYTKIIPSQHFHAQGYKHDFLNILKAPFTSDSVHKK
BPChr09G20935         VVGLLVFVSTTVTGAKGYAGAGMNPARCLGPAIVRGGHLWNGHWVFWAGPSIASVAFYLYTKIIPSQHFHAQGYKHDFLNILKAPFTSDSVHKK
Bnana_CAOK010205141   VVGLLVFVSTTVTGAKGYAGAGMNPARCLGPAIVRGGHLWNGHWVFWAGPSIASVAFYLYTKIIPSQHFHAQGYKHDFLNILKAPFTSGSVHKK
```
