# Supplementary material for: Genome-Wide Identification, Structure Characterization, and Expression Pattern Profiling of the Aquaporin Gene Family in Betula pendula
Source: Int J Mol Sci. 2021 Jul 6;22(14):7269. doi: 10.3390/ijms22147269 (PMC8304918; doi:10.3390/ijms22147269)

Figure S5

Figure S5. Predicted 3D models of the *Bpe*AQP monomers (*Bpe*PIP1s; *Bpe*PIP2s; *Bpe*TIPs; *Bpe*XIPs; *Bpe*NIPs; *Bpe*SIPs). Structures were generated by using the PHYRE2.0 Protein Fold Recognition server, using the Normal mode modeling based on alignment to experimentally solved protein structures, and built with the Mole2.5 software. Each structure is supported by the hydropathy and charge, indexes of the channel (right of the 3D modelisation), obtained by MOLE2.5 software. Each member is represented in lateral view. Models were visualized by rainbow color from *N*- to *C*-terminus, where the intracellular (B,D) and extracellular (A,C,E) loops, and transmembrane helices are shown, as firstly illustrated with the control structure (*So*PIP2,1 from *Spinacia oleracea*) with its lateral and top views, plus a representation of the structure embedded in the membrane.

Control structure, *So*PIP2;1

Lateral view

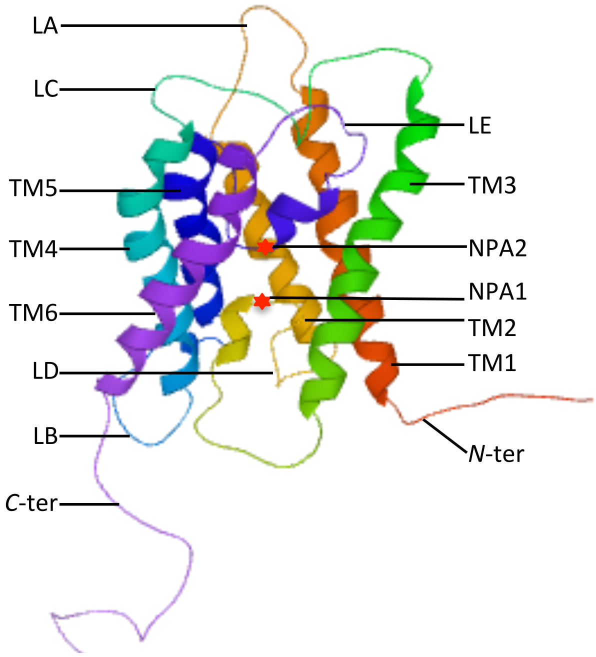

*So*PIP2;1 structure embedded in the membrane

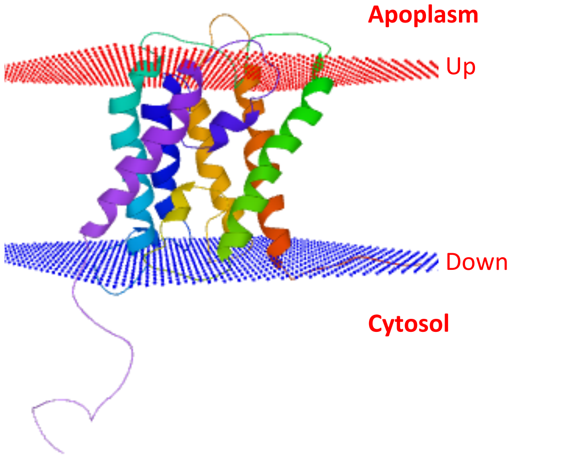

Top view (apoplastic face)

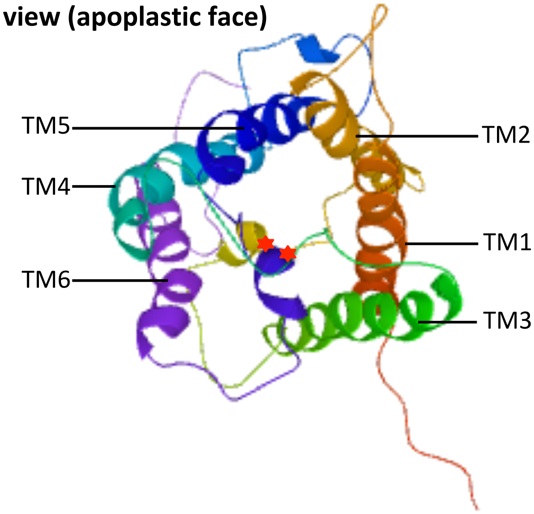

Physicochemical properties of *So*PIP2;1pore structure

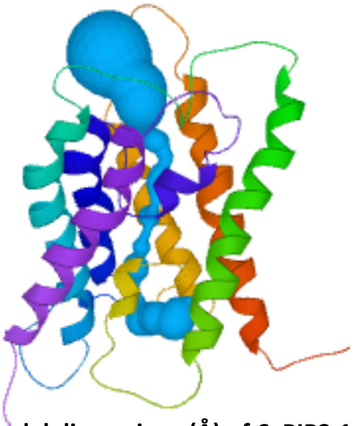

Model dimensions (Å) of *So*PIP2;1

X:76.562 **Y**:49.788 **Z**:51.612

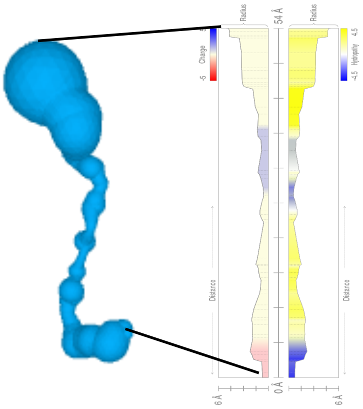

Length of the channel : 54 Å
Radius of the channel bottleneck : 0.6 Å
Average of hydropathy : 1.27
Charge : 0
Polarity (Average of lining amino acd polarities) : 8.47
Mutability, Average of relative mutability : 86
Lipophilicity, logP-scale : 1.04
Lipophilicity, logD-scale : 0.85
Ionizable residues : 2

*Bpe*PIP1s

*Bpe*PIP1;1

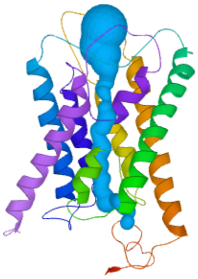

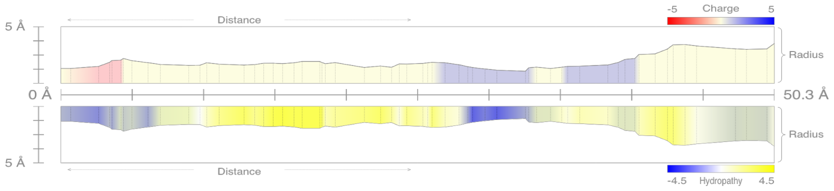

*Bpe*PIP1;2

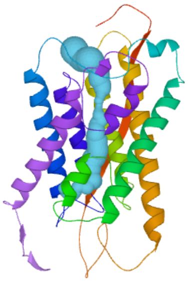

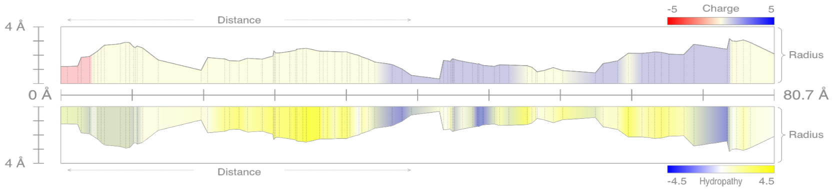

*Bpe*PIP1;3

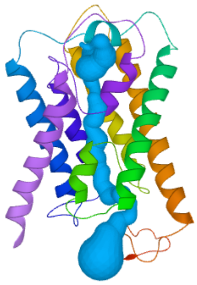

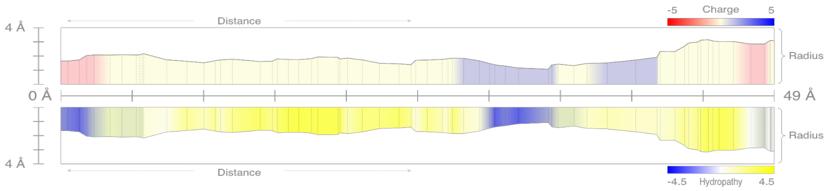

*Bpe*PIP1;4

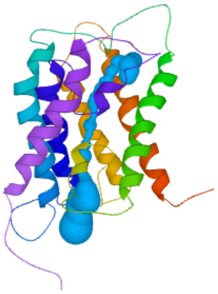

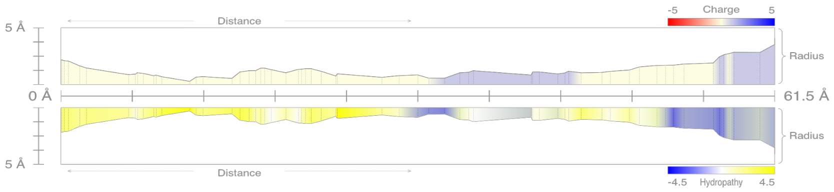

*Bpe*PIP2s

*Bpe*PIP2;1

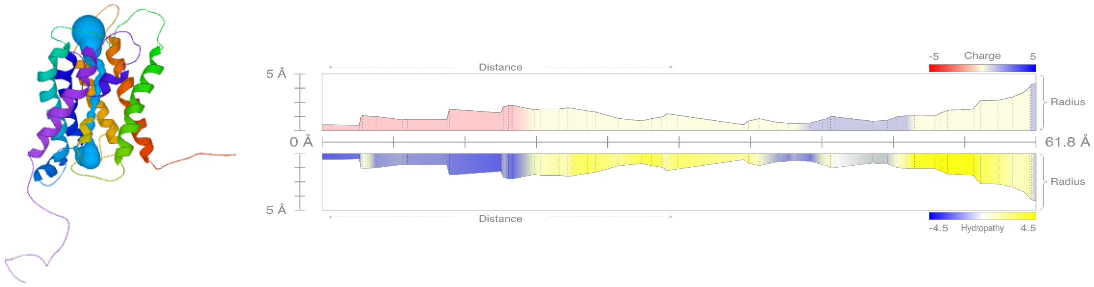

*Bpe*PIP2;2

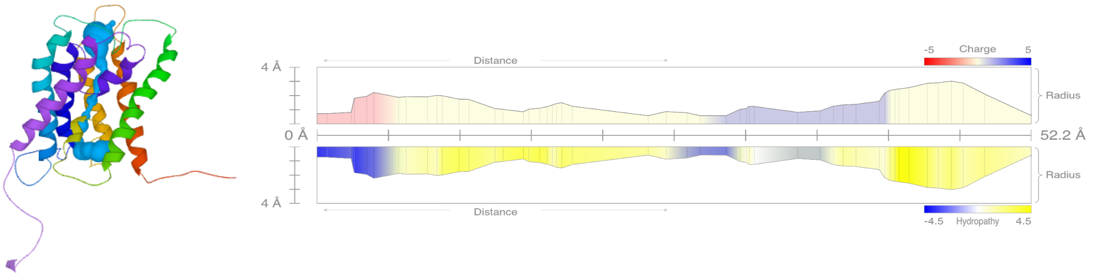

*Bpe*PIP2;3

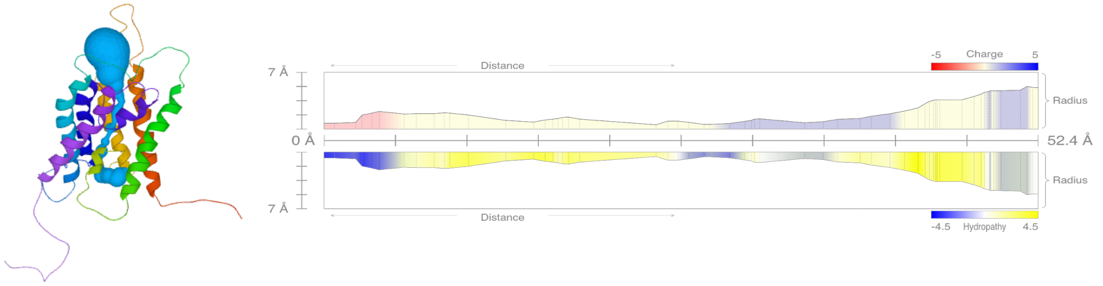

*Bpe*PIP2;4

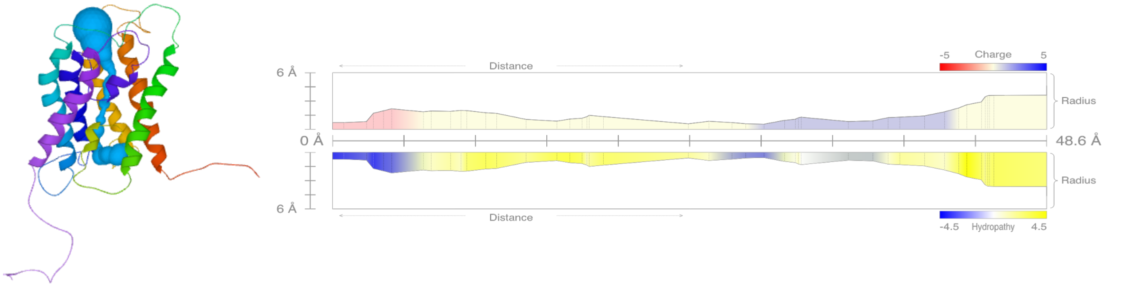

*Bpe*PIP2;5

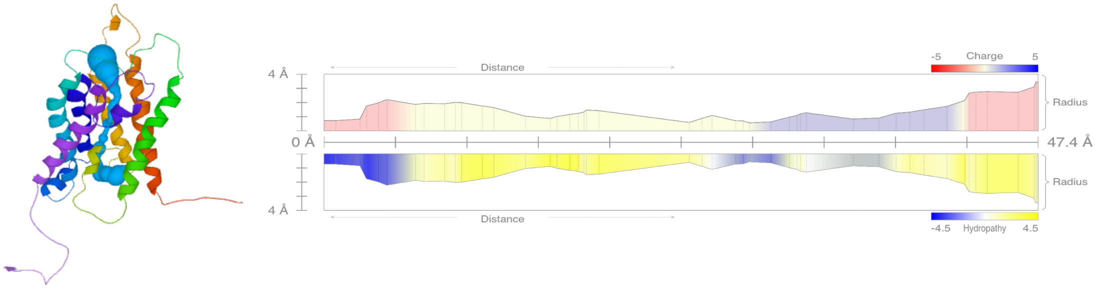

*Bpe*PIP2;6

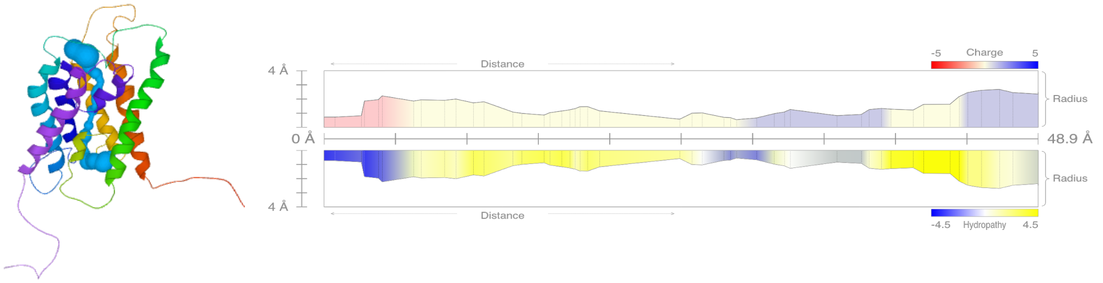

*Bpe*TIPs

*Bpe*XIP1;1

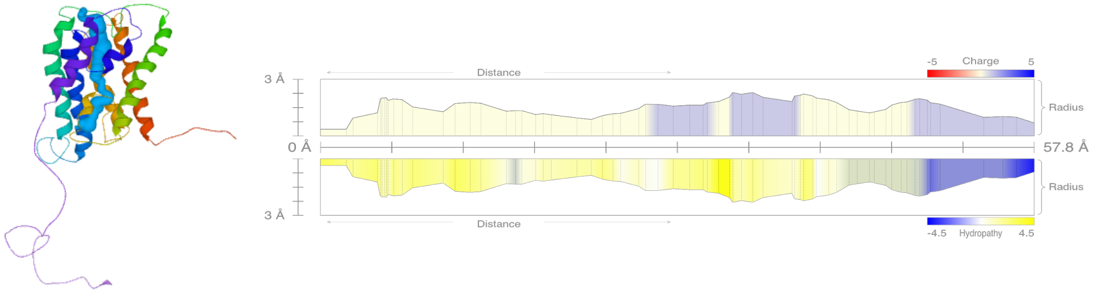

*Bpe*XIP1;2

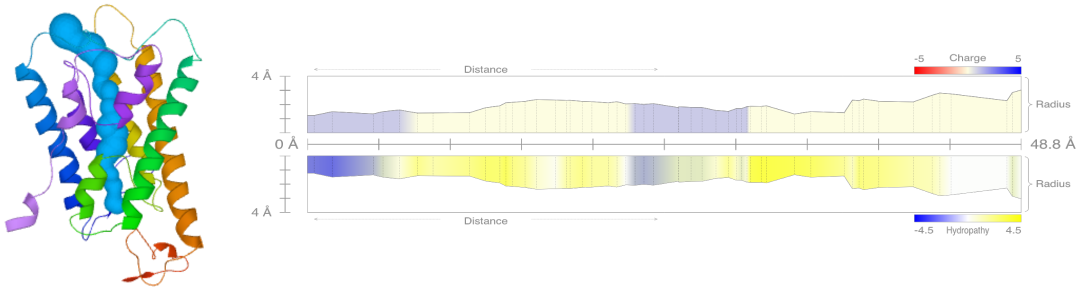

*Bpe*XIP1;3

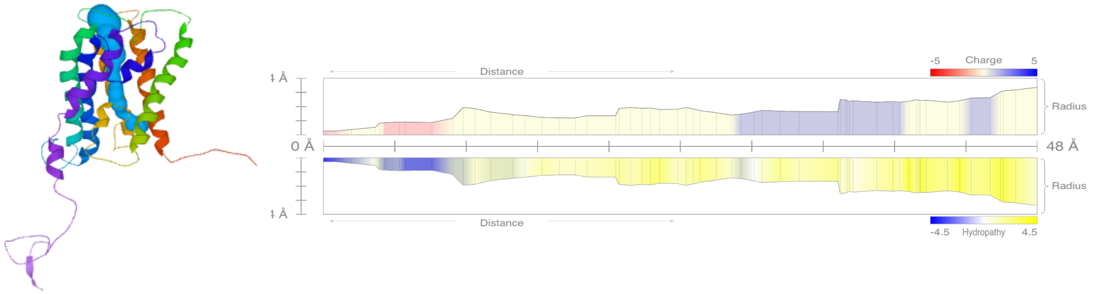

*Bpe*XIP2;1

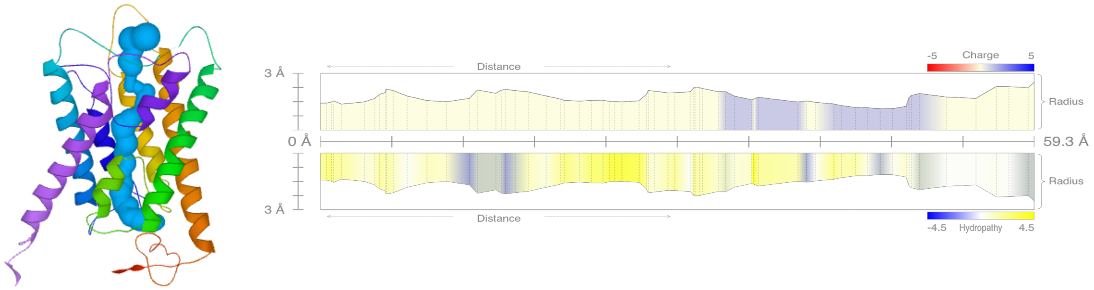

*Bpe*TIPs

***Bpe*TIP1;1**

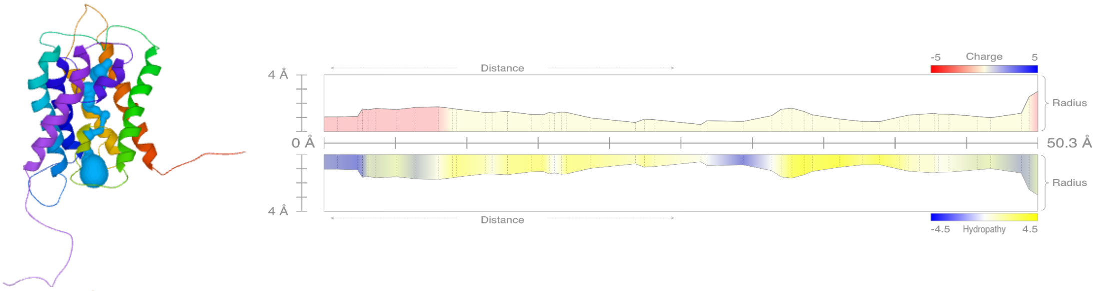

***Bpe*TIP1;2**

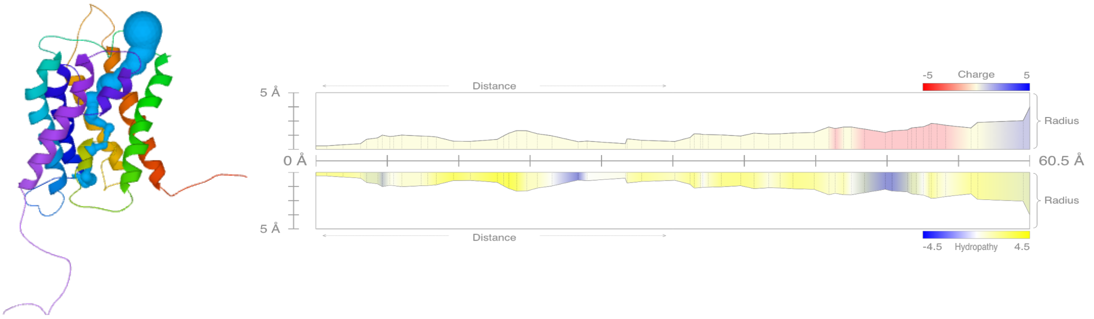

***Bpe*TIP1;3**

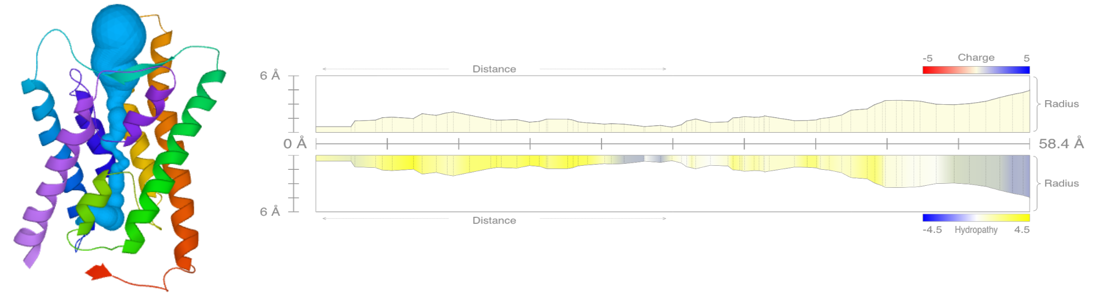

***Bpe*TIP2;1**

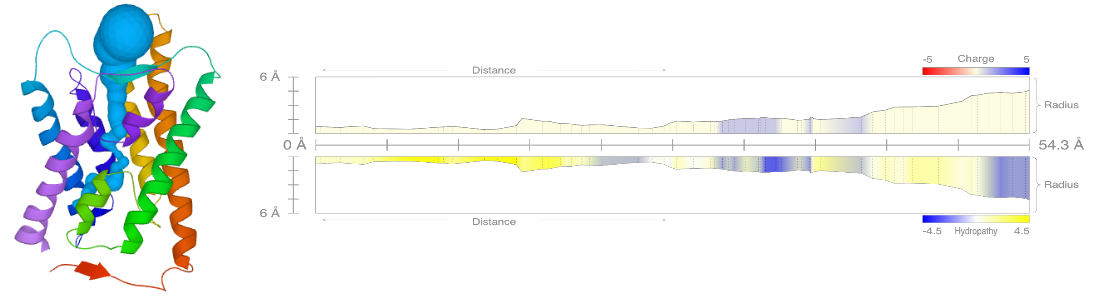

***Bpe*TIP2;2**

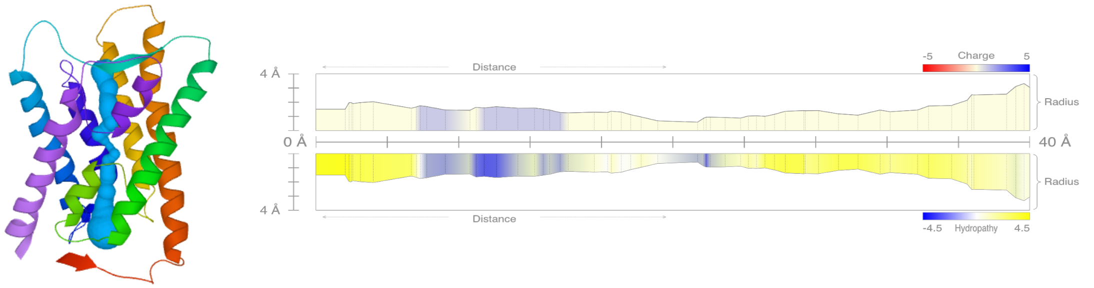

***Bpe*TIP3;1**

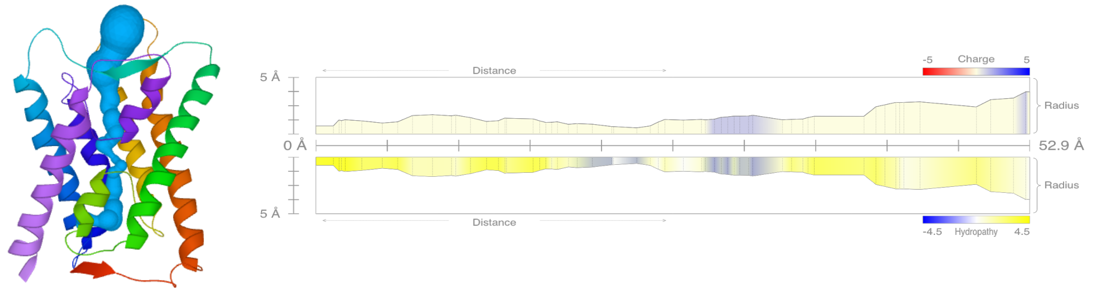

***Bpe*TIPs**

***Bpe*TIP4;1**

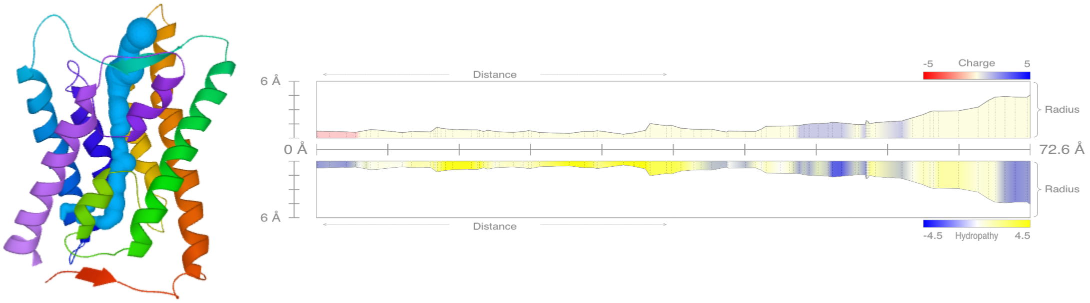

***Bpe*TIP5;1**

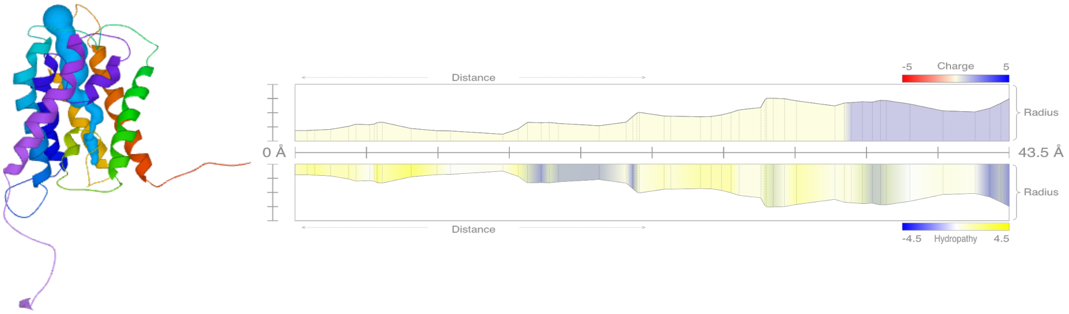

***Bpe*NIPs**

***Bpe*NIP1;1**

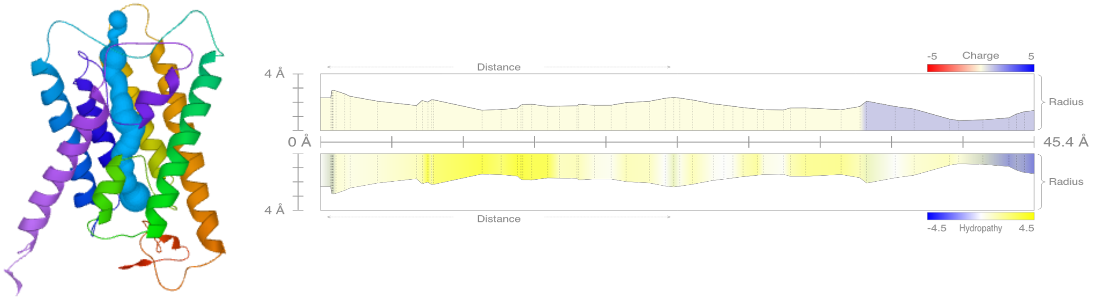

***Bpe*NIP1;2**

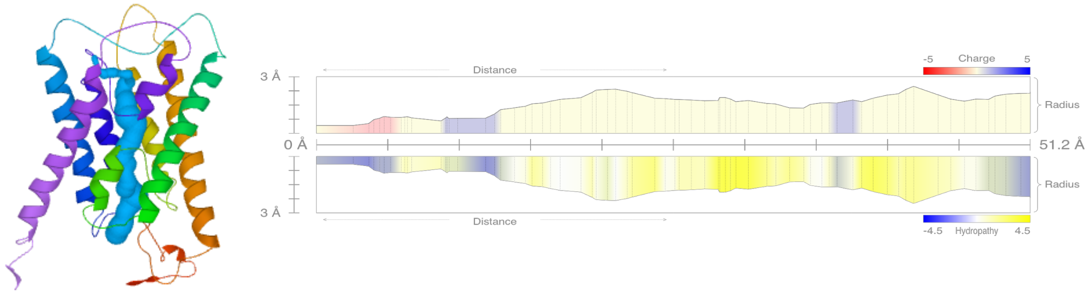

***Bpe*NIP2;1**

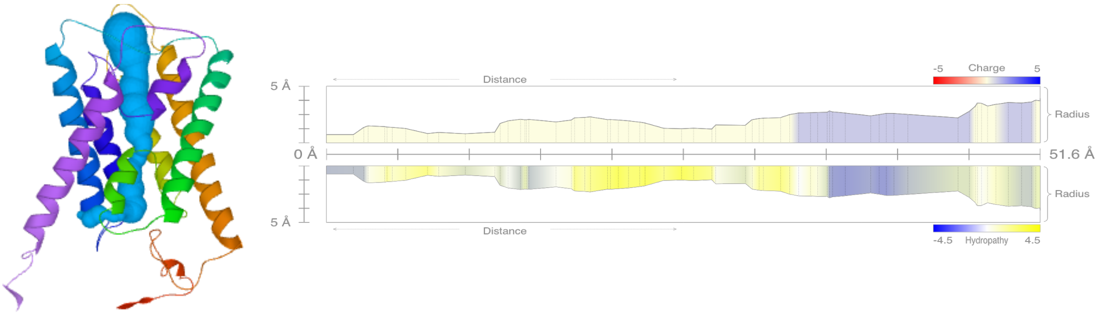

***Bpe*NIPs**

***Bpe*NIP4;1**

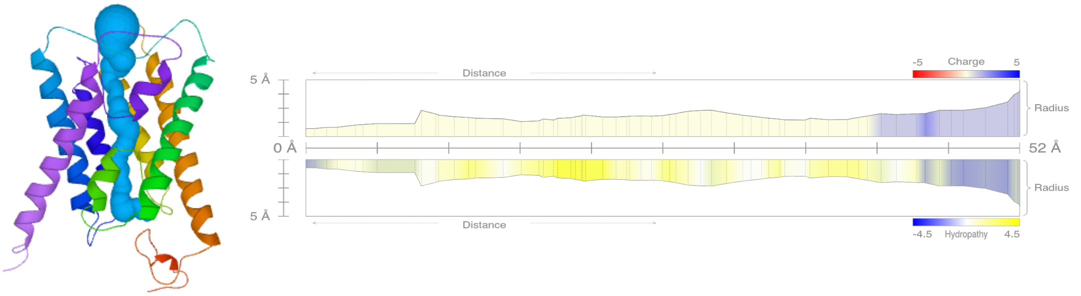

***Bpe*NIP5;1**

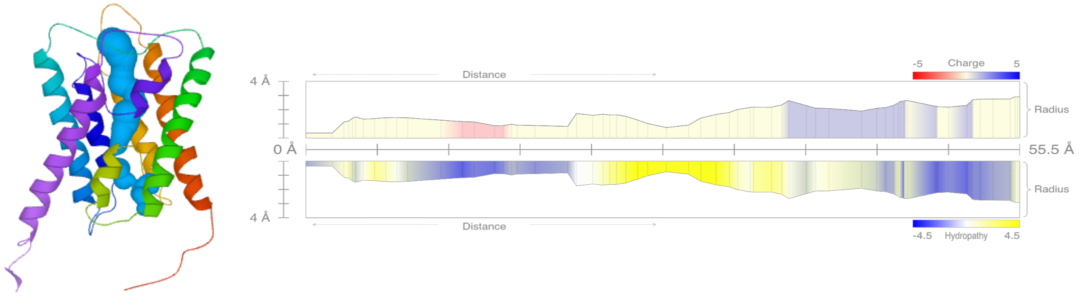

***Bpe*NIP5;2**

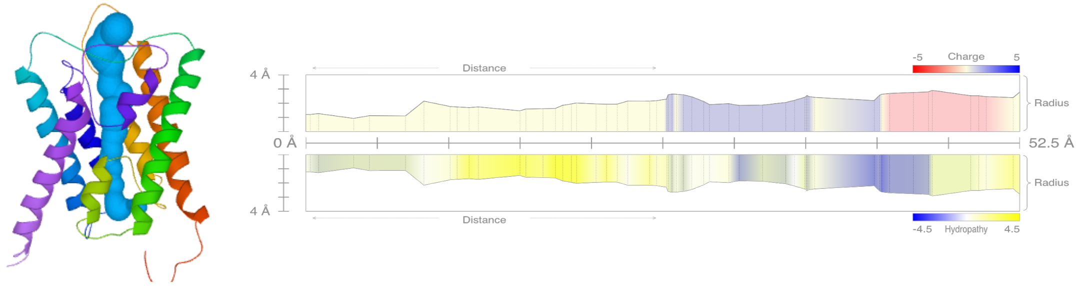

***Bpe*NIP6;1**

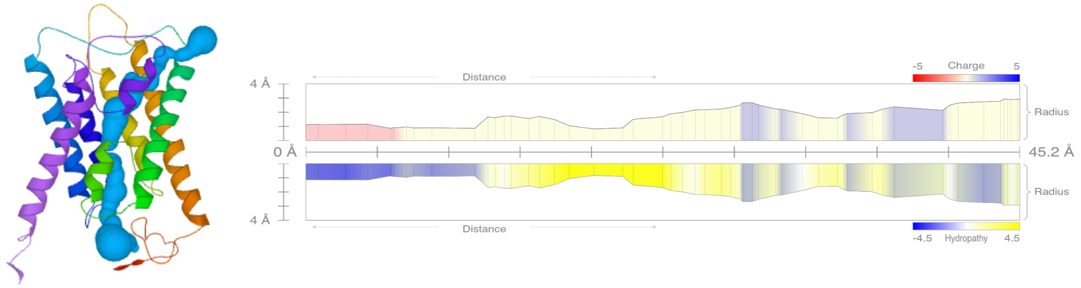

***Bpe*NP7;1**

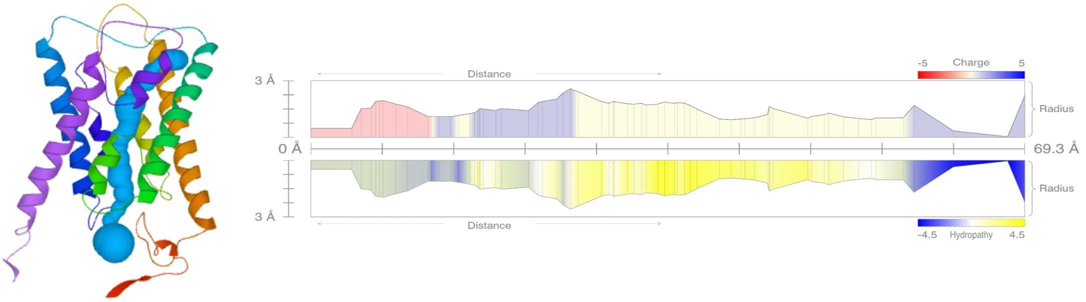

*Bpe*SIP1

*Bpe*SIP1;1

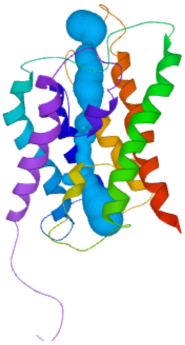

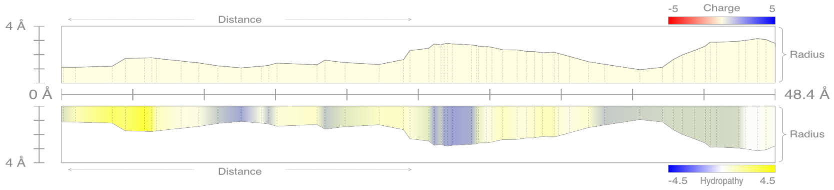

*Bpe*SIP1;2

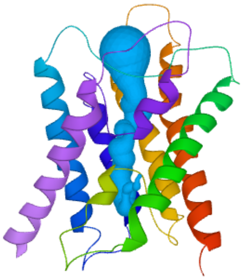

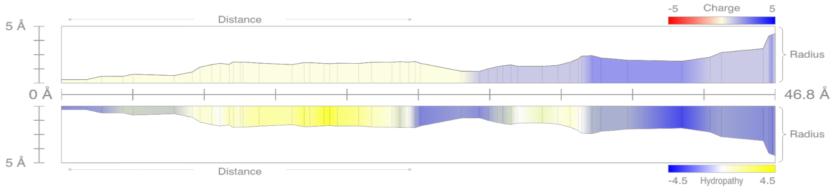

*Bpe*SIP2;1

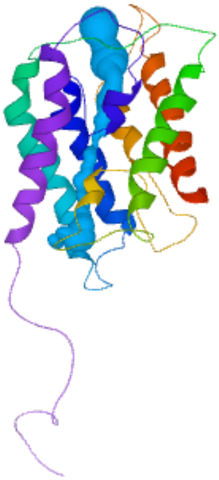

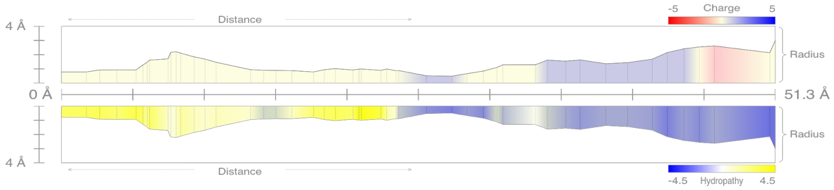

Supplement: Supplementary file 1 [file ijms-22-07269-s001.zip › Supplementary Files Betula MIPs/FigureS5.pdf]
