# Supplementary material for: Genome-Wide Identification, Structure Characterization, and Expression Pattern Profiling of the Aquaporin Gene Family in Betula pendula
Source: Int J Mol Sci. 2021 Jul 6;22(14):7269. doi: 10.3390/ijms22147269 (PMC8304918; doi:10.3390/ijms22147269)
Supplement: Supplementary file 1 [file ijms-22-07269-s001.zip › Supplementary Files Betula MIPs/FigureS6.pdf]

Figure S6. Amino acid sequence alignments per subfamily of the *Bpe*AQP members from *Betula pendula*. S6A, PIP; **S6B**, TIP; **S6C**, XIP; **S6D**, NIP; **S6E**, SIP. Legends are detailed at the end of each subfamily alignment.

Plasma membrane intrinsic proteins (PIPs)

```
                     10        20        30        40        50        60        70        80        90       100
                      |         |         |  *      |         |         |         |         |         |         |
AtPIP1;1     -------MEGKEEDVRVGANKFPERQPIGT--SAQSD---KDYKEPPPAPFFEPGELSSWSFWRAGIAEFIATFLFLYITVLTVMGVKRSPNM------CASVGI
SlPIP1;1     ------MAENKEEDVNLGANKFREPQPLGT--SAQTD---KDYKEPPPAPLYEPGELSSWSFYRAGIAEFMATFLFLYITILTVMGLKRSDSL------CSSVGI
BpePIP1;1    -------MEGKDEDVRLGANRYRERQPIGT--AAQSQDT-KDYQEPPAAPFFEPGELSSWSFYRAGIAEFVATFLFLYITVLTVMGVSKSPSK------CSTVGI
BpePIP1;3    -------MEGKEEDVRLGANKFPERQPIGT--AAQSQDEGKDYIEPPPAPLFEPSELTSWSFYRAGIAEFVATFLFLYITVLTVMGVAKSPSK------CSTVGI
BpePIP1;2    -------MEGKEEDVRLGANKYTERQPLGT--AAQTD---KDYKEPPPAPLFEPGELCSWSFYRAGIAEFVATFLFLYITVLTVMGVKRSSSV------CASVGI
BpePIP1;4    -------MEGKEEDVKVGASKFPERQAIGT--SAQTD---KDYKEPPPAPLFEPGELQSWSFWRAGIAEFIATFLFLYITLLTVMGVSRAPSK------CASVGV
AtPIP2;1     -------------------------MAKDVEAVPGEG-FQTRDYQDPPPAPFIDGAELKKWSFYRAVIAEFVATLLFLYITVLTVIGYKIQSDTDAGGVDCGGVGI
SlPIP2;1     MVRHEWLKESTRAKKQRQPKKKTETQKWAKIWRLVLNMHQKTYQDPPPAPLIDPEELGKWSFYRAIIAEFIATLLFLYITVLTVIGYKSQSST----DQCGGVGI
BpePIP2;1    -------------------------MSKEVS-EEGQSHQHGKDYVDPPPAPLLDLAELKLWSFYRSLIAEFIATLLFLYVTVATVIGHKKATGP------CDGVGL
BpePIP2;2    -------------------------MSKEVT-EEREASQPVKDYVDPPATPLFDVAEFTMWSFYRAVIAEFIAALLFLYITVATVIGYKKQTDP------CETVGL
BpePIP2;3    -------------------------MSKEVT-EEAEASQPVRDYVDPPPAPLFDVEELTEWSFYRAVIAEFISTLLFLYITVATVIGYKKQNDQ------CETVGL
BpePIP2;4    -------------------------MAKDAEVPEH-GSFTAKDYHDPPPAPLFDAVELTKWSFYRALIAEFIATLLFLYITVLTVIGYKSQIDPANDGEACGGVGV
BpePIP2;5    -------------------------MAKDVEVAEHQGEYSAKDYHDPPPAPLIDYDELIKWSFYRALIAEFIATLLFLYITVLTVIGYKSQTDPSKNPDQCGGVGI
BpePIP2;6    -------------------------MAKDMEVGGQSG-FSAKDYHDPPPAPLIDAEELTKWSFYRAIIAEFIATLLFLYITVLTVIGYRAQSDTTRGGDDCGGVGI
                                                                         *
                                                                             -----------TM1---------               ---


                    115       125       135       145       155       165       175       185       195       205
                      |         |         |         |         |         |         |         |         |         |
AtPIP1;1     QGIAWAFGGMIFALVYCTAGISGGHINPAVTFGLFLARKLSLTRALYYIVMQCLGAICGAGVVKGFQPKQYQALGGGANTVAHGYTKGSGLGAEIIGTFVLVYTV
SlPIP1;1     QGVAWAFGGMIFALVYCTAGISGGHINPAVTFGLFLARKLSLTRAVFYMVMQCLGAICGAGVVKGFMQGPYQRLGGGANVVQPGYTKGDGLGAEIIGTFVLVYTV
BpePIP1;1    QGIAWAFGGMIFALVYCTAGISGGHINPAVTFGLFLARKLSLTRAVFYIVMQCLGAICGAGVVKGFQKNQYERLGGGANTISSGYSKGDGLGAEIVGTFVLVYTV
BpePIP1;3    QGIAWAFGGMIFALVYCTAGISGGHINPAVTFGLFLARKLSLTRALFYIVMQCLGAICGAGVVKGFQKSQYEILGGGANFVNPGYTKGDGLGAEIVGTFVLVYTV
BpePIP1;2    QGIAWAFGGMIFALVYCTAGISGGHINPAVTFGLLLARKLSLTRAVFYIIMQCLGAICGAGVVKGFGKTPFEINGGGANVVNHGYTKGDGLGAEIVGTFVLVYTV
BpePIP1;4    QGIAWAFGGMIFALVYCTAGISGGHINPAVTFGLFLARKLSLTRALFYIVMQCLGAICGAGVVKGFQKTQYERVGGGANVVNHGYSKGDGLGAEIVGTFVLVYTV
AtPIP2;1     LGIAWAFGGMIFILVYCTAGISGGHINPAVTFGLFLARKVSLPRALLYIIAQCLGAICGVGFVKAFQSSYYTRYGGGANSLADGYSTGTGLAAEIIGTFVLVYTV
SlPIP2;1     LGIAWAFGGMIFVLVYCTAGISGGHINPAVTFGLFLARKVSLVRAIMYIVAQCLGAICGCGLVKAFQKAYYVKYGGGANTLNDGYSTGTGLGAEIIGTFVLVYTV
BpePIP2;1    LGIAWAFGGMIFVLVYCTAGISGGHINPAVTFGLFLARKVSLIRAVSYMVAQCLGAICGVGLVKAFMKHDYNMQGGGANSVSPGYSTGTALGAEIIGTFVLVYTV
BpePIP2;2    LGVAWAFGGMIFVLVYSTAGISGGHINPAVTFGLFLARKVSLIRAICYMVAQCLGAICGAGLVKGIMKDSYDAFGGGTNTVSPTYSKGTALGAEIIGTFVLVYTV
BpePIP2;3    LGVAWAFGGMIFILVYSTAGISGGHINPAVTFGLFLARKVSLIRAICYMVAQCLGAICGAGLVKGIMKDYYDALGGGTNTVSPTYNKGTALGAEIIGTFVLVYTV
BpePIP2;4    LGIAWAFGGMIFVLVYCTAGISGGHINPAVTFGLFLARKVSLVRAVSYMVAQCLGAISGVGLVKAFNKAHYTRYGGGANTLADGYSTGVGLGAEIIGTFVLVYTV
BpePIP2;5    LGIAWAFGGMIFVLVYCTAGISGGHINPAVTFGLFLARKVSLIRALLYMVAQCLGAICGVGLVKAFQKSDYNRYGGGANELSVGYNKGVGLGAEIIGTFVLVYTV
BpePIP2;6    LGIALAFGGMIFVLVYCTAGISGGHINPAVTFGLFLARKVSLVRAILYMVAQCLGAICGCGLVKAFQRAHYTRYGGGANELSVGYSKGTGLAAEIIGTFVLVYTV
                   H2                   -NPA;LB-     *                         P1
             ---TM2---------                             -----------TM3---------                                ---------TM4------
```

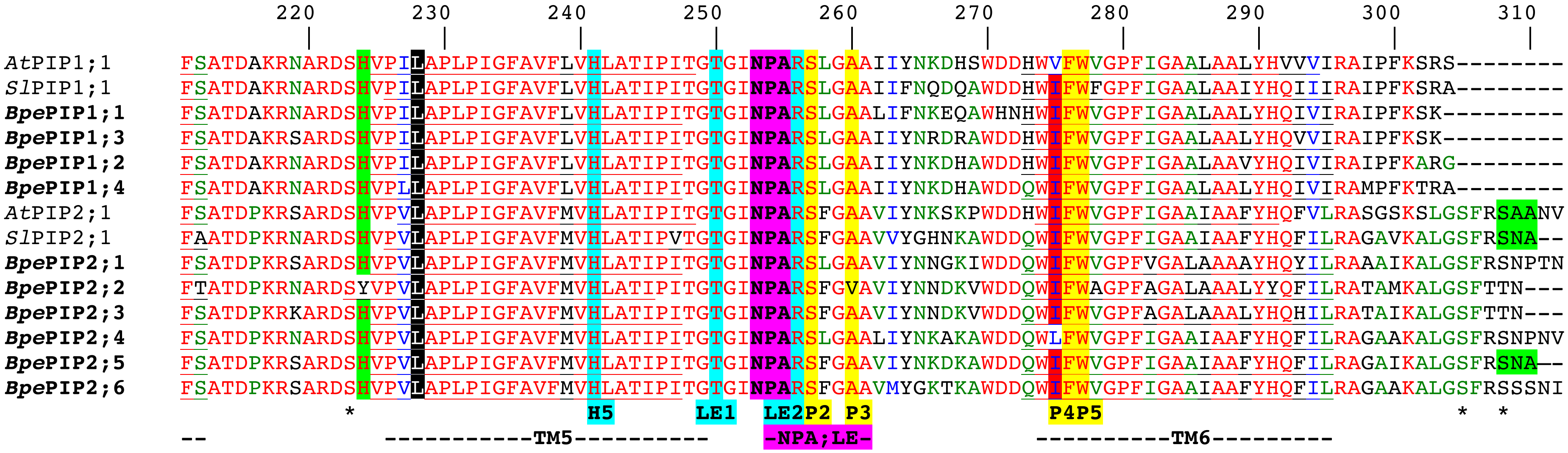

Figure S6A. Amino acid sequence alignment of the *Bpe*PIP subfamily members from *Betula pendula*. *Sl*PIP1;1 and *Sl*PIP2;1 (Solyc10g054840.1.1; Reuscher et al., 2013) from Tomato, and *At*PIP1;1 (At3g61430) and *At*PIP2;1 (At3g53420) (Muries et al., 2011) from *Arabidopsis thaliana* were used as references. The highest residue similarities between MIP members are shown in red (100%), and green (>80%). Residues with similar physicochemical behaviors are shown in blue. The six transmembrane regions (TM1-TM6) and the two hemi-helicoidal regions that include the «NPA» motives «NPA1» (in Loop B) and «NPA2» (in Loop E) (framed in purple) are underlined. The amino acids residues constituting the ar/R constriction region are shown with blue highlights and labeled H2, H5, LE1 and LE2. Residues constituting the Froger's positions are shown with yellow highlights and labeled P1 to P5. In addition, AEF motif, the His residue involved in gating, the RKXSXXR motif, the SxA motif for putative phosphorylation by PKC are marked in green, the KdvE motif for putative conserved methylation sites are marked in red, and the putative conserved blocking residue (L) is marked in black. Lastly, the Cysteines (C) involved in the tetramerization of PIPs are mentioned in purpled. Columns or regions with conserved putative phosphorylation sites are marked with an asterisk. Isoleucine (I) at the position 275 on the alignment and surligned in red and placed in loop E would be key for the transport of CO_2. Serine (S) at the position 146 on the alignment and surligned in red would be key for the transport of H_2O_2.

Tonoplast intrinsic proteins (TIPs)

```
                   10        20        30        40        50        60        70        80        90       100
                    |         |         |         |         |         |         |         |         |         |
AtTIP1;2   -MPTRNIAIGGVQEEVYHPNALRAALAEFISTLIFVFAGSGSGIAFNKITDNGATTPSGLVAAALAHAFGLFVAVSVGANISGGHVNPAVTFGVLLGGNI
SlTIP1;1   -MPINQITIGS-HEELRHPGALKAALAEFISTLIFVFAGQGSGMAFNKLTD-GVATPAGLISASIAHAFGLFVAVSVGANISGGHVNPAVTFGAFVGGNI
BpeTIP1;1  -MPFRNIAVGR-PEEARHPDTLRSALAEFISTLIFVFAGEGSGMAFNKLTDNGSTTPSGLVQAALAHAFGLFVAVSISANISGGHVNPAVTFGAFIGGNI
BpeTIP1;2  -MPINQIAVGH-PAEFGDSGALKAALAEFISVLIFVFAGEGSGVAFNKLTDNGSTTPAGLVAAALAHGFGLFVAVSIGANISGGHVNPAVTFGAFLGGHI
BpeTIP1;3  -MPISRIAIGN-PEEASRPDAFRAAFAEFFSMLIFVFAGEGSGMAFNKLTDNGSTTPSGLVAAALAHAFALFVAVSVGANISGGHVNPAVTFGAFIGGNI
BpeTIP3;1  -MP-RRYAFGR-AEDATHPDSMRAALSEFIATFIFVFAGEGSLLAL--IYKDTGSTPSELVAIALAHAFSLFAAVAASLNISGGHVNPAVTFGTLLGGRI
BpeTIP2;1  ---MARIAFGR-FDDSFSVGSFKAYLAEFISTLLFVFAGVGSAIAYNKLTSNAALDPSGLVGIAICHGFALFVAVAVGANISGGHVNPAVTFGLALGGQI
BpeTIP2;2  ---MVKLAFGH-FGESFNVESLKAYLAEFIATLLFVFAGVGSAISYGKLTSDAALDPAGLVAVAVAHAFALFVGVSIAANISGGHLNPAVTFGLALGGNI
BpeTIP4;1  ---MAKIALGT-TREATQPDCIKALVVEFITTFLFVFAGVGSAMAADKLDSGSLV---GLFAVAITHALVVAVMISAG-HISGGHLNPAVTLGLLAGGHI
AtTIP5;1   MRRMIPTSFSSKFQGVLSMNALRCYVSEFISTFFFVLAAVGSVMSSRKLMAGDVSGPFGVLIPAIANALALSSSVYISWNVSGGHVNPAVTFAMAVAGRI
SlTIP5;1   -----MASLASRLQHSVTPNALRSYLAEFLSTFFFVFAAAGASMSTRKMVPDATSDPSSLVAIAVANAFALSVAVYISANISGGHVNPAVTFGMAVGGHI
BpeTIP5;1  ---MGPTMVAARFQHSVTPNALRSYLAEFLSTFFYVFAVVGSAMSSRKLMPDVASDASSLVVVAITNAFALSAAVYVAANISGGHVNPAVTFGMAVGGHI
                                                                          H2                   -NPA;LB-
                                -----------TM1------------          -----------TM2----------

                  110       120       130       140       150       160       170       180       190       200
                    |         |         |         |         |         |         |         |         |         |
AtTIP1;2   TLLRGILYWIAQLLGSVAACFLLSFATGGEPIPAFGLSAGVGSLNALVFEIVMTFGLVYTVYATAVDPKNGSLGTIAPIAIGFIVGANILAGGAFSGASM
SlTIP1;1   TLFRGILYIIAQLLGSTAACALLEFATGGMSTGSFALSAGVSVWNAFVFEIVMTFGLVYTVYATAVDPKKGDLGVIAPIAIGFIVGANILAGGAFTGASM
BpeTIP1;1  TLLRGIVYWIAQLLGSTVACLLLKFATNGLTTSAFSLSSGVGVWNAFVLEIVMTFGLVYTVYATAVDPKRGSVGVIAPIAIGFIVGANILAGGAFDGASM
BpeTIP1;2  SLIRGLLYWVAQLLGSVVACLLLKFSTGGLETPAFSLSSDVSVWNAVVLEIVMTFGLVYTVYATAVDPKKGNIGILAPIAIGLIVGANILAGGAFDGASM
BpeTIP1;3  TLLRTILYWIAQLLGSVVACLLLKFATGGLETSAFSLSSGVSVWNAVVFEIVMTFGLVYTVYATAVDPKKGNVGTVAPIAIGFIVGANILVGGAFDGASM
BpeTIP3;1  SVLRAVFYWIAQLLGAVVASLLLRLVTNNMRPVGFYVTSGVGEWHGLIFEIVMTFTLVYTVFATAIDPKRGSLGTIAPLAIGFIVGANILAGGPFDGACM
BpeTIP2;1  TLLTGIFYWIAQLLGSIAACFLLKAVT-GLAIPTHSVASGVGAIEGVVMEIIITFALVYTVYATAADPKKGSLGTIAPIAIGFIVGANILAAGPFSGGSM
BpeTIP2;2  TILTGIFYWIAQCLGSIVACLLLQFVTNGLSIPTHGVASGMNAVEGVVMEIVITFALVYTVYATAADPKKGSLGIIAPIAIGFIVGANILAAGPFSGGSM
BpeTIP4;1  TIFRSFLYWIDQLLAASAACIILYYITGGLTTPIHSLASGVGYLQGVIWEVILTFSLLFTVYATIVDPKKGALDGLGPTLTGFVVGANILAGGAFSGASM
AtTIP5;1   SVPTAMFYWTSQMIASVMACLVLKVTVMEQHVPIYKIAGEMTGFGASVLEGVLAFVLVYTVFT-ASDPRRGLPLAVGPIFIGFVAGANVLAAGPFSGGSM
SlTIP5;1   SIPMSIFYWISQMIGSVTACLLLKFTN--QQVPTHGIPQEMTGFGAAVLEGVMTFGLVYTVYA-AADPRRCVHAAIGPLAIGLMLGANVMASGPFTGGSM
BpeTIP5;1  SVPTALFYWLSQMLASVMASLLLKMTVVGQHVPTYVIAEEMTGFGASLMEGVLTFALVYTVYA-AGDTRRGTMGAIGPIAIGSIAGANVLAAGPFSGGSM
                                      P1                                                             H5     LE1
              -----------TM3------------         -----------TM4----------       -----------TM5--------
```

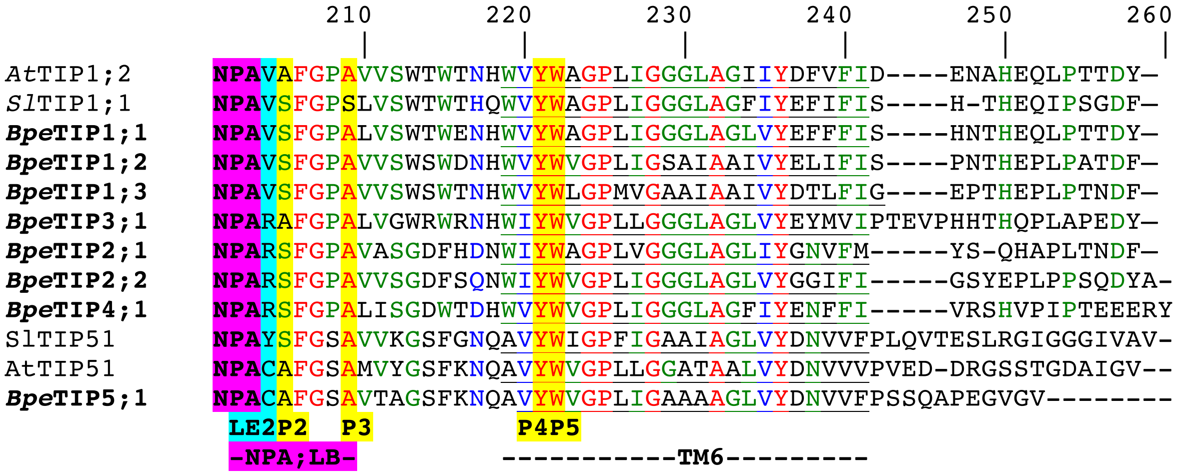

Figure S6B. Amino acid sequence alignment of the *Bpe*TIP subfamily members from *Betula pendula*. *Sl*TIP1;1 (Solyc06g074820.1) and *Sl*TIP5;1 (Solyc03g093230.1) (Reuscher et al., 2013) from Tomato and *At*TIP1;1 (At2g36830) and *At*TIP5;1 (At3g47440) from *Arabidopsis thaliana* were used as references. The highest residue similarities between MIP members are shown in red (100%), and green (>80%). Residues with similar physicochemical behaviors are shown in blue. The six transmembrane domains (TMH1-6) and the two hemi-helicoidal regions that include the «NPA» motives «NPA1» (in Loop B) and «NPA2» (in Loop E) (framed in purple) are underlined. The amino acids residues constituting the ar/R constriction region are shown with blue highlights and labeled H2, H5, LE1 and LE2, and residues constituting the Froger's positions are shown with yellow highlights and labeled P1 to P5. Histidine (H) surligned in red and placed in loop C would be key for the de-protonation of NH_4^+, which would allow the transport of NH_3 independent of pH.

Uncharacterized X intrinsic proteins (XIPs)

The *Bpe*XIP2;1 sequence used for the alignment was the redefined sequence (redefinition detailed in the Figure S6)

```
                        10        20        30        40        50        60        70        80        90       100
                         |         |         |         |         |         |         |         |         |         |
SlXIP1;1       MASNSNVVFGDEESQISGGTNRVQPCSSTPRKINTIDDDEGKKHNFLSLSQRLGVADFFCLDVWRASMGELLGSAVLVFMLDTIVISTFESETKMPNLIM
PoptrXIP2;1    MAGNAGVV-QDEEIGYGG--NKVQPFASTPRPSKT----ERGKRDSSALSRILGLDELVSLNVWRASLAEVFGTAVLVFAMDTIVISSYETQTKTPNLVM
BpeXIP2;1      MAQNVQRV-EDEESLYVG-SRVQP---------------EGNR-NTSKLSEILGLEEFFSLNVWRASLAELLATAIFVFVVDTIVISSFETKTESPKLIM
PoptrXIP1;1    ----------MDVVIHQHHQDDGNQAFSKSLQGCEATDLKEKKSIMIKVLNSVGAHKIFSSEMWRATLTELVATACLLFTLTTSIISCLESTTAEPKFLI
BpeXIP1;1      ----------MDMVV----PGQGDQESPTSFEKCEAQNFRTK------FLVSIGAHEYFSPEMWRGALTELVATAFLMFTLISSIIACLDSQEVDPKLLV
BpeXIP1;2      ----------MDIVV----PGQGDQEFPTSFEKCEAQNFSTK------FLVSVGAHEYFSPEMWRAALTELIATAFLMFTLTSSIIACLDSHEVNPKLLV
BpeXIP1;3      ----------MDIVV----PGQGDQEFPTSFEKCEAQNFRTK------FLVSVGAHEYFSPEMWRGALTELVATAFLMFTLTSSIIACLDSHEVDPKLLV
                                                                              -----------TM1------------         ---

                       110       120       130       140       150       160       170       180       190       200
                         |         |         |         |         |         |         |         |         |         |
SlXIP1;1       SILIAVVITILLLAVVPVSGGHINPVISFSAALVGIISMSRAIIYIVAQCVGAILGALALRAVVSSSIEDTFSLGGCTVTIIAPGPNGPVIVGLETAQAL
PoptrXIP2;1    ATLIAITIAILLLATFPISGGHINPAITLSAMFTGLITVSRAAIYILAQCIGAILGALALKAVVNSTIEQTFSLGGCTLKIVAPGPSGPVAIGLETGQAL
BpeXIP2;1      SLLIAIITAILLLAANPVSGGHINPVITLVAALVGLISVSRAAVYILAQCAGAVLGALALKAVVNNTIEDTFSLGGCTLTVIAPGPNGPISVGLGTSQGL
PoptrXIP1;1    PFAIFVIAFFFLLTTVPLSGGHMSPVFTFIAALEGVITPVRALFYMSAQCVGSIVAYLVIKSVMDKNAEEKYSLGGCMIDGNGEG--------ISPTNAF
BpeXIP1;1      PLAVFIIAFLFLLATIPLSGGHMNPILTFIACLKGLITLARASIYVLAQCLGSIIGFFIIKNVMNHQTGHNYFLGGCTISALNRG------------TAL
BpeXIP1;2      PLAVFIIAFLFLMATAPLSGGHMSPIFTFIACLKGLITLARASIYVLAQCLGSIIGFFIIKNVMNHQIGHKYFLGGCTISALSRG------------TAL
BpeXIP1;3      PLAVFIIAFLFLMATVPLSGGHMSPIFTFIACLKGLITLARASMYVLAQCLGSIIGFFIIKNVMNHQTGHKYFLGGCTISTLSRG------------TAL
                  H2                -NPA;LB-                                  P1
               --------TM2------                                        ---------TM3-----------                           -----
```

```
                    210       220       230       240       250       260       270       280       290       300
                      |         |         |         |         |         |         |         |         |         |
SlXIP1;1     WLEIFCTFVFLFASIWMAYDHRQAKALGHVTVLSIVGLVLGLLVFISTTVTAKKGYGGAGINPARCLGPAIIRGGHLWDGHWIFWVGPTIGCVAFYVYTK
PoptrXIP2;1  WLEIICTFVFLFSSIYIAFDRRQAIALGRVVFCSIIGLVVGLLVFISTTVTATKGYAGVGMNPARCLGPALVRGGHLWKGHWVFWVGPIVASLTFSLYTK
BpeXIP2;1    WLEIICTFVLLFASVWIALDHRQARALGKVIIFSVIGIVVGLLVFVSTTVTGAKGYAGAGMNPARCLGPAIVRGGHLWNGHWVFWAGPSIASVAFYLYTK
PoptrXIP1;1  ILEFSCTFIVLFVGVTVAFDKRRCKELGLQMVCGILAGAMTLAFFVSISVTGRAGYAGAGLNPARCLGPSLLKGGRLWYGHWVFWVGPFVACIVYYGFTL
BpeXIP1;1    VLEFCCTFVLLFVGVTVAFDKKRCKELGLAVVCAVVAAAWALAVFVSITVTGQVGYAGVGLNPARCLGPALLHGGVLWDGHWVFWVGPFLACIVYYGYSM
BpeXIP1;2    VLEFCCTFVVLFVGVTVAFDKKRCKELGLAVVCVVVAGAMALAVFVSITVTGQASYAGVGLNPARCLGPVLLQGGVLWDGHWVLWVGPFLACIVYYGYSM
BpeXIP1;3    VLEFCCTFVVLFVGVTVAFDKKRCKELGVAVVCVVVAGAMALAVFVSITVTGQAGYAGVGLNPARCLGPALLQGGVLWDGHWIFWVGPFLACIVYYGYSM
                                                       H5       LE1   LE2P2 P3             P4P5
             ------TM4---------      -------------TM5------------         -NPA;LE-    ----------TM6------------

                     310       320       330
                       |         |         |
SlXIP1;1     IIPTKHFLAEYGFKHDFVGVVKALSNV-----------
PoptrXIP2;1  IIPREHLLGAESK-------------------------
BpeXIP2;1    IIPSQHFHAQ-GYKHDFLNILKAPFTSDSVHKK-----
PoptrXIP1;1  TLPTGTS-------------------------------
BpeXIP1;1    SLPKEGLVEVEG-EYDISRLVGACFGENGSPTSLEGKF
BpeXIP1;2    NLPKEGLVGVEG-EYDISKLVGACFGENGSPTSLERKF
BpeXIP1;3    NLPKEGLVGVEG-EYDISRLVGACFGENGSPTSPEGKF
```

Figure S6C. Amino acid sequence alignment of the *Bpe*XIP subfamily members from *Betula pendula*. *Sl*XIP1;1 (Solyc10g054840.1; Reuscher et al., 2013) from Tomato and *Poptr*XIP2;1 and *Poptr*XIP1;1 (Potri.009G128500.1, Potri.009G128100, resp.; Lopez et al, 2012) from Poplar were used as references. The highest residue similarities between MIP members are shown in red (100%), and green (>80%). Residues with similar physicochemical behaviors are shown in blue. The six transmembrane domains (TMH1-6) and the two hemi-helicoidal regions that include the «NPA» motives «NPA1» (in Loop B) and «NPA2» (in Loop E) (framed in purple) are underlined. The amino acids residues constituting the ar/R constriction region are shown with blue highlights and labeled H2, H5, LE1 and LE2, and residues constituting the Froger's positions are shown with yellow highlights and labeled P1 to P5.

Nodulin-26 like intrinsic proteins (NIPs)

```
                    10        20        30        40        50        60        70        80        90       100
                     |         |         |         |         |         |         |         |         |         |
AtNIP1;1     ---------------------MADISGNGYGNAREEVVMVNLKDEVEHQQEMEDIHNPRPLKKQDSLLSVSVPFLQKLIAEFLGTYFLVFTGCASVVVNM
SlNIP1;1     ---------------------MADHQINVNGNINHGVSL-NIKEDHDLNNHKESSST----SSF-----LTVPFIQKVIAEMIGTYFLIFAGCGSVVVN-
BpeNIP1;1    ----------------------------------MGIEELEISKVEEGVGTSAATKTNSDTVTAFCSSASVVTITQKLIAEVIGTYFVIFAGCGAVAVN-
BpeNIP4;1    ------------------------------------MSSQDITKVEEGNSSSSNNNGNSGV----CNSSEIVQLVQKVIAEVIGTYFLIFAGCGSVVVN-
BpeNIP1;2    ------------------------MAENSGSNGSQSITL-NVKECEANHNQASSASK----TNHGGGFCVSVPFMQKLVAEVLGTYFLIFAGCGAVVVNL
BpeNIP2;1    -----------------------------MATDPKPKNLAEANEFVSLENPISRKPKP-GLLRALLEEHYPPGFLSKVVAETIATYLLVFVTCGSAALSA
BpeNIP5;1    ---------------------------------------------------MYYFFFGGKSLGFIYLF--LS---AFYLGAEFVGTFILIFMATAGPIVNQ
BpeNIP5;2    -------------------------------------MILGLNIFASMDQCLLIEIIKVLKFIFILNDMH---NSTLGAEFVGTFIMVFSAAAAPIVNE
BpeNIP6;1    MENEEVPSAPSTPATPGTPGAPLFGGFKAERSGNGRRSLLKNCSKCFSVDQEWALEEGTLPKLSCSLPPPPIPLAKKVGAEFIGTLILIFAGTATAMVNQ
AtNIP7;1     -------------------------------MNGEARSRVVDQEAGSTPSTLRDEDHPSRQRLFGCLPYDIDLNPLRIVMAELVGTFILMFSVCGVISSTQ
SlNIP7;1     ---------------------MKLPSYENGLSVEFQVDASASEQSTYDQETTSSNVEMLERRNVCNSILGIDPIFLRMVLAEALGTFLLMFCICGMMASME
BpeNIP7;1    ---------------------MKGQPHHLDHTIDHAPTSGQSKDDQEMGVNAMSKSDVLKNSPFSCFPQGMDLNLGRVVLAELVGTFILMFCVCGIIACTQ
                                                                                              ----------TM1----------

  110       120       130       140       150       160       170       180       190       200
                     |         |         |         |         |         |         |         |         |         |
AtNIP1;1     QNDNVVTLPGIAIVWGLTIMVLIYSLGHISGAHINPAVTIAFASCGRFPLKQVPAYVISQVIGSTLAAATLRLLFGLDHDVCSGKHDVFIGSSPVGSDLQ
SlNIP1;1     ADKGMITFPGVAITWGLVVMVMVYSVGHISGAHFNPSVTIAFASVKRFPWKQVPAYVAAQVLGATLASGTLRLIF-------NGKHDHFAGTLPSGTDFQ
BpeNIP1;1    KIYGSVTFPGICVTWGLIVTVMIYTVGHISGAHFNPAVTITSAIFRRFPYKEVPLYIAAQLMGSILASGTLALVF-------DVTPDAYFGTVPTGSNSQ
BpeNIP4;1    KIYGSVSFPGICVVWGLIVMVMIYSVGHISGAHFNPAVTITFAIFRRFPLKQVPLYIVAQMIGSTLASATLCALF-------PVDKKSFFGTAPVGSNFQ
BpeNIP1;2    GNDKVVTAPGVSIVWGLAVMVLVYSVGHISGAHFNPAVTIAFATCRRFPWKQVPAYVSAQVLGSTMASGTLRLIF-------NGHENRFVGTIPAGSNLQ
BpeNIP2;1    ADENKVSKLAASVAGGLIVTVMIYAVGHISGAHMNPAVTLAFAAVRHFPWKQVPLYAAAQLAGAISAAFTLRVIL--------NPIKELGTTSPTGTEIE
BpeNIP5;1    KYNGAESLIGNAACAGLAVMVVILSTGHISGAHLNPSLTIAFATLRHFPWLQVPAYIAAQVSGSICASFALKGVF--------HPYMSGGVTVPSVTEGQ
BpeNIP5;2    KYQGAETLIGSAACAGLAVMAMVLSTGHISGAHFNPSVTIAFAALRHFPWVQVPGYLVAQVSASICAAFTLKGVF--------HPFMSGGVTVPAVGIGQ
BpeNIP6;1    KTHGSETLLGCAASSGLAVMVVILSTGHISGAHLNPALTIAFAALKHFPWKHVPMYIGAQVVASFCAAFALKGIF--------HPIMGGGVTVPSGSYGQ
AtNIP7;1     LSGGHVGLLEYAVTAGLSVVVVVYSIGHISGAHLNPSITIAFAVFGGFPWSQVPLYITAQTLGATAATLVGVSVY--------GVNADIMATKPALSCVS
SlNIP7;1     IMGVQVGLMEYATTAALTVVVVVFSIGPISGAHINPAVTLAFAAVGHFPWSKVPLYVVAQVGGSILATYTGKLVY--------GLKAEFVTTKPLHSCTS
BpeNIP7;1    LSKGEVGLLEYAATAGLTVVVVIFSIGHISCAHVNPAVTIAFATFGHFPWSKVPLYILAQTLGSVLATCIGQSVY--------DVKSEFMTTRPLRGCSS
                             H2                                                           P1
                        ----------TM2----------      -NPA;LB-              ----------TM3----------
```

```
                     210       220       230       240       250       260       270       280       290       300
                       |         |         |         |         |         |         |         |         |         |
AtNIP1;1     AFTMEFIVTFYLMFIISGVAT-DNRAIGELAGLAIGSTVLLNVLIAAPVSSASMNPGRSLGPALVYGCYKGIWIYLVAPTLGAIAGAWVYNTVRYTDKPL
SlNIP1;1     SFVIEFIITFYLMFVISGVAT-DNRAIGELAGLAVGATILLNVMFTGPISGASMNPARSLGPAIVSSHYKGLWIYLVSPTLGAIAGAWVYNIIRFTDKPL
BpeNIP1;1    SLVLEILCSFLLMFVISGVTT-DSRAISEVAGIAVGMTILLNVFIAGPVSGASMNPARSIRPAVIKHVYKGLWVYIVGPIIGTIAGAFAYNLIRSTDKPL
BpeNIP4;1    ALIIEIIISFLLMFVICGVAT-DNRAIGELAGIAVGMTITLNVFVAGPVSGASMNPARSIGPALLTHMYKGLWIYILGPLAGTILGGFAYNMIRFTDKPL
BpeNIP1;2    SFALEFIITFYLMFVISGVAT-DNRAIGELAGLAVGSTILLNVMFAGPISGASMNPARSLGPAIVHNKYESIWVYLVATTLGAIAGGMVYNIIRFTDKPL
BpeNIP2;1    ALVMEIVVTFTMMFTASAVAT-DTKAIGELAGLAVGSAVCITSILAGPISGGSMNPARTIGPAIASASYKGIWVYLVGPVTGTLLGAWSYKLIRVSDEPL
BpeNIP5;1    AFALEFIITFNLLFVVTAVAT-DTRAVGELAGIAVGATVLVNILVAGPSSGGSMNPVRTLGPAIAAGNYKSLWIYLVAPTLGALTGAGVYTAVKLREDEV
BpeNIP5;2    AFALEFIVTFTLLFVITAVAT-DTCAVGEFAGIAIGGTVMLNILVAGTTTGASMNPVRTIGPAIAAGTYKGFWIYMVAPILGALAGAGTYTFVKLAEDGV
BpeNIP6;1    AFALEFIISFNLMFVVTAVAT-DTRAVGELAGIAVGATVMLNILIAGPTTGASMNPVRTLGPAVAANNYKAIWVYLTAPILGALCGAGIYSAVKLPEEDG
AtNIP7;1     AFFVELIATSIVVFLASALHCGPHQNLGNLTGFVIGTVISLGVLITGPISGGSMNPARSLGPAVVAWDFEDLWIYMTAPVIGAIIGVLTYRSISLKTRPC
SlNIP7;1     AFFVELLATFIVLFLSASLTNYDPQSTGPLSGFLVGVAIGLAVLISGPVSGGSMNPARSLGPAIVAWKFNNLWIYVIAPIIGAVAGVVFYRFLRLQGWS-
BpeNIP7;1    AFWVELIATFIIMFLAASVT-YQHQSVGHLSGFVVGIAISLAV----PVSGGSMNPARSLGPAIISGKFNDIWIYMTAPTIGAVAGALLFRILRLQQRRP
                                                       H5     LE1     LE2P2   P3           P4P5
              --------TM4---------          ----------TM5----------       -NPA;LE-       ----------TM6----------

                      310       320
                        |         |
AtNIP1;1     --REITKS-GSFLKTVRIGST-
SlNIP1;1     --REITKS-GSFLKSKNSST---
BpeNIP1;1    --QHLPRSTGSFLRL--------
BpeNIP4;1    --RELTKS-GSFLKTCPKNGF-
BpeNIP1;2    --REITKS-GSFLKASRNNNSV-
BpeNIP2;1    --YPMSKEGHSKEMVNKYPNNSV
BpeNIP5;1    ---EAPRQVRSFRR---------
BpeNIP5;2    ---EGQDN---------------
BpeNIP6;1    GIHEKPSTTSSFRR---------
AtNIP7;1     PSPVSPSVSSLLR----------
SlNIP7;1     CKPNSTPTTHQHI----------
BpeNIP7;1    CNPTSTEEQEKPKAKSE------
```

Figure S6D. Amino acid sequence alignment of the *Bpe*NIP subfamily members from *Betula pendula*. *Sl*NIP1;1 (Solyc03g0059.2) and *Sl*NIP7;1 (Solyc03g0059.1) (Reuscher et al., 2013) from Tomato and *At*NIP1;1 (AT4G19030) and *At*NIP7;1 (AT3G06100) from *Arabidopsis thaliana* were used as references. The highest residue similarities between MIP members are shown in red (100%), and green (>80%). Residues with similar physicochemical behaviors are shown in blue. The six transmembrane domains (TMH1-6) and the two hemi-helicoidal regions that include the «NPA» motives «NPA1» (in Loop B) and «NPA2» (in Loop E) (framed in purple) are underlined. The amino acids residues constituting the ar/R constriction region are shown with blue highlights and labeled H2, H5, LE1 and LE2, and residues constituting the Froger's positions are shown with yellow highlights and labeled P1 to P5. The putative conserved Calcium-dependent Protein Kinase recognition sites in the C-terminus are highlighted in green. Tyrosine (Y) surligned in red and localized in the TM2 would allow the transport of Urea and Glycine.

Small basic intrinsic proteins (SIPs)

```
                       10        20        30        40        50        60        70        80        90       100
                        |         |         |         |         |         |         |         |         |         |
AtSIP1;1    -MMGVLKSAIGDMLMTFSWVVLSATFGIQTAAIISAGDFQAITWAPLVILTSLIFVYYVSIFTVI---FGSASFNPTGSAAFYVAGVP-GDTLFSLAIRLP
SlSIP1;1    --MGVIKAAIADGLLTFLWVFCSSNIGVSTYFIASYFGIVNEI-PSLFITTLIVFVIFLMFDFLGDVLGGAGFNPTGNAAFYAAGLG-DDSLVSAAVRCP
BpeSIP1;1   --MGAIKAAIGDAVLTFMWVFSASTLGAATTVTARALGIEGLVWPSVFITTVLVFVLVFFFTLIANALGGASFNPTGTASFYAAGLG-PDTLFSMALRFP
BpeSIP1;2   --MGLMRVAIGDAILTSLWVFSAPMMGILTPIIATYLGVQAKSLAGLFITTLLATSLVLIFQLIGKVLGGASFNPSTTVSFYAAGLKHNSSLISMAVQFP
AtSIP2;1    --MGRIGLVVTDLVLSFMWIWAGVLVNI---LVHGVLGFSRTDPSGEIVRYLFSIISMFIFAYLQQATKGGLYNPLTALAAGVSG-GFSSFIFSVFVRIP
SlSIP2;1    MGVSRRSLVISDFIMSFMWVWSSVLIKM---FVHKILGYGAHD------------------------LKGGAYNPLTILSGAISG-DLTNFIFTVAARIP
BpeSIP2;1   --MASAGLLVSDFIISFMWVWSGVLIKI---FVYKVLELGH-EPSGEVIKCALSIVNMFFFAFLGKTTKGGAYNPLTVFAGAISG-DFKRFLFDAGARIP
                 ------------TM1------------           ----------TM2----------  -NPA;LB-    ------TM3-----

110       120       130       140       150       160       170       180       190       200
                        |         |         |         |         |         |         |         |         |         |
AtSIP1;1    AQAIGAAGGALAIMEFIPEKYKHMIGGPSLQVDVHTGAIAETILSFGITFAVLLIILRGPRRLLAKTFLLALATISFVVAGSKYTGPAMNPAIAFGWAYM
SlSIP1;1    AQVAGAVAGSLALVELIPKHYHHMLDGPALKVDVQTGAIAEGVLTFVITFMIFVIVLRGPESVLLKNWLLTMVTLPLVLAGSNFTGPSMNPANAFGWAYL
BpeSIP1;1   AQAAGAVGGALAIMEVMPKNYKHMLGGPSLKVDLHIGAIAEGVLTFLISFAVLVIVLRGPRSPILKTWLLAMATVALVIAGSNYTGPSMNPANAFGWAYV
BpeSIP1;2   AQAAGGLVGAKAILQVLPSQYKQRLRGPSLKVDLLTGVIAEAALTLGLSFAILLIMLRGPKNPLVKVWLVAVATVGLVVAGSGYTGPSMNPANAFGWAFV
AtSIP2;1    VEVIGSILAVKHIIHVFPEIGK----GPKLNVAIHHGALTEGILTFFIVLLSMGLTRKIPGSFFMKTWIGSLAKLTLHILGSDLTGGCMNPAAVMGWAYA
SlSIP2;1    SQVFGSITGVRFIIAAFPNIGR----GPVLSIDIHRGALTEGILTFAIVSISLGLSRRSRASTFMKTWISSLSKLTLHILGSDLTGGCMNPASVMGWAYA
BpeSIP2;1   AQVIGSIIGVKLIIETFPEIGL----GPRLNVDIHRGALTEGFLTFAIVIISLGLARKIPGSFVMKTWISSVSKLTLHILGSDLTGGCMNPASVMGWAYA
                                                                                 H5          LE1  LE2P2 P3
            ---TM3---                        ---------TM4---------        ----------TM5--------  -NPA;LE-

210       220       230       240       250
                        |         |         |         |         |
AtSIP1;1    YSSHNTWDHIYVYWISSFVGALSAALLFRSIFPPPRPQK-KKQKKA-----
SlSIP1;1    SNTHKTLEHFYVYWISPFIGAILAAWIFRVLFPPPVEQKPQKQKRN-----
BpeSIP1;1   NNRHNTWEQFYVYWICPFVGAILAAWVFRALFPPT----PAKQKKA-----
BpeSIP1;2   NNQHNTWEHFYVYWIGSFTGATVAGWIYRFLFP------------------
AtSIP2;1    RGEHITKEHLLVYWLGPVKATLLAVWFFKVVFKPLTEEQEKPKAKSE----
SlSIP2;1    RGDHITKEHIHVYWLAPIQATLLAVWTFNLLVSPSKDKEAKKTEKKSE---
BpeSIP2;1   RGDHITKEHILVYWLAPIEATLLAVWIFRLVVRPIKEEKANVKKNESLRIC
                          P4P5
                         ----------TM4----------
```

Figure S6E. Amino acid sequence alignment of the *Bpe*NIP subfamily members from *Betula pendula*. *Sl*SIP1;1 (Solyc12g0196.1), *Sl*SIP1;2 (Solyc10g0784.1) and *Sl*SIP2;1 (Solyc01g0567.1) (Reuscher et al., 2013) from Tomato, and *At*SIP1;1 (AT3G04090), *At*SIP1;2 (AT5G18290) and *At*SIP2;1 (AT3G56950) from *Arabidopsis thaliana* were used as references. The highest residue similarities between MIP members are shown in red (100%), and green (>80%). Residues with similar physicochemical behaviors are shown in blue. The six transmembrane domains (TMH1-6) and the two hemi- helicoidal regions that include the «NPA» motives «NPA1» (in Loop B) and «NPA2» (in Loop E) (framed in purple) are underlined. The amino acids residues constituting the ar/R constriction region are shown with blue highlights and labeled H2, H5, LE1 and LE2, and residues constituting the Froger's positions are shown with yellow highlights and labeled P1 to P5.
