# Supplementary material for: Genome-Wide Identification, Structure Characterization, and Expression Pattern Profiling of the Aquaporin Gene Family in Betula pendula
Source: Int J Mol Sci. 2021 Jul 6;22(14):7269. doi: 10.3390/ijms22147269 (PMC8304918; doi:10.3390/ijms22147269)
Supplement: Supplementary file 1 [file ijms-22-07269-s001.zip › Supplementary Files Betula MIPs/FigureS7.pdf]

Figure S7. Specificity determining positions (SDP) analysis of the *Bpe*AQPs transporting non-aqua substrates from *Betula pendula*. SDP were predicted from alignments with putative amino acid sequences of the AQPs predicted to transport non-aqua substrates (marked in red). Multiple alignments were performed using ClustalX. The SDPs are highlighted in yellow. The Genbank accession numbers of the representative sequences used for alignments: *At*PIP1;2 (Q06611), *At*PIP2;1 (P43286), *At*PIP2;4 (Q9FF53), *At*TIP1;1 (P25818), *At*TIP1;2 (Q41963), *At*TIP2;1 (Q41951), *At*TIP2;3 (Q9FGL2), *At*TIP4;1 (O82316), *At*NIP1;2 (Q8LFP7), *Cp*NIP1 (CAD67694), *Gm*NOD26 (P08995), *Hv*PIP1;3 (BAA23745), *Hv*PIP1;4 (BAF33068), *Hv*PIP2;1 (BAA23744), *Nt*AQP1 (O24662), *Nt*TIPa (Q9XG70), *Os*NIP2;1 (Q6Z2T3), *Ta*TIP2;1 (AAS19468), *Ta*TIP2;2 (AAS19469), *Zm*PIP1;1 (Q41870), *Zm*PIP1;5 (Q9AR14).

*The *Bpe*XIP2;1 sequence used for the analyses was the redefined sequence (redefinition detailed in the Figure S4)

Ammonia transporters

TIP

```
                    110       120       130       140       150       160       170       180       190       200
                      |         |         |         |         |         |         |         |         |         |
AtTIP2;1     VNPAVTFGLAVGGQITVITGVFYWIAQLLGSTAACFLLKYVTGGLAVPTHSVAAGLGSIEGVVMEIIITFALVYTVYATAADPKKGSLGTIAPLAIGLIV
AtTIP2;3     LNPAVTLGLAIGGNITLITGFFYWIAQCLGSIVACLLLVFVTNGKSVPTHGVSAGLGAVEGVVMEIVVTFALVYTVYATAADPKKGSLGTIAPIAIGFIV
TaTIP2;1     VNPAVTFGLALGGQITILTGIFYWVAQLLGAIVGAFLVQFCTG-VATPTHGL-SGVGAFEGVVMEIIVTFGLVYTVYATAADPKKGSLGTIAPIAIGFIV
TaTIP2;2     VNPAVTFGLALGGQITILTGIFYWVAQLLGAIVGAFLVQFCTG-VATPTHGL-SGVGAFEGVVMEIIVTFGLVYTVYATAADPKKGSLGTIAPIAIGFIV
GmNOD2;6     FNPAVTIAFASTRRFPLIQVPAYVVAQLLGSILASGTLRLLFMGNHDQFSGTVPNGTNLQAFVFEFIMTFFLMFVICGVATDNR--AVGEFAGIAIGSTL
BpeTIP1;1    VNPAVTFGAFIGGNITLLRGIVYWIAQLLGSTVACLLLKFATNGLTTSAFSLSSGVGVWNAFVLEIVMTFGLVYTVYATAVDPKRGSVGVIAPIAIGFIV
BpeTIP1;2    VNPAVTFGAFLGGHISLIRGLLYWVAQLLGSVVACLLLKFSTGGLETPAFSLSSDVSVWNAVVLEIVMTFGLVYTVYATAVDPKKGNIGILAPIAIGLIV
BpeTIP1;3    VNPAVTFGAFIGGNITLLRTILYWIAQLLGSVVACLLLKFATGGLETSAFSLSSGVSVWNAVVFEIVMTFGLVYTVYATAVDPKKGNVGTVAPIAIGFIV
BpeTIP2;1    VNPAVTFGLALGGQITLLTGIFYWIAQLLGSIAACFLLKAVTG-LAIPTHSVASGVGAIEGVVMEIIITFALVYTVYATAADPKKGSLGTIAPIAIGFIV
BpeTIP2;2    LNPAVTFGLALGGNITILTGIFYWIAQCLGSIVACLLLQFVTNGLSIPTHGVASGMNAVEGVVMEIVITFALVYTVYATAADPKKGSLGIIAPIAIGFIV
BpeTIP3;1    VNPAVTFGTLLGGRISVLRAVFYWIAQLLGAVVASLLLRLVTNNMRPVGFYVTSGVGEWHGLIFEIVMTFTLVYTVFATAIDPKRGSLGTIAPLAIGFIV
BpeTIP4;1    LNPAVTLGLLAGGHITIFRSFLYWIDQLLAASAACIILYYITGGLTTPIHSLASGVGYLQGVIWEVILTFSLLFTVYATIVDPKKGALDGLGPTLTGFVV
BpeTIP5;1    VNPAVTFGMAVGGHISVPTALFYWLSQMLASVMASLLLKMTVVGQHVPTYVIAEEMTGFGASLMEGVLTFALVYTVYA-AGDTRRGTMGAIGPIAIGSIA

                    210       220       230       240       250       260       270
                      |         |         |         |         |         |         |
AtTIP2;1     GANILAAGPFSGGSMNPARSFGPAVAAGDFSGHWVYWVGPLIGGGLAGLIYGNVFMG----SSEHVPLASADF-----
AtTIP2;3     GANILAAGPFSGGSMNPARSFGPAVVSGDLSQIWIYWVGPLVGGALAGLIYGDVFIG----SYEAVETREIRV-----
TaTIP2;1     GANILVAGPFSGGSMNPARSFGPAVASGDFTNIWVYWAGPLIGGGLAGVVYRYLYMC----D-DHSSVAGNDY------
TaTIP2;2     GANILVAGPFSGGSMNPARSFGPAVASGDFTNIWIYWAGPLIGGGLAGVVYRYVYMC----D-DHSSVAGNDY------
GmNOD2;6     LLNVIIGGPVTGASMNPARSLGPAFVHGEYEGIWIYLLAPVVGAIAGAWVYNIVRYT----DKPLSETTKSASFLKGRAASK
BpeTIP1;1    GANILAGGAFDGASMNPAVSFGPALVSWTWENHWVYWAGPLIGGGLAGLVYEFFFI-----SHNTHEQLPTTDY-----
BpeTIP1;2    GANILAGGAFDGASMNPAVSFGPAVVSWSWDNHWVYWVGPLIGSAIAAIVYELIFI-----SPNTHEPLPATDF-----
BpeTIP1;3    GANILVGGAFDGASMNPAVSFGPAVVSWSWTNHWVYWLGPMVGAAIAAIVYDTLFIG----EPTHEPLPTNDF-----
BpeTIP2;1    GANILAAGPFSGGSMNPARSFGPAVASGDFHDNWIYWAGPLVGGGLAGLIYGNVFMY-----SQHAPLTN-DF-----
BpeTIP2;2    GANILAAGPFSGGSMNPARSFGPAVVSGDFSQNWIYWVGPLIGGGLAGLVYGGIFIG----SYEPLPPSQDYA-----
BpeTIP3;1    GANILAGGPFDGACMNPARAFGPALVGWRWRNHWIYWVGPLLGGGLAGLVYEYMVIPTEVPHHTHQPLAPEDY-----
BpeTIP4;1    GANILAGGAFSGASMNPARSFGPALISGDWTDHWVYWVGPLIGGGLAGFIYENFFIVR---SHVPIPTEEERY-----
BpeTIP5;1    GANVLAAGPFSGGSMNPACAFGSAVTAGSFKNQAVYWVGPLIGAAAAGLVYDNVVFPS---SQAPEGVGV--------
```

NIP

```
                     110       120       130       140       150       160       170       180       190       200
                       |         |         |         |         |         |         |         |         |         |
AtTIP2;1    KLTSDAALDTPGLVAIAVCHGFALFVAVAIGANISGGHVNPAVTFGLAVGGQITVITGVFYWIAQLLGSTAACFLLKYVTGGLAVPTHSVAAGLGSIEGV
AtTIP2;3    KLTSDGALDPAGLVAIAIAHAFALFVGVSIAANISGGHLNPAVTLGLAIGGNITLITGFFYWIAQCLGSIVACLLLVFVTNGKSVPTHGVSAGLGAVEGV
TaTIP2;1    KVSGGAPLDPSGLIAVAICHGFGLFVAVAIGANISGGHVNPAVTFGLALGGQITILTGIFYWVAQLLGAIVGAFLVQFCT-GVATPTHGLS-GVGAFEGV
TaTIP2;2    KVSGGAPLDPSGLIAVAICHGFGLFVAVAIGANISGGHVNPAVTFGLALGGQITILTGIFYWVAQLLGAIVGAFLVQFCT-GVATPTHGLS-GVGAFEGV
GmNOD2;6    NYYN-----MITFPGIAIVWGLVLTVLVYTVGHISGGHFNPAVTIAFASTRRFPLIQVPAYVVAQLLGSILASGTLRLLFMGNHDQFSGTVPNGTNLQAF
BpeNIP1;1   IYGS------VTFPGICVTWGLIVTVMIYTVGHISGAHFNPAVTITSAIFRRFPYKEVPLYIAAQLMGSILASGTLALVFDVTPDAYFGTVPTGSNSQSL
BpeNIP4;1   IYGS------VSFPGICVVWGLIVMVMIYSVGHISGAHFNPAVTITFAIFRRFPLKQVPLYIVAQMIGSTLASATLCALFPVDKKSFFGTAPVGSNFQAL
BpeNIP1;2   GNDK-----VVTAPGVSIVWGLAVMVLVYSVGHISGAHFNPAVTIAFATCRRFPWKQVPAYVSAQVLGSTMASGTLRLIFNGHENRFVGTIPAGSNLQSF
BpeNIP2;1   ADEN-----KVSKLAASVAGGLIVTVMIYAVGHISGAHMNPAVTLAFAAVRHFPWKQVPLYAAAQLAGAISAAFTLRVILNPIKE-LGTTSPTGTEIEAL
BpeNIP5;1   KYNG-----AESLIGNAACAGLAVMVVILSTGHISGAHLNPSLTIAFATLRHFPWLQVPAYIAAQVSGSICASFALKGVFHPYMSG-GVTVPSVTEGQAF
BpeNIP5;2   KYQG-----AETLIGSAACAGLAVMAMVLSTGHISGAHFNPSVTIAFAALRHFPWVQVPGYLVAQVSASICAAFTLKGVFHPFMSG-GVTVPAVGIGQAF
BpeNIP6;1   KTHG-----SETLLGCAASSGLAVMVVILSTGHISGAHLNPALTIAFAALKHFPWKHVPMYIGAQVVASFCAAFALKGIFHPIMGG-GVTVPSGSYGQAF
BpeNIP7;1   LSKG-----EVGLLEYAATAGLTVVVVIFSIGHISCAHVNPAVTIAFATFGHFPWSKVPLYILAQTLGSVLATCIGQSVYD-VKSEFMTTRPLRGCSSAF

                     210       220       230       240       250       260       270       280       290       300
                       |         |         |         |         |         |         |         |         |         |
AtTIP2;1    VMEIIITFALVYTVYATAADPKKGSLGTIAPLAIGLIVGANILAAGPFSGGSMNPARSFGPAVAAGDFSGHWVYWVGPLIGGGLAGLIYGNVFMGSSEHV
AtTIP2;3    VMEIVVTFALVYTVYATAADPKKGSLGTIAPIAIGFIVGANILAAGPFSGGSMNPARSFGPAVVSGDLSQIWIYWVGPLVGGALAGLIYGDVFIGSYEAV
TaTIP2;1    VMEIIVTFGLVYTVYATAADPKKGSLGTIAPIAIGFIVGANILVAGPFSGGSMNPARSFGPAVASGDFTNIWVYWAGPLIGGGLAGVVYRYLYM-CDDHS
TaTIP2;2    VMEIIVTFGLVYTVYATAADPKKGSLGTIAPIAIGFIVGANILVAGPFSGGSMNPARSFGPAVASGDFTNIWIYWAGPLIGGGLAGVVYRYVYM-CDDHS
GmNOD2;6    VFEFIMTFFLMFVICGVATDNR--AVGEFAGIAIGSTLLLNVIIGGPVTGASMNPARSLGPAFVHGEYEGIWIYLLAPVVGAIAGAWVYNIVRYTDKPLS
BpeNIP1;1   VLEILCSFLLMFVISGVTTDSR--AISEVAGIAVGMTILLNVFIAGPVSGASMNPARSIRPAVIKHVYKGLWVYIVGPIIGTIAGAFAYNLIRSTDKPLQ
BpeNIP4;1   IIEIIISFLLMFVICGVATDNR--AIGELAGIAVGMTITLNVFVAGPVSGASMNPARSIGPALLTHMYKGLWIYILGPLAGTILGGFAYNMIRFTDKPLR
BpeNIP1;2   ALEFIITFYLMFVISGVATDNR--AIGELAGLAVGSTILLNVMFAGPISGASMNPARSLGPAIVHNKYESIWVYLVATTLGAIAGGMVYNIIRFTDKPLR
BpeNIP2;1   VMEIVVTFTMMFTASAVATDTK--AIGELAGLAVGSAVCITSILAGPISGGSMNPARTIGPAIASASYKGIWVYLVGPVTGTLLGAWSYKLIRVSDEPLY
BpeNIP5;1   ALEFIITFNLLFVVTAVATDTR--AVGELAGIAVGATVLVNILVAGPSSGGSMNPVRTLGPAIAAGNYKSLWIYLVAPTLGALTGAGVYTAVKLREDEV-
BpeNIP5;2   ALEFIVTFTLLFVITAVATDTC--AVGEFAGIAIGGTVMLNILVAGTTTGASMNPVRTIGPAIAAGTYKGFWIYMVAPILGALAGAGTYTFVKLAEDGV-
BpeNIP6;1   ALEFIISFNLMFVVTAVATDTR--AVGELAGIAVGATVMLNILIAGPTTGASMNPVRTLGPAVAANNYKAIWVYLTAPILGALCGAGIYSAVKLPEEDGG
BpeNIP7;1   WVELIATFIIMFLAASVTYQHQ--SVGHLSGFVVGIAISLAV----PVSGGSMNPARSLGPAIISGKFNDIWIYMTAPTIGAVAGALLFRILRLQQRRPC
```

```
                   310       320
                    |         |
GmNOD2;6   ETTKS-ASF-LKGRAASK–
AtTIP2;1   PLASADF-------------
AtTIP2;3   ETREIRV-------------
TaTIP2;1   SVAGNDY-------------
TaTIP2;2   SVAGNDY-------------
BpeNIP1;1  HLPRSTGSF-LRL-------
BpeNIP4;1  ELTKS-GSF-LKTCPKNGF-
BpeNIP1;2  EITKS-GSF-LKASRNNNSV
BpeNIP2;1  PMSKEGHSKEMVNKYPNNSV
BpeNIP5;1  --EAPRQVRSFRR-------
BpeNIP5;2  --EGQDN-------------
BpeNIP6;1  IHEKPSTTSSFRR-------
BpeNIP7;1  NPTS----------------
```

Boric Acid Transporters

PIP1

```
                          10        20        30        40        50        60        70        80        90       100
                           |         |         |         |         |         |         |         |         |         |
HvPIP1;3          MEGKEEDVRLGANRYSEHQPIGTAAQGGGADEKDYKEPPPAPFFEAGELTSWSFYRAGIAEFLATFLFLYISVLTVMGVVGNPSGSKCGTVGIQGIAWSF
HvPIP1;4          MEGKEEDVRLGANRYSERQPIGTAAQGGGADEKDYKEPPPAPLFEAEELSSWSFYRAGIAEFLATFLFLYISVLTVMGVVGNPSGSKCGTVGIQGIAWSF
ZmPIP1;1          MEGKEEDVRLGANKFSERHAIGTAAQGTD--DKDYKEPPPAPLFEPGELKSWSFYRPGIAEFVATFLFLYISILTVMGVSKSTS--KCATVGIQGIAWSF
OsNIP2;1          MASNNSRTNSRANYSNEIHDLST-VQNGTMPTMYYGEKAIADFFPPHLL------KKVVSEVVATFLLVFMTC-GAAGISGSD----LSRISQLGQSIAG
BpePIP1;1         MEGKDEDVRLGANRYRERQPIGTAAQSQD-T-KDYQEPPAAPFFEPGELSSWSFYRAGIAEFVATFLFLYITVLTVMGVSKSPS--KCSTVGIQGIAWAF
BpePIP1;2         MEGKEEDVRLGANKYTERQPLGTAAQTD----KDYKEPPPAPLFEPGELCSWSFYRAGIAEFVATFLFLYITVLTVMGVKRSSS--VCASVGIQGIAWAF
BpePIP1;3         MEGKEEDVRLGANKFPERQPIGTAAQSQD-EGKDYIEPPPAPLFEPSELTSWSFYRAGIAEFVATFLFLYITVLTVMGVAKSPS--KCSTVGIQGIAWAF
BpePIP1;4         MEGKEEDVKVGASKFPERQAIGTSAQTD----KDYKEPPPAPLFEPGELQSWSFWRAGIAEFIATFLFLYITLLTVMGVSRAPS--KCASVGVQGIAWAF

                         110       120       130       140       150       160       170       180       190       200
                           |         |         |         |         |         |         |         |         |         |
HvPIP1;3          GGMIFVLVYCTAGISGGHINPAVTFGLFLARKLSLTRAVFYIVMQCLGAICGAGVVKGFQTTLYQGNGGGANSVAAGYTKGDGLGAEIVGTFVLVYTVFS
HvPIP1;4          GGMIFVLVYCTAGISGGHINPAVTFGLFLARKLSLTRAVFYIVMQCLGAICGAGVVKGFQTTLYQGNGGGANSVAAGYTKGDGLGAEIVGTFVLVYTVFS
ZmPIP1;1          GGMILALVYCTAGISG-HINPAVTFGLFLARKLSLTRAVFYIIMQCLGAICGRGVVKGFQQGLYMGNGGRRNVVAPGYTKGDGLGAEIVGTFILVYTVFS
OsNIP2;1          GLIVTVMIYAVGHISGAHMNPAVTLAFAVFRHFPWIQVPFYWAAQFTGAICASFVLKAVIHPVDVI--GTTTPVGPHW---HSLVVEVIVTFNMMFVTLA
BpePIP1;1         GGMIFALVYCTAGISGGHINPAVTFGLFLARKLSLTRAVFYIVMQCLGAICGAGVVKGFQKNQYERLGGGANTISSGYSKGDGLGAEIVGTFVLVYTVFS
BpePIP1;2         GGMIFALVYCTAGISGGHINPAVTFGLLLARKLSLTRAVFYIIMQCLGAICGAGVVKGFGKTPFEINGGGANVVNHGYTKGDGLGAEIVGTFVLVYTVFS
BpePIP1;3         GGMIFALVYCTAGISGGHINPAVTFGLFLARKLSLTRALFYIVMQCLGAICGAGVVKGFQKSQYEILGGGANFVNPGYTKGDGLGAEIVGTFVLVYTVFS B
BpePIP1;4         GGMIFALVYCTAGISGGHINPAVTFGLFLARKLSLTRALFYIVMQCLGAICGAGVVKGFQKTQYERVGGGANVVNHGYSKGDGLGAEIVGTFVLVYTVFS

                         210       220       230       240       250       260       270       280       290       300
                           |         |         |         |         |         |         |         |         |         |
HvPIP1;3          ATDAKRSARDSHVPILAPLPIGFAVFLVHLATIPITGTGINPARSLGAAIIYNKKQAWDDHWIFWVGPFIGAALAA-IYHVVVIRAIPFKSRGIGAALA//
HvPIP1;4          ATDAKRSARDSHVPILAPLPIGFAVFLVHLATIPITGTGINPARSLGAAIIYNKKQAWDDHWIFWVGPFIGAALAA-IYHVVVIRAIPFKSRDIGAALA//
ZmPIP1;1          ATDAKRRARDSHVPILAPLPIGFAVFLVHLATMGITGTGINPARSLGAAVIYNQHHAWADHWIFWVGPFIGAALAA-IYHQVIIRAIPFKSRS-------
OsNIP2;1          VATDTRAVGE-----LAGLAVGSAVCITSIFAGAISGGSMNPARTLGPALASNK---FDGLWIYFLGPVMGTLSGAWTYTFIRFEDTPKEGSSQKLSSF//
BpePIP1;1         ATDAKRNARDSHVPILAPLPIGFAVFLVHLATIPITGTGINPARSLGAALIFNKEQAWHNHWIFWVGPFIGAALAA-LYHQIVIRAIPFKSK--------
BpePIP1;2         ATDAKRNARDSHVPILAPLPIGFAVFLVHLATIPITGTGINPARSLGAAIIYNKDHAWDDHWIFWVGPFIGAALAA-VYHQIVIRAIPFKARG-------
BpePIP1;3         ATDAKRSARDSHVPILAPLPIGFAVFLVHLATIPITGTGINPARSLGAAIIYNRDRAWDDHWIFWVGPFIGAALAA-LYHQVVIRAIPFKSK--------
BpePIP1;4         ATDAKRNARDSHVPLLAPLPIGFAVFLVHLATIPITGTGINPARSLGAAIIYNKDHAWDDQWIFWVGPFIGAALAA-LYHQIVIRAMPFKTRA-------
```

NIP

```
                  110       120       130       140       150       160       170       180       190       200
                    |         |         |         |         |         |         |         |         |         |
HvPIP1;3    SGSKCGTVGIQGIAWSFGGMIFVLVYCTAGISGGHINPAVTFGLFLARKLSLTRAVFYIVMQCLGAICGAGVVKGFQTTLYQGNGGGANSVAAGYTKGDG
HvPIP1;4    SGSKCGTVGIQGIAWSFGGMIFVLVYCTAGISGGHINPAVTFGLFLARKLSLTRAVFYIVMQCLGAICGAGVVKGFQTTLYQGNGGGANSVAAGYTKGDG
ZmPIP1;1    STSKCATVGIQGIAWSFGGMILALVYCTAGISG-HINPAVTFGLFLARKLSLTRAVFYIIMQCLGAICGRGVVKGFQQGLYMGNGGRRNVVAPGYTKGDG
OsNIP2;1    GSDL-SRISQLGQSIAGGLIVTVMIYAVGHISGAHMNPAVTLAFAVFRHFPWIQVPFYWAAQFTGAICASFVLKA----VIH-PVDVIGTTTPVGPHWHS
BpeNIP1;1   KIYG-SV-TFPGICVTWGLIVTVMIYTVGHISGAHFNPAVTITSAIFRRFPYKEVPLYIAAQLMGSILASGTLAL----VFDVTPDAYFGTVPTGSNSQS
BpeNIP1;2   LGND-KVVTAPGVSIVWGLAVMVLVYSVGHISGAHFNPAVTIAFATCRRFPWKQVPAYVSAQVLGSTMASGTLRL----IFNGHENRFVGTIPAGSNLQS
BpeNIP2;1   AADE-NKVSKLAASVAGGLIVTVMIYAVGHISGAHMNPAVTLAFAAVRHFPWKQVPLYAAAQLAGAISAAFTLRV----ILN-PIKELGTTSPTGTEIEA
BpeNIP4;1   KIYG-SV-SFPGICVVWGLIVMVMIYSVGHISGAHFNPAVTITFAIFRRFPLKQVPLYIVAQMIGSTLASATLCA----LFPVDKKSFFGTAPVGSNFQA
BpeNIP5;1   QKYN-GAESLIGNAACAGLAVMVVILSTGHISGAHLNPSLTIAFATLRHFPWLQVPAYIAAQVSGSICASFALKG----VFH-PYMSGGVTVPSVTEGQA
BpeNIP5;2   EKYQ-GAETLIGSAACAGLAVMAMVLSTGHISGAHFNPSVTIAFAALRHFPWVQVPGYLVAQVSASICAAFTLKG----VFH-PFMSGGVTVPAVGIGQA
BpeNIP6;1   QKTH-GSETLLGCAASSGLAVMVVILSTGHISGAHLNPALTIAFAALKHFPWKHVPMYIGAQVVASFCAAFALKG----IFH-PIMGGGVTVPSGSYGQA
BpeNIP7;1   QLSK-GEVGLLEYAATAGLTVVVVIFSIGHISCAHVNPAVTIAFATFGHFPWSKVPLYILAQTLGSVLATCIGQS----VYD-VKSEFMTTRPLRGCSSA

                 210       220       230       240       250       260       270       280       290       300
                    |         |         |         |         |         |         |         |         |         |
HvPIP1;3    LGAEIVGTFVLVYTVFSATDAKRSARDSHVPILAPLPIGFAVFLVHLATIPITGTGINPARSLGAAIIYNKKQAWDDHWIFWVGPFIGAALAA-IYHVVV
HvPIP1;4    LGAEIVGTFVLVYTVFSATDAKRSARDSHVPILAPLPIGFAVFLVHLATIPITGTGINPARSLGAAIIYNKKQAWDDHWIFWVGPFIGAALAA-IYHVVV
ZmPIP1;1    LGAEIVGTFILVYTVFSATDAKRRARDSHVPILAPLPIGFAVFLVHLATMGITGTGINPARSLGAAVIYNQHHAWADHWIFWVGPFIGAALAA-IYHQVI
OsNIP2;1    LVVEVIVTFNMMFVTLAVATDTRAVGE-----LAGLAVGSAVCITSIFAGAISGGSMNPARTLGPALASNK---FDGLWIYFLGPVMGTLSGAWTYTFIR
BpeNIP1;1   LVLEILCSFLLMFVISGVTTDSRAISE-----VAGIAVGMTILLNVFIAGPVSGASMNPARSIRPAVIKHV---YKGLWVYIVGPIIGTIAGAFAYNLIR
BpeNIP1;2   FALEFIITFYLMFVISGVATDNRAIGE-----LAGLAVGSTILLNVMFAGPISGASMNPARSLGPAIVHNK---YESIWVYLVATTLGAIAGGMVYNIIR
BpeNIP2;1   LVMEIVVTFTMMFTASAVATDTKAIGE-----LAGLAVGSAVCITSILAGPISGGSMNPARTIGPAIASAS---YKGIWVYLVGPVTGTLLGAWSYKLIR
BpeNIP4;1   LIIEIIISFLLMFVICGVATDNRAIGE-----LAGIAVGMTITLNVFVAGPVSGASMNPARSIGPALLTHM---YKGLWIYILGPLAGTILGGFAYNMIR
BpeNIP5;1   FALEFIITFNLLFVVTAVATDTRAVGE-----LAGIAVGATVLVNILVAGPSSGGSMNPVRTLGPAIAAGN---YKSLWIYLVAPTLGALTGAGVYTAVK
BpeNIP5;2   FALEFIVTFTLLFVITAVATDTCAVGE-----FAGIAIGGTVMLNILVAGTTTGASMNPVRTIGPAIAAGT---YKGFWIYMVAPILGALAGAGTYTFVK
BpeNIP6;1   FALEFIISFNLMFVVTAVATDTRAVGE-----LAGIAVGATVMLNILIAGPTTGASMNPVRTLGPAVAANN---YKAIWVYLTAPILGALCGAGIYSAVK
BpeNIP7;1   FWVELIATFIIMFLAASVTYQHQSVGH-----LSGFVVGIAISL----AVPVSGGSMNPARSLGPAIISGK---FNDIWIYMTAPTIGAVAGALLFRILR
```

CO_2 Transporters

PIP1

```
                        10        20        30        40        50        60        70        80        90       100
                         |         |         |         |         |         |         |         |         |         |
AtPIP12      -MEGKEEDVRVGANKFPERQPIGTSAQSD---KDYKEPPPAPLFEPGELASWSFWRAGIAEFIATFLFLYITVLTVMGVKRSPNM--------CASVGIQ
NtAQP1       MAENKEEDVKLGANKFRETQPLGTAAQTD---KDYKEPPPAPLFEPGELSSWSFYRAGIAEFMATFLFLYITILTVMGLKRSDSL--------CSSVGIQ
BpePIP11     -MEGKDEDVRLGANRYRERQPIGTAAQSQDT-KDYQEPPAAPFFEPGELSSWSFYRAGIAEFVATFLFLYITVLTVMGVSKSPSK--------CSTVGIQ
BpePIP13     -MEGKEEDVRLGANKFPERQPIGTAAQSQDEGKDYIEPPPAPLFEPSELTSWSFYRAGIAEFVATFLFLYITVLTVMGVAKSPSK--------CSTVGIQ
BpePIP12     -MEGKEEDVRLGANKYTERQPLGTAAQTD---KDYKEPPPAPLFEPGELCSWSFYRAGIAEFVATFLFLYITVLTVMGVKRSSSV--------CASVGIQ
BpePIP14     -MEGKEEDVKVGASKFPERQAIGTSAQTD---KDYKEPPPAPLFEPGELQSWSFWRAGIAEFIATFLFLYITLLTVMGVSRAPSK--------CASVGVQ
HvPIP2_1     ----------------MAKDIEAAPQGGEFSSKDYSDPPPAPIVDFEELTKWSLYRAVIAEFVATLLFLYITVATVIGYKHQSDPTVNTTDAACSGVGTL
BpePIP21     ----------------MSKEVSEEGQSHQ-HGKDYVDPPPAPLLDLAELKLWSFYRSLIAEFIATLLFLYVTVATVIGHKKATGP--------CDGVGLL
BpePIP22     ----------------MSKEVTEEREASQ-PVKDYVDPPATPLFDVAEFTMWSFYRAVIAEFIAALLFLYITVATVIGYKKQTDP--------CETVGLL
BpePIP23     ----------------MSKEVTEEAEASQ-PVRDYVDPPPAPLFDVEELTEWSFYRAVIAEFISTLLFLYITVATVIGYKKQNDQ--------CETVGLL
BpePIP24     ----------------MAKDAEVPEH-GSFTAKDYHDPPPAPLFDAVELTKWSFYRALIAEFIATLLFLYITVLTVIGYKSQIDPANDGEA--CGGVGVL
BpePIP25     ----------------MAKDVEVAEHQGEYSAKDYHDPPPAPLIDYDELIKWSFYRALIAEFIATLLFLYITVLTVIGYKSQTDPSKNPDQ--CGGVGIL
BpePIP26     ----------------MAKDMEVGGQSG-FSAKDYHDPPPAPLIDAEELTKWSFYRAIIAEFIATLLFLYITVLTVIGYRAQSDTTRGGDD--CGGVGIL

                       110       120       130       140       150       160       170       180       190       200
                         |         |         |         |         |         |         |         |         |         |
AtPIP12      GIAWAFGGMIFALVYCTAGISGGHINPAVTFGLFLARKLSLTRAVYYIVMQCLGAICGAGVVKGFQPKQYQALGGGANTIAHGYTKGSGLGAEIIGTFVL
NtAQP1       GVAWAFGGMIFALVYCTAGISGGHINPAVTFGLFLARKLSLTRAIFYIVMQCLGAICGAGVVKGFMVGPYQRLGGGANVVNHGYTKGDGLGAEIIGTFVL
BpePIP11     GIAWAFGGMIFALVYCTAGISGGHINPAVTFGLFLARKLSLTRAVFYIVMQCLGAICGAGVVKGFQKNQYERLGGGANTISSGYSKGDGLGAEIVGTFVL
BpePIP13     GIAWAFGGMIFALVYCTAGISGGHINPAVTFGLFLARKLSLTRALFYIVMQCLGAICGAGVVKGFQKSQYEILGGGANFVNPGYTKGDGLGAEIVGTFVL
BpePIP12     GIAWAFGGMIFALVYCTAGISGGHINPAVTFGLLLARKLSLTRAVFYIIMQCLGAICGAGVVKGFGKTPFEINGGGANVVNHGYTKGDGLGAEIVGTFVL
BpePIP14     GIAWAFGGMIFALVYCTAGISGGHINPAVTFGLFLARKLSLTRALFYIVMQCLGAICGAGVVKGFQKTQYERVGGGANVVNHGYSKGDGLGAEIVGTFVL
HvPIP2_1     GIAWAFGGMIFVLVYCTAGVSGGHINPAVTFGLFLARKVSLIRALLYIIAQCLGAICGVGLVKGFQSSYYVRYGGGANELSAGYSKGTGLAAEIIGTFVL
BpePIP21     GIAWAFGGMIFVLVYCTAGISGGHINPAVTFGLFLARKVSLIRAVSYMVAQCLGAICGVGLVKAFMKHDYNMQGGGANSVSPGYSTGTALGAEIIGTFVL
BpePIP22     GVAWAFGGMIFVLVYSTAGISGGHINPAVTFGLFLARKVSLIRAICYMVAQCLGAICGAGLVKGIMKDSYDAFGGGTNTVSPTYSKGTALGAEIIGTFVL
BpePIP23     GVAWAFGGMIFILVYSTAGISGGHINPAVTFGLFLARKVSLIRAICYMVAQCLGAICGAGLVKGIMKDYYDALGGGTNTVSPTYNKGTALGAEIIGTFVL
BpePIP24     GIAWAFGGMIFVLVYCTAGISGGHINPAVTFGLFLARKVSLVRAVSYMVAQCLGAISGVGLVKAFNKAHYTRYGGGANTLADGYSTGVGLGAEIIGTFVL
BpePIP25     GIAWAFGGMIFVLVYCTAGISGGHINPAVTFGLFLARKVSLIRALLYMVAQCLGAICGVGLVKAFQKSDYNRYGGGANELSVGYNKGVGLGAEIIGTFVL
BpePIP26     GIALAFGGMIFVLVYCTAGISGGHINPAVTFGLFLARKVSLVRAILYMVAQCLGAICGCGLVKAFQRAHYTRYGGGANELSVGYSKGTGLAAEIIGTFVL
```

```
210         220         230         240         250         260         270         280         290         300         310         320
                                    |           |           |           |           |           |           |           |           |           |
AtPIP12     VYTVFSATDAKRNARDSHVPILAPLPIGFAVFLVHLATIPITGTGINPARSLGAAIIFNKDNAWDDHWVFWVGPFIGAALAALYHVIVIRAIPFKSRS--
NtAQP1      VYTVFSATDAKRNARDSYVPILAPLPIGFAVFLVHLATIPITGTGINPARSLGAAIIYNTDQAWDDHWIFWVGPFIGAALAAVYHQIIIRAIPFHKSS--
BpePIP11    VYTVFSATDAKRNARDSHVPILAPLPIGFAVFLVHLATIPITGTGINPARSLGAALIFNKEQAWHNHWIFWVGPFIGAALAALYHQIVIRAIPFKSK---
BpePIP13    VYTVFSATDAKRSARDSHVPILAPLPIGFAVFLVHLATIPITGTGINPARSLGAAIIYNRDRAWDDHWIFWVGPFIGAALAALYHQVVIRAIPFKSK---
BpePIP12    VYTVFSATDAKRNARDSHVPILAPLPIGFAVFLVHLATIPITGTGINPARSLGAAIIYNKDHAWDDHWIFWVGPFIGAALAAVYHQIVIRAIPFKARG--
BpePIP14    VYTVFSATDAKRNARDSHVPLLAPLPIGFAVFLVHLATIPITGTGINPARSLGAAIIYNKDHAWDDQWIFWVGPFIGAALAALYHQIVIRAMPFKTRA--
HvPIP2_1    VYTVFSATDPKRNARDSHIPVLAPLPIGFAVFMVHLATIPITGTGINPARSLGAAVIYNTDKAWDDQWIFWVGPLIGAAIAAAYHQYVLRASAAK-LGSY//
BpePIP21    VYTVFSATDPKRSARDSHVPVLAPLPIGFAVFMVHLATIPITGTGINPARSLGAAVIYNNGKIWDDQWIFWVGPFVGALAAAAYHQYILRAAAIKALGSF//
BpePIP22    VYTVFTATDPKRNARDSYVPVLAPLPIGFAVFMVHLATIPITGTGINPARSFGVAVIYNNDKVWDDQWIFWAGPFAGALAAALYYQFILRATAMKALGSF//
BpePIP23    VYTVFSATDPKRKARDSHVPVLAPLPIGFAVFMVHLATIPITGTGINPARSFGAAVIYNNDKVWDDQWIFWVGPFAGALAAALYHQHILRATAIKALGSF//
BpePIP24    VYTVFSATDPKRNARDSHVPVLAPLPIGFAVFMVHLATIPITGTGINPARSLGAALIYNKAKAWDDQWLFWVGPFIGAAIAAFYHQFILRAGAAKALGSF//
BpePIP25    VYTVFSATDPKRSARDSHVPVLAPLPIGFAVFMVHLATIPITGTGINPARSFGAAVIYNKDKAWDDQWIFWVGPFIGAAIAAFYHQFILRAGAIKALGSF//
BpePIP26    VYTVFSATDPKRSARDSHVPVLAPLPIGFAVFMVHLATIPITGTGINPARSFGAAVMYGKTKAWDDQWIFWVGPFIGAAIAAFYHQFILRAGAAKALGSF//
```

H_2O_2 Transporters

PIP2

```
                        110         120         130         140         150         160         170         180         190         200
                          |           |           |           |           |           |           |           |           |           |
AtPIP2;1    WAFGGMIFILVYCTAGISGGHINPAVTFGLFLARKVSLPRALLYIIAQCLGAICGVGFVKAFQSSYYTRYGGGANSLADGYSTGTGLAAEIIGTFVLVYT
AtPIP2;4    WAFGGMIFVLVYCTAGISGGHINPAVTVGLFLARKVSLVRTVLYIVAQCLGAICGCGFVKAFQSSYYTRYGGGANELADGYNKGTGLGAEIIGTFVLVYT
AtNIP1;2    IVWGLTVMVLVYSLGHISGAHFNPAVTIAFASCGRFPLKQVPAYVISQVIGSTLAAATLRLLFGLDQDVCSGKHDVFVGTLPSGSNLQSFVIEFIITFYL
AtTIP1;1    VAHAFGLFVAVSVGANISGGHVNPAVTFGAFIGGNITLLRGILYWIAQLLGSVVACLILKFATGGLAVPAFG----LSAGVGVLNAFVFEIVMTFGLVYT
AtTIP1;2    LAHAFGLFVAVSVGANISGGHVNPAVTFGVLLGGNITLLRGILYWIAQLLGSVAACFLLSFATGGEPIPAFG----LSAGVGSLNALVFEIVMTFGLVYT
AtTIP2;3    IAHAFALFVGVSIAANISGGHLNPAVTLGLAIGGNITLITGFFYWIAQCLGSIVACLLLVFVTNGKSVPTHG----VSAGLGAVEGVVMEIVVTFALVYT
BpePIP1;1   WAFGGMIFALVYCTAGISGGHINPAVTFGLFLARKLSLTRAVFYIVMQCLGAICGAGVVKGFQKNQYERLGGGANTISSGYSKGDGLGAEIVGTFVLVYT
BpePIP1;2   WAFGGMIFALVYCTAGISGGHINPAVTFGLLLARKLSLTRAVFYIIMQCLGAICGAGVVKGFGKTPFEINGGGANVVNHGYTKGDGLGAEIVGTFVLVYT
BpePIP1;3   WAFGGMIFALVYCTAGISGGHINPAVTFGLFLARKLSLTRALFYIVMQCLGAICGAGVVKGFQKSQYEILGGGANFVNPGYTKGDGLGAEIVGTFVLVYT
BpePIP1;4   WAFGGMIFALVYCTAGISGGHINPAVTFGLFLARKLSLTRALFYIVMQCLGAICGAGVVKGFQKTQYERVGGGANVVNHGYSKGDGLGAEIVGTFVLVYT
BpePIP2;1   WAFGGMIFVLVYCTAGISGGHINPAVTFGLFLARKVSLIRAVSYMVAQCLGAICGVGLVKAFMKHDYNMQGGGANSVSPGYSTGTALGAEIIGTFVLVYT
BpePIP2;2   WAFGGMIFVLVYSTAGISGGHINPAVTFGLFLARKVSLIRAICYMVAQCLGAICGAGLVKGIMKDSYDAFGGGTNTVSPTYSKGTALGAEIIGTFVLVYT
BpePIP2;3   WAFGGMIFILVYSTAGISGGHINPAVTFGLFLARKVSLIRAICYMVAQCLGAICGAGLVKGIMKDYYDALGGGTNTVSPTYNKGTALGAEIIGTFVLVYT
BpePIP2;4   WAFGGMIFVLVYCTAGISGGHINPAVTFGLFLARKVSLVRAVSYMVAQCLGAISGVGLVKAFNKAHYTRYGGGANTLADGYSTGVGLGAEIIGTFVLVYT
```

```
BpePIP2;5     WAFGGMIFVLVYCTAGISGGHINPAVTFGLFLARKVSLIRALLYMVAQCLGAICGVGLVKAFQKSDYNRYGGGANELSVGYNKGVGLGAEIIGTFVLVYT
BpePIP2;6     LAFGGMIFVLVYCTAGISGGHINPAVTFGLFLARKVSLVRAILYMVAQCLGAICGCGLVKAFQRAHYTRYGGGANELSVGYSKGTGLAAEIIGTFVLVYT

                       210       220       230       240       250       260       270       280       290       300
                         |         |         |         |         |         |         |         |         |         |
AtPIP2;1      VFSATDPKRSARDSHVPVLAPLPIGFAVFMVHLATIPITGTGINPARSFGAAVIYNKSKPWDDHWIFWVGPFIGAAIAAFYHQFVLRA----SGSKSLGS
AtPIP2;4      VFSATDPKRNARDSHVPVLAPLPIGFAVFMVHLATIPITGTGINPARSFGAAVIYNNEKAWDDQWIFWVGPMIGAAAAAFYHQFILRA----AAIKALGS
AtNIP1;2      MFVISGVATDNRA--IGELAGLAVGSTVLLNVIIAGPVSGASMNPGRSLGPAMVYS---CYRGLWIYIVSPIVGAVSGAWVYNMVRYTDKPLREITKSGS
AtNIP1;1      VYATA---IDPKNGSLGTIAPIAIGFIVGANILAGGAFSGASMNPAVAFGPAVVSW---TWTNHWVYWAGPLVGGGIAGLIYEVFFIN-TTHEQLPTTDY
AtTIP1;2      VYATA---VDPKNGSLGTIAPIAIGFIVGANILAGGAFSGASMNPAVAFGPAVVSW---TWTNHWVYWAGPLIGGGLAGIIYDFVFIDENAHEQLPTTDY
AtTIP2;3      VYATA---ADPKKGSLGTIAPIAIGFIVGANILAAGPFSGGSMNPARSFGPAVVSG---DLSQIWIYWVGPLVGGALAGLIYGDVFIG-SYEAVETREI
BpePIP1;1     VFSATDAKRNARDSHVPILAPLPIGFAVFLVHLATIPITGTGINPARSLGAALIFNKEQAWHNHWIFWVGPFIGAALAALYHQIVIRA----IPFKSK--
BpePIP1;2     VFSATDAKRNARDSHVPILAPLPIGFAVFLVHLATIPITGTGINPARSLGAAIIYNKDHAWDDHWIFWVGPFIGAALAAVYHQIVIRA----IPFKARG-
BpePIP1;3     VFSATDAKRSARDSHVPILAPLPIGFAVFLVHLATIPITGTGINPARSLGAAIIYNRDRAWDDHWIFWVGPFIGAALAALYHQVVIRA----IPFKSK--
BpePIP1;4     VFSATDAKRNARDSHVPLLAPLPIGFAVFLVHLATIPITGTGINPARSLGAAIIYNKDHAWDDQWIFWVGPFIGAALAALYHQIVIRA----MPFKTRA-
BpePIP2;1     VFSATDPKRSARDSHVPVLAPLPIGFAVFMVHLATIPITGTGINPARSLGAAVIYNNGKIWDDQWIFWVGPFVGALAAAAYHQYILRA----AAIKALGS
BpePIP2;2     VFTATDPKRNARDSYVPVLAPLPIGFAVFMVHLATIPITGTGINPARSFGVAVIYNNDKVWDDQWIFWAGPFAGALAAALYYQFILRA----TAMKALGS
BpePIP2;3     VFSATDPKRKARDSHVPVLAPLPIGFAVFMVHLATIPITGTGINPARSFGAAVIYNNDKVWDDQWIFWVGPFAGALAAALYHQHILRA----TAIKALGS
BpePIP2;4     VFSATDPKRNARDSHVPVLAPLPIGFAVFMVHLATIPITGTGINPARSLGAALIYNKAKAWDDQWLFWVGPFIGAAIAAFYHQFILRA----GAAKALGS
BpePIP2;5     VFSATDPKRSARDSHVPVLAPLPIGFAVFMVHLATIPITGTGINPARSFGAAVIYNKDKAWDDQWIFWVGPFIGAAIAAFYHQFILRA----GAIKALGS
BpePIP2;6     VFSATDPKRSARDSHVPVLAPLPIGFAVFMVHLATIPITGTGINPARSFGAAVMYGKTKAWDDQWIFWVGPFIGAAIAAFYHQFILRA----GAAKALGS
```

TIP & XIP

```
                       110       120       130       140       150       160       170       180       190       200
                         |         |         |         |         |         |         |         |         |         |
AtTIP1;2      AHAFGLFVAVSVGANISGGHVNPAVTFGVLLGGNITLLRGILYWIAQLLGSVAACFLLSFATGGEPIPAFGLSAGVGSLN----------------ALVF
AtTIP2;3      AHAFALFVGVSIAANISGGHLNPAVTLGLAIGGNITLITGFFYWIAQCLGSIVACLLLVFVTNGKSVPTHGVSAGLGAVE----------------GVVM
AtPIP2;1      AFGGMIFILVYCTAGISGGHINPAVTFGLFLARKVSLPRALLYIIAQCLGAICGVGFVKAFQSSYYTRYGGGANSLADGYSTGT------------GLAA
AtPIP2;4      AFGGMIFVLVYCTAGISGGHINPAVTVGLFLARKVSLVRTVLYIVAQCLGAICGCGFVKAFQSSYYTRYGGGANELADGYNKGT------------GLGA
AtNIP1;2      VWGLTVMVLVYSLGHISGAHFNPAVTIAFASCGRFPLKQVPAYVISQVIGSTLAAATLRLLFGLDQDVCSGKHDVFVGTLPSGSN---------LQSFVI
BpeTIP1;1     AHAFGLFVAVSISANISGGHVNPAVTFGAFIGGNITLLRGIVYWIAQLLGSTVACLLLKFATNGLTTSAFSLSSGVGVWN----------------AFVL
BpeTIP1;2     AHGFGLFVAVSIGANISGGHVNPAVTFGAFLGGHISLIRGLLYWVAQLLGSVVACLLLKFSTGGLETPAFSLSSDVSVWN----------------AVVL
BpeTIP1;3     AHAFALFVAVSVGANISGGHVNPAVTFGAFIGGNITLLRTILYWIAQLLGSVVACLLLKFATGGLETSAFSLSSGVSVWN----------------AVVF
BpeTIP2;1     CHGFALFVAVAVGANISGGHVNPAVTFGLALGGQITLLTGIFYWIAQLLGSIAACFLLKAVT-GLAIPTHSVASGVGAIE----------------GVVM
BpeTIP2;2     AHAFALFVGVSIAANISGGHLNPAVTFGLALGGNITILTGIFYWIAQCLGSIVACLLLQFVTNGLSIPTHGVASGMNAVE----------------GVVM
BpeTIP3;1     AHAFSLFAAVAASLNISGGHVNPAVTFGTLLGGRISVLRAVFYWIAQLLGAVVASLLLRLVTNNMRPVGFYVTSGVGEWH----------------GLIF
BpeTIP4;1     THALVVAVMISAG-HISGGHLNPAVTLGLLAGGHITIFRSFLYWIDQLLAASAACIILYYITGGLTTPIHSLASGVGYLQ----------------GVIW
BpeTIP5;1     TNAFALSAAVYVAANISGGHVNPAVTFGMAVGGHISVPTALFYWLSQMLASVMASLLLKMTVVGQHVPTYVIAEEMTGFG----------------ASLM
BpeXIP2;1*    LIAIITAILLLAANPISGGHMNPVITLAAALVGVISVSRAAVYILAQCAGAVLGALALKAVVNSTIEDTFSLGGCTLTVIAPGPNGPISVGLGTSQGLWL
BpeXIP1;1     AVFIIAFLFLLATIPLSGGHMNPILTFIACLKGLITLARASIYVLAQCLGSIIGFFIIKNVMNHQTGHNYFLGGCTISALNRGT------------ALVL
```

```
BpeXIP1;2    AVFIIAFLFLMATAPLSGGHMSPIFTFIACLKGLITLARASIYVLAQCLGSIIGFFIIKNVMNHQIGHKYFLGGCTISALSRGT------------ALVL
BpeXIP1;3    AVFIIAFLFLMATVPLSGGHMSPIFTFIACLKGLITLARASMYVLAQCLGSIIGFFIIKNVMNHQTGHKYFLGGCTISTLSRGT------------ALVL
                         210       220       230       240       250       260       270       280       290       300
                           |         |         |         |         |         |         |         |         |         |
AtPIP2;4     EIIGTFVLVYTVFSATDPKRNARDSHVPVLAPL---PIGFAVFMVHLAT--IPITGTGINPARSFGAAVIYNNEKAWDDQWIFWVGPMIGAAAAAFYHQF
AtTIP23      EIVVTFALVYTVYATAADPKKG------SLGTIAPIAIGFIVGANILAAG--PFSGGSMNPARSFGPAVVSGD---LSQIWIYWVGPLVGGALAGLIYGD
AtNIP1;2     EFIITFYLMFVISGVATDNR--------AIGELAGLAVGSTVLLNVIIAG--PVSGASMNPGRSLGPAMVYSC---YRGLWIYIVSPIVGAVSGAWVYNM
AtTIP11      EIVMTFGLVYTVYATAIDPKNG------SLGTIAPIAIGFIVGANILAGG--AFSGASMNPAVAFGPAVVSWT---WTNHWVYWAGPLVGGGIAGLIYEV
AtTIP12      EIVMTFGLVYTVYATAVDPKNG------SLGTIAPIAIGFIVGANILAGG--AFSGASMNPAVAFGPAVVSWT---WTNHWVYWAGPLIGGGLAGIIYDF
BpeTIP1;1    EIVMTFGLVYTVYATAVDPKRG------SVGVIAPIAIGFIVGANILAGG--AFDGASMNPAVSFGPALVSWT---WENHWVYWAGPLIGGGLAGLVYEF
BpeTIP1;2    EIVMTFGLVYTVYATAVDPKKG------NIGILAPIAIGLIVGANILAGG--AFDGASMNPAVSFGPAVVSWS---WDNHWVYWVGPLIGSAIAAIVYEL
BpeTIP1;3    EIVMTFGLVYTVYATAVDPKKG------NVGTVAPIAIGFIVGANILVGG--AFDGASMNPAVSFGPAVVSWS---WTNHWVYWLGPMVGAAIAAIVYDT
BpeTIP2;1    EIIITFALVYTVYATAADPKKG------SLGTIAPIAIGFIVGANILAAG--PFSGGSMNPARSFGPAVASGD---FHDNWIYWAGPLVGGGLAGLIYGN
BpeTIP2;2    EIVITFALVYTVYATAADPKKG------SLGIIAPIAIGFIVGANILAAG--PFSGGSMNPARSFGPAVVSGD---FSQNWIYWVGPLIGGGLAGLVYGG
BpeTIP3;1    EIVMTFTLVYTVFATAIDPKRG------SLGTIAPLAIGFIVGANILAGG--PFDGACMNPARAFGPALVGWR---WRNHWIYWVGPLLGGGLAGLVYEY
BpeTIP4;1    EVILTFSLLFTVYATIVDPKKG------ALDGLGPTLTGFVVGANILAGG--AFSGASMNPARSFGPALISGD---WTDHWVYWVGPLIGGGLAGFIYEN
BpeTIP5;1    EGVLTFALVYTVYA-AGDTRRG------TMGAIGPIAIGSIAGANVLAAG--PFSGGSMNPACAFGSAVTAGS---FKNQAVYWVGPLIGAAAAGLVYDN
BpeXIP2;1*   EIICTFVLLFASIWIALDHRQARALGRVIIFSVIGIVVGLLVFVSTTVTGAKGYAGAGMNPARCLGPAIVRGGHL-WNGHWVFWAGPSIASVAFYLYTKI
BpeXIP1;1    EFCCTFVLLFVGVTVAFDKKRCKELGLAVVCAVVAAAWALAVFVSITVTGQVGYAGVGLNPARCLGPALLHGGVL-WDGHWVFWVGPFLACIVYYGYSMS
BpeXIP1;2    EFCCTFVVLFVGVTVAFDKKRCKELGLAVVCVVVAGAMALAVFVSITVTGQASYAGVGLNPARCLGPVLLQGGVL-WDGHWVLWVGPFLACIVYYGYSMN
BpeXIP1;3    EFCCTFVVLFVGVTVAFDKKRCKELGVAVVCVVVAGAMALAVFVSITVTGQAGYAGVGLNPARCLGPALLQGGVL-WDGHWIFWVGPFLACIVYYGYSMN
```

Silicic Acid Transporters

NIP

```
                   10        20        30        40        50        60        70        80        90       100
                    |         |         |         |         |         |         |         |         |         |
HvNIP2;1    -MASNSRSNSRATFSSEIHDIGTVQNSTTPSMVYYTERSIADYFPPHLLKKVVSEVVSTFLLVFVTCGAAAISAHDVTRISQLGQSVAGGLIVVVMIYAV
ZmNIP2;1    -MSTNSRSNSRANFNNEIHDIGTAQNSSMPPT-YY-DRSLADIFPPHLLKKVVSEVVSTFLLVFVTCGAAGIYGSDKDRISQLGQSVAGGLIVTVMIYAV
OsNIP2;1    MASNNSRTNSRANYSNEIHDLSTVQNGTMPTM-YYGEKAIADFFPPHLLKKVVSEVVATFLLVFMTCGAAGISGSDLSRISQLGQSIAGGLIVTVMIYAV
BpeNIP2;1   -MATDPKPKNLAEANEFVSLENPISRKPKPGLL---RALLEEHYPPGFLSKVVAETIATYLLVFVTCGSAALSAADENKVSKLAASVAGGLIVTVMIYAV

                  110       120       130       140       150       160       170       180       190       200
                    |         |         |         |         |         |         |         |         |         |
HvNIP2;1    GHISGAHMNPAVTLAFAIFRHFPWIQVPFYWAAQFTGAICASFVLKAVLHPITVIGTTEPVGPHWHALVIEVVVTFNMMFVTLAVATDTRAVGELAGLAV
ZmNIP2;1    GHISGAHMNPAVTLAFAVFRHFPWIQVPFYWAAQFTGSICASFVLKAVLHPIAVLGTTTPTGPHWHSLVIEIIVTFNMMFVTLAVATDTRAVGELAGLAV
OsNIP2;1    GHISGAHMNPAVTLAFAVFRHFPWIQVPFYWAAQFTGAICASFVLKAVIHPVDVIGTTTPVGPHWHSLVVEVIVTFNMMFVTLAVATDTRAVGELAGLAV
BpeNIP2;1   GHISGAHMNPAVTLAFAAVRHFPWKQVPLYAAAQLAGAISAAFTLRVILNPIKELGTTSPTGTEIEALVMEIVVTFTMMFTASAVATDTKAIGELAGLAV

                  210       220       230       240       250       260       270       280       290       300
                    |         |         |         |         |         |         |         |         |         |
HvNIP2;1    GSAVCITSIFAGAVSGGSMNPARTLGPALASNLYTGLWLYFLGPVLGTLSGAWTYTYIRFEDPP--KDAPQKLSSFKLRRLQSQ-SVAADD-DELDHIPV
ZmNIP2;1    GSAVCITSIFAGAVSGGSMNPARTLGPALASNLYTGLWIYFLGPVLGTLSGAWTYTYIRFEEAPSHKDMSQKLSSFKLRRLQSQ-SVAVDD-DELDHIQV
OsNIP2;1    GSAVCITSIFAGAISGGSMNPARTLGPALASNKFDGLWIYFLGPVMGTLSGAWTYTFIRFEDTPK-EGSSQKLSSFKLRRLRSQQSIAADDVDEMENIQV
BpeNIP2;1   GSAVCITSILAGPISGGSMNPARTIGPAIASASYKGIWVYLVGPVTGTLLGAWSYKLIRVSDEPLYPMSKEGHSKEMVNKYPNN-SV------------
```

Urea Transporters

PIP1 & PIP2

```
                   110       120       130       140       150       160       170       180       190       200
                     |         |         |         |         |         |         |         |         |         |
NtAQP1       TVMGLKRSDSL------CSSVGIQGVAWAFGGMIFALVYCTAGISGGHINPAVTFGLFLARKLSLTRAIFYIVMQCLGAICGAGVVKGFMVGPYQRLGGG
NtTIPa       SAMAANKLNGD---------------AMAHALVVAVTISAGFRISGGHLNPAVTLGLCMGGHITVFRSILYWIDQLLASVAACALLNYLTAG----LETP
AtTIP4;1     SAMATDSLVGN-------TLVGLFAVAVAHAFVVAVMISAG-HISGGHLNPAVTLGLLLGGHISVFRAFLYWIDQLLASSAACFLLSYLTGG----MGTP
CpNIP1       AAALNGSDAQR---------VSQLGASVAGGLIVTVMIYAVGHISGAHMNPAVTTAFAATRHFPWKQVPLYAAAQLSGATCAAFTLRLLLHPIKHLGT--
AtNIP6;1     TAIVNQKTDGA---------ETLIGCAASAGLAVMIVILSTGHISGAHLNPAVTIAFAALKHFPWKHVPVYIGAQVMASVSAAFALKAVFEPTMSGGV--
AtNIP2;1     AIAVNAQHNHV---------VTLVGIAVVWGIVIMVLVYCLGHLS-AHFNPAVTLALASSQRFPLNQVPAYITVQVIGSTLASATLRLLFDLNNDVCSKK
BpePIP1;2    TVMGVKRSSSV------CASVGIQGIAWAFGGMIFALVYCTAGISGGHINPAVTFGLLLARKLSLTRAVFYIIMQCLGAICGAGVVKGFGKTPFEINGGG
BpePIP1;4    TVMGVSRAPSK------CASVGVQGIAWAFGGMIFALVYCTAGISGGHINPAVTFGLFLARKLSLTRALFYIVMQCLGAICGAGVVKGFQKTQYERVGGG
BpePIP1;1    TVMGVSKSPSK------CSTVGIQGIAWAFGGMIFALVYCTAGISGGHINPAVTFGLFLARKLSLTRAVFYIVMQCLGAICGAGVVKGFQKNQYERLGGG
BpePIP1;3    TVMGVAKSPSK------CSTVGIQGIAWAFGGMIFALVYCTAGISGGHINPAVTFGLFLARKLSLTRALFYIVMQCLGAICGAGVVKGFQKSQYEILGGG
BpePIP2;1    TVIGHKKATGP------CDGVGLLGIAWAFGGMIFVLVYCTAGISGGHINPAVTFGLFLARKVSLIRAVSYMVAQCLGAICGVGLVKAFMKHDYNMQGGG
BpePIP2;2    TVIGYKKQTDP------CETVGLLGVAWAFGGMIFVLVYSTAGISGGHINPAVTFGLFLARKVSLIRAICYMVAQCLGAICGAGLVKGIMKDSYDAFGGG
BpePIP2;3    TVIGYKKQNDQ------CETVGLLGVAWAFGGMIFILVYSTAGISGGHINPAVTFGLFLARKVSLIRAICYMVAQCLGAICGAGLVKGIMKDYYDALGGG
BpePIP2;4    TVIGYKSQIDPANDGEACGGVGVLGIAWAFGGMIFVLVYCTAGISGGHINPAVTFGLFLARKVSLVRAVSYMVAQCLGAISGVGLVKAFNKAHYTRYGGG
BpePIP2;5    TVIGYKSQTDPSKNPDQCGGVGILGIAWAFGGMIFVLVYCTAGISGGHINPAVTFGLFLARKVSLIRALLYMVAQCLGAICGVGLVKAFQKSDYNRYGGG
BpePIP2;6    TVIGYRAQSDTTRGGDDCGGVGILGIALAFGGMIFVLVYCTAGISGGHINPAVTFGLFLARKVSLVRAILYMVAQCLGAICGCGLVKAFQRAHYTRYGGG

                   210       220       230       240       250       260       270       280       290       300
                     |         |         |         |         |         |         |         |         |         |
NtAQP1       ANV---VNHGYTKGDGLGAEIIGTFVLVYTVFSA-TDAKRNARDSYVPILAPLPIGFAVFLVHLATIPITGTGINPARSLGAAIIYNTDQAWDDHWIFWV
NtTIPa       VHT---LANGVSYGQGIIMEVILTFSLLFTVYTTIVDPKKGILEGMGPLLTGLVVGANI----MAGGPFSGASMNPARSFGPAFVSGI---WTDHWVYWV
AtTIP4;1     VHT---LASGVSYTQGIIWEIILTFSLLFTVYATIVDPKKGSLDGFGPLLTGFVVGANI----LAGGAFSGASMNPARSFGPALVSGN---WTDHWVYWV
CpNIP1       ------TTPSGSDLQALVMEIVVTFSMMFVTCAVATDTKA------VGELAGLAVGSAVCITSILAGPVSGGSMNPVRTLGPAMASDN---YKGLWVYFV
AtNIP6;1     ------TVPTVGLSQAFALEFIISFNLMFVVTAVATDTRA------VGELAGIAVGATVMLNILIAGPATSASMNPVRTLGPAIAANN---YRAIWVYLT
AtNIP2;1     HDVFLGSSPSGSDLQAFVMEFIITGFLMLVVCAVTTTKRT------TEELEGLIIGATVTLNVIFAGEVSGASMNPARSIGPALVWGC---YKGIWIYLL
BpePIP1;2    ANV---VNHGYTKGDGLGAEIVGTFVLVYTVFSA-TDAKRNARDSHVPILAPLPIGFAVFLVHLATIPITGTGINPARSLGAAIIYNKDHAWDDHWIFWV
BpePIP1;4    ANV---VNHGYSKGDGLGAEIVGTFVLVYTVFSA-TDAKRNARDSHVPLLAPLPIGFAVFLVHLATIPITGTGINPARSLGAAIIYNKDHAWDDQWIFWV
BpePIP1;1    ANT---ISSGYSKGDGLGAEIVGTFVLVYTVFSA-TDAKRNARDSHVPILAPLPIGFAVFLVHLATIPITGTGINPARSLGAALIFNKEQAWHNHWIFWV
BpePIP1;3    ANF---VNPGYTKGDGLGAEIVGTFVLVYTVFSA-TDAKRSARDSHVPILAPLPIGFAVFLVHLATIPITGTGINPARSLGAAIIYNRDRAWDDHWIFWV
BpePIP2;1    ANS---VSPGYSTGTALGAEIIGTFVLVYTVFSA-TDPKRSARDSHVPVLAPLPIGFAVFMVHLATIPITGTGINPARSLGAAVIYNNGKIWDDQWIFWV
BpePIP2;2    TNT---VSPTYSKGTALGAEIIGTFVLVYTVFTA-TDPKRNARDSYVPVLAPLPIGFAVFMVHLATIPITGTGINPARSFGVAVIYNNDKVWDDQWIFWA
BpePIP2;3    TNT---VSPTYNKGTALGAEIIGTFVLVYTVFSA-TDPKRKARDSHVPVLAPLPIGFAVFMVHLATIPITGTGINPARSFGAAVIYNNDKVWDDQWIFWV
BpePIP2;4    ANT---LADGYSTGVGLGAEIIGTFVLVYTVFSA-TDPKRNARDSHVPVLAPLPIGFAVFMVHLATIPITGTGINPARSLGAALIYNKAKAWDDQWLFWV
BpePIP2;5    ANE---LSVGYNKGVGLGAEIIGTFVLVYTVFSA-TDPKRSARDSHVPVLAPLPIGFAVFMVHLATIPITGTGINPARSFGAAVIYNKDKAWDDQWIFWV
BpePIP2;6    ANE---LSVGYSKGTGLAAEIIGTFVLVYTVFSA-TDPKRSARDSHVPVLAPLPIGFAVFMVHLATIPITGTGINPARSFGAAVMYGKTKAWDDQWIFWV
```

TIP & XIP

```
                     110       120       130       140       150       160       170       180       190       200
                       |         |         |         |         |         |         |         |         |         |
CpNIP1       AAALNGSDAQRVS-----QLGASVAGGLIVTVMIYAVGHISGAHMNPAVTTAFAATRHFPWKQVPLYAAAQLSGATCAAFTLRLLLHPIKHLGT------
AtNIP6;1     TAIVNQKTDGAET-----LIGCAASAGLAVMIVILSTGHISGAHLNPAVTIAFAALKHFPWKHVPVYIGAQVMASVSAAFALKAVFEPTMSGGV------
AtNIP2;1     AIAVNAQHNHVVT-----LVGIAVVWGIVIMVLVYCLGHLS-AHFNPAVTLALASSQRFPLNQVPAYITVQVIGSTLASATLRLLFDLNNDVCSKKHDVF
NtAQP1       TVMGLKRSDSLCSS--VGIQGVAWAFGGMIFALVYCTAGISGGHINPAVTFGLFLARKLSLTRAIFYIVMQCLGAICGAGVVKGFM---VGPYQRLGGGA
AtTIP4;1     SAMATDSLVGNTLV---GLFAVAVAHAFVVAVMISAG-HISGGHLNPAVTLGLLLGGHISVFRAFLYWIDQLLASSAACFLLSYLTGGMGTPVH------
NtTIPa       SAMAANKLNGD-----------AMAHALVVAVTISAGFRISGGHLNPAVTLGLCMGGHITVFRSILYWIDQLLASVAACALLNYLTAGLETPVH------
BpeTIP1;1    SGMAFNKLTDNGSTTPSGLVQAALAHAFGLFVAVSISANISGGHVNPAVTFGAFIGGNITLLRGIVYWIAQLLGSTVACLLLKFATNGLTTSAF------
BpeTIP1;2    SGVAFNKLTDNGSTTPAGLVAAALAHGFGLFVAVSIGANISGGHVNPAVTFGAFLGGHISLIRGLLYWVAQLLGSVVACLLLKFSTGGLETPAF------
BpeTIP1;3    SGMAFNKLTDNGSTTPSGLVAAALAHAFALFVAVSVGANISGGHVNPAVTFGAFIGGNITLLRTILYWIAQLLGSVVACLLLKFATGGLETSAF------
BpeTIP3;1    SLLALI-YKDTGST-PSELVAIALAHAFSLFAAVAASLNISGGHVNPAVTFGTLLGGRISVLRAVFYWIAQLLGAVVASLLLRLVTNNMRPVGF------
BpeTIP2;1    SAIAYNKLTSNAALDPSGLVGIAICHGFALFVAVAVGANISGGHVNPAVTFGLALGGQITLLTGIFYWIAQLLGSIAACFLLKAVT-GLAIPTH------
BpeTIP2;2    SAISYGKLTSDAALDPAGLVAVAVAHAFALFVGVSIAANISGGHLNPAVTFGLALGGNITILTGIFYWIAQCLGSIVACLLLQFVTNGLSIPTH------
BpeTIP4;1    SAMAADKLDSGSLV---GLFAVAITHALVVAVMISAG-HISGGHLNPAVTLGLLAGGHITIFRSFLYWIDQLLAASAACIILYYITGGLTTPIH------
BpeTIP5;1    SAMSSRKLMPDVASDASSLVVVAITNAFALSAAVYVAANISGGHVNPAVTFGMAVGGHISVPTALFYWLSQMLASVMASLLLKMTVVGQHVPTY------
BpeXIP1;1    SIIACLDSQEVDPK--LL---VPLAVFIIAFLFLLATIPLSGGHMNPILTFIACLKGLITLARASIYVLAQCLGSIIGFFIIKNVMNHQTGHNYFLGGCT
BpeXIP1;2    SIIACLDSHEVNPK--LL---VPLAVFIIAFLFLMATAPLSGGHMSPIFTFIACLKGLITLARASIYVLAQCLGSIIGFFIIKNVMNHQIGHKYFLGGCT
BpeXIP1;3    SIIACLDSHEVDPK--LL---VPLAVFIIAFLFLMATVPLSGGHMSPIFTFIACLKGLITLARASMYVLAQCLGSIIGFFIIKNVMNHQTGHKYFLGGCT
BpeXIP2;1    IVISSFETKTESPK--LI---MSLLIAIITAILLLAANPISGGHMNPAITLAAALVGVISVSRAAVYILAQCAGAVLGALALKAVVNSTIEDTFSLGGCT
                     210       220       230       240       250       260       270       280       290       300
                       |         |         |         |         |         |         |         |         |         |
CpNIP1       --TTPSGSDL---------QALVMEIVVTFSMMFVTCAVATDTK--A------VGELAGLAVGSAVCITSILAG--PVSGGSMNPVRTLGPAMASD---N
AtNIP6;1     --TVPTVGLS---------QAFALEFIISFNLMFVVTAVATDTR--A------VGELAGIAVGATVMLNILIAG--PATSASMNPVRTLGPAIAAN---N
AtNIP2;1     LGSSPSGSDL---------QAFVMEFIITGFLMLVVCAVTTTKR--T------TEELEGLIIGATVTLNVIFAG--EVSGASMNPARSIGPALVWG---C
AtTIP4;1     -TLASGVSYT---------QGIIWEIILTFSLLFTVYATIVDPKKGS------LDGFGPLLTGFVVGANILAGG--AFSGASMNPARSFGPALVSG---N
NtAQP1       NVVNHGYTKG---------DGLGAEIIGTFVLVYTVFSATDAKRNARD---SYVPILAPLPIGFAVFLVHLAT--IPITGTGINPARSLGAAIIYNTDQA
NtTIPa       -TLANGVSYG---------QGIIMEVILTFSLLFTVYTTIVDPKKGI------LEGMGPLLTGLVVGANIMAGG--PFSGASMNPARSFGPAFVSG---I
BpeTIP1;1    -SLSSGVGVW---------NAFVLEIVMTFGLVYTVYATAVDPKRGS------VGVIAPIAIGFIVGANILAGG--AFDGASMNPAVSFGPALVSW---T
BpeTIP1;2    -SLSSDVSVW---------NAVVLEIVMTFGLVYTVYATAVDPKKGN------IGILAPIAIGLIVGANILAGG--AFDGASMNPAVSFGPAVVSW---S
BpeTIP1;3    -SLSSGVSVW---------NAVVFEIVMTFGLVYTVYATAVDPKKGN------VGTVAPIAIGFIVGANILVGG--AFDGASMNPAVSFGPAVVSW---S
BpeTIP3;1    -YVTSGVGEW---------HGLIFEIVMTFTLVYTVFATAIDPKRGS------LGTIAPLAIGFIVGANILAGG--PFDGACMNPARAFGPALVGW---R
BpeTIP2;1    -SVASGVGAI---------EGVVMEIIITFALVYTVYATAADPKKGS------LGTIAPIAIGFIVGANILAAG--PFSGGSMNPARSFGPAVASG---D
BpeTIP2;2    -GVASGMNAV---------EGVVMEIVITFALVYTVYATAADPKKGS------LGIIAPIAIGFIVGANILAAG--PFSGGSMNPARSFGPAVVSG---D
BpeTIP4;1    -SLASGVGYL---------QGVIWEVILTFSLLFTVYATIVDPKKGA------LDGLGPTLTGFVVGANILAGG--AFSGASMNPARSFGPALISG---D
BpeTIP5;1    -VIAEEMTGF---------GASLMEGVLTFALVYTVYA-AGDTRRGT------MGAIGPIAIGSIAGANVLAAG--PFSGGSMNPACAFGSAVTAG---S
BpeXIP1;1    ISALNRGT-------------ALVLEFCCTFVLLFVGVTVAFDKKRCKELGLAVVCAVVAAAWALAVFVSITVTGQVGYAGVGLNPARCLGPALLHG-GVL
BpeXIP1;2    ISALSRGT-------------ALVLEFCCTFVVLFVGVTVAFDKKRCKELGLAVVCVVVAGAMALAVFVSITVTGQASYAGVGLNPARCLGPVLLQG-GVL
BpeXIP1;3    ISTLSRGT-------------ALVLEFCCTFVVLFVGVTVAFDKKRCKELGVAVVCVVVAGAMALAVFVSITVTGQAGYAGVGLNPARCLGPALLQG-GVL
BpeXIP2;1    LTVIAPGPNGPISVGLGTSQGLWLEIICTFVLLFASIWIALDHRQARALGRVIIFSVIGIVVGLLVFVSTTVTGAKGYAGAGMNPARCLGPAIVRG-GHL
```

NIP

```
110       120       130       140       150       160       170       180       190       200
                    |         |         |         |         |         |         |         |         |         |
NtAQP1       VMGLKRSDSLCSSVGIQGVAWAFGGMIFALVYCTAGISGGHINPAVTFGLFLARKLSLTRAIFYIVMQCLGAICGAGVVKGFMVGPYQRLGGG---ANVV
NtTIPa       AMAANKLNG-D--------AMAHALVVAVTISAGFRISGGHLNPAVTLGLCMGGHITVFRSILYWIDQLLASVAACALLNYLTAG----LETP---VHTL
AtTIP4;1      AMATDSLVG-NTLVGLFAVAVAHAFVVAVMISAG-HISGGHLNPAVTLGLLLGGHISVFRAFLYWIDQLLASSAACFLLSYLTGG----MGTP---VHTL
AtNIP6;1      AIVNQKTDGAETLIG---CAASAGLAVMIVILSTGHISGAHLNPAVTIAFAALKHFPWKHVPVYIGAQVMASVSAAFALKAVFEPTMSG--------GVT
AtNIP2;1      IAVNAQHNHVVTLVG---IAVVWGIVIMVLVYCLGHLS-AHFNPAVTLALASSQRFPLNQVPAYITVQVIGSTLASATLRLLFDLNNDVCSKKHDVFLGS
CpNIP1       AALNGSDAQRVSQLG---ASVAGGLIVTVMIYAVGHISGAHMNPAVTTAFAATRHFPWKQVPLYAAAQLSGATCAAFTLRLLLHPIKH--------LGTT
BpeNIP1;1     VAVNKIYGSV-TFPG---ICVTWGLIVTVMIYTVGHISGAHFNPAVTITSAIFRRFPYKEVPLYIAAQLMGSILASGTLALVFDVTPDA-------YFGT
BpeNIP4;1     VVVNKIYGSV-SFPG---ICVVWGLIVMVMIYSVGHISGAHFNPAVTITFAIFRRFPLKQVPLYIVAQMIGSTLASATLCALFPVDKKS-------FFGT
BpeNIP1;2     VVVNLGNDKVVTAPG---VSIVWGLAVMVLVYSVGHISGAHFNPAVTIAFATCRRFPWKQVPAYVSAQVLGSTMASGTLRLIFNGHENR-------FVGT
BpeNIP2;1     AALSAADENKVSKLA---ASVAGGLIVTVMIYAVGHISGAHMNPAVTLAFAAVRHFPWKQVPLYAAAQLAGAISAAFTLRVILNPIKE--------LGTT
BpeNIP5;1     PIVNQKYNGAESLIG---NAACAGLAVMVVILSTGHISGAHLNPSLTIAFATLRHFPWLQVPAYIAAQVSGSICASFALKGVFHPYMSG--------GVT
BpeNIP5;2     PIVNEKYQGAETLIG---SAACAGLAVMAMVLSTGHISGAHFNPSVTIAFAALRHFPWVQVPGYLVAQVSASICAAFTLKGVFHPFMSG--------GVT
BpeNIP6;1     AMVNQKTHGSETLLG---CAASSGLAVMVVILSTGHISGAHLNPALTIAFAALKHFPWKHVPMYIGAQVVASFCAAFALKGIFHPIMGG--------GVT
BpeNIP7;1     IACTQLSKGEVGLLE---YAATAGLTVVVVIFSIGHISCAHVNPAVTIAFATFGHFPWSKVPLYILAQTLGSVLATCIGQSVYDVKSE--------FMTT

210       220       230       240       250       260       270       280       290       300
                    |         |         |         |         |         |         |         |         |         |
NtAQP1       NHGYTKGDGLGAEIIGTFVLVYTVFSA-TDAKRNARDSYVPILAPLPIGFAVFLVHLATIPITGTGINPARSLGAAIIYNTDQAWDDHWIFWVGPFIGAA
NtTIPa       ANGVSYGQGIIMEVILTFSLLFTVYTTIVDPKKGILEGMGPLLTGLVVGANI----MAGGPFSGASMNPARSFGPAFVSGI---WTDHWVYWVGPLIGGG
AtTIP4;1      ASGVSYTQGIIWEIILTFSLLFTVYATIVDPKKGSLDGFGPLLTGFVVGANI----LAGGAFSGASMNPARSFGPALVSGN---WTDHWVYWVGPLIGGG
AtNIP2;1      SPSGSDLQAFVMEFIITGFLMLVVCAVTTTKRT------TEELEGLIIGATVTLNVIFAGEVSGASMNPARSIGPALVWGC---YKGIWIYLLAPTLGAV
AtNIP6;1      VPTVGLSQAFALEFIISFNLMFVVTAVATDTRA------VGELAGIAVGATVMLNILIAGPATSASMNPVRTLGPAIAANN---YRAIWVYLTAPILGAL
CpNIP1       TPSGSDLQALVMEIVVTFSMMFVTCAVATDTKA------VGELAGLAVGSAVCITSILAGPVSGGSMNPVRTLGPAMASDN---YKGLWVYFVGPVTGTL
BpeNIP1;1     VPTGSNSQSLVLEILCSFLLMFVISGVTTDSRA------ISEVAGIAVGMTILLNVFIAGPVSGASMNPARSIRPAVIKHV---YKGLWVYIVGPIIGTI
BpeNIP4;1     APVGSNFQALIIEIIISFLLMFVICGVATDNRA------IGELAGIAVGMTITLNVFVAGPVSGASMNPARSIGPALLTHM---YKGLWIYILGPLAGTI
BpeNIP1;2     IPAGSNLQSFALEFIITFYLMFVISGVATDNRA------IGELAGLAVGSTILLNVMFAGPISGASMNPARSLGPAIVHNK---YESIWVYLVATTLGAI
BpeNIP2;1     SPTGTEIEALVMEIVVTFTMMFTASAVATDTKA------IGELAGLAVGSAVCITSILAGPISGGSMNPARTIGPAIASAS---YKGIWVYLVGPVTGTL
BpeNIP5;1     VPSVTEGQAFALEFIITFNLLFVVTAVATDTRA------VGELAGIAVGATVLVNILVAGPSSGGSMNPVRTLGPAIAAGN---YKSLWIYLVAPTLGAL
BpeNIP5;2     VPAVGIGQAFALEFIVTFTLLFVITAVATDTCA------VGEFAGIAIGGTVMLNILVAGTTTGASMNPVRTIGPAIAAGT---YKGFWIYMVAPILGAL
BpeNIP6;1     VPSGSYGQAFALEFIISFNLMFVVTAVATDTRA------VGELAGIAVGATVMLNILIAGPTTGASMNPVRTLGPAVAANN---YKAIWVYLTAPILGAL
BpeNIP7;1     RPLRGCSSAFWVELIATFIIMFLAASVTYQHQS------VGHLSGFVVGIAISL----AVPVSGGSMNPARSLGPAIISGK---FNDIWIYMTAPTIGAV
```
