# Supplementary material for: Genome-Wide Identification, Structure Characterization, and Expression Pattern Profiling of the Aquaporin Gene Family in Betula pendula
Source: Int J Mol Sci. 2021 Jul 6;22(14):7269. doi: 10.3390/ijms22147269 (PMC8304918; doi:10.3390/ijms22147269)
Supplement: Supplementary file 1 [file ijms-22-07269-s001.zip › Supplementary Files Betula MIPs/FigureS8.pdf]

Figure S8 Predicted subcellular localization of *Betula pendula* aquaporins.
Predictions were made by using WolF PSORT server.
Abbreviations of protein localization sites in the dataset are as follows: **Plas**, plasma membrane; **ER**, Endoplasmic reticulum; **Gol**, Golgi apparatus; **Pero**, peroxisomes; **Chlo**, chloroplasts; **Mito**, mitochondria; **Vac**, Vacuole. The number mentioned besides localization roughly indicates the number of the nearest neighbors to the query which localizes to each site, but are adjusted to account for the possibility of dual localization. The strongest occurrences are highlighted in bold.

*Bpe*PIP1;1	**plas**: 14
*Bpe*PIP1;2	**plas**: 11, vac: 3
*Bpe*PIP1;3	**plas**: 13, vac: 1
*Bpe*PIP1;4	**plas**: 13, vac: 1
*Bpe*PIP2;1	**plas**: 12, vac: 2
*Bpe*PIP2;2	**plas**: 10, vac: 4
*Bpe*PIP2;3	**plas**: 13, vac: 1
*Bpe*PIP2;4	**plas**: 12, vac: 1, ER: 1
*Bpe*PIP2;5	**plas**: 14
*Bpe*PIP2;6	**plas**: 12, E.R.: 1, gol: 1
*Bpe*TIP1;1	**plas**: 8, vac: 6
*Bpe*TIP1;2	**vac**: 9, plas: 5
*Bpe*TIP1;3	**plas**: 8, vac: 6
*Bpe*TIP2;1	**plas**: 8, vac: 6
*Bpe*TIP2;2	**vac**: 8, plas: 6
*Bpe*TIP3;1	**plas**: 7, vac: 6, ER: 1
*Bpe*TIP4;1	**vac**: 11, plas: 2
*Bpe*TIP5;1	**chlo**: 14
*Bpe*XIP2;1	**vac**: 11, plas: 2, ER: 1
*Bpe*XIP1;1	**plas**: 6, ER: 3, vac: 2, chlo: 1, pero: 1, gol: 1
*Bpe*XIP1;2	**plas**: 9, vac: 4, E.R.: 1
*Bpe*XIP2-like	**vac**: 7, plas: 5, gol: 2
*Bpe*XIP1;3	**plas**: 6, ER: 4, vac: 2, chlo: 1, pero: 1
*Bpe*NIP1;1	**plas**: 9, vac: 5
*Bpe*NIP1;2	**plas**: 8, golg: 3, ER: 2, vac: 1
*Bpe*NIP2;1	**plas**: 9, vac: 4, E.R.: 1
*Bpe*NIP4;1	**plas**: 11, ER: 2, cyto: 1
*Bpe*NIP5;1	**vac**: 6, plas: 5, mito: 1, ER: 1
*Bpe*NIP5;2	**plas**: 9, vac: 5
*Bpe*NIP6;1	**plas**: 10, extr: 1, vac: 1, ER: 1, gol: 1
*Bpe*NIP7;1	**plas**: 7, ER: 3, vac: 2, gol: 2
*Bpe*SIP1;1	**vac**: 6, extr: 3, plas: 2, chlo: 1, ER: 1, gol: 1
*Bpe*SIP1;2	**plas**: 5, chlo: 3, gol: 2, vac: 1
*Bpe*SIP2;1	**vac**: 9, plas: 4, ER: 1
