# Supplementary material for: Genome-Wide Identification, Structure Characterization, and Expression Pattern Profiling of the Aquaporin Gene Family in Betula pendula
Source: Int J Mol Sci. 2021 Jul 6;22(14):7269. doi: 10.3390/ijms22147269 (PMC8304918; doi:10.3390/ijms22147269)
Supplement: Supplementary file 1 [file ijms-22-07269-s001.zip › Supplementary Files Betula MIPs/FigureS9.pdf]

Figure S9

	Low-temperature stress (6°C) on leaves							Organs			
	C	0.5hpt	1hpt	1.5hpt	2hpt	2.5hpt	3hpt	flower	leaf	root	xylem
BpePIP1;1	152	128	81	112	107	116	133	89	65	294	1352
BpePIP1;2	851	763	569	642	653	788	714	1045	879	839	846
BpePIP1;3	201	443	450	406	228	411	345	51	36	927	51
BpePIP1;4	106	96	58	75	91	87	105	366	295	198	935
BpePIP1-like	NA	NA	NA	NA	NA	NA	NA	NA	NA	NA	NA
BpePIP1-like	NA	NA	NA	NA	NA	NA	NA	NA	NA	NA	NA
BpePIP2;1	767	723	416	531	718	918	761	764	515	555	1004
BpePIP2;2	NA	NA	NA	NA	NA	NA	NA	NA	NA	NA	NA
BpePIP2;3	NA	NA	NA	NA	NA	NA	NA	NA	NA	43	38
BpePIP2;4	1553	1936	1081	1659	1476	1361	1864	725	730	280	19
BpePIP2;5	132	187	347	250	228	440	362	236	51	1958	387
BpePIP2;6	74	119	41	128	65	70	80	7	6	264	71
BpeTIP1;1	2825	1908	667	1884	2126	1834	1383	1165	1253	761	707
BpeTIP1;2	54	14	13	36	28	24	22	NA	3	888	2
BpeTIP1;3	52	52	15	51	16	13	12	7	17	268	3
BpeTIP2;1	122	105	77	111	93	133	94	58	101	26	140
BpeTIP2;2	NA	NA	NA	NA	NA	NA	NA	NA	NA	117	NA
BpeTIP3;1	NA	NA	NA	NA	NA	NA	NA	NA	NA	NA	5
BpeTIP4;1	25	35	22	32	35	45	38	15	4	26	133
BpeTIP5;1	NA	NA	NA	NA	NA	NA	NA	NA	NA	NA	NA
BpeXIP1;1	NA	2	2	1	NA	2	1	NA	NA	4	NA
BpeXIP1;2	1	2	3	1	1	2	1	2	NA	NA	NA
BpeXIP1;3	1	7	15	6	3	13	11	2	NA	NA	NA
BpeXIP2;1 *	84	87	35	86	82	70	86	57	37	NA	NA
BpeNIP1;1	NA	NA	NA	NA	NA	NA	NA	NA	NA	NA	NA
BpeNIP1;2	110	92	37	65	69	54	81	244	88	50	13
BpeNIP2;1	NA	NA	NA	NA	NA	NA	NA	NA	NA	NA	2
BpeNIP4;1	NA	NA	NA	NA	NA	NA	NA	NA	NA	NA	NA
BpeNIP5;1	12	10	29	16	22	79	24	66	16	112	92
BpeNIP5;2	NA	NA	NA	NA	NA	NA	NA	NA	NA	NA	NA
BpeNIP6;1	3	NA	16	1	3	18	4	20	5	6	16
BpeNIP7;1	NA	NA	NA	NA	NA	NA	NA	NA	NA	NA	NA
BpeSIP1;1	67	73	76	63	62	81	78	130	103	105	95
BpeSIP1;2	41	44	16	20	28	16	14	60	39	56	16
BpeSIP2;1	26	29	49	34	31	37	45	35	13	62	12

0	250	500	750	1000	1250	1500	1750	2000	2250	2500	2750	3000

Figure S9. Trimmed mean of M-values (TMM) values related to the expression profiles of *BpeAQP* genes from *Betula pendula* in leaves under cold stress (6 °C), and in various organs (Flower, Leaf, Root and Xylem). The *Bpe*AQPs are grouped by subfamily. The horizontal color scales at the bottom of the Figure represent the normalized expression of the aquaporins in terms of Trimmed mean of M-values (TMM). Expression levels are displayed by the depth of red and yellow. hpt, hours post treatment ; C, control sample which corresponds to the plants left at 25 °C. PIP, Plasma Intrinsic Protein ; TIP, Tonoplast Intrinsic Protein ; XIP, *X*-Intrinsic Protein ; NIP, Nodulin-like Intrinsic Protein ; SIP, small basic intrinsic protein. NA, TMM non detectable.
