# Supplementary material for: Genome-Wide Identification, Structure Characterization, and Expression Pattern Profiling of the Aquaporin Gene Family in Betula pendula
Source: Int J Mol Sci. 2021 Jul 6;22(14):7269. doi: 10.3390/ijms22147269 (PMC8304918; doi:10.3390/ijms22147269)
Supplement: Supplementary file 1 [file ijms-22-07269-s001.zip › Supplementary Files Betula MIPs/TableS1.pdf]

Table S1. Nomenclature of the *Bpe*AQPs from *Betula pendula.*
Genomic, CDS and protein sequences are detailled in FigureS1

	Name	CoGe	Localization	Nucleotide location	NCBI
**PIP1**	***Bpe*PIP1;1**	Bpev01.c0190.g0072.m0001	Contig190	850,536-851,776	FXXK01000191.1
	***Bpe*PIP1;2**	Bpev01.c0170.g0038.m0001	Chr14	5,426,780-5,430,337	FXXK01000171.1
	***Bpe*PIP1;3**	Bpev01.c1699.g0001.m0001	Chr5	1,627,018-1,628,297	FXXK01001700.1
	***Bpe*PIP1;4**	Bpev01.c0027.g0083.m0001	Chr5	22,880,015-22,885,763	FXXK01000028.1
	***Bpe*PIP1-like**	Bpev01.c0379.g0007.m0001	Chr14	13,436,305-13,436,932	FXXK01000380.1
	***Bpe*PIP1-like**	Bpev01.c0007.g0002.m0001	Chr5	15,083,781-15,094,079	FXXK01000008.1
**PIP2**	***Bpe*PIP2;1**	Bpev01.c0658.g0014.m0001	Chr3	19090629-19091774	FXXK01000659.1
	***Bpe*PIP2;2**	Bpev01.c0577.g0022.m0001	Chr9	1,816,676-1,817,768	FXXK01000578.1a
	***Bpe*PIP2;3**	Bpev01.c0577.g0023.m0001	Chr9	1,830,657-1,832,603	FXXK01000578.1b
	***Bpe*PIP2;4**	Bpev01.c0042.g0019.m0001	Chr5	4,990,578-4,993,085	FXXK01000043.1
	***Bpe*PIP2;5**	Bpev01.c0552.g0003.m0001	Chr13	14,364,373-14,366,680	FXXK01000553.1
	***Bpe*PIP2;6**	Bpev01.c0483.g0002.m0001	Chr8	4,856,860-4,858,776	FXXK01000484.1
**TIP**	***Bpe*TIP1;1**	Bpev01.c0278.g0002.m0001	Chr8	6,808,526-6,809,382	FXXK01000279.1
	***Bpe*TIP1;2**	Bpev01.c0396.g0015.m0001	Chr13	17,015,493-17,016,481	FXXK01000397.1
	***Bpe*TIP1;3**	Bpev01.c2330.g0012.m0001	Chr5	3,465,120-3,466,550	FXXK01002331.1
	***Bpe*TIP2;1**	Bpev01.c0665.g0011.m0001	Chr5	13,209,768-13,211,363	FXXK01000666.1
	***Bpe*TIP2;2**	Bpev01.c0120.g0064.m0001	Chr14	4,812,457-4,815,151	FXXK01000121.1
	***Bpe*TIP3;1**	Bpev01.c1026.g0003.m0001	Chr7	20,005,317-20,006,689	FXXK01001027.1
	***Bpe*TIP4;1**	Bpev01.c0921.g0003.m0001	Chr19	15,031,065-15,032,014	FXXK01000922.1
	***Bpe*TIP5;1**	Bpev01.c0477.g0022.m0001	Contig477	209,208-211,230	FXXK01000478.1
**XIP**	***Bpe*XIP1;1**	Bpev01.c1577.g0026.m0001	Chr3	19,989,890-19,991,672	FXXK01001578.1b
	***Bpe*XIP2-like**	Bpev01.c1577.g0027.m0001	Chr3	19,966,597-19,968,432	FXXK01001578.1a
	***Bpe*XIP1;2**	Bpev01.c1577.g0028.m0001	Chr3	19,966,597-19,968,432	FXXK01001578.1c
	***Bpe*XIP2;1**	Bpev01.c2937.g0004.m0001	Contig2937	22,352-23,164	FXXK01002938.1a
	***Bpe*XIP1;3**	Bpev01.c2937.g0004.m0001	Contig2937	27,828-30,813	FXXK01002938.1b
**NIP**	***Bpe*NIP1;1**	Bpev01.c0145.g0009.m0001	Chr2	8,012,612-8,018,807	FXXK01000146.1
	***Bpe*NIP1;2**	Bpev01.c0230.g0002.m0001	Contig230	17,343-19,985	FXXK01000231.1
	***Bpe*NIP2;1**	Bpev01.c0281.g0063.m0001	Chr2	4,593,561-4,596,959	FXXK01000282.1
	***Bpe*NIP4;1**	Bpev01.c1162.g0002.m0001	Chr1	16,645,942-16,653,541	FXXK01001163.1
	***Bpe*NIP5;1**	Bpev01.c1045.g0004.m0001	Chr11	981,169-985,394	FXXK01001046.1
	***Bpe*NIP5;2**	Bpev01.c1084.g0010.m0001	Chr6	19,692,291-19,696,739	FXXK01001085.1
	***Bpe*NIP6;1**	Bpev01.c0044.g0051.m0001	Chr5	8,366,764-8,370,948	FXXK01000045.1
	***Bpe*NIP7;1**	Bpev01.c0330.g0006.m0001	Chr1	34,485,381-34,487,761	FXXK01000331.1
**SIP**	***Bpe*SIP1;1**	Bpev01.c0082.g0002.m0001	Chr8	23,172,529-23,181,063	FXXK01000083.1
	***Bpe*SIP1;2**	Bpev01.c0387.g0011.m0001	Contig387	91,110-91,829	FXXK01000388.1
	***Bpe*SIP2;1**	Bpev01.c0212.g0010.m0001	Chr8	12,136,640-12,142,650	FXXK01000213.1

("Pseudogenes/truncated sequences" are in red)
