# Supplementary figures and images for: Genome-Wide Identification, Structure Characterization, and Expression Pattern Profiling of the Aquaporin Gene Family in Betula pendula
Source: Int J Mol Sci. 2021 Jul 6;22(14):7269. doi: 10.3390/ijms22147269 (PMC8304918; doi:10.3390/ijms22147269)

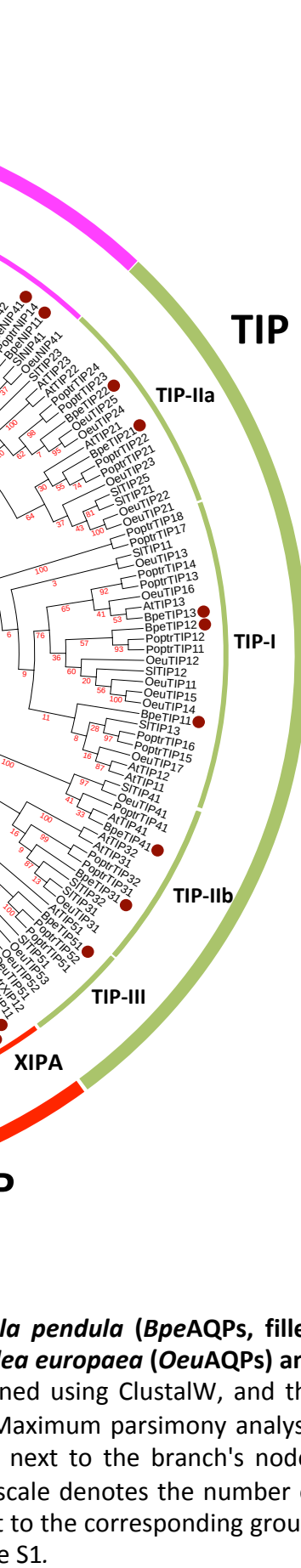[illegible]

Supplement: Supplementary file 1 [file ijms-22-07269-s001.zip › Supplementary Files Betula MIPs/FigureS2.pdf]
